# Tritylamine as an Ammonia Surrogate in the Ugi Reaction Provides Access to Unprecedented 5-Sulfamido Oxazoles Using Burgess-type Reagents

**DOI:** 10.1021/acs.orglett.1c01002

**Published:** 2021-04-29

**Authors:** Irene Preet Bhela, Marta Serafini, Erika Del Grosso, Gian Cesare Tron, Tracey Pirali

**Affiliations:** † Dipartimento di Scienze del Farmaco, Università del Piemonte Orientale, Largo Donegani 2, Novara 28100, Italy; ‡ ChemICare S.r.l., Enne3, Corso Trieste 15/A, Novara 28100, Italy

## Abstract

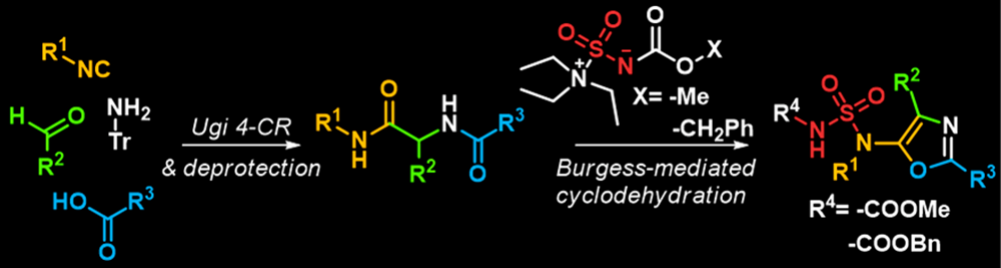

Starting from a wide range of α-acylamino amide substructures synthesized using tritylamine as an ammonia surrogate in the Ugi reaction, Burgess-type reagents enable cyclodehydration and afford unprecedented oxazole scaffolds with four points of diversity, including a sulfamide moiety in the 5-position. The synthetic procedure employs readily available starting materials and proceeds smoothly under mild reaction conditions with good tolerance for a variety of functional groups, coming to fill a gap in the field of oxazole compounds.

Oxazoles are heterocycles extensively used by medicinal chemists not only as pharmacophoric groups *per se* but also as structural elements that impose a degree of conformational restriction.^1^ Moreover, because of the similarity in the electronic properties, they behave as isosteres of amides, endowed with a better chemical and metabolic stability along with an acceptable oral bioavailability.^2^ Oxazoles also find wide application in medicinal chemistry for the design of peptidomimetic structures.^3^ Finally, their inclination to undergo cycloaddition transformations as azadienes is often exploited for generating new molecular scaffolds.^4^ It is therefore not surprising that the development of efficient methodologies for access to this high-value substructure has drawn considerable attention over the years.

Several procedures have been reported to generate oxazoles bearing a point of diversity in the 5-position. For example, 5-aminooxazoles displaying a tertiary nitrogen at the 5-position can be prepared by the multicomponent reaction (MCR) reported by Zhu et al., among aldehydes, amines, and tertiary α-isocyanoacetamides promoted by ammonium chloride (Figure 1).^5^ The most common methodology available for the preparation of 5-aminooxazoles bearing a secondary nitrogen at the 5-position is represented by the trifluoroacetic acid (TFA)/trifluoroacetic anhydride (TFAA)-mediated cyclodehydration of diamide/dipeptide precursors. In this regard, 10 years after the pioneering work of Fleury and coworkers that reported in 1973 the cyclization of α-acyl amino acids to give 5-(acetamido)oxazoles,^6^ Lipshutz described the cyclization of α-acylamino amides to give 5-(trifluoroacetamido)oxazoles (Figure 1).^7^ Other examples emerged from the literature, but the preparation of the substrates usually required lengthy routes that included protection and deprotection steps.^8^ A step forward was taken in 2009 by Thompson et al. that described the preparation of the diamide precursor by an Ugi reaction in trifluoroethanol followed by cyclization in the presence of TFA/TFAA to give 5-(trifluoroacetamido)-oxazoles (Figure 1).^9^ Further cyclization procedures have been reported, including the rhodium-catalyzed reaction between a diazocompound and a protected l-leucinamide followed by an I_2_/PhP_3_-mediated cyclization (Figure 1),^3^ the coupling of two molecules of isocyanides with carboxylic acid promoted by zinc bromide,^10^ the [4 + 1] cycloaddition between an isocyanide and an *N*-acylimine,^11^ and the Cp*Co(III)-catalyzed reaction between an *N*-(pivaloyloxy)amide and an ynamide (Figure 1),^12^ but challenges still remain with regard to limitations in the range of applicable substrates and the reaction efficiency.

**Figure 1:**
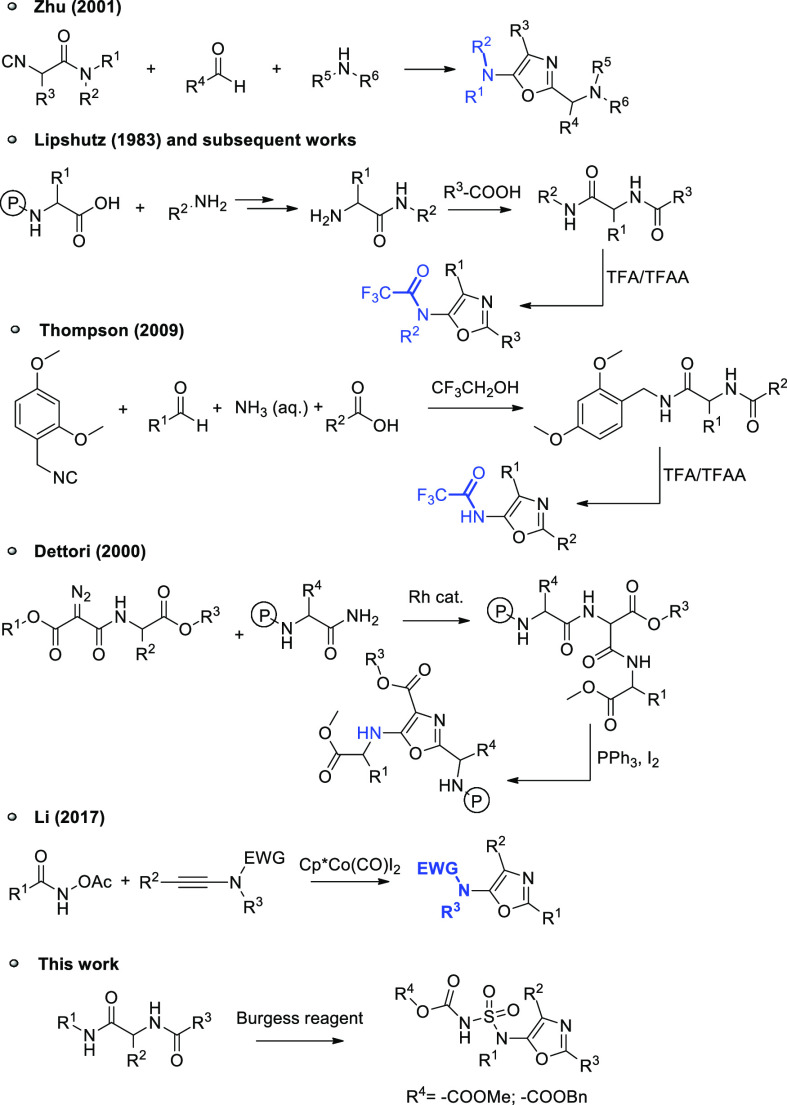
Synthetic approaches to 5-substituted oxazoles.

None of the aforementioned cyclodehydrations allows the insertion of a sulfamide moiety in the 5-position of the oxazole ring. Herein we disclose a reaction in which the use of Burgess-type reagents^13^ leads to the simultaneous formation of the oxazole ring and insertion of a sulfamide group on the heterocyclic system.

In addition to being a powerful dehydrating agent, the Burgess reagent has also been described for its ability to mediate the synthesis of sulfamidates, epoxyalcohols, α- and β-glycosilamines, and cyclic sulfamides. Taking inspiration from the versatile applications of the Burgess reagent, we decided to investigate its dual nature as both a dehydrative and a nondehydrative reagent in a single modality, and we speculated that the application of this reagent to a diamide substructure could result in the formation of oxazoles bearing an *N*,*N*′-unsimmetrical sulfamide at position 5.^14^ To verify our hypothesis, we initially performed a prototype reaction of the diamide precursor **1a** (1 equiv) in the presence of an excess of Burgess reagent (2 equiv) in dry tetrahydrofuran (THF) at reflux. Gratifyingly, we observed the formation of 5-sulfamido oxazole **2a**, even if in moderate yield (40%, Table 1, entry 3).

**Table 1:**
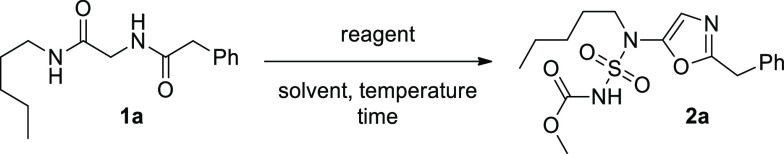
Screening of Conditions for the Cyclization Reaction

entry	reagent	solvent	temp (°C)	time (h)	yield (%)
**1**	Burgess (3 equiv)	dry THF	66	1	34^a^
**2**	Burgess (3 equiv)	dry THF	66	5	26^a^
**3**	Burgess (2 equiv)	dry THF	66	1	40^a^
**4**	Burgess (1 equiv)	dry THF	40	1	trace^b^
**5**	Burgess (3 equiv)	dry CH_2_Cl_2_	40	1	59^a^
**6**	**Burgess (2 equiv)**	**dry CH**_**2**_**Cl**_**2**_	**40**	**1**	**71**^a^
**7**	Burgess (1 equiv)	dry CH_2_Cl_2_	40	1	20^a^

a Yields based on isolated product after gravimetric chromatography are given.

b Based on TLC.

Prompted by the challenge to expand the chemical space around oxazoles, we further optimized the reaction conditions, as summarized in Table 1.

During the optimization process, it was clear that neither a higher temperature nor a prolonged reaction time favors the formation of the product. In particular, after 1 h, the starting material has usually reacted completely, with the exception of those reactions in which 1 equiv of Burgess reagent is used (entries 4 and 7). Regarding the Burgess reagent, the highest yield is achieved using 2 equiv, a result in accordance with the proposed reaction mechanism, summarized in Scheme 1. The reaction between the oxygen of the amide and the Burgess reagent gives intermediate **4** (pathway A, Scheme 1), which is then intramolecularly intercepted by the second amide oxygen to afford intermediate **5**. After an irreversible intramolecular E2 elimination, intermediate **5** restores the aromaticity of oxazole to give the 5-aminooxazole **7**. It is reasonable that a competing mechanism, triggered by the reaction between the Burgess reagent and the oxygen of the other amide, can take place (pathway B, Scheme 1). It should be noted that intermediate **7** can not be isolated even if 1 equiv of Burgess reagent is used, suggesting that once formed, it immediately attacks a second molecule of the Burgess reagent, giving the corresponding 5-sulfamido oxazole **2** (Scheme 1).

**Scheme 1:**
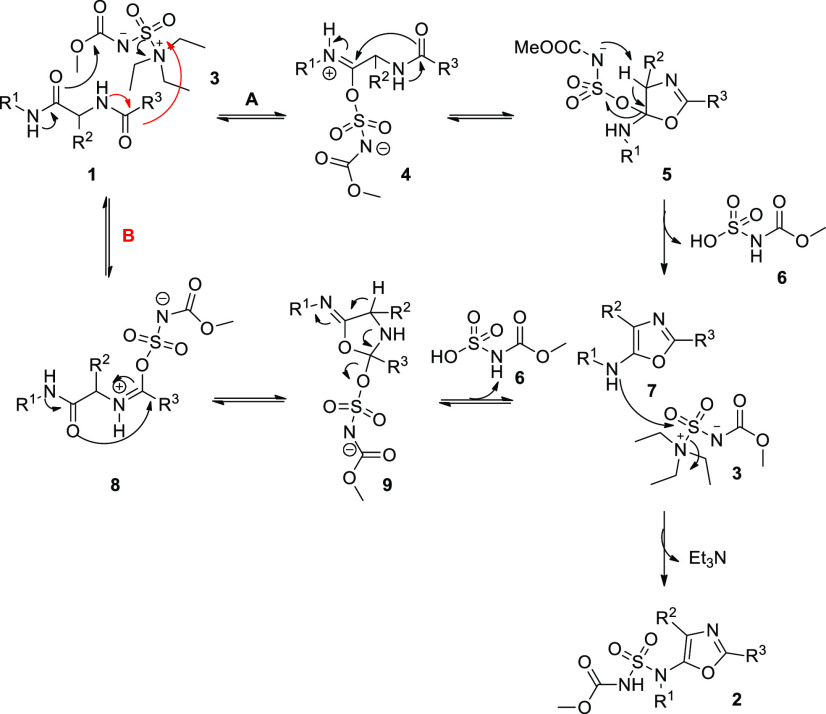
Proposed Mechanism for the Dehydration of Diamide **5** by Means of the Burgess Reagent

Once the optimal conditions had been established, a library of diamide precursors was synthesized. From our experience in the field of MCRs,^15^ we assumed that the simplest procedure to afford the required diamide substructures was represented by the Ugi 4-component reaction. However, when this MCR is conducted in the presence of ammonia it is known that yields are poor, especially when formaldehyde is used as an oxo reactive partner, due to the formation of side products.^16^ This limitation was evident when, during a medicinal chemistry campaign aimed at identifying novel IDO1 inhibitors,^17^ two compounds, **1o** and **1p** (Scheme 2), bearing a diamide substructure were required for our structure–activity relationship study. Indeed, the Ugi MCR afforded the two compounds in poor yields, with the use of either ammonia or one of its surrogates,^18^ for example, 2,5-dimethoxy benzylamine, as described by Thompson et al. (Figure 1).^9^

**Scheme 2:**
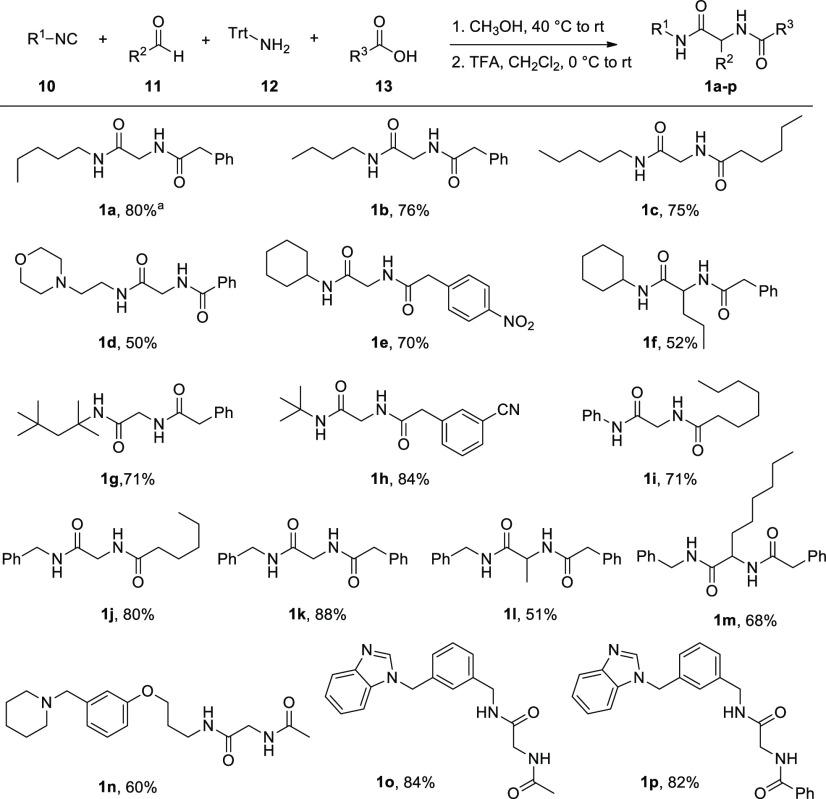
Diamide Starting Materials This reaction was scaled up to 6 mmol of **10a** with a yield of 80%. Reaction conditions: (1) **10** (0.8 mmol, 1 equiv), **11** (0.8 mmol, 1 equiv; in the case of **11a**: 1.6 mmol, 2 equiv), **12** (0.8 mmol, 1 equiv), **13** (0.8 mmol, 1 equiv), CH_3_OH (1.5 mL), 40 °C for 40 min, rt overnight. (2) TFA (3.4 mL), CH_2_Cl_2_ (3.4 mL), 0 °C for 30 min, rt for 3 h. Yields based on isolated product after gravimetric chromatography are given.

To circumvent this limitation, we investigated the use of tritylamine as an amine component in the Ugi reaction. Despite its steric hindrance, tritylamine was reported by Dömling to be an efficient and easily cleavable surrogate of ammonia in a modified version of the Ugi tetrazole synthesis to afford α-aminotetrazoles,^19^ but, surprisingly, to the best of our knowledge, this amine had never been applied in a classical Ugi reaction. First of all, an Ugi MCR was performed under classical conditions, and isocyanide **10**, formaldehyde **11a**, tritylamine **12**, and acetic acid **13o** reacted together in methanol, affording an intermediate that was next deprotected using TFA in CH_2_Cl_2_ at 0 °C to cleave the trityl group. With this approach, compound **1o** was obtained in one pot in a yield of 84%. Similarly, compound **1p** was synthesized using benzoic acid under the same conditions in 82% of yield.

This straightforward approach was applied to different isocyanides to investigate the scope of the reaction. As shown in Scheme 2, primary (**1a**–**d**, **1n**), secondary (**1e**, **1f**), tertiary (**1g**, **1h**), aromatic (**1i**), and benzylic (**1j**–**m**, **1o**,**p**) isocyanides are well tolerated, leading to the corresponding products in high yields. Carboxylic acids bearing different functional groups, such as nitro (**1e**) and nitrile (**1h**), resulted in being well tolerated, whereas the reactivity of our MCR was highly influenced by the nature of the carbonyl components. Indeed, linear aldehydes (**1f**, **1l**,**m**) lead to good yields, but more sterically hindered reagents, such as aromatic aldehydes or ketones, did not provide the corresponding compounds (data not shown).

With these Ugi precursors in hand, we next performed the cyclodehydration step under the previously optimized reaction conditions. Before setting up the cyclodehydrations, the Ugi precursors were purified by column chromatography, as the reaction mixture resulting from the Ugi reaction and the trityl deprotection usually presents several byproducts. The corresponding *N*,*N*′-disubstituted 5-sulfamido oxazoles **2a**–**o** were obtained in good to excellent yields (Scheme 3). Low yields were observed when the oxazole displayed a hindered substituent at position 4 (**2f**).

**Scheme 3:**
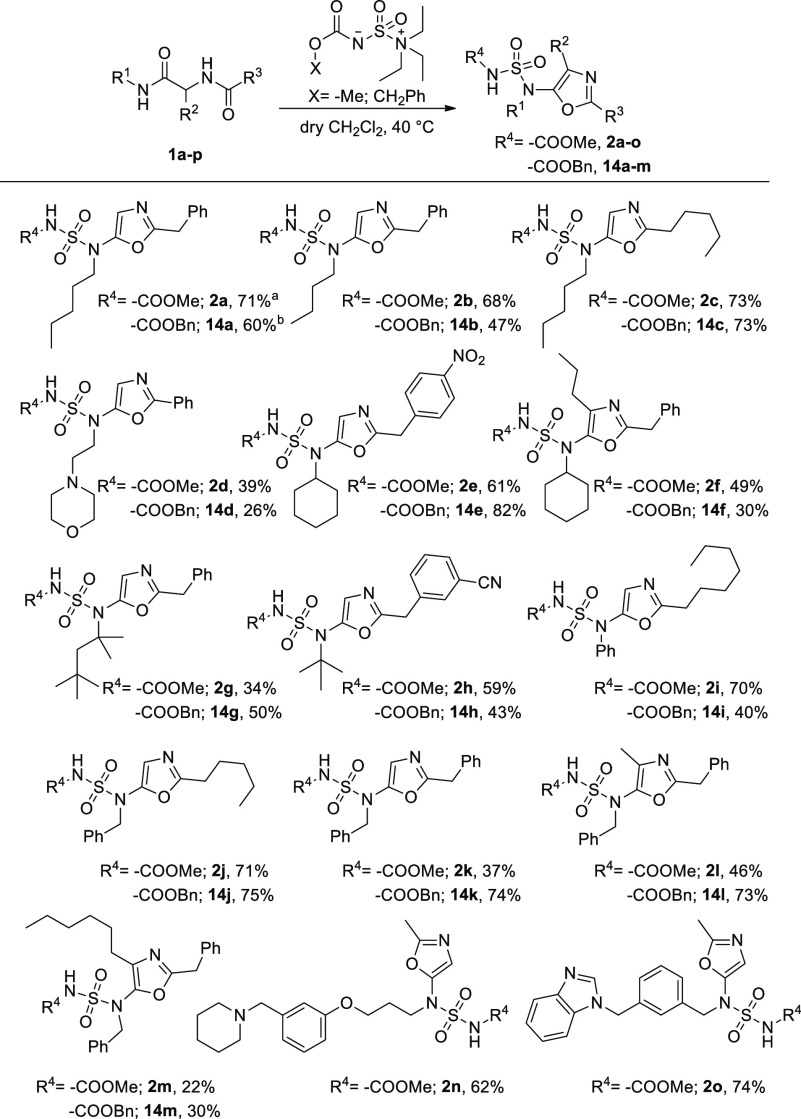
Synthesized 5-Sulfamido Oxazoles This reaction was scaled up to 4.5 mmol of **1a** with a yield of 87%. This reaction was scaled up to 3 mmol of **1a** with a yield of 68%. Reaction conditions: **1** (0.4 mmol, 1 equiv), Burgess reagent (0.8 mmol, 2 equiv), dry CH_2_Cl_2_ (1.4 mL), 40 °C, 1 h. Yields based on isolated product after gravimetric chromatography are given.

Finally, we envisaged the opportunity of using a modified version of the Burgess reagent^14,20^ bearing a benzyl group: The cyclization under the same conditions yielded products **14a**–**m**, which can free the amino group under hydrogenolysis. This is exemplified by the results reported in Scheme 4: Seven products (**14a**,**b**, **14f**, **14h**, **14j**–**l**) in the presence of H_2_ and Pd/C reacted to afford the corresponding 5-sulfamido oxazoles (**15a**,**b**, **15f**, **15h**, **15j**–**l**). Compounds **15h**, **15k**, and **15l** were afforded in only low yields due to the decomposition of the corresponding starting materials. In particular, the deprotection of compounds **15k** and **15l** leads to the formation of a byproduct corresponding to the monosubstituted 5-sulfamido oxazole in which the second nitrogen bound to the oxazole ring loses the benzyl group.

**Scheme 4:**
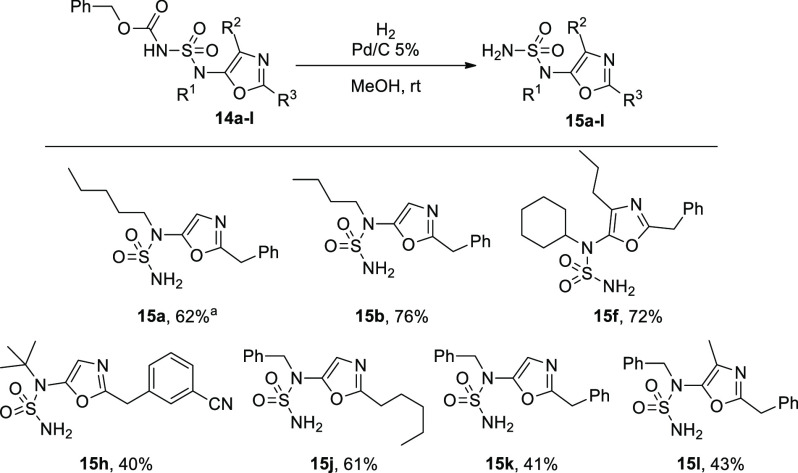
Deprotection of 5-Sulfamido Oxazoles to Afford Monosubstituted 5-Sulfamido Oxazoles This reaction was scaled up to 1 mmol of **14a** with a yield of 56%. Reaction conditions: **14** (0.10 mmol, 1 equiv), Pd/C 5% (18 mg), MeOH (0.6 mL), rt, 1 h. Yields based on isolated product after gravimetric chromatography are given.

In summary, the Ugi reaction mediated by tritylamine as a convenient ammonia surrogate leads to diamide products that, through cyclodehydration triggered by Burgess-type reagents, are transformed into unprecedented oxazoles bearing *N*,*N*′-disubstituted sulfamides at the 5-position. When displaying the benzyl group, the obtained products undergo hydrogenolysis, yielding monosubstitued 5-sulfamido oxazoles. Overall, the sequential synthesis proceeds smoothly and cleanly under mild reaction conditions, is scalable up to 1 mmol, provides high yields, and displays good tolerance to a variety of functional groups, coming to fill a gap in the preparation of both Ugi products and oxazoles.

The reported methodology provides a means for making unique heterocyclic sulfamides. The sulfamide moiety has recently drawn attention^21^ and received increasing acceptance in medicinal chemistry due to its potential to form polar interactions with proteins of interest^22^ and due to the tetrahedral nature of the sulfur atom, which provides an additional dimension for target recognition. Because it is still fairly under-represented, the sulfamide is an attractive means of decorating biologically active compounds while conferring novelty from a patent perspective. Furthermore, it is a bioisostere of urea, carbamate, and sulfonamide that has found wide application in the field of antibacterial agents, as exemplified by doripenem,^23^ a carbapenem approved by the United States Food and Drug Administration (FDA) in 2007, in the endothelin receptor antagonist macitentan,^24^ approved in 2013 for the treatment of pulmonary arterial hypertension, and in the IDO1 inhibitor epacadostat.^25^ We therefore foresee that the reported sequence will find wide application in drug discovery campaigns in the near future.

## Supporting Information Available

General experimental procedures, characterization data, and copies of ^1^H NMR and ^13^C NMR spectra(PDF)
