# Supplementary material for: Tritylamine as an Ammonia Surrogate in the Ugi Reaction Provides Access to Unprecedented 5-Sulfamido Oxazoles Using Burgess-type Reagents
Source: Org Lett. 2021 Apr 29;23(9):3610–4. doi: 10.1021/acs.orglett.1c01002 (PMC8289289; doi:10.1021/acs.orglett.1c01002)
Supplement: Supplementary file 1 — ol1c01002_si_001.pdf [file ol1c01002_si_001.pdf]

SUPPORTING INFORMATION

Tritylamine as an ammonia surrogate in Ugi reaction provides access to unprecedented 5-sulfamido oxazoles using Burgess-type reagents

Irene Preet Bhela, Marta Serafini, Erika Del Grosso, Gian Cesare Tron and Tracey Pirali*

Dipartimento di Scienze del Farmaco, Università del Piemonte Orientale; Novara, 28100; Italy

Table of contents

Experimental procedures and spectra data S2

Copies of ^{1}H and 13C spectra S26

References S78

Experimental procedures and spectra data

General experimental methods

Commercially available reagents and solvents were used as purchased without further purification with the exception of phenyl isocyanide,[1] 1-(3-(isocyanomethyl)benzyl)-1*H*-benzo[*d*]imidazole,[2] 1-(3-(3-isocyanopropoxy)benzyl)piperidine[3] and the modified Burgess reagent,[4] synthesized with the procedure reported in literature. When needed, solvents were distilled and stored on molecular sieves. Column chromatography was performed on silica gel. Thin layer chromatography (TLC) was carried out on 5 cm × 20 cm plates with a layer thickness of 0.25 mm. When necessary, TLC plates were visualized with aqueous KMnO4 or with Erlich solution. Melting points were determined in open glass capillary with a Stuart scientific SMP3 apparatus. All the target compounds were checked by IR (FT-IR Bruker Alpha II), ^{1}H-NMR and ^{13}C-NMR (Bruker Avance Neo 400 MHz), mass spectrometry (Thermo Finningan LCQ-deca XP-plus) equipped with an ESI source and an ion trap detector and HRMS (Thermo Fisher Q-Exactive Plus) equipped with an Orbitrap (ion trap) mass analyzer. Chemical shifts are reported in parts per million (ppm).

General procedure A for the synthesis of compounds 1a-1p.

A solution of tritylamine **12** (1 equiv, 0.80 mmol) and aldehyde **11** (1 equiv, 0.80 mmol) in CH_3OH (1.5 mL) was stirred at 40 °C in an oil bath for one hour. Isocyanide **10** (1 equiv, 0.80 mmol) and carboxylic acid **13** (1 equiv, 0.80 mmol) were then added at room temperature and the resulting mixture was stirred overnight. The next day the volatile was removed under vacuo and the crude was solubilized in CH_2Cl_2 (3.4 mL). At 0 °C, CF_3COOH (3.4 mL) was added and after 30 minutes the mixture was allowed to reach rt and stirred for 3 h. Then, the reaction was alkalized until pH 8-9 with NaOH 2M. The aqueous layer was extracted with CH_2Cl_2 (x2) and organic layers were dried over sodium sulfate and evaporated. The crude product was purified by column chromatography.
The present reaction was also scaled up to 6 mmol as reported for compound **1a**.

***N*-(2-oxo-2-(pentylamino)ethyl)-2-phenylacetamide, (1a).**

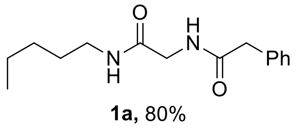

1a, 80%

The title compound was synthesized following the general procedure A. The crude material was purified by column chromatography using PE/EtOAc 4:6, affording compound **1a** (1.3 g, 4.79 mmol,

80%) a white solid. Mp 139-140 °C; ^{1}H-NMR (400 MHz; $CDCl_3$): δ 7.28-7.24 (m, 2H), 7.22-7.18 (m, 3H), 6.56 (br s, 1H), 6.43 (br s, 1H), 3.79 (d, *J* = 5.2 Hz, 2H), 3.51 (s, 2H), 3.10 (q, *J* = 6.1 Hz, 2H), 1.36 (quint, *J* = 6.1 Hz, 2H), 1.25-1.16 (m, 4H), 0.81 (t, *J* = 6.1 Hz, 3H). ^{13}C-NMR (101 MHz; $CDCl_3$): δ 171.7, 168.6, 134.6, 129.3, 129.0, 127.4, 43.5, 43.4, 39.6, 29.1, 29.0, 22.3, 13.9.
IR (neat): ṽ = 3282, 3065, 2926, 2858, 1638, 1543, 1254, 723, 695, 548 cm^{-1}. HRMS (ESI) m/z $(M+H)^+$ calcd for $C_{15}H_{23}N_2O_2$ 263.1754, found 263.1767.

***N*-butyl-2-(2-phenylacetamido)acetamide, (1b).**

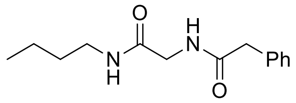

1b, 76%

The title compound was synthesized following the general procedure A. The crude material was purified by column chromatography using PE/EtOAc 3:7, affording compound **1b** (160.0 mg, 0.608 mmol, 76%) a white solid. Mp 148-150 °C; ^{1}H-NMR (400 MHz; $CDCl_3$): δ 7.34 (d, *J* = 7.4 Hz, 2H), 7.28 (t, *J* = 7.4 Hz, 3H), 6.86 (br s, 1H), 6.74 (br s, 1H), 6.39 (d, *J* = 5.1 Hz, 2H), 3.59 (s, 2H), 3.18 (q, *J* = 6.6 Hz, 2H), 1.42 (quint, *J* = 6.6 Hz, 2H), 1.30 (sext, *J* = 6.6 Hz, 2H), 0.90 (t, *J* = 6.6 Hz, 3H). ^{13}C-NMR (101 MHz; $CDCl_3$): δ 171.76, 168.75, 134.68, 129.27, 128.9, 127.3, 43.5, 43.3, 39.3, 31.4, 20.0, 13.7. IR (neat): ṽ = 3284, 2929, 2870, 1637, 1546, 1434, 1367, 963, 727, 695 cm^{-1}. HRMS (ESI) m/z $(M+H)^+$ calcd for $C_{14}H_{21}N_2O_2$ 249.1599, found 249.1607.

***N*-(2-oxo-2-(pentylamino)ethyl)hexanamide, (1c).**

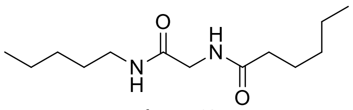

1c, 75%

The title compound was synthesized following the general procedure A. The crude material was purified by column chromatography using PE/EtOAc 5:5, affording compound **1c** (145.4 mg, 0.60 mol, 75%) a white solid. Mp 157-159 °C; ^{1}H-NMR (400 MHz; $CDCl_3$): δ 7.00 (br s, 1H), 6.92 (br s, 1H), 3.86 (d, J = 5.1 Hz, 2H), 3.16 (q, , J = 6.3 Hz, 2H), 2.17 (t, , J = 7.0 Hz, 2H), 1.56 (quint, J = 6.3 Hz, 2H), 1.43 (quint, , J = 7.0 Hz, 2H), 1.27-1.20 (m, 8H), 0.84-0.80 (m, 6H). ^{13}C-NMR (101 MHz; $CDCl_3$): δ 174.1, 169.1, 43.4, 39.6, 36.2, 31.4, 29.0 (2C), 25.3, 22.3 (2C), 13.9 (2C). IR (neat): ṽ = 3281, 2923, 2857, 1635, 1554, 1436, 1375, 1252, 700, 567 cm^{-1}. HRMS (ESI) m/z $(M+H)^+$ calcd for $C_{13}H_{27}N_2O_2$ 243.2067, found 243.2075.

_N_-(2-((2-morpholinoethyl)amino)-2-oxoethyl)benzamide, (1d).

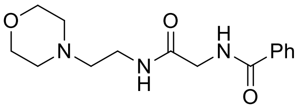

1d, 50%

The title compound was synthesized following the general procedure A. The crude material was purified by column chromatography using PE/EtOAc 4:6 as eluent, affording compound **1d** (116.5 mg, 0.40 mmol, 50%) an orange oil. ^{1}H-NMR (400 MHz; CD_3OD): δ 8.39 (t, *J* = 5.9 Hz, 1H), 7.87 (t, *J* = 5.6 Hz, 1H), 7.83 (d, *J* = 7.3 Hz, 2H), 7.50 (t, *J* = 7.3 Hz, 1H), 7.41 (t, *J* = 7.3 Hz, 2H), 4.05 (d, *J* = 5.6 Hz, 2H), 3.96-3.92 (m, 6H), 3.68-3.63 (m, 4H), 3.22 (t, *J* = 5.6 Hz, 2H). ^{13}C-NMR (101 MHz; CD_3OD): δ 171.9, 169.4, 133.3, 131.8, 128.3, 127.2, 63.6, 56.9, 52.2, 43.1, 33.6. IR (neat): ṽ = 3065, 2863, 1750, 1666, 1534, 1447, 1126, 832, 798, 516 cm^{-1}. HRMS (ESI) m/z $(M+H)^+$ calcd for $C_{15}H_{22}N_3O_3$ 292.1656, found 292.1667.

_N_-cyclohexyl-2-(2-(4-nitrophenyl)acetamido)acetamide, (1e).

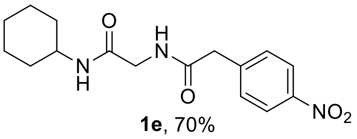

1e, 70%

The title compound was synthesized following the general procedure A. The crude material was purified by column chromatography using PE/EtOAc 5:5 as eluent, affording compound **1e** (178.8 mg, 0.56 mmol, 70%) a white solid. Mp 196-197 °C; ^{1}H-NMR (400 MHz; DMSO-d_6): δ 8.33 (br s, 1H), 8.18 (d, J = 8.6 Hz, 2H), 7.68 (d, J = 7.6 Hz, 1H), 7.56 (d, J = 8.6 Hz, 2H), 3.67 (s, 2H), 3.18 (d, J = 5.1 Hz, 2H), 1.72-1.64 (m, 4H), 1.56-1.53 (m, 1H), 1.29-1.09 (m, 6H). ^{13}C-NMR (101 MHz; DMSO-d_6): δ 169.7, 167.9, 146.8, 145.0, 130.9, 123.7, 48.0, 42.6, 42.2, 32.8, 25.7, 24.9. IR (neat): ṽ = 3285, 2919, 2853, 2459, 1624, 1508, 1448, 1340, 1249, 712, 512 cm^{-1}. HRMS (ESI) m/z $(M+H)^+$ calcd for $C_{16}H_{22}N_3O_4$ 320.1605, found 320.1612.

_N_-cyclohexyl-2-(2-phenylacetamido)pentanamide, (1f).

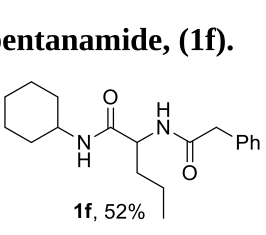

1f, 52%

The title compound was synthesized following the general procedure A. The crude material was purified by column chromatography using PE/EtOAc 5:5 as eluent, affording compound **1f** (131.6 mg, 0.416 mmol, 52%) a white solid. Mp 165-166 °C; ^{1}H-NMR (400 MHz; $CDCl_3$): δ 7.32 (t, *J* = 6.4 Hz, 2H), 7.28-7.25 (m, 3H), 6.63 (d, J = 8.2 Hz, 1H), 6.58 (d, *J* = 8.1 Hz, 1H), 4.43 (q, *J* = 7.2 Hz,

1H), 3.68 (sext, J = 4.0 Hz, 1H), 3.56 (s, 2H), 1.81 (q, J = 7.2 Hz, 2H), 1.70-1.67 (m, 2H), 1.54 (sext, J = 7.2 Hz, 2H), 1.31-1.28 (m, 4H), 1.16-1.04 (m, 4H), 0.87 (t, J = 7.2 Hz, 3H). ^{13}C-NMR (101 MHz; $CDCl_3$): δ 171.0, 170.7, 135.0, 129.2, 128.8, 127.1, 48.3, 34.7, 32.8, 32.6, 25.4, 24.7, 18.7, 13.8. IR (Kbr): ṽ = 3484, 3403, 3251, 3065, 2931, 2854, 1716, 1552, 1495, 1364, 1153, 694 cm^{-1}. HRMS (ESI) m/z $(M+H)^+$ calcd for $C_{19}H_{29}N_2O_2$ 317.2224, found 317.2228.

***N*-(2-oxo-2-((2,4,4-trimethylpentan-2-yl)amino)ethyl)-2-phenylacetamide, (1g).**

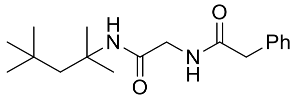

1g,71%

The title compound was synthesized following the general procedure A. The crude material was purified by column chromatography using PE/EtOAc 6:4 as eluent, affording compound **1g** (172.9 mg, 0.568 mmol, 71%) a white solid. Mp 143-145 °C; ^{1}H-NMR (400 MHz; $CDCl_3$): δ 7.36-7.27 (m, 5H), 6.71 (br s, 1H), 6.36 (br s, 1H), 3.81 (d, J = 5.1 Hz, 2H), 3.59 (s, 2H), 1.72 (s, 2H), 1.35 (s, 6H), 0.97 (s, 9H). ^{13}C-NMR (101 MHz; $CDCl_3$): δ 171.5, 167.5, 134.7, 129.3, 128.9, 127.3, 55.3, 51.2, 44.5, 43.4, 31.6, 31.4, 29.2. IR (Kbr): ṽ = 3346, 3315, 2088, 2953, 1686, 1638, 1387, 1345, 729, 694 cm^{-1}. HRMS (ESI) m/z $(M+H)^+$ calcd for $C_{18}H_{29}N_2O_2$ 305.2224, found 305.2232.

***N*-(*tert*-butyl)-2-(2-(3-cyanophenyl)acetamido)acetamide, (1h).**

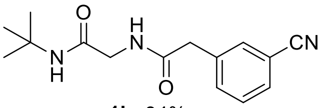

1h, 84%

The title compound was synthesized following the general procedure A. The crude material was purified by column chromatography using PE/EtOAc 4:6 as eluent, affording compound **1h** (183.7 mg, 0.672 mmol, 84%) a white solid. Mp 162-164 °C; ^{1}H-NMR (400 MHz; $CDCl_3$): δ 7.52-7.45 (m, 3H), 7.36 (t, J = 7.8 Hz, 1H), 7.01 (br s, 1H), 6.20 (br s, 1H), 3.76 (d, J = 4.9 Hz, 2H), 3.53 (s, 2H), 1.24 (s, 9H). ^{13}C-NMR (101 MHz; $CDCl_3$): δ 170.3, 167.8, 136.5, 133.8, 132.8, 130.8, 129.4, 118.6, 112.7, 51.5, 44.0, 42.4, 28.6. IR (neat): ṽ = 3297, 2968, 2870, 2230, 1637, 1543, 1456, 1364, 1217, 681 cm^{-1}. HRMS (ESI) m/z $(M+H)^+$ calcd for $C_{15}H_{20}N_3O_2$ 274.1550, found 274.1562.

N-(2-oxo-2-(phenylamino)ethyl)octanamide, (1i).

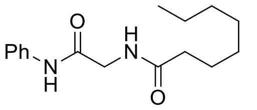

1i, 71%

The title compound was synthesized following the general procedure A. The crude material was purified by column chromatography using PE/EtOAc 6:4 as eluent, affording compound **1i** (160.0 mg, 0.568 mmol, 71%) a yellow solid. Mp 162-164 °C; ^{1}H-NMR (400 MHz; $CDCl_3$): δ 8.92 (br s, 1H), 7.57 (d, *J* = 7.7 Hz, 2H), 7.33 (t, *J* = 7.7 Hz, 2H), 7.13 (t, *J* = 7.7 Hz, 1H), 4.17 (d, *J* = 5.0 Hz, 2H), 2.32 (t, *J* = 7.4 Hz, 2H), 1.69 (quint, *J* = 7.4 Hz, 2H), 1.35-1.29 (m, 8H), 0.89 (t, *J* = 7.4 Hz, 3H). ^{13}C-NMR (101 MHz; $CDCl_3$): δ 174.4, 167.3, 137.8, 129.0, 124.4, 120.0, 44.6, 36.4, 31.6, 29.2, 29.0, 25.6, 22.6, 14.0. IR (neat): ṽ = 3044, 2922, 2850, 1735, 1670, 1640, 1536, 1442, 887, 742, 689 cm^{-1}. HRMS (ESI) m/z $(M+H)^+$ calcd for $C_{16}H_{25}N_2O_2$ 277.1917, found 277.1921.

N-(2-(benzylamino)-2-oxoethyl)hexanamide, (1j).

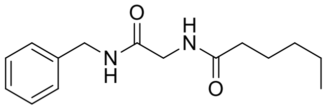

1j, 80%

The title compound was synthesized following the general procedure A. The crude material was purified by column chromatography using EtOAc/MeOH 9:1 as eluent, affording compound **1j** (167.9 mg, 0.64 mmol, 80%) a white solid. Mp 119-120 °C; ^{1}H-NMR (400 MHz; $CDCl_3$): δ 7.58 (br s, 1H), 7.30-7.22 (m, 5H), 7.04 (br s, 1H), 4.38 (d, *J* = 5.6 Hz, 2H), 3.95 (d, *J* = 5.0 Hz, 2H), 2.17 (t, *J* = 7.4 Hz, 2H), 1.56 (quint, *J* = 7.4 Hz, 2H), 1.30-1.27 (m, 4H), 0.88 (t, *J* = 7.4 Hz, 3H). ^{13}C-NMR (101 MHz; $CDCl_3$): δ 174.2, 169.3, 138.0, 128.6, 127.6, 127.4, 43.4, 43.3, 36.1, 31.4, 25.3, 22.3, 13.9.
IR (neat): ṽ = 3296, 2927, 2858, 1635, 1543, 1453, 1244, 729, 695 cm^{-1}. HRMS (ESI) m/z $(M+H)^+$ calcd for $C_{15}H_{23}N_2O_2$ 263.1754, found 263.1767.

N-benzyl-2-(2-phenylacetamido)acetamide, (1k).

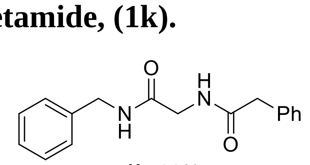

1k, 88%

The title compound was synthesized following the general procedure A. The crude material was purified by column chromatography using PE/EtOAc 4:6 as eluent, affording compound **1k** (198.8 mg, 0.704 mmol, 88%) an amorphous white solid. ^{1}H-NMR (400 MHz; DMSO-d_6): δ 7.31-7.28 (m,

6H), 7.26-7.21 (m, 4H), 4.30 (d, *J* = 5.9 Hz, 2H), 3.76 (d, *J* = 5.8 Hz, 2H), 3.51 (s, 2H). ^{13}C-NMR (101 MHz; DMSO): δ 171.1, 169.4, 139.8, 136.8, 129.6, 128.7, 128.6, 127.6, 127.2, 126.8, 42.7, 42.6, 42.5. IR (neat): ṽ = 3294, 3062, 2925, 2872, 2076, 1655, 1453, 1384, 722, 696 cm^{-1}. HRMS (ESI) m/z (M+H)$^+$ calcd for $C_{17}H_{19}N_2O_2$ 283.1441, found 283.1453.

***N*-benzyl-2-(2-phenylacetamido)propanamide, (1l).**

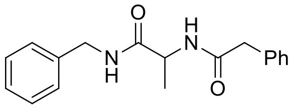

1l, 51%

The title compound was synthesized following the general procedure A. The crude material was purified by column chromatography using PE/EtOAc 4:6 as eluent, affording compound **1l** (120.9 mg, 0.408 mmol, 51%) a white solid. Mp 160-162 °C; ^{1}H-NMR (400 MHz; $CDCl_3$): δ 7.34-7.26 (m, 6H), 7.22-7.20 (m, 4H), 7.13 (br s, 1H), 6.55 (d, *J* = 7.2 Hz, 1H), 4.58 (quint, *J* = 7.2 Hz, 1H), 4.40-4.30 (m, 2H), 3.51 (s, 2H), 1.30 (d, *J* = 7.0 Hz, 3H). ^{13}C-NMR (101 MHz; $CDCl_3$): δ 172.4, 171.2, 138.1, 134.8, 129.2, 128.8, 128.6, 127.5, 127.3, 127.2. IR (neat): ṽ = 3272, 2978, 2930, 2008, 1632, 1536, 1423, 1241, 729, 694 cm^{-1}. HRMS (ESI) m/z (M+H)$^+$ calcd for $C_{18}H_{21}N_2O_2$ 297.1598, found 297.1609.

***N*-benzyl-2-(2-phenylacetamido)octanamide, (1m).**

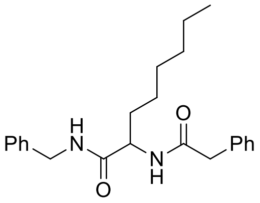

1m, 68%

The title compound was synthesized following the general procedure A. The crude material was purified by column chromatography using CH_2Cl_2/EtOAc 8:2 as eluent, affording compound **1m** (199.4 mg, 0.544 mmol, 68%) a white solid. Mp 125-127 °C; ^{1}H-NMR (400 MHz; $CDCl_3$): δ 7.34-7.27 (m, 6H), 7.23-7.21 (m, 4H), 6.94 (br s, 1H), 6.34 (d, *J* = 8.2 Hz, 1H), 4.50-4.44 (m, 1H), 4.43-4.39 (m, 1H), 4.34-4.29 (m, 1H), 3.53 (s, 2H), 1.82-1.73 (m, 1H), 1.58-1.50 (m, 1H), 1.28-1.21 (m, 8H), 0.88 (t, *J* = 6.7 Hz, 3H). ^{13}C-NMR (101 MHz; $CDCl_3$): δ 171.8, 171.2, 138.1, 134.9, 129.2, 128.8, 128.6, 127.6, 127.3, 127.2, 53.2, 43.4, 43.3, 32.5, 31.6, 28.9, 25.3, 22.5, 14.0. IR (neat): ṽ = 3270, 2923, 2856, 1629, 1539, 1356, 1239, 1029, 746, 692 cm^{-1}. HRMS (ESI) m/z (M+H)$^+$ calcd for $C_{23}H_{31}N_2O_2$ 367.2395, found 367.2380.

2-Acetamido-*N*-(3-(3-(piperidin-1-ylmethyl)phenoxy)propyl)acetamide, (1n).

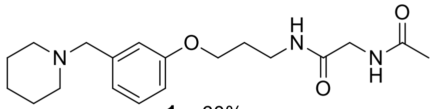

1n, 60%

The title compound was synthesized following the general procedure A. The crude material was purified by column chromatography using EtOAc/MeOH 8:2 as eluent, affording compound **1n** (166.8 mg, 0.480 mmol, 60%) an amorphous white solid. ^{1}H-NMR (400 MHz; CD_3OD): δ 7.23 (t, *J* = 7.9 Hz, 1H), 6.94 (s, 1H), 6.90 (d, *J* = 7.9 Hz, 1H), 6.85 (d, *J* = 7.9 Hz, 1H), 4.03 (t, *J* = 6.1 Hz, 2H), 3.83 (s, 2H), 3.48 (s, 2H), 3.41 (t, *J* = 6.1 Hz, 2H), 2.44-2.41 (m, 4H), 2.01 (s, 3H), 1.99-1.96 (m, 2H), 1.61 (quint, *J* = 5.8 Hz, 4H), 1.50-1.46 (m, 2H). ^{13}C-NMR (101 MHz; CD_3OD): δ 172.5, 170.4, 159.0, 138.3, 128.8, 121.9, 115.6, 113.2, 65.1, 63.3, 53.9, 42.3, 36.2, 28.8, 25.0, 23.7, 21.1. IR (KBr): ṽ = 3490, 3270, 2930, 2852, 2752, 2792, 1558, 1488, 1260, 1038, 796, 773 cm^{-1}. HRMS (ESI) m/z $(M+H)^+$ calcd for $C_{19}H_{30}N_3O_3$ 348.2282, found 348.2293.

***N*-(3-((1*H*-benzo[*d*]imidazol-1-yl)methyl)benzyl)-2-acetamidoacetamide, (1o).**

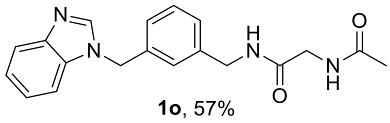

1o, 57%

The title compound was synthesized following the general procedure A. The crude material was purified by column chromatography using EtOAc/MeOH 9:1 as eluent, affording compound **1o** (153.4 mg, 0.456 mmol, 57%) a white solid. Mp 164-166 °C; ^{1}H-NMR (400 MHz; CD_3OD): δ 8.27 (s, 1H), 7.99 (br s, 1H), 7.72-7.68 (m, 2H), 7.50 (br s, 1H), 7.33 (s, 1H), 7.26-7.20 (m, 4H), 7.15 (d, J = 6.9 Hz, 1H), 5.46 (s, 2H), 4.37 (d, *J* = 5.9 Hz, 2H), 3.89 (d, *J* = 5.8 Hz, 2H), 1.94 (s, 3H).^{13}C-NMR (101 MHz; CDCl3): δ 170.3, 169.4, 140.2 (2C), 137.0, 128.8 (2C), 126.8, 126.5, 125.9, 122.5 (2C), 121.7, 119.8, 110.5, 48.1, 42.9, 42.2, 22.0. IR (neat): ṽ = 3476, 3415, 3228, 3054, 2927, 2811, 1617, 1548, 1286 cm^{-1}. HRMS (ESI) m/z $(M+H)^+$ calcd for $C_{19}H_{21}N_4O_2$ 337.1659, found 337.1665**.**

***N*-(2-((3-((1*H*-benzo[*d*]imidazol-1-yl)methyl)benzyl)amino)-2-oxoethyl)benzamide, (1p).**

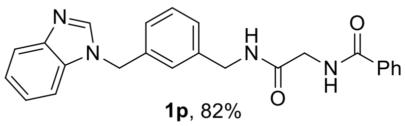

1p, 82%

The title compound was synthesized following the general procedure A. The crude material was purified by column chromatography using EtOAc/MeOH 9:1 as eluent, affording compound **1p** (261.4 mg, 0.656 mmol, 82%) a white solid. Mp 136-138 °C; ^{1}H-NMR (400 MHz; CD_3OD): 8.16 (s,

1H), 7.78 (d, *J* = 7.4 Hz, 2H), 7.56 (d, *J* = 8.0 Hz, 1H), 7.43 (t, *J* = 7.3 Hz, 1H), 7.37-7.33 (m, 3H), 7.18-7.12 (m, 5H), 7.03 (d, *J* = 7.0 Hz, 1H). ^{13}C-NMR (101 MHz; CD_3OD): δ 170.5, 169.2, 142.7, 139.5, 136.4, 134.1, 133.7, 131.5, 128.8, 128.2, 127.1, 126.9, 126.2, 125.9, 123.0, 122.3, 118.7, 110.6, 48.3, 42.9, 42.4. IR (KBr): ṽ = 3465, 3415, 3081, 2931, 1686, 1453, 1437, 1207, 1142, 737, 725 cm^{-1}. HRMS (ESI) m/z $(M+H)^+$ calcd for $C_{24}H_{23}N_4O_2$ 399.1816, found 399.1823.

General procedure B for the synthesis of compounds 2a-o.

To a flame-dried screw-capped tube containing a solution of the corresponding diamide (**1a-p**) (1 equiv, 0.40 mmol) in dry CH_2Cl_2 (1.4 mL) Burgess reagent (2 equiv, 0.80 mmol) is added under nitrogen atmosphere and the reaction is heated to 40 °C in an oil bath for one hour. Then, the reaction is diluted with CH_2Cl_2 and washed with water (x1). The organic layer was dried over sodium sulfate and evaporated. The crude product was purified by column chromatography.

The present reaction was also scaled up to 4.5 mmol as reported for compound **2a**.

Methyl *N*-(2-benzyloxazol-5-yl)-*N*-pentylsulfamoylcarbamate, (2a).

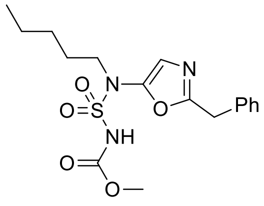

2a, 87%

The title compound was synthesized following the general procedure B. The crude material was purified by column chromatography using PE/EtOAc 4:6 as eluent, affording compound **2a** (1.5 g, 3.99 mmol, 87%) a yellow oil. ^{1}H-NMR (400 MHz; $CDCl_3$): δ 7.31-7.23 (m, 3H), 7.20 (d, *J* = 7.5 Hz, 2H), 6.87 (s, 1H), 3.99 (s, 2H), 3.73 (t, *J* = 7.4 Hz, 2H), 3.67 (s, 3H), 1.49-1.45 (m, 2H), 1.29-1.21 (m, 4H), 0.84 (t, *J* = 7.4 Hz, 3H). ^{13}C-NMR (101 MHz; $CDCl_3$): δ 162.0, 152.6, 144.5, 134.8, 128.8, 128.6, 127.2, 122.6, 53.3, 52.6, 34.6, 28.3, 28.2, 22.1, 13.9. IR (neat): ṽ = 3031, 2956, 2926, 2120, 1750, 1624, 1454, 1375, 1165, 865 cm^{-1}. HRMS (ESI) m/z $(M-H)^-$ calcd for $C_{17}H_{22}N_3O_5S$ 380.1286, found 380.1285.

Methyl *N*-(2-benzyloxazol-5-yl)-*N*-butylsulfamoylcarbamate, (2b).

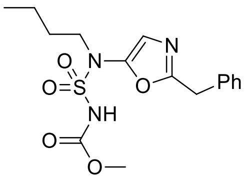

2b, 68%

The title compound was synthesized following the general procedure B. The crude material was purified by column chromatography using EtOAc/MeOH 9:1 as eluent, affording compound **2b** (99.9 mg, 0.272 mmol, 68%) a white solid. Mp 92-94 °C; ^{1}H-NMR (400 MHz; $CDCl_3$): δ 7.33 (d, *J* = 7.7 Hz, 2H), 7.27-7.25 (m, 3H), 6.92 (s, 1H), 4.06 (s, 2H), 3.79-3.77 (m, 2H), 3.75 (s, 3H), 1.50 (quint, *J* = 7.2 Hz, 2H), 1.34 (sext, *J* = 7.2 Hz, 2H), 0.89 (t, *J* = 7.2 Hz, 3H). ^{13}C-NMR (101 MHz; $CDCl_3$): 151.6, 134.6, 128.8, 128.6, 127.3, 122.7, 53.5, 52.6, 34.6, 30.7, 19.3, 13.5. IR (neat): ṽ = 3065, 2954, 2929, 2100, 1762, 1625, 1492, 1378, 1152, 857 cm^{-1}. HRMS (ESI) m/z $(M-H)^-$ calcd for $C_{16}H_{20}N_3O_5S$ 366.1129, found 366.1128.

Methyl *N*-pentyl-*N*-(2-pentyloxazol-5-yl)sulfamoylcarbamate, (2c).

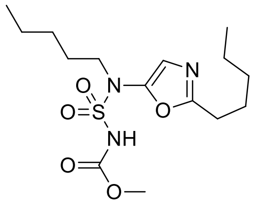

2c, 73%

The title compound was synthesized following the general procedure B. The crude material was purified by column chromatography using PE/EtOAc 5:5 as eluent, affording compound **2c** (105.5 mg, 0.292 mmol, 73%) a yellow oil. ^{1}H-NMR (400 MHz; $CDCl_3$): δ 6.81 (s, 1H), 3.84 (s, 3H), 3.78 (t, *J* = 7.3 Hz, 2H), 2.68 (t, *J* = 7.6 Hz, 2H), 1.67 (quint, *J* = 7.3 Hz, 2H), 1.54 (quint, *J* = 7.6 Hz, 2H), 1.35-1.31 (m, 8H), 0.92-0.87 (m, 6H). ^{13}C-NMR (101 MHz; $CDCl_3$): δ 164.4, 151.7, 143.3, 122.6, 53.4, 52.8, 31.1, 28.3, 28.2 (2C), 26.5, 22.2, 22.1, 13.8, 13.7. IR (neat): ṽ = 2957, 2930, 2860, 2105, 1752, 1624, 1458, 1375, 1166, 865 cm^{-1}. HRMS (ESI) m/z $(M-H)^-$ calcd for $C_{15}H_{26}N_3O_5S$ 360.1599, found 360.1599.

Methyl *N*-(2-morpholinoethyl)-*N*-(2-phenyloxazol-5-yl)sulfamoylcarbamate, (2d)

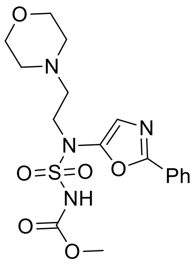

2d, 39%

The title compound was synthesized following the general procedure B. The crude material was purified by column chromatography using EtOAc as eluent, affording compound **2d** (64.0 mg, 0.156 mmol, 39%) a yellow oil. ^{1}H-NMR (400 MHz; CD_3OD): δ 7.99-7.96 (m, 2H), 7.52-7.50 (m, 3H), 7.08 (s, 1H), 4.10 (t, *J* = 5.2 Hz, 2H), 4.02-4.00 (m, 6H), 3.66 (s, 3H), 3.34 (t, *J* = 4.7 Hz, 4H). ^{13}C-NMR (101 MHz; CD_3OD): δ 161.9, 159.4, 146.9, 130.7, 128.8, 126.8, 125.8, 121.6, 64.0, 57.7, 52.8, 51.9, 45.0. IR (neat): ṽ = 3447, 2952, 2922, 2120, 1615, 1440, 1377, 1158, 870, 619 cm^{-1}. HRMS (ESI) m/z $(M-H)^-$ calcd for $C_{17}H_{21}N_4O_6S$ 409.1187, found 409.1184.

Methyl *N*-cyclohexyl-*N*-(2-(4-nitrobenzyl)oxazol-5-yl)sulfamoylcarbamate, (2e).

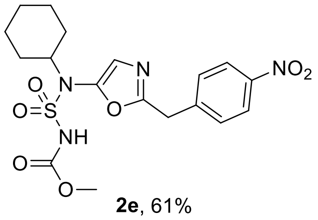

2e, 61%

The title compound was synthesized following the general procedure B. The crude material was purified by column chromatography using Cy/EtOAc 4:6 as eluent, affording compound **2e** (107.0 mg, 0.244 mmol, 61%) a brown solid. Mp 135-136 °C; ^{1}H-NMR (400 MHz; $CDCl_3$): δ 8.20 (d, *J* = 8.6 Hz, 2H), 7.47 (d, *J* = 8.6 Hz, 2H), 6.95 (s, 1H), 4.20 (s, 2H), 4.04 (quint, *J* = 4.4 Hz, 1H), 3.78 (s, 3H), 2.03-2.00 (m, 2H), 1.81-1.76 (m, 2H), 1.42-1.28 (m, 4H), 1.11-1.06 (m, 2H). ^{13}C-NMR (101 MHz; $CDCl_3$): δ 161.0, 147.3, 142.5, 142.2, 129.7 (2C), 126.0, 123.9, 61.1, 53.6, 34.7, 32.3, 25.6, 25.0. IR (neat): ṽ = 2932, 2858, 2170, 1745, 1522, 1480, 1343, 1159, 857, 588 cm^{-1}. HRMS (ESI) m/z $(M-H)^-$ calcd for $C_{18}H_{21}N_4O_7S$ 437.1136, found 437.1136.

Methyl *N*-(2-benzyl-4-propyloxazol-5-yl)-*N*-cyclohexylsulfamoylcarbamate, (2f).

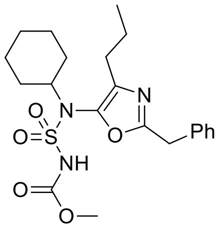

2f, 49%

The title compound was synthesized following the general procedure B. The crude material was purified by column chromatography using Cy/EtOAc 8:2 as eluent, affording compound **2f** (85.4 mg, 0.196 mmol, 49%) a pale yellow oil. ^{1}H-NMR (400 MHz; $CDCl_3$): δ 7.31 (d, *J* = 6.8 Hz, 2H), 7.27-7.24 (m, 3H), 4.17-4.15 (m, 1H), 4.04 (s, 2H), 3.73 (s, 3H), 2.39 (t, *J* = 7.8 Hz, 2H), 2.04-2.01 (m, 2H), 1.77-1.67 (m, 4H), 1.35-1.28 (m, 6H), 0.98 (t, *J* = 7.8 Hz, 3H). ^{13}C-NMR (101 MHz; $CDCl_3$): δ 161.6, 151.0, 139.7, 136.2, 135.2, 128.6 (2C), 127.0, 62.6, 53.4, 35.2, 32.4, 27.5, 25.7, 25.0, 21.5, 14.1. IR (neat): ṽ = 2924, 2853, 1748, 1454, 1373, 1258, 1165, 728, 593 cm^{-1}. HRMS (ESI) m/z $(M-H)^-$ calcd for $C_{21}H_{29}N_3O_5$ 434.1755, found 434.1753.

Methyl *N*-(2-benzyloxazol-5-yl)-*N*-(2,4,4-trimethylpentan-2-yl)sulfamoylcarbamate, (2g).

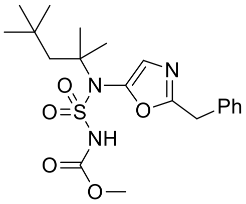

2g, 34%

The title compound was synthesized following the general procedure B. The crude material was purified by column chromatography using PE/EtOAc 3:7 as eluent, affording compound **2g** (57.6 mg, 0.136 mmol, 34%) a yellow oil. ^{1}H-NMR (400 MHz; $CDCl_3$): δ 7.32 (d, *J* = 6.8 Hz, 2H), 7.27-7.25 (m, 3H), 6.95 (s, 1H), 4.06 (s, 2H), 3.75 (s, 3H), 2.00 (s, 2H), 1.46 (s, 6H), 1.00 (s, 9H). ^{13}C-NMR (101 MHz; $CDCl_3$): δ 162.3, 144.3, 135.0, 129.8, 128.8, 128.6, 127.2, 126.1, 53.4, 53.0, 34.8, 31.6, 31.3, 28.9, 26.9. IR (neat): ṽ = 2952, 2923, 2098, 1754, 1651, 1552, 1440, 1365, 1163, 868 cm^{-1}. HRMS (ESI) m/z $(M-H)^-$ calcd for $C_{20}H_{28}N_3O_5S$ 422.1755, found 422.1755.

Methyl *N*-(tert-butyl)-*N*-(2-(3-cyanobenzyl)oxazol-5-yl)sulfamoylcarbamate, (2h).

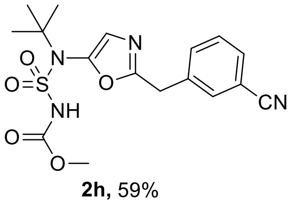

2h, 59%

The title compound was synthesized following the general procedure B. The crude material was purified by column chromatography using PE/EtOAc 6:4 as eluent, affording compound **2h** (92.6 mg,

0.236 mmol, 59%) a red oil. ^{1}H-NMR (400 MHz; $CDCl_3$): δ 7.58-7.57 (m, 2H), 7.53 (d, *J* = 8.0 Hz, 1H), 7.45 (t, *J* = 8.0 Hz, 1H), 6.98 (s, 1H), 4.12 (s, 2H), 3.79 (s, 3H), 1.43 (s, 9H).
^{13}C-NMR (101 MHz; $CDCl_3$): δ 160.8, 151.4, 145.1, 136.5, 133.3, 132.2, 131.0, 129.7, 125.7, 118.4, 112.8, 64.1, 53.4, 34.2, 29.4. IR (neat): ṽ = 2980, 2922, 2229, 2107, 1736, 1553, 1363, 1157, 940, 603 cm^{-1}. HRMS (ESI) m/z $(M-H)^-$ calcd for $C_{17}H_{19}N_4O_5S$ 391.1082, found 391.1084.

Methyl *N*-(2-heptyloxazol-5-yl)-*N*-phenylsulfamoylcarbamate, (2i).

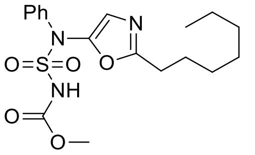

2i, 70%

The title compound was synthesized following the general procedure B. The crude material was purified by column chromatography using Cy/EtOAc 3:7 as eluent, affording compound **2i** (110.7 mg, 0.280 mmol, 70%) a yellow oil. ^{1}H-NMR (400 MHz; $CDCl_3$): δ 7.51 (d, *J* = 7.6 Hz, 2H), 7.37 (t, *J* = 7.6 Hz, 2H), 7.31 (t, *J* = 7.6 Hz, 1H), 7.03 (s, 1H), 3.74 (s, 3H), 2.66 (t, *J* = 7.7 Hz, 2H), 1.67 (quint, *J* = 7.7 Hz, 2H), 1.30-1.25 (m, 8H), 0.88 (t, *J* = 7.7 Hz, 3H).
^{13}C-NMR (101 MHz; $CDCl_3$): δ 164.1, 144.6, 139.4, 129.4 (2C), 128.2, 127.4, 122.6, 53.7, 31.6, 29.0, 28.8, 28.4, 26.7, 22.5, 14.0. IR (neat): ṽ = 2927, 2855, 1752, 1594, 1378, 1292, 1163, 865, 694, 565 cm^{-1}. HRMS (ESI) m/z $(M-H)^-$ calcd for $C_{18}H_{24}N_3O_5S$ 394.1442, found 394.1440.

Methyl *N*-benzyl-*N*-(2-pentyloxazol-5-yl)sulfamoylcarbamate, (2j).

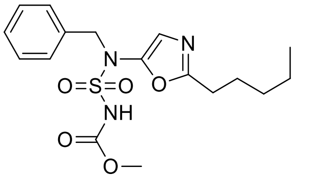

2j, 71%

The title compound was synthesized following the general procedure B. The crude material was purified by column chromatography using PE/EtOAc 5:5 as eluent, affording compound **2j** (108.3 mg, 0.284 mmol, 71%) a yellow oil. ^{1}H-NMR (400 MHz; $CDCl_3$): δ 7.29-7.22 (m, 5H), 6.64 (s, 1H), 4.91 (s, 2H), 3.75 (s, 3H), 2.59 (t, *J* = 7.6 Hz, 2H), 1.57 (quint, *J* = 7.6 Hz, 2H), 1.31-1.26 (m, 2H), 1.22-1.14 (m, 2H), 0.87 (t, *J* = 7.6 Hz, 3H).^{13}C-NMR (101 MHz; $CDCl_3$): δ 163.8, 154.0, 143.8, 135.6, 128.5, 128.4, 128.0, 122.5, 56.0, 53.3, 31.0, 28.2, 26.5, 22.2, 13.8. IR (neat): ṽ = 2955, 2929, 2122, 1765, 1620, 1438, 1379, 1153, 862, 621 cm^{-1}. HRMS (ESI) m/z $(M-H)^-$ calcd for $C_{17}H_{22}N_3O_5S$ 380.1286, found 380.1286.

Methyl *N*-benzyl-*N*-(2-benzyloxazol-5-yl)sulfamoylcarbamate, (2k).

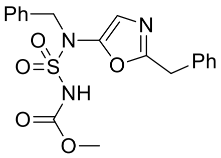

2k, 37%

The title compound was synthesized following the general procedure B. The crude material was purified by column chromatography using EtOAc/MeOH 9:1 as eluent, affording compound **2k** (59.4 mg, 0.148 mmol, 37%) a yellow oil. ^{1}H-NMR (400 MHz; $CDCl_3$): δ 7.31-7.28 (m, 4H), 7.23-7.21 (m, 4H), 7.16 (d, *J* = 5.5 Hz, 2H), 7.10 (d, *J* = 5.6 Hz, 2H), 6.69 (s, 1H), 4.88 (s, 2H), 3.94 (s, 2H), 3.69 (s, 3H). ^{13}C-NMR (101 MHz; $CDCl_3$): δ 135.1, 134.8, 128.8 (2C), 128.6 (2C), 128.5 (4C), 128.1, 127.2, 56.1, 53.5, 34.5. IR (neat): ṽ = 2955, 2924, 2122, 1765, 1627, 1455, 1367, 1149, 886, 725 cm^{-1}. HRMS (ESI) m/z (M-H)$^-$ calcd for $C_{19}H_{18}N_3O_5S$ 400.0973, found 400.0972.

Methyl *N*-benzyl-*N*-(2-benzyl-4-methyloxazol-5-yl)sulfamoylcarbamate, (2l).

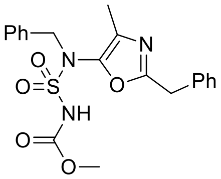

2l, 46%

The title compound was synthesized following the general procedure B. The crude material was purified by column chromatography using PE/EtOAc 2:8 as eluent, affording compound **2l** (76.4 mg, 0.184 mmol, 46%) a yellow oil. ^{1}H-NMR (400 MHz; $CDCl_3$): δ 7.34-7.30 (m, 3H), 7.27-7.22 (m, 2H), 7.18-7.14 (m, 5H), 4.84 (s, 2H), 3.94 (s, 2H), 3.72 (s, 3H), 1.73 (s, 3H). ^{13}C-NMR (101 MHz; $CDCl_3$): δ 161.1, 138.6, 135.2, 134.2, 129.0, 128.7 (2C), 128.6 (2C), 128.5, 128.2, 127.1, 56.5, 53.4, 34.8, 10.4. IR (neat): ṽ = 2956, 2923, 2121, 1757, 1662, 1489, 1361, 1150, 877, 692 cm^{-1}. HRMS (ESI) m/z (M-H)$^-$ calcd for $C_{20}H_{20}N_3O_5S$ 414.1129, found 414.1129.

Methyl *N*-benzyl-*N*-(2-benzyl-4-hexyloxazol-5-yl)sulfamoylcarbamate, (2m).

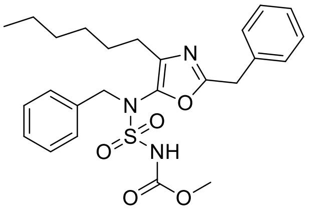

2m, 22%

The title compound was synthesized following the general procedure B. The crude material was purified by column chromatography using Cy/EtOAc 6:4 as eluent, affording compound **2m** (42.7

mg, 0.088 mmol, 22%) pale yellow oil. ^{1}H-NMR (400 MHz; $CDCl_3$): δ 7.35-7.30 (m, 4H), 7.27-7.24 (m, 2H), 7.20-7.18 (m, 2H), 7.15-7.14 (m 2H), 3.98 (s, 2H), 3.75 (s, 3H), 2.09 (t, *J* = 7.4 Hz, 2H), 1.29-1.23 (m, 4H), 1.17-1.11 (m, 4H), 0.88 (t, *J* = 7.4 Hz, 3H). ^{13}C-NMR (101 MHz; $CDCl_3$): δ 161.3, 151.3, 139.1, 137.2, 135.2, 134.8, 129.2, 128.7, 128.6, 128.5, 128.4, 127.1, 57.1, 53.6, 35.1, 31.5, 29.1, 27.7, 25.0, 22.5, 14.0. IR (neat): ṽ = 3031, 2928, 1749, 1455, 1373, 1237, 1159, 870, 696, 576 cm^{-1}. HRMS (ESI) m/z $(M-H)^-$ calcd for $C_{25}H_{30}N_3O_5S$ 484.1912, found 484.1911.

Methyl *N*-(2-methyloxazol-5-yl)-*N*-(3-(3-(piperidin-1ylmethyl)phenoxy)propyl)sulfamoyl carbamate (2n).

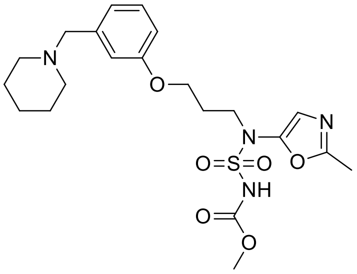

2n, 62%

The title compound was synthesized following the general procedure B. The crude material was purified by column chromatography using EtOAc/MeOH 9:1 as eluent, affording compound **2n** (111.7 mg, 0.248 mmol, 62%) a yellow oil. ^{1}H-NMR (400 MHz; $CDCl_3$): δ 7.23 (t, J = 7.9 Hz, 1H), 7.09 (s, 1H), 6.90-6.85 (m, 2H), 6.81 (s, 1H), 4.13 (t, *J* = 6.2 Hz, 2H), 3.91 (s, 2H), 3.84 (t, *J* = 6.2 Hz, 2H), 3.51 (s, 3H), 2.89-2.83 (m, 2H), 2.33 (s, 3H), 2.00 (quint, *J* = 6.2 Hz, 2H), 1.74 (quint, *J* = 4.8 Hz, 2H), 1.52-1.46 (m, 2H), 1.29-1.27 (m, 4H). ^{13}C-NMR (101 MHz; $CDCl_3$): δ 159.8, 159.3, 159.2, 147.2, 132.2, 129.6, 123.1, 121.6, 116.8, 116.2, 65.2, 53.1, 52.3, 48.0, 31.2, 28.5, 23.2, 22.4, 14.4. IR (neat): ṽ = 2926, 2852, 2120, 1680,1566, 1437, 1389, 1183, 866, 607 cm^{-1}. HRMS (ESI) m/z $(M-H)^-$ calcd for $C_{21}H_{29}N_4O_6S$ 465.1813, found 465.1811.

Methyl *N*-(3-((1*H*-benzo[*d*]imidazol-1-yl)methyl)benzyl)-*N*-(2-methyloxazol-5-ylsulfamoyl carbamate, (2o).

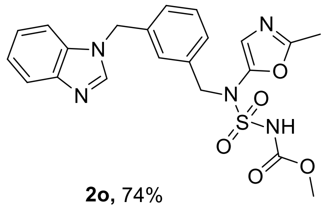

2o, 74%

The title compound was synthesized following the general procedure B. The crude material was purified by column chromatography using EtOAc/MeOH 9:1 as eluent, affording compound **2o** (134.8 mg, 0.296 mmol, 74%) a yellow oil. ^{1}H-NMR (400 MHz; CD_3OD): 8.32 (s, 1H), 7.72-7.70 (m, 1H), 7.41-7.39 (m, 1H), 7.32-7.30 (m, 2H), 7.28-7.25 (m, 3H), 7.20 (d, J = 7.1 Hz, 1H), 6.53 (s, 1H), 5.49 (s, 2H), 3.72 (s, 2H), 3.70 (s, 3H), 2.25 (s, 3H). ^{13}C-NMR (101 MHz; CD_3OD): δ 160.3, 154.8, 144.8, 143.4, 137.0, 136.3, 128.9, 128.0, 127.2, 126.8, 123.4, 122.8, 122.1, 120.9, 118.4, 111.9, 110.8, 54.9, 52.2, 51.5, 12.5. IR (neat): ṽ = 3055, 2951, 2708, 1725, 1682, 1457, 1369, 1160, 871, 672 cm^{-1}. HRMS (ESI) m/z $(M-H)^-$ calcd for $C_{21}H_{20}N_5O_5S$ 454.1191, found 454.1190.

General procedure C for the synthesis of compounds 14a-m.

To a flame-dried screw-capped tube containing a solution of the corresponding diamide (**1a-p**) (1 equiv, 0.40 mmol) in dry CH_2Cl_2 (1.4 mL) modified Burgess reagent (2 equiv, 0.80 mmol) is added under nitrogen atmosphere and the reaction is heated to 40 °C in an oil bath for one hour. Then, the reaction is diluted with CH_2Cl_2 and washed with water (x1). The organic layer was dried over sodium sulfate and evaporated. The crude product was purified by column chromatography. Compounds **14 a**, **14b**, **14c, 14f**, **14k**, **14l** and **14m** were then washed with an HCl 3N aqueous solution in order to remove any residual traces of Burgess reagent.

The present reaction was also scaled up to 3 mmol as reported for compound **14a.**

Benzyl *N*-(2-benzyloxazol-5-yl)-*N*-pentylsulfamoylcarbamate, (14a).

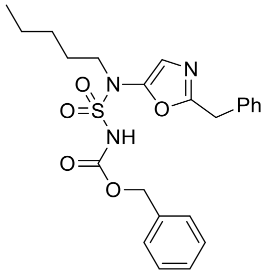

14a, 68%

The title compound was synthesized following the general procedure C. The crude material was purified by column chromatography using Cy/EtOAc 3:7 as eluent, affording compound **14a** (928.8 mg, 2.03 mmol, 68%) a yellow solid. Mp 109-111 °C; ^{1}H-NMR (400 MHz; $CDCl_3$): δ 7.37-7.35 (m,

5H), 7.31-7.26 (m, 3H), 7.17 (d, *J* = 6.4 Hz, 2H), 6.79 (s, 1H), 5.18 (s, 2H), 3.90 (s, 2H), 3.67 (t, J = 7.3 Hz, 2H), 1.44 (quint, *J* = 7.3 Hz, 2H), 1.30-1.29 (m, 2H), 1.26-1.21 (m, 2H), 0.85 (t, *J* = 7.3 Hz, 3H). ^{13}C-NMR (101 MHz; $CDCl_3$): δ 162.2, 150.8, 143.7, 134.7, 134.6, 128.9, 128.8, 128.6, 128.4, 128.2, 127.2, 123.2, 68.6, 53.0, 34.6, 28.2, 28.1, 22.1, 13.8. IR (neat): ṽ = 3032, 2925, 1762, 1553, 1455, 1365, 1153, 853, 726, 574 cm^{-1}. HRMS (ESI) m/z $(M-H)^-$ calcd for $C_{23}H_{26}N_3O_5S$ 456.1599, found 456.1596.

Benzyl *N*-(2-benzyloxazol-5-yl)-*N*-butylsulfamoylcarbamate, (14b).

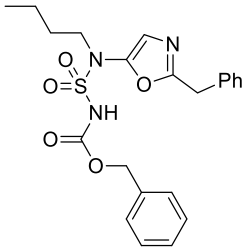

14b, 47%

The title compound was synthesized following the general procedure C. The crude material was purified by column chromatography using Cy/EtOAc 6:4 as eluent, affording **14b** (83.4 mg, 0.188 mmol, 47%) a white solid. Mp 108-110 °C; ^{1}H-NMR (400 MHz; $CDCl_3$): δ 7.37-7.35 (m, 5H), 7.30-7.26 (m, 3H), 7.17 (d, *J* = 6.6 Hz, 2H), 6.78 (s, 1H), 5.18 (s, 2H), 3.89 (s, 2H), 3.68 (t, *J* = 7.2 Hz, 2H), 1.42 (quint, *J* = 7.2 Hz, 2H), 1.32-1.28 (m, 2H), 0.85 (t, *J* = 7.2 Hz, 3H). ^{13}C-NMR (101 MHz; CDCl3): δ 162.2, 151.0, 143.9, 134.7 (2C), 128.9, 128.8 (2C), 128.6 (2C), 127.2, 123.1, 68.5, 52.7, 34.5, 30.7, 19.3, 13.5. IR (neat): ṽ = 3030, 2923, 1754, 1487, 1363, 1147, 851, 757, 729, 585 cm^{-1}. HRMS (ESI) m/z $(M-H)^-$ calcd for $C_{22}H_{24}N_3O_5S$ 442.1442, found 442.1441.

Benzyl *N*-pentyl-*N*-(2-pentyloxazol-5-yl)sulfamoylcarbamate, (14c).

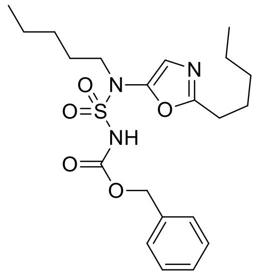

14c, 73%

The title compound was synthesized following the general procedure C. The crude material was purified by column chromatography using Cy/EtOAc 5:5 as eluent, affording compound **14c** (127.8 mg, 0.292 mmol, 73%) a white solid. Mp 67-69 °C; ^{1}H-NMR (400 MHz; $CDCl_3$): δ 7.40-7.36 (m, 5H), 6.70 (s, 1H), 5.24 (s, 2H), 3.71 (t, *J* = 7.4 Hz, 2H), 2.54 (t, *J* = 7.6 Hz, 2H), 1.57 (quint, *J* = 7.4

Hz, 2H), 1.46 (quint, *J* = 7.6 Hz, 2H), 1.30-1.21 (m, 8H), 0.90-0.87 (m, 6H). ^{13}C-NMR (101 MHz; $CDCl_3$): δ 164.4, 151.1 (2C), 143.2, 134.8, 128.7, 128.6, 122.8, 68.5, 53.0, 31.1, 28.3, 28.2, 28.1, 26.4, 22.2, 22.1, 13.8 (2C). IR (neat): ṽ = 3034, 2925, 1755, 1557, 1467, 1365, 1150, 852, 755, 585 cm^{-1}. HRMS (ESI) m/z $(M-H)^-$ calcd for $C_{21}H_{30}N_3O_5S$ 436.1912, found 436.1910.

Benzyl *N*-(2-morpholinoethyl)-*N*-(2-phenyloxazol-5-yl)sulfamoylcarbamate, (14d).

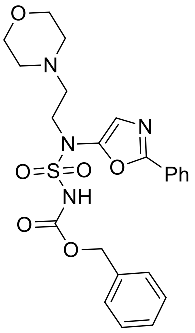

14d, 26%

The title compound was synthesized following the general procedure C. The crude material was purified by column chromatography using EtOAc/MeOH 9.8:0.2 as eluent, affording compound **14d** (50.6 mg, 0.104 mmol, 26%) a yellow oil. ^{1}H-NMR (400 MHz; CD_3OD): δ 7.95 (d, *J* = 8.0 Hz, 2H), 7.51-7.48 (m, 3H), 7.35-7.25 (m, 5H), 7.00 (s, 1H), 5.12 (s, 2H), 4.11 (t, *J* = 5.3 Hz, 2H), 3.95-3.92 (m, 4H), 3.38-3.33 (m, 4H), 1.33-1.27 (m, 2H).
^{13}C-NMR (101 MHz; CD_3OD): δ 159.6, 146.3, 137.0, 130.7, 128.7, 128.1, 128.0, 127.6, 127.3, 126.8, 125.9, 121.9, 67.1, 63.7, 57.4, 52.7, 45.0. IR (neat): ṽ = 2924, 2853, 1718, 1615, 1334, 1158, 1091, 868, 694, 578 cm^{-1}. HRMS (ESI) m/z $(M-H)^-$ calcd for $C_{23}H_{25}N_4O_6S$ 485.1500, found 485.1499.

Benzyl *N*-cyclohexyl-*N*-(2-(4-nitrobenzyl)oxazol-5-yl)sulfamoylcarbamate, (14e).

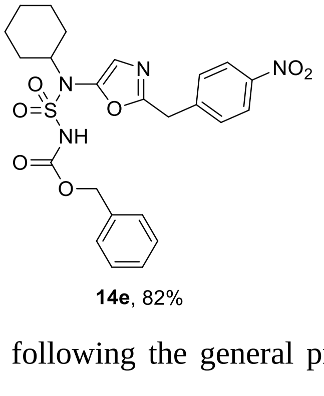

14e, 82%

The title compound was synthesized following the general procedure B. The crude material was purified by column chromatography using Cy/EtOAc 3.5/6.5 as eluent, affording compound **14e** (168.8 mg, 0.328 mmol, 82%) an amorphous yellow solid. ^{1}H-NMR (400 MHz; $CDCl_3$): δ 9.46 (br s, 1H), 8.12 (d, *J* = 8.4 Hz, 2H), 7.38-7.36 (m, 5H), 7.34 (d, 8.4 Hz, 2H), 6.82 (s, 1H), 5.22 (s, 2H), 4.05 (s, 2H), 1.91-1.88 (m, 2H), 1.72-1.69 (m, 2H), 1.58-1.55 (m, 1H), 1.32-1.20 (m, 4H), 1.00-0.92

(m, 2H). ^{13}C-NMR (101 MHz; $CDCl_3$): δ 161.3, 150.6, 147.2, 142.1, 142.0, 134.7, 129.6, 128.9, 128.8, 128.5, 125.9, 123.9, 68.7, 61.4, 34.4, 32.2, 25.5, 24.9. IR (neat): ṽ = 2929, 2856, 1750, 1519, 1452, 1345, 1164, 856, 733, 570 cm^{-1}. HRMS (ESI) m/z (M-H)$^-$ calcd for $C_{24}H_{22}N_4O_7S$ 513.1449, found 513.1448.

Benzyl *N*-(2-benzyl-4-propyloxazol-5-yl)-*N*-cyclohexylsulfamoylcarbamate, (14f).

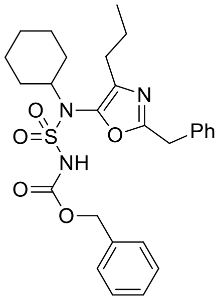

14f, 30%

The title compound was synthesized following the general procedure C. The crude material was purified by column chromatography using Cy/EtOAc 7:3 as eluent, affording compound **14f** (61.4 mg, 0.120 mmol, 30%) a pale yellow oil. ^{1}H-NMR (400 MHz; $CDCl_3$): δ 7.37-7.35 (m, 5H), 7.30-7.25 (m, 3H), 7.22 (t, *J* = 5.5 Hz, 2H), 5.17 (s, 2H), 4.13 (quint, *J* = 8.1 Hz, 1H), 3.95 (s, 2H), 2.36 (t, *J* = 7.7 Hz, 2H), 1.97-1.93 (m, 2H), 1.68 (quint, *J* = 8.1 Hz, 4H), 1.57-1.54 (m, 2H), 1.30-1.22 (m, 4H), 0.94 (t, 7.7 Hz, 3H). ^{13}C-NMR (101 MHz; $CDCl_3$): δ 161.8, 150.6, 139.5, 136.3, 135.1, 134.8, 128.7 (2C), 128.6 (2C), 128.4, 127.0, 68.4, 62.6, 35.0, 32.4, 27.3, 25.6, 25.0, 21.4, 14.1. IR (neat): ṽ = 2930, 2856, 1746, 1453, 1365, 1664, 1148, 858, 695, 578 cm^{-1}. HRMS (ESI) m/z (M-H)$^-$ calcd for $C_{27}H_{32}N_3O_5S$ 510.2068, found 510.2066.

Benzyl *N*-(2-benzyloxazol-5-yl)-*N*-(2,4,4-trimethylpentan-2-yl)sulfamoylcarbamate (14g).

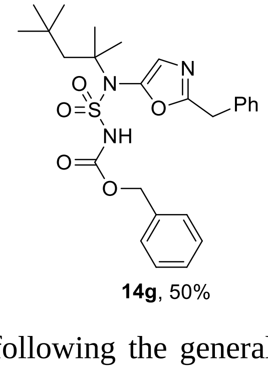

14g, 50%

The title compound was synthesized following the general procedure C. The crude material was purified by column chromatography using Cy/EtOAc 5:5 as eluent, affording compound **14g** (99.9 mg, 0.200 mmol, 50%) a yellow oil. ^{1}H-NMR (400 MHz; $CDCl_3$): δ 7.37-7.35 (m, 5H), 7.31-7.25 (m, 3H), 7.19 (d, *J* = 8.0 Hz, 2H), 6.87 (s, 1H), 5.19 (s, 2H), 3.92 (s, 2H), 1.45 (s, 2H), 1.40 (s, 6H), 0.97 (s, 9H). ^{13}C-NMR (101 MHz; $CDCl_3$): δ 162.4, 150.1, 144.2, 134.9, 134.8, 128.8, 128.7 (2C),

128.6 (2C), 127.1, 126.1, 70.2, 68.3, 53.0, 34.6, 31.6, 31.3, 28.9. IR (neat): ṽ = 2921, 2854, 1743, 1455, 1363, 1241, 1157, 888, 719, 614 cm^{-1}. HRMS (ESI) m/z $(M-H)^-$ calcd for $C_{26}H_{32}N_3O_5S$ 498.2068, found 498.2067.

Benzyl *N*-(*tert*-butyl)-*N*-(2-(3-cyanobenzyl)oxazol-5-yl)sulfamoylcarbamate, (14h).

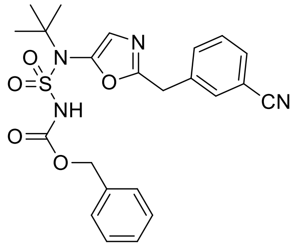

14h, 43%

The title compound was synthesized following the general procedure C. The crude material was purified by column chromatography using Cy/EtOAc 8:2 as eluent, affording compound **14h** (80.6 mg, 0.172 mmol, 43%) a yellow oil. ^{1}H-NMR (400 MHz; $CDCl_3$): δ 9.77 (br s, 1H), 7.55 (d, *J* = 7.3 Hz, 1H), 7.48-7.46 (m, 2H), 7.44 (d, *J* = 7.3 Hz, 1H), 7.37-7.35 (m, 5H), 6.89 (s, 1H), 5.22 (s, 2H), 3.98 (s, 2H), 1.37 (s, 9H). ^{13}C-NMR (101 MHz; $CDCl_3$): δ 160.8, 150.2, 144.8, 136.4, 134.8, 133.2, 132.1, 131.0, 129.6, 128.9, 128.8, 128.6, 125.8, 118.4, 112.9, 68.5, 64.4, 34.0, 29.4. IR (neat): ṽ = 2981, 2231, 1752, 1553, 1361, 1176, 962, 856, 735, 612 cm^{-1}. HRMS (ESI) m/z $(M-H)^-$ calcd for $C_{23}H_{23}N_4O_5S$ 467.1395, found 467.1393.

Benzyl *N*-(2-heptyloxazol-5-yl)-*N*-phenylsulfamoylcarbamate, (14i).

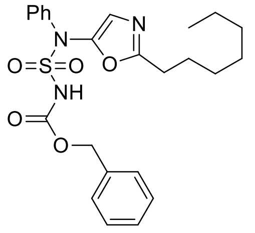

14i, 40%

The title compound was synthesized following the general procedure C. The crude material was purified by column chromatography using Cy/EtOAc 2:8 as eluent, affording compound **14i** (75.5 mg, 0.160 mmol, 40%) a yellow oil. ^{1}H-NMR (400 MHz; $CDCl_3$): δ 7.44 (d, *J* = 5.2 Hz, 2H), 7.40-7.38 (m, 5H), 7.34-7.32 (m, 3H), 7.01 (s, 1H), 5.27 (s, 2H), 2.58 (t, *J* = 7.7 Hz, 2H), 1.61 (quint, *J* = 7.7 Hz, 2H), 1.32-1.30 (m, 2H), 1.28-1.24 (m, 6H), 0.90 (t, *J* = 7.7 Hz, 3H). ^{13}C-NMR (101 MHz; $CDCl_3$): δ 164.5, 150.4, 138.7, 134.7, 129.5, 128.8, 128.7 (2C), 128.6, 128.3, 127.8, 122.9, 68.6, 31.6, 28.9, 28.8, 28.2, 26.7, 22.6, 14.0. IR (neat): ṽ = 2926, 2854, 1751, 1454, 1368, 1157, 862, 751, 693, 564 cm^{-1}. HRMS (ESI) m/z $(M-H)^-$ calcd for $C_{24}H_{28}N_3O_5S$ 470.1755, found 470.1754.

Benzyl *N*-benzyl-*N*-(2-pentyloxazol-5-yl)sulfamoylcarbamate, (14j).

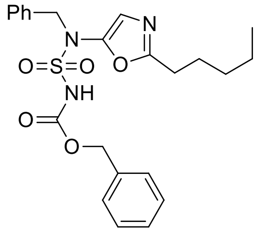

14j, 75%

The title compound was synthesized following the general procedure C. The crude material was purified by column chromatography using Cy/EtOAc 6:4 as eluent, affording compound **14j** (137.3 mg, 0.300 mmol, 75%) a yellow oil. ^{1}H-NMR (400 MHz; $CDCl_3$): δ 7.42-7.40 (m, 5H), 7.29-7.28 (m, 3H), 7.22-7.20 (m, 2H), 6.55 (s, 1H), 5.28 (s, 2H), 4.89 (s, 2H), 2.47 (t, *J* = 7.5 Hz, 2H), 1.49 (quint, *J* = 7.5 Hz, 2H), 1.31-1.22 (m, 2H), 1.16-1.10 (m, 2H), 0.89 (t, *J* = 7.5 Hz, 3H). ^{13}C-NMR (101 MHz; $CDCl_3$): δ 164.4, 151.2, 142.8, 135.1, 134.7, 128.9, 128.8, 128.7, 128.6, 128.5, 128.2, 123.2, 68.6, 56.6, 30.9, 28.0, 26.4, 22.2, 13.8. IR (neat): ṽ = 3032, 2926, 1763, 1624, 1369, 1156, 1087, 870, 733, 579 cm^{-1}. HRMS (ESI) m/z $(M-H)^-$ calcd for $C_{23}H_{26}N_3O_5S$ 456.1599, found 456.1599.

Benzyl *N*-benzyl-*N*-(2-benzyloxazol-5-yl)sulfamoylcarbamate, (14k).

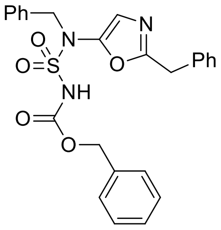

14k, 74%

The title compound was synthesized following the general procedure C. The crude material was purified by column chromatography using Cy/EtOAc 2:8 as eluent, affording compound **14k** (141.3 mg, 0.296 mmol, 74%) a white solid. Mp 124-126 °C; ^{1}H-NMR (400 MHz; $CDCl_3$): δ 7.38-7.36 (m, 5H), 7.31-7.28 (m, 2H), 7.25-7.22 (m, 4H), 7.11 (d, *J* = 7.8 Hz, 2H), 7.06 (d, *J* = 8.1 Hz, 2H), 6.62 (s, 1H), 5.21 (s, 2H), 4.82 (s, 2H), 3.85 (s, 2H). ^{13}C-NMR (101 MHz; $CDCl_3$): δ 162.1, 151.0, 143.4, 134.8, 134.7, 134.6, 128.9, 128.8, 128.7 (2C), 128.6, 128.5 (2C), 128.2, 127.2, 123.4, 68.6, 56.5, 34.5. IR (neat): ṽ = 3279, 2920, 1764, 1555, 1369, 1156, 1017, 866, 723, 537 cm^{-1}. HRMS (ESI) m/z $(M-H)^-$ calcd for $C_{25}H_{22}N_3O_5S$ 476.1286, found 476.1283.

Benzyl *N*-benzyl-*N*-(2-benzyl-4-methyloxazol-5-yl)sulfamoylcarbamate, (14l).

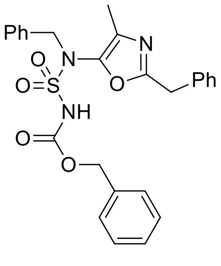

14l, 73%

The title compound was synthesized following the general procedure C. The crude material was purified by column chromatography using Cy/EtOAc 5:5 as eluent, affording compound **14l** (143.5 mg, 0.292 mmol, 73%) a white solid. Mp 109-111 °C; ^{1}H-NMR (400 MHz; $CDCl_3$): δ 7.38-7.36 (m, 5H), 7.30-7.26 (m, 3H), 7.24-7.20 (m, 2H), 7.13-7.09 (m, 5H), 5.20 (s, 2H), 4.77 (s, 2H), 3.85 (s, 2H) 1.67 (s, 3H). ^{13}C-NMR (101 MHz; $CDCl_3$): δ 161.4, 150.9, 137.9, 135.0, 134.9, 134.7, 134.6, 129.0, 128.9, 128.7 (2C), 128.6 (2C), 128.5, 128.3, 127.1, 68.6, 56.9, 34.7, 10.4.
IR (neat): ṽ = 3032, 2924, 1768, 1526, 1454, 1359, 1146, 890, 690, 573 cm^{-1}. HRMS (ESI) m/z $(M-H)^-$ calcd for $C_{26}H_{24}N_3O_5S$ 490.1442, found 490.1441.

Benzyl *N*-benzyl-*N*-(2-benzyl-4-hexyloxazol-5-yl)sulfamoylcarbamate, (14m).

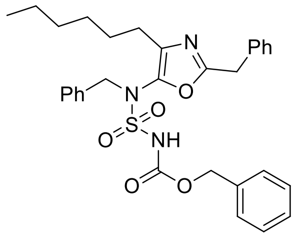

14m, 30%

The title compound was synthesized following the general procedure C. The crude material was purified by column chromatography using Cy/EtOAc 7:3 as eluent, affording compound **14m** (67.4 mg, 0.120 mmol, 30%) a pale yellow oil. ^{1}H-NMR (400 MHz; $CDCl_3$): δ 7.39-7.38 (m, 5H), 7.33-7.29 (m, 3H), 7.24-7.20 (m, 3H), 7.16 (d, J = 7.8 Hz, 2H), 7.08 (d, J = 6.5 Hz, 2H), 5.21 (s, 2H), 4.77 (s, 2H), 3.94 (s, 2H), 2.08 (t, J = 7.5 Hz, 2H), 1.32-1.29 (m, 2H), 1.25-1.22 (m, 2H), 1.15 (sext, J = 7.5 Hz, 2H), 1.11-1.07 (m, 2H), 0.87 (t, J = 7.5 Hz, 3H). ^{13}C-NMR (101 MHz; $CDCl_3$): δ 161.5, 150.7, 138.7, 137.4, 135.5, 135.0, 134.7, 134.6, 129.1, 128.9, 128.8, 128.7, 128.6, 128.5, 128.4, 127.1, 68.7, 57.1, 34.9, 31.5, 29.1, 27.6, 24.8, 22.5, 14.0. IR (neat): ṽ = 3031, 2926, 1747, 1454, 1366, 1222, 1157, 868, 696, 576 cm^{-1}. HRMS (ESI) m/z $(M-H)^-$ calcd for $C_{31}H_{34}N_3O_5S$ 560.2225, found 560.2221.

General procedure D for the synthesis of compounds 15a-l.

To a solution of Pd/C 5% (18 mg) in methanol (0.6 mL) the corresponding 5-sulfamido oxazoles **14a-l** (1 equiv, 0.14 mmol) were added; the mixture was flushed with hydrogen and stirred for one

hour. The reaction was filtered and concentrated. The crude product was purified by column chromatography.

The present reaction was also scaled up to 1 mmol as reported for compound **15a**.

***N*-(2-benzyloxazol-5-yl)-*N*-pentylsulfamide, (15a).**

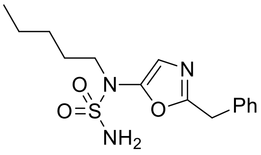

15a, 56%

The title compound was synthesized following the general procedure D. The crude material was purified by column chromatography using Cy/EtOAc 7:3 as eluent, affording compound **15a** (181.1 mg, 0.56 mmol, 56%) a white solid. Mp 124-126 °C; ^{1}H-NMR (400 MHz; $CDCl_3$): δ 7.40-7.32 (m, 2H), 7.30-7.26 (m, 3H), 6.86 (s, 1H), 5.05 (br s, 2H), 4.07 (s, 2H), 3.49 (t, *J* = 7.3 Hz, 2H), 1.51 (quint, *J* = 7.3 Hz, 2H), 1.32-1.27 (m, 4H), 0.88 (t, *J* = 7.3 Hz, 3H). ^{13}C-NMR (101 MHz; $CDCl_3$): δ 161.7, 145.4, 134.9, 128.8 (2C), 127.2, 122.6, 51.2, 35.0, 28.4, 27.8, 22.1, 13.9. IR (neat): ṽ = 3315, 3124, 2959, 2930, 1615, 1551, 1373, 1335, 1163, 752 cm^{-1}. HRMS (ESI) m/z $(M-H)^-$ calcd for $C_{15}H_{20}N_3O_3S$ 322.1228, found 322.1231.

***N*-(2-benzyloxazol-5-yl)-*N*-butylsulfamide, (15b).**

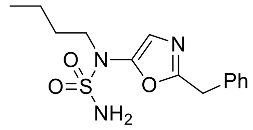

15b, 76%

The title compound was synthesized following the general procedure D. The crude material was purified by column chromatography using Cy/EtOAc 7:3 as eluent, affording compound **15b** (23.5 mg, 0.076 mmol, 76%) a white solid. Mp 130-132 °C; ^{1}H-NMR (400 MHz; $CDCl_3$): δ 7.37-7.33 (m, 2H), 7.30-7.26 (m, 3H), 6.85 (s, 1H), 5.06 (br s, 2H), 4.07 (s, 2H), 3.50 (t, *J* = 7.3 Hz, 2H), 1.49 (quint, *J* = 7.3 Hz, 2H), 1.34 (sext, *J* = 7.3 Hz, 2H), 0.89 (t, *J* = 7.3 Hz, 3H). ^{13}C-NMR (101 MHz; $CDCl_3$): δ 161.7, 145.4, 134.9, 128.8, 127.2, 122.6 (2C), 50.9, 35.0, 30.2, 19.5, 13.5. IR (neat): ṽ = 3126, 2931, 2869, 1551, 1373, 1164, 1116, 904, 725, 543 cm^{-1}. HRMS (ESI) m/z $(M-H)^-$ calcd for $C_{14}H_{18}N_3O_3S$ 308.1074, found 308.1073.

***N*-(2-benzyl-4-propyloxazol-5-yl)-*N*-cyclohexylsulfamide, (15f).**

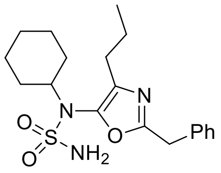

15f, 72%

The title compound was synthesized following the general procedure D. The crude material was purified by column chromatography using Cy/EtOAc 8:2 as eluent, affording compound **15f** (27.2 mg, 0.072 mmol, 72%) a white solid. Mp 126-128 °C; ^{1}H-NMR (400 MHz; $(CD_3)_2CO$): δ 7.33-7.31 (m, 4H), 7.27-7.26 (m, 1H), 6.41 (br s, 2H), 4.05 (s, 2H), 3.91-3.86 (m, 1H), 2.40 (t, *J* = 7.7 Hz, 2H), 2.10-2.04 (m, 2H), 1.75-1.65 (m, 4H), 1.58-1.55 (m, 2H), 1.38-1.29 (m, 2H), 1.15-1.06 (m, 2H), 0.96 (t, *J* = 7.7 Hz, 3H). ^{13}C-NMR (101 MHz; $(CD_3)_2CO$): δ 160.3, 138.8, 137.9, 136.3, 128.6, 128.4, 126.7, 59.7, 34.6, 32.2, 27.4, 25.6, 25.0, 21.2, 13.5. IR (neat): ṽ = 3305, 3031, 2929, 2869, 1555, 1359, 1168, 1116, 697, 597 cm^{-1}. HRMS (ESI) m/z $(M-H)^-$ calcd for $C_{19}H_{26}N_3O_3S$ 376.1700, found 376.1697.

***N*-(*tert*-butyl)-*N*-(2-(3-cyanobenzyl)oxazol-5-yl)sulfamide, (15h).**

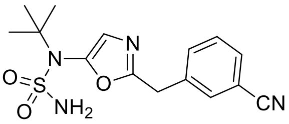

15h, 40%

The title compound was synthesized following the general procedure D. The crude material was purified by column chromatography using Cy/EtOAc 6:4 as eluent, affording compound **15h** (13.4 mg, 0.040 mmol, 40%) a white solid. Mp 126-128 °C; ^{1}H-NMR (400 MHz; $(CD_3)_2CO$): δ 7.75 (s, 1H), 7.71-7.69 (m, 2H), 7.58 (t, *J* = 7.0 Hz, 1H), 6.92 (s, 1H), 6.48 (br s, 2H), 4.21 (s, 2H), 1.41 (s, 9H). ^{13}C-NMR (101 MHz; $(CD_3)_2CO$): δ 160.1, 146.6, 137.8, 132.3, 130.6, 129.7, 129.6, 124.8, 118.2, 112.5, 61.3, 33.8, 28.9. IR (neat): ṽ = 3322, 2923, 2852, 2228, 1356, 1159, 1114, 946, 758, 621 cm^{-1}. HRMS (ESI) m/z $(M-H)^-$ calcd for $C_{15}H_{17}N_4O_3S$ 333.1027, found 333.1028.

***N*-benzyl-*N*-(2-pentyloxazol-5-yl)sulfamide, (15j).**

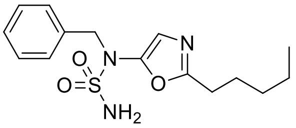

15j, 61%

The title compound was synthesized following the general procedure D. The crude material was purified by column chromatography using Cy/EtOAc 7:3 as eluent, affording compound **15j** (19.7

mg, 0.061 mmol, 61%) a white solid. Mp 125-127 °C; ^{1}H-NMR (400 MHz; CD_3OD): δ 7.31-7.29 (m, 5H), 6.68 (s, 1H), 4.70 (s, 2H), 2.66 (t, *J* = 7.4 Hz, 2H), 1.66 (quint, *J* = 7.4 Hz, 2H), 1.32 (quint, *J* = 7.4 Hz, 2H), 1.24 (sext, *J* = 7.4 Hz, 2H), 0.91 (t, *J* = 7.4 Hz, 3H). ^{13}C-NMR (101 MHz; $CDCl_3$): δ 163.7, 144.4, 134.9, 128.8, 128.7, 128.4, 122.7, 55.3, 31.1, 28.4, 26.4, 22.2, 13.9. IR (neat): ṽ = 3294, 2925, 2857, 1558, 1362, 1152, 1022, 934, 696, 536 cm^{-1}. HRMS (ESI) m/z $(M-H)^-$ calcd for $C_{15}H_{20}N_3O_3S$ 322.1231, found 322.1229.

***N*-benzyl-*N*-(2-benzyloxazol-5-yl)sulfamide, (15k).**

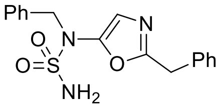

15k, 41%

The title compound was synthesized following the general procedure D. The crude material was purified by column chromatography using Cy/EtOAc 7:3 as eluent, affording compound **15k** (14.1 mg, 0.041 mmol, 41%) a white solid. Mp 154-156 °C; ^{1}H-NMR (400 MHz; $(CD_3)_2CO$): δ 7.34 (m, 3H), 7.27-7.25 (m, 5H), 7.20 (d, J = 7.0 Hz, 2H), 6.72 (s, 1H), 4.72 (s, 2H), 4.01 (s, 2H). ^{13}C-NMR (101 MHz; $(CD_3)_2CO$): δ 135.9, 128.6 (2C), 128.5, 128.4, 128.3 (2C), 127.7 (2C), 126.7, 122.2, 54.0, 34.3. IR (neat): ṽ = 3358, 3132, 3030, 1558, 1373, 1159, 1015, 919, 695, 541 cm^{-1}. HRMS (ESI) m/z $(M-H)^-$ calcd for $C_{17}H_{16}N_3O_3S$ 342.0918, found 342.0916.

***N*-benzyl-*N*-(2-benzyl-4-methyloxazol-5-yl)sulfamide, (15l).**

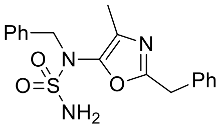

15l, 43%

The title compound was synthesized following the general procedure D. The crude material was purified by column chromatography using Cy/EtOAc 7:3 as eluent, affording compound **15l** (15.4 mg, 0.043 mmol, 43%) a white solid. Mp 127-129 °C; ^{1}H-NMR (400 MHz; $CDCl_3$): δ 7.36-7.31 (m, 5H), 7.24-7.19 (m, 3H), 7.15 (d, *J* = 6.2 Hz, 2H), 4.87 (br s, 2H), 4.60 (s, 2H), 4.00 (s, 2H), 1.83 (s, 3H). ^{13}C-NMR (101 MHz; $CDCl_3$): δ 160.6, 139.6, 135.3, 134.5, 133.7, 129.1, 128.7 (2C), 128.6, 128.4, 127.1, 54.8, 35.1, 10.7. IR (neat): ṽ = 3361, 3031, 2925, 1630, 1551, 1372, 1167, 692, 604, 531 cm^{-1}. HRMS (ESI) m/z $(M-H)^-$ calcd for $C_{18}H_{18}N_3O_3S$ 356.1074, found 356.1074.

Copies of ^{1}H and ^{13}C spectra

1a: ^{1}H (400 MHz, CDCl$_3$), ^{13}C (101 MHz, CDCl$_3$)

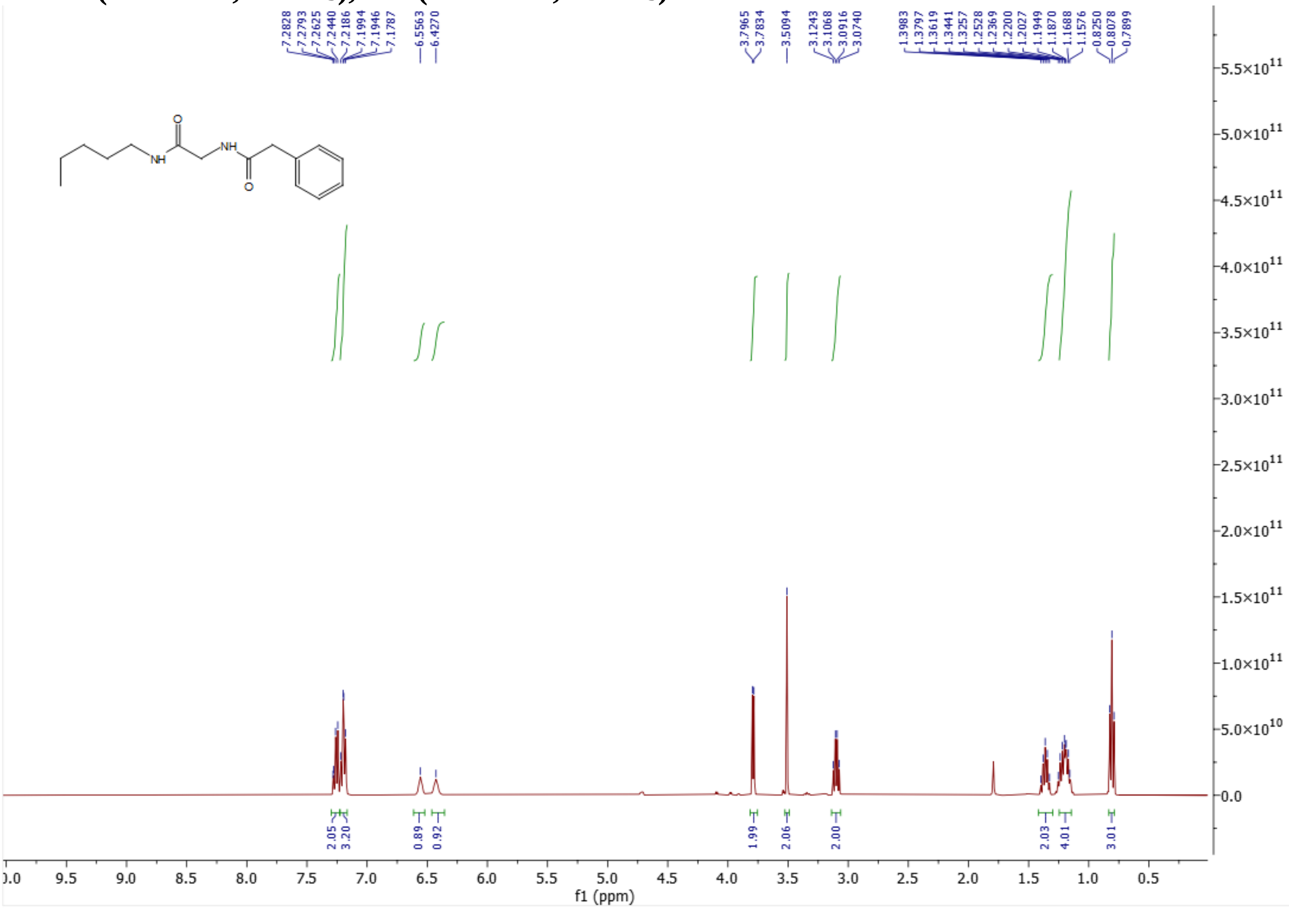

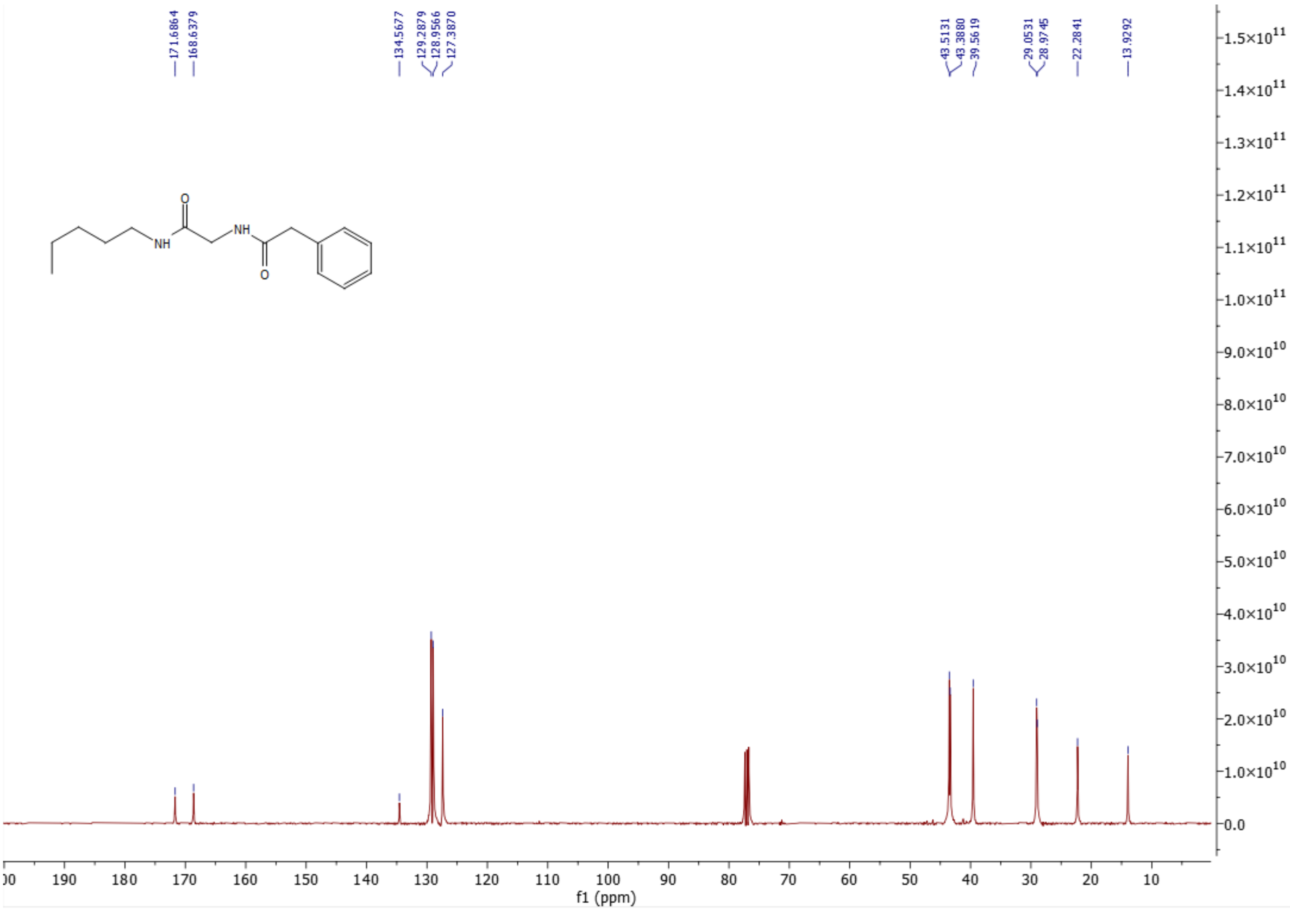

1b: ^{1}H (400 MHz, $CDCl_3$), ^{13}C (101 MHz, $CDCl_3$)

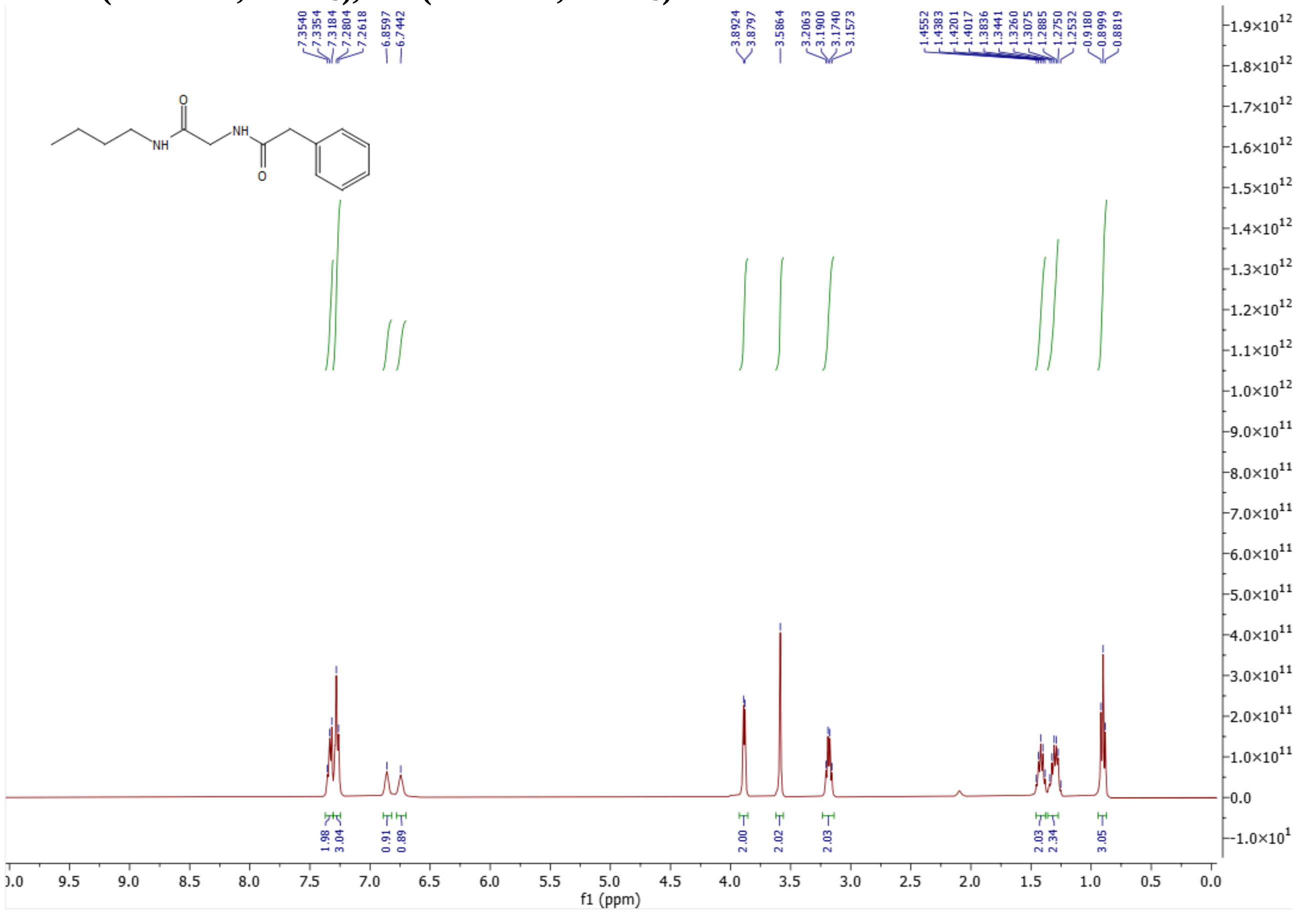

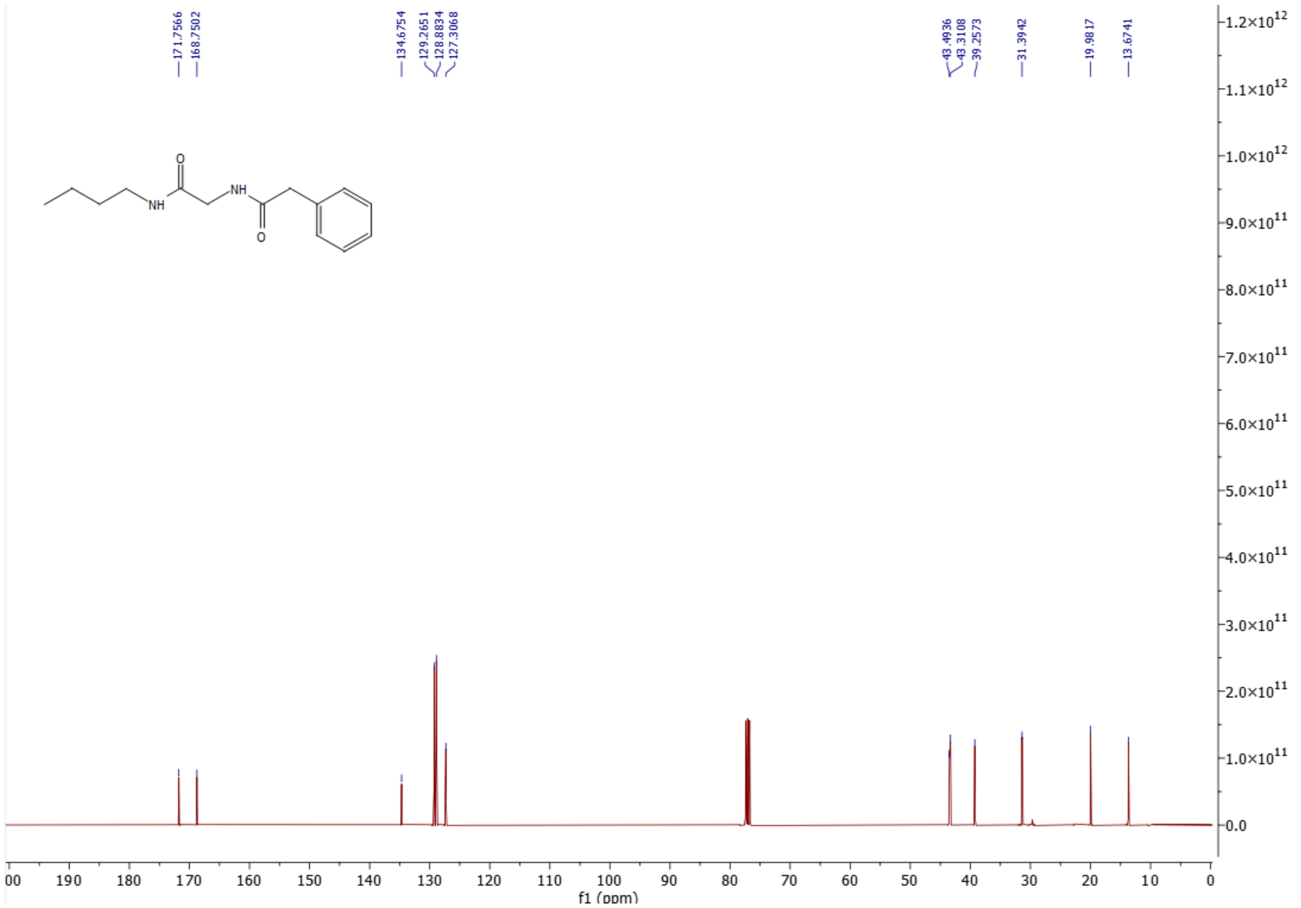

1c: ^{1}H (400 MHz, $CDCl_3$), ^{13}C (101 MHz, $CDCl_3$)

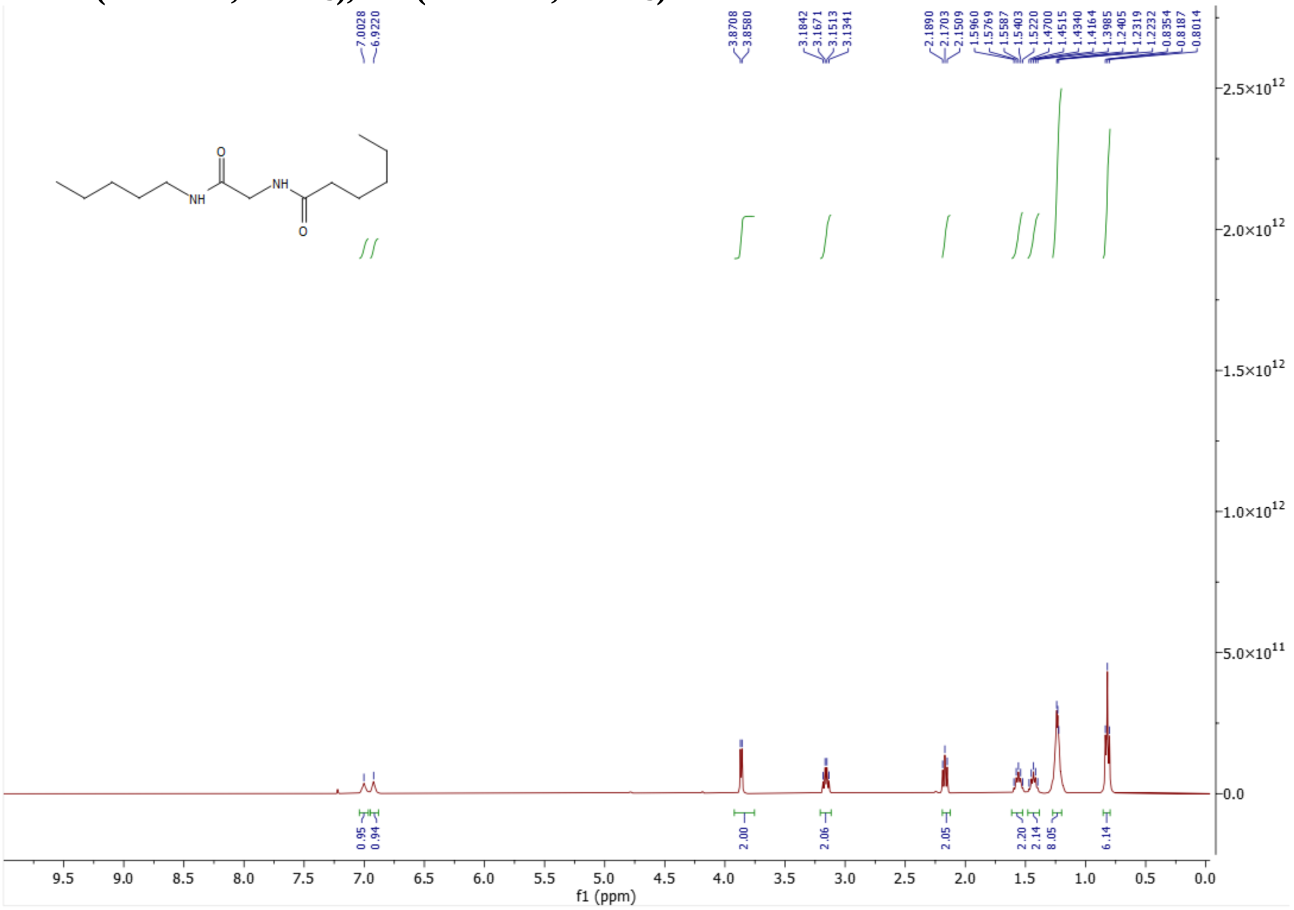

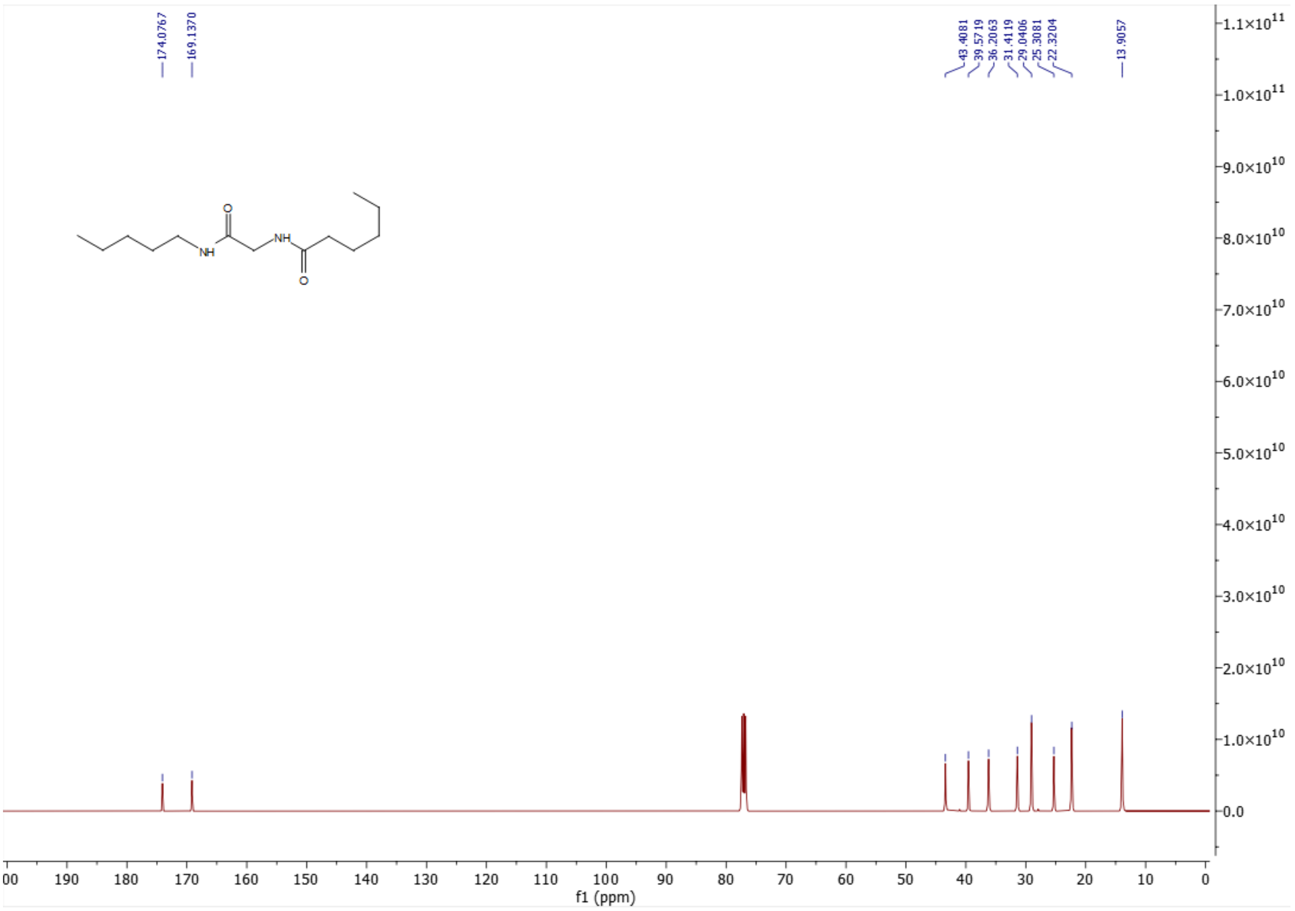

1d: ^{1}H (400 MHz, CD_3OD), ^{13}C (101 MHz, CD_3OD)

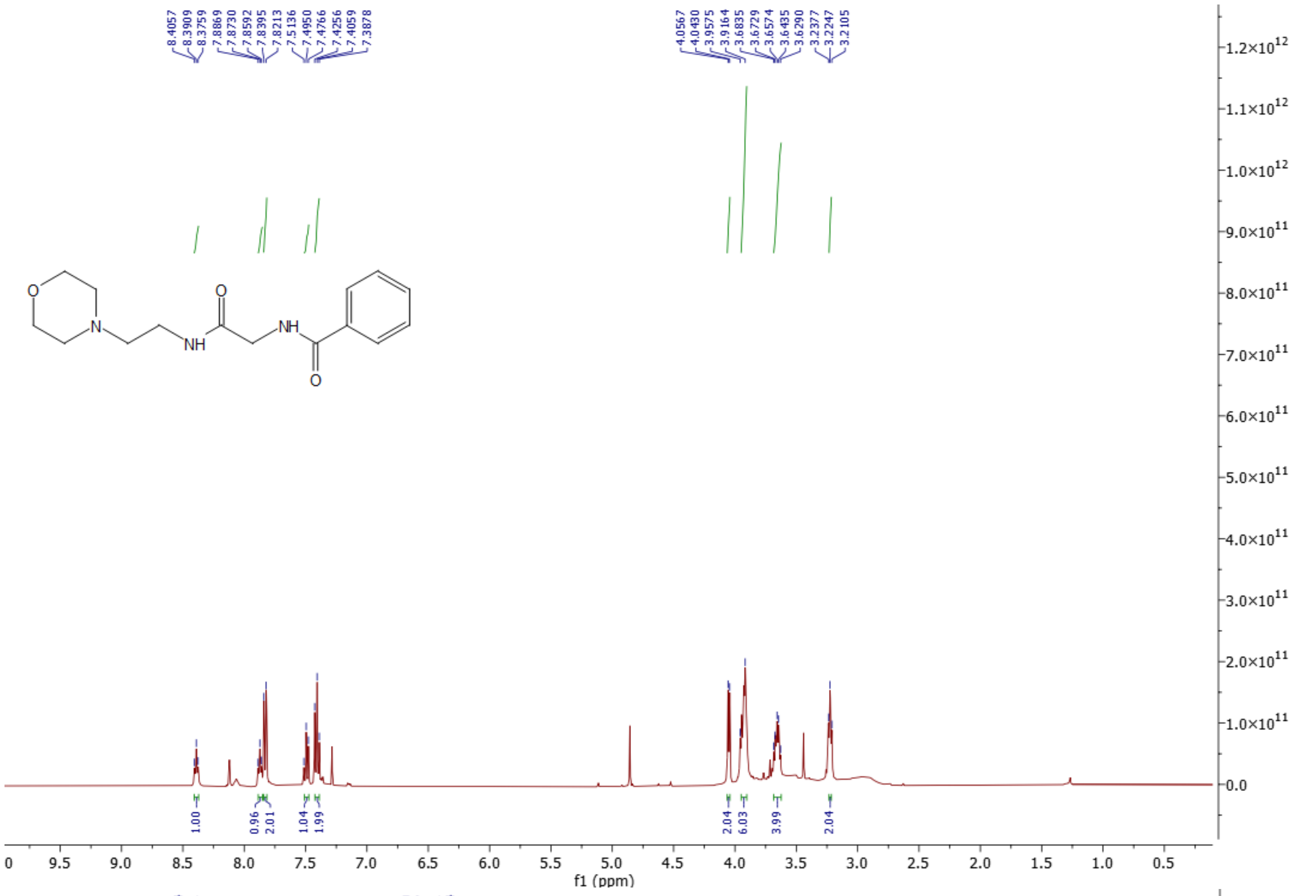

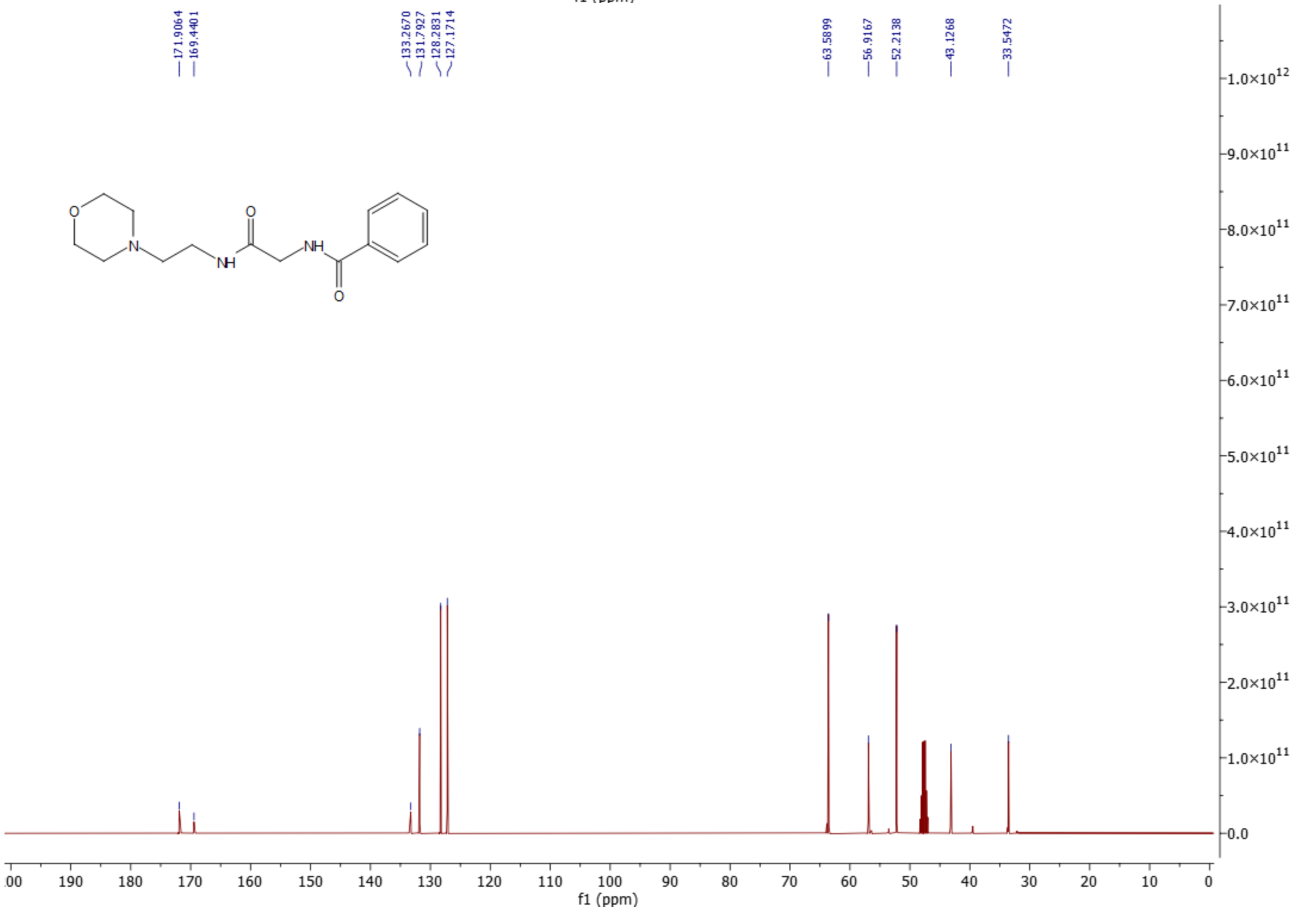

1e: ^{1}H (400 MHz, DMSO-d_6), ^{13}C (101 MHz, DMSO-d_6)

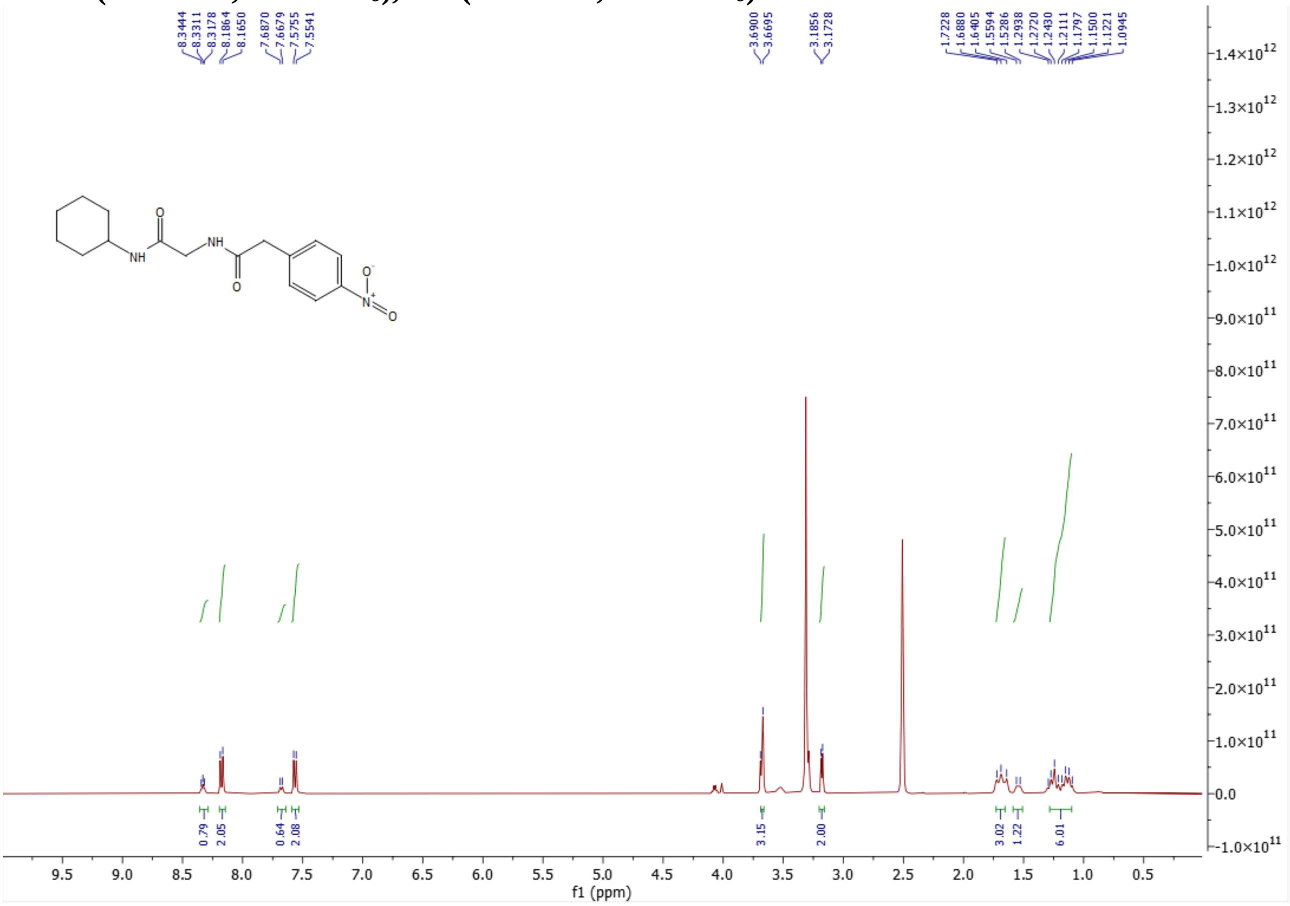

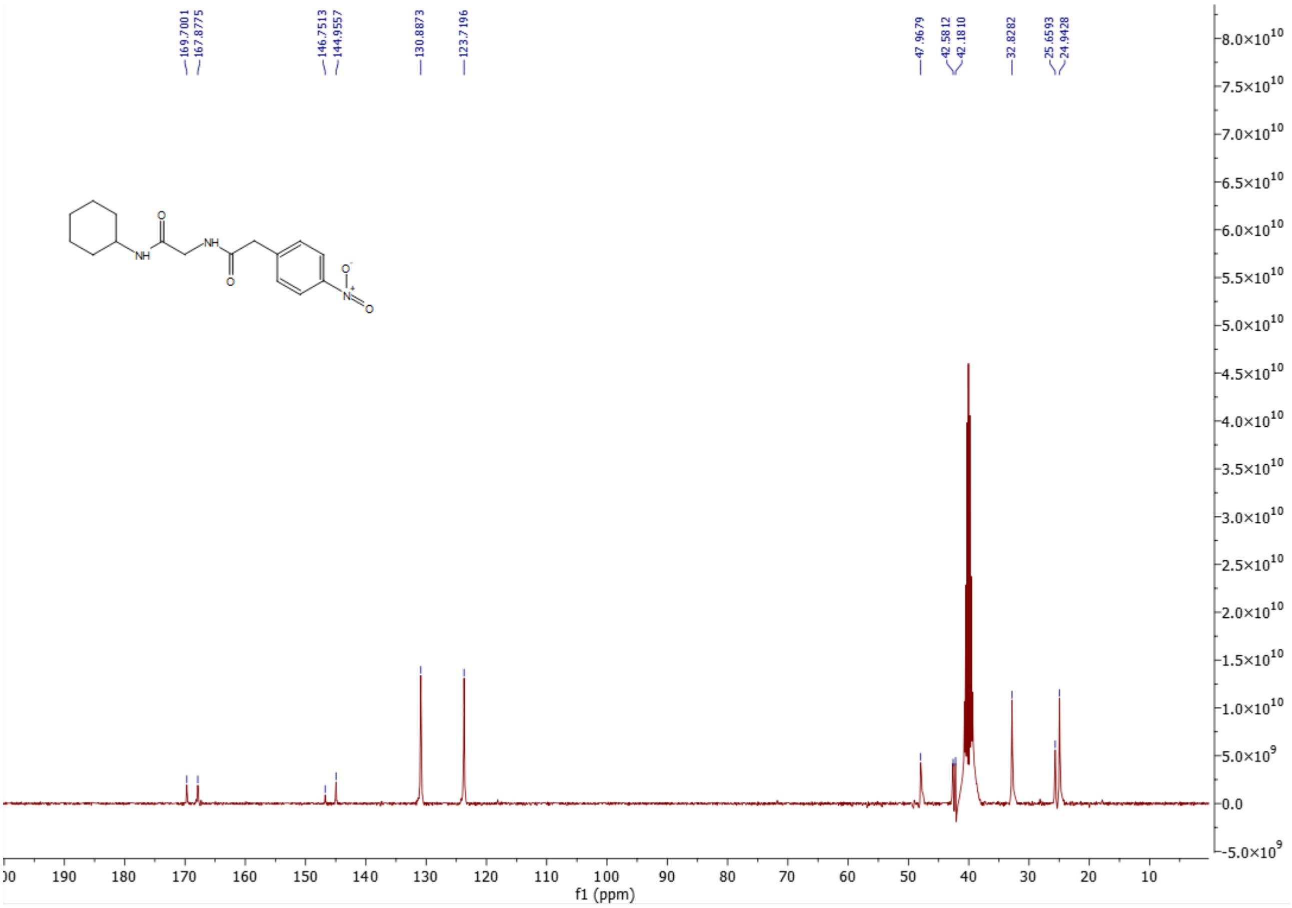

1f: ^{1}H (400 MHz, CDCl$_3$), ^{13}C (101 MHz, CDCl$_3$)

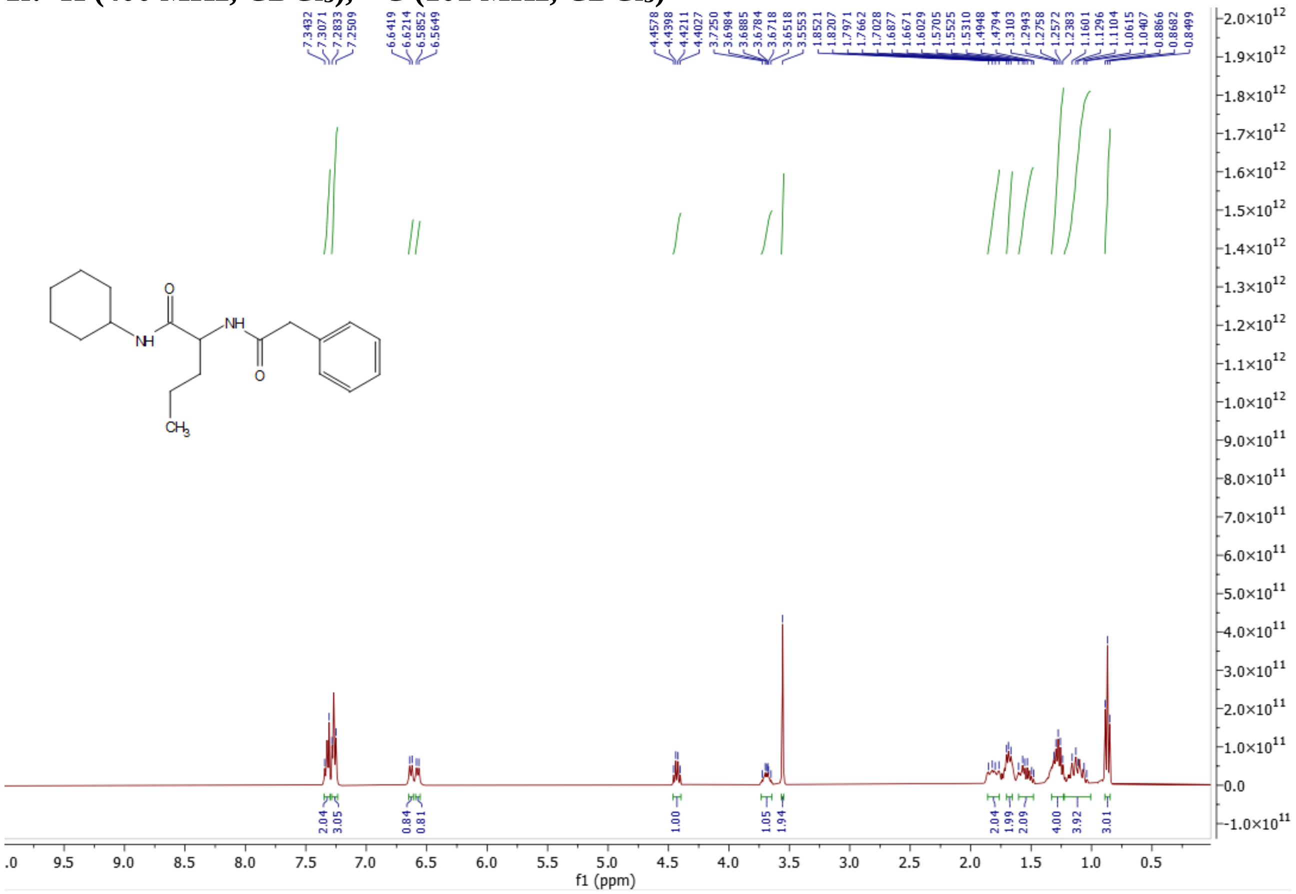

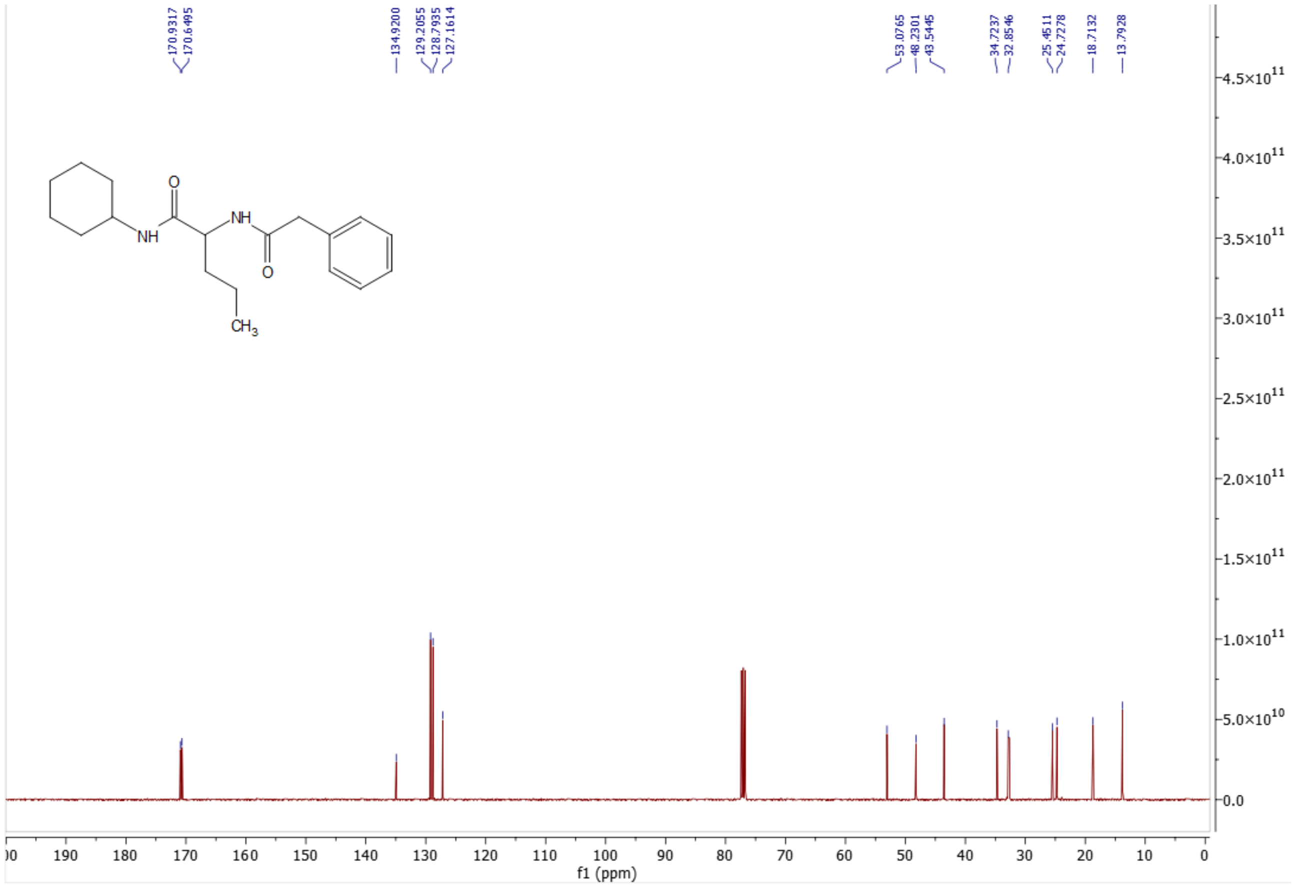

1g: ^{1}H (400 MHz, $CDCl_3$), ^{13}C (101 MHz, $CDCl_3$)

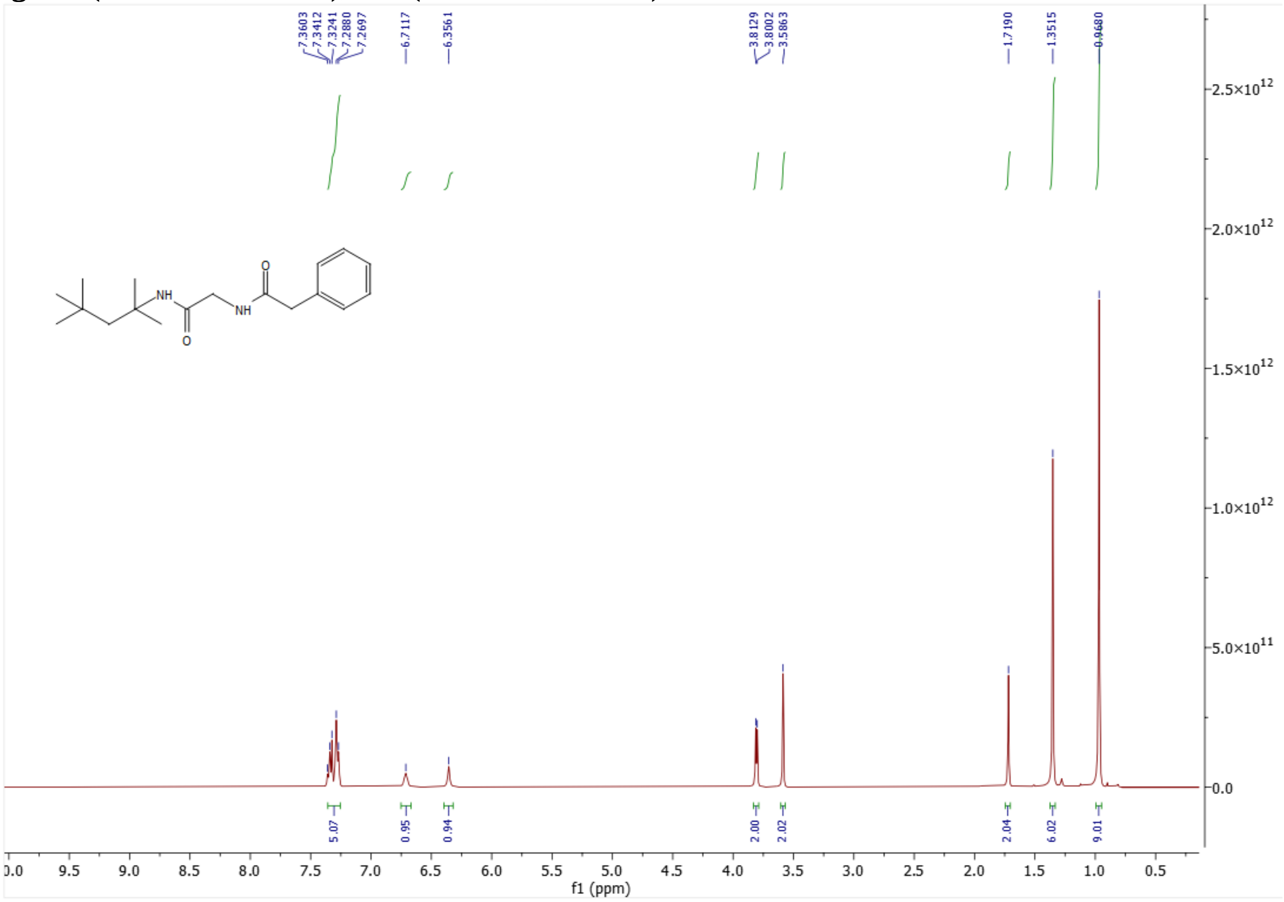

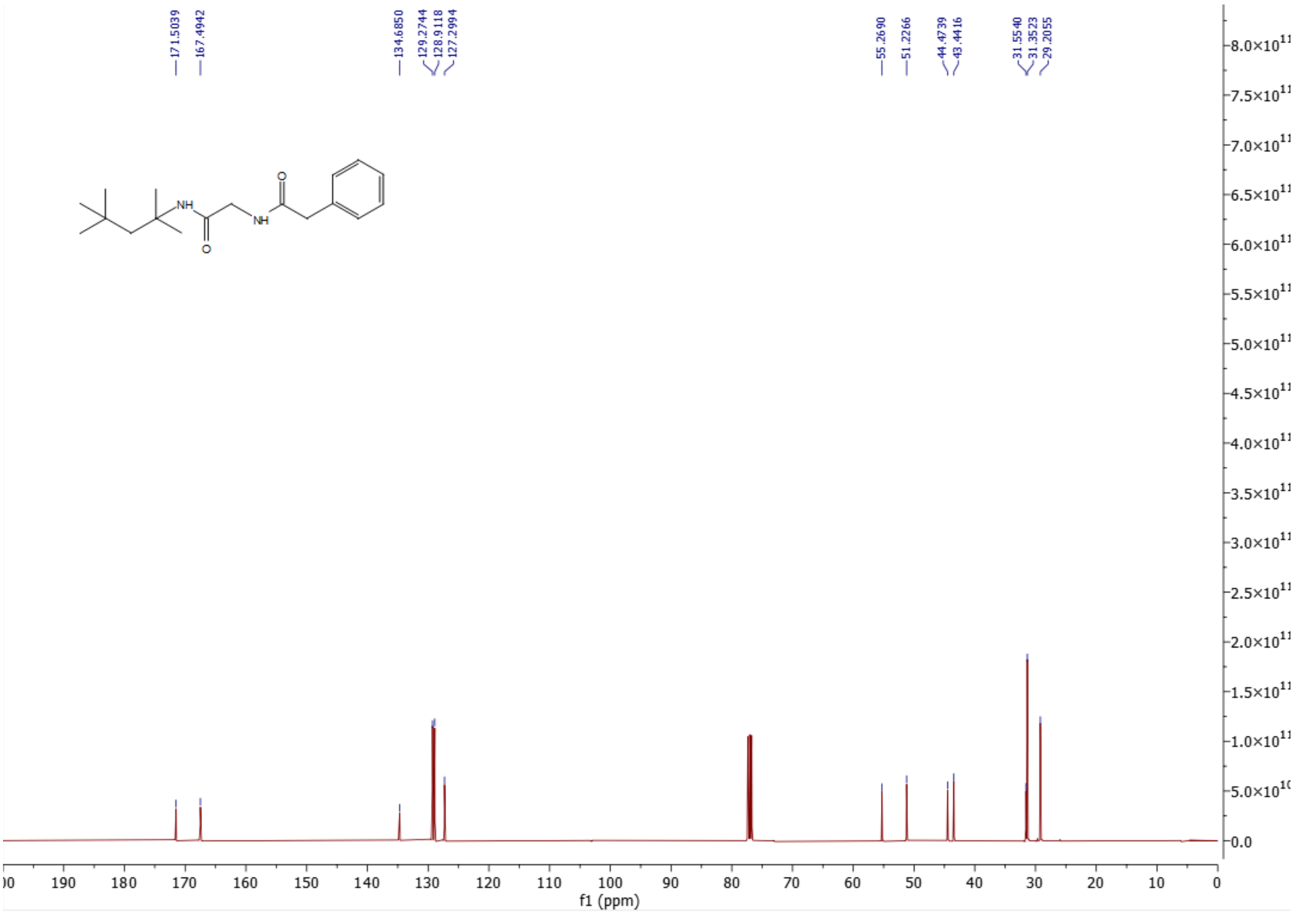

1h: ^{1}H (400 MHz, $CDCl_3$), ^{13}C (101 MHz, $CDCl_3$)

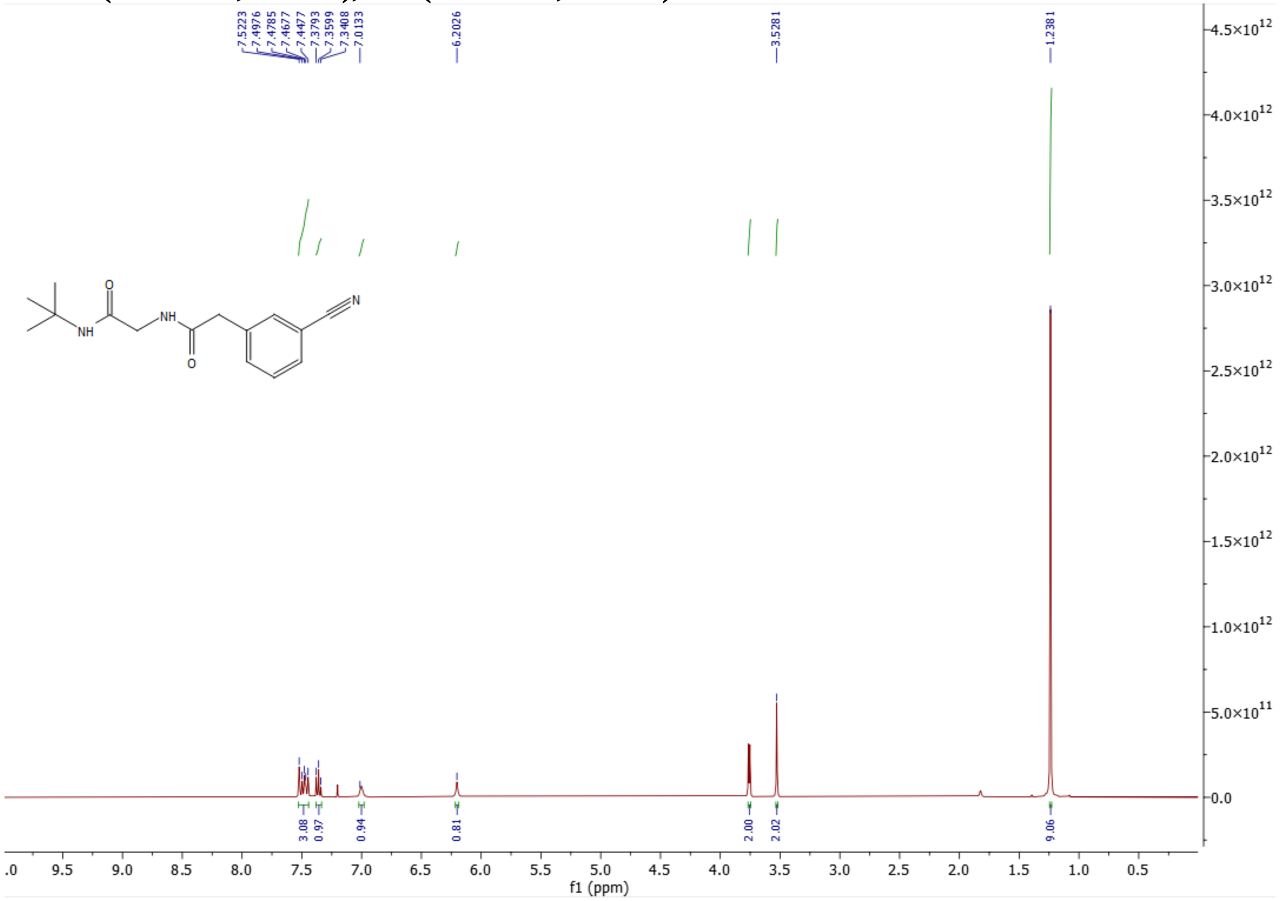

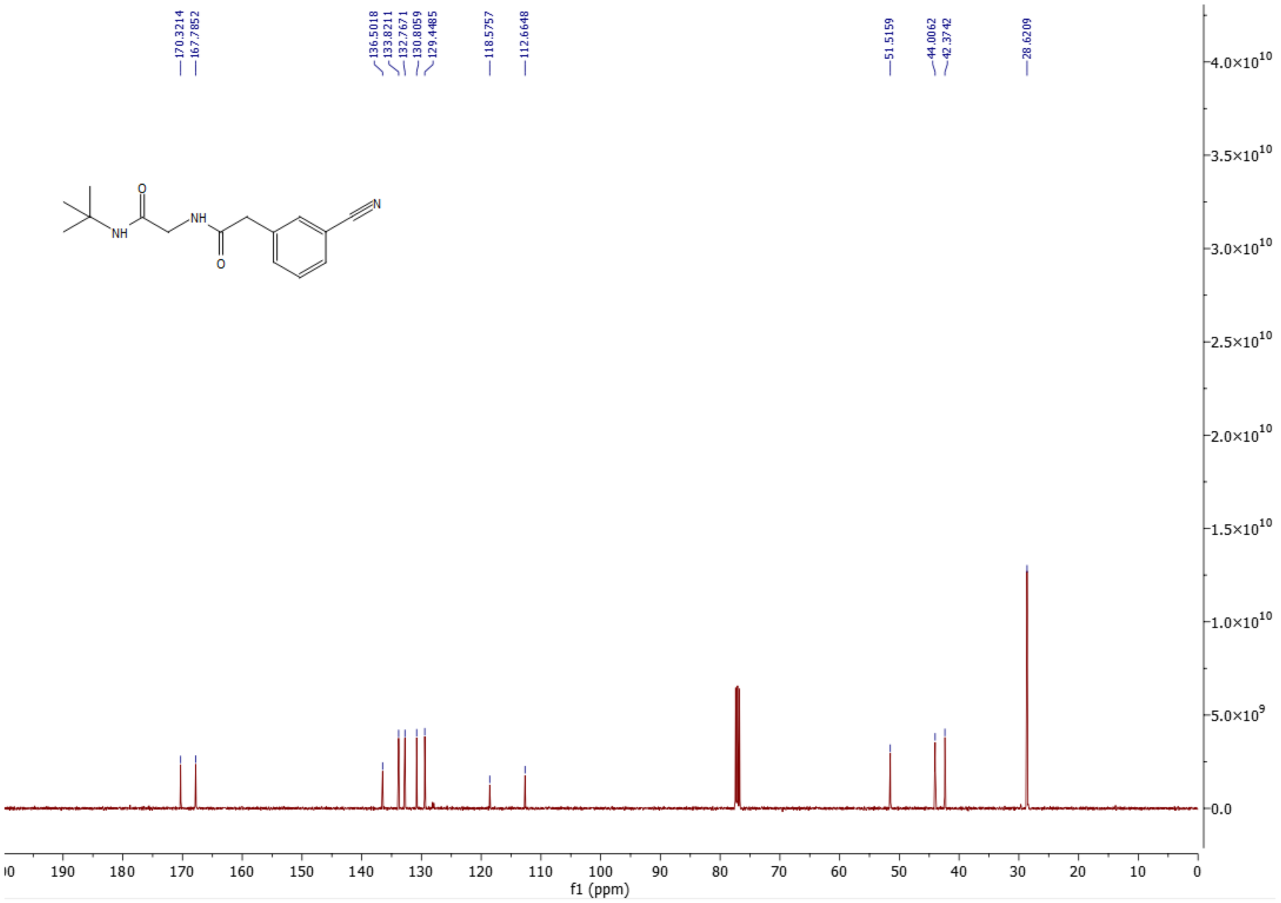

1i: ^{1}H (400 MHz, $CDCl_3$), ^{13}C (101 MHz, $CDCl_3$)

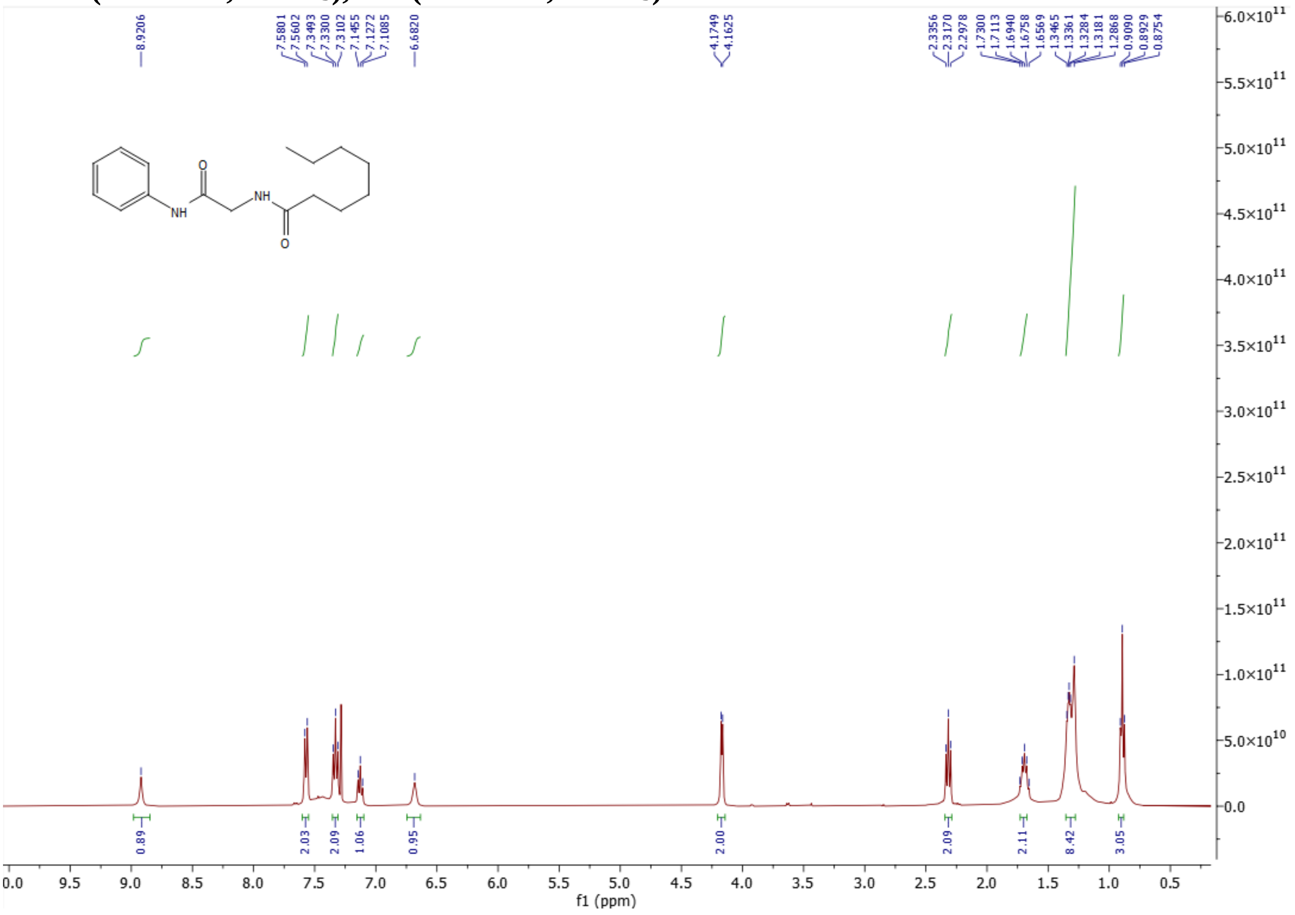

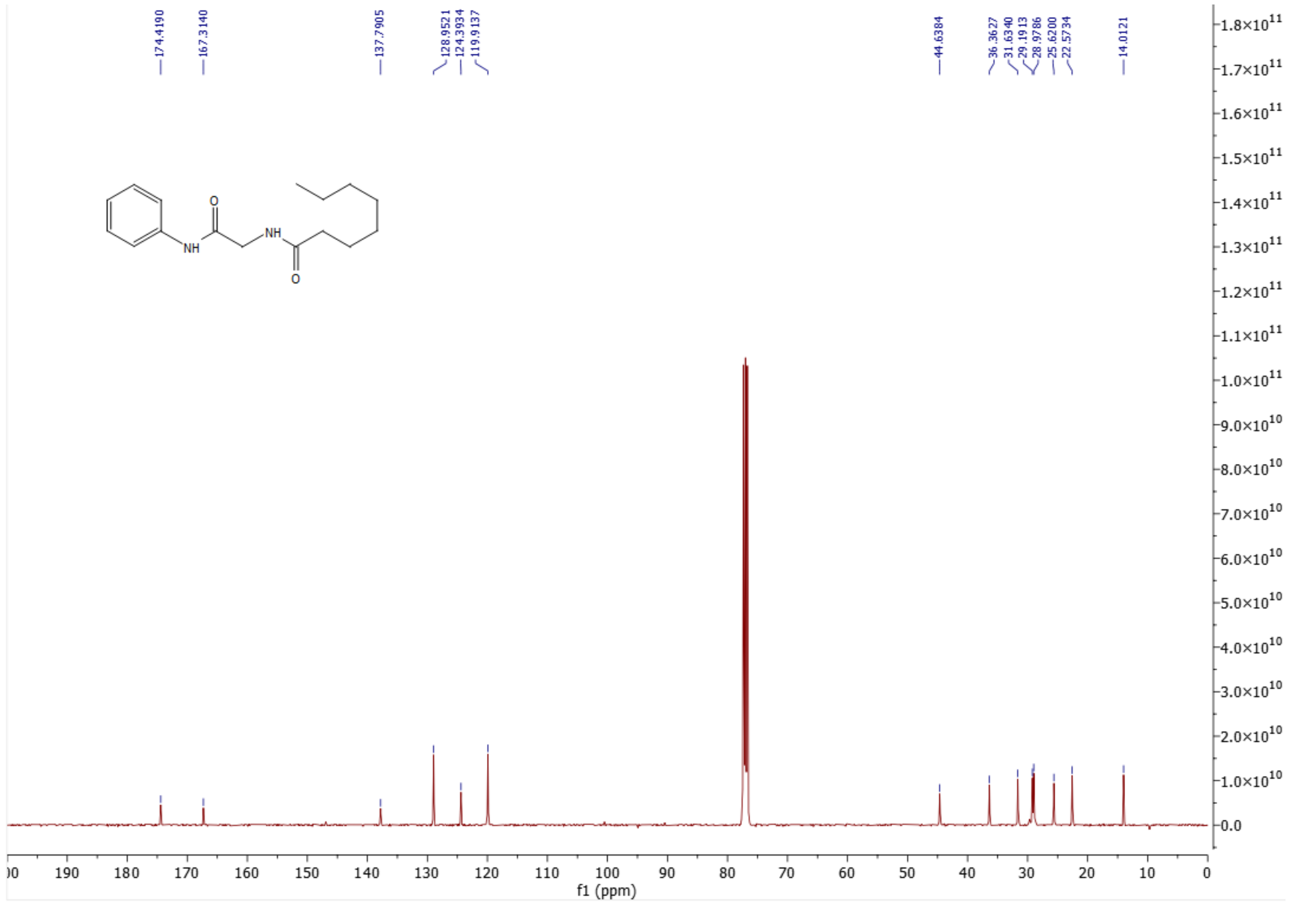

1j: ^{1}H (400 MHz, $CDCl_3$), ^{13}C (101 MHz, $CDCl_3$)

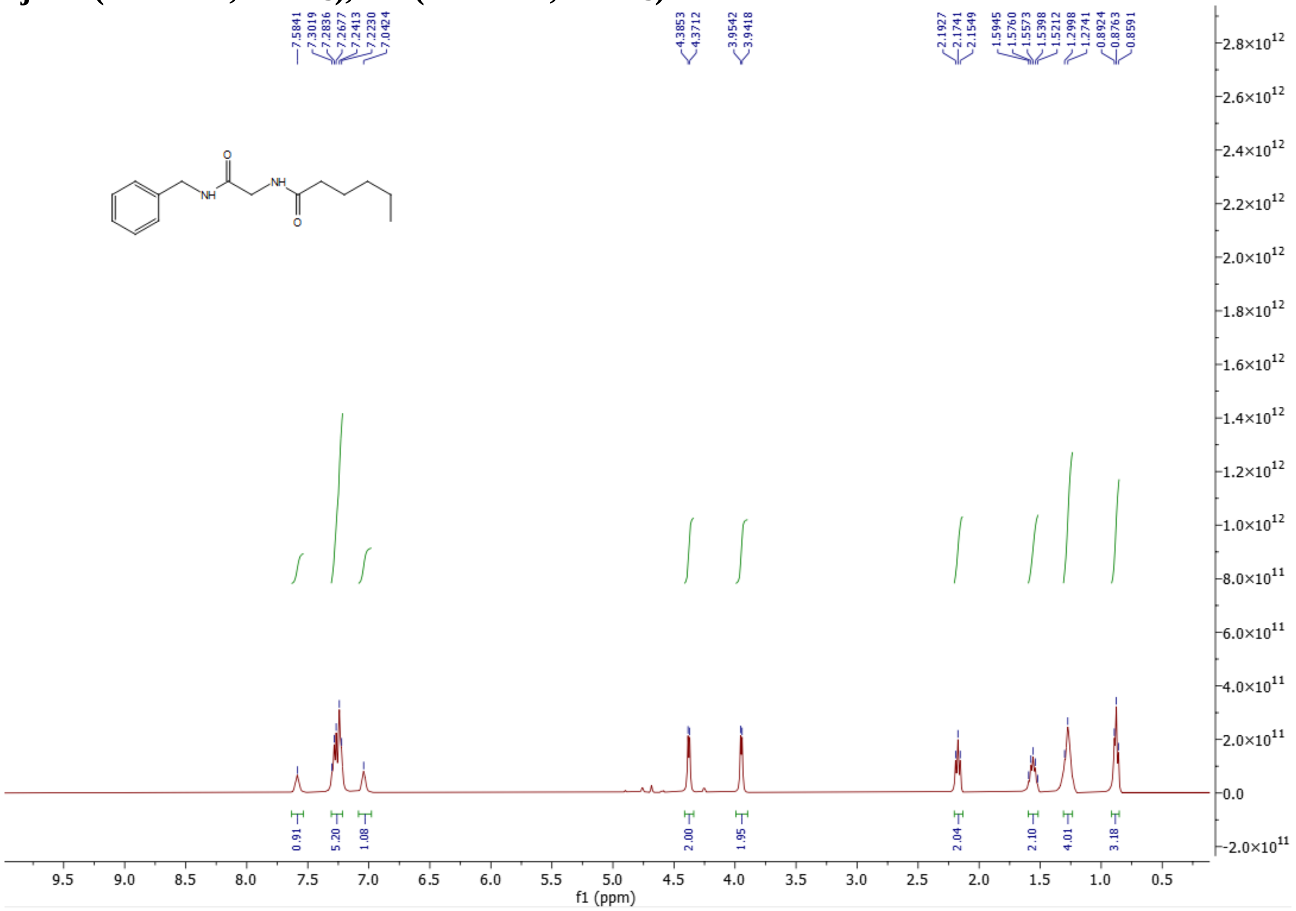

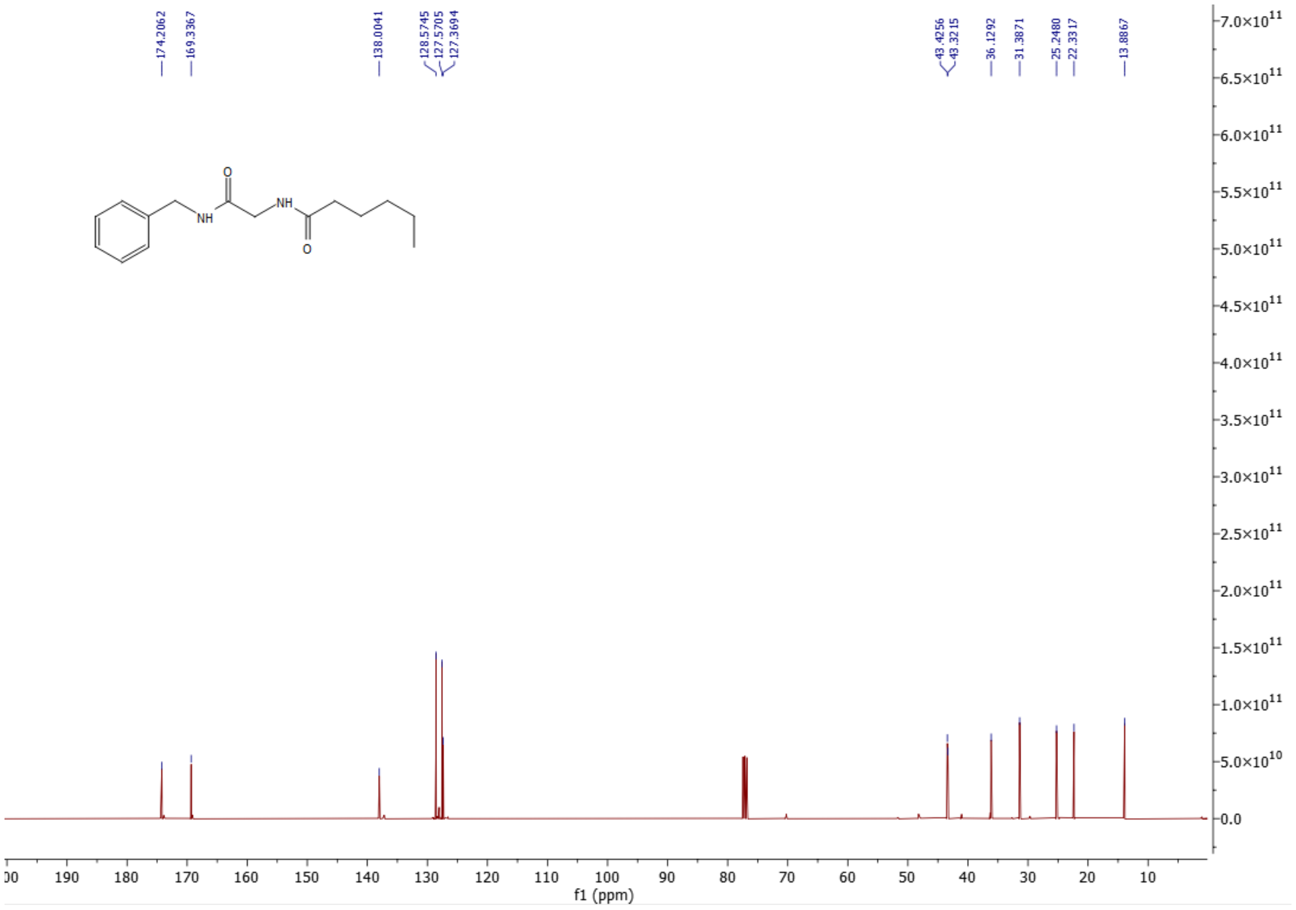

1k: ^{1}H (400 MHz, DMSO-d_6), ^{13}C (101 MHz, DMSO-d_6)

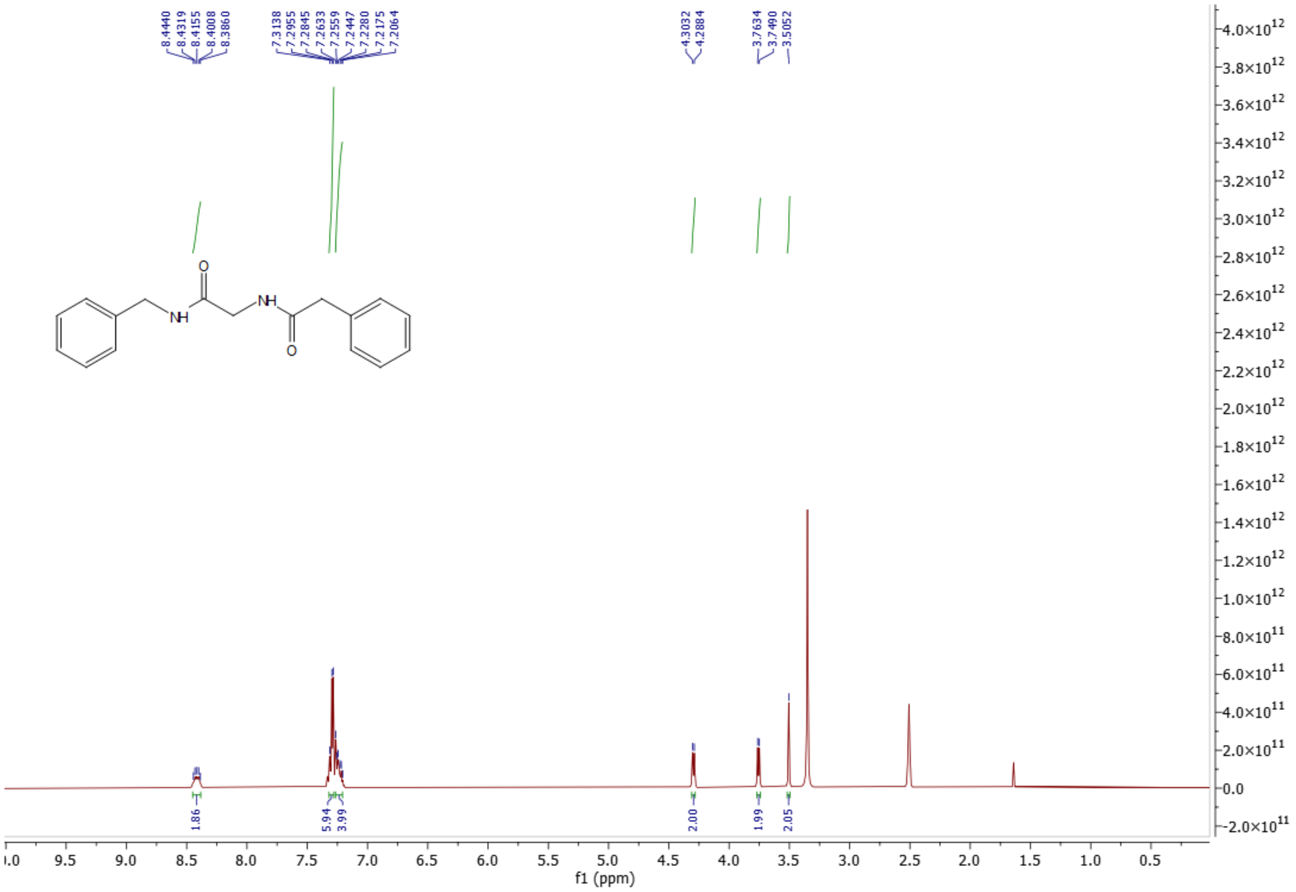

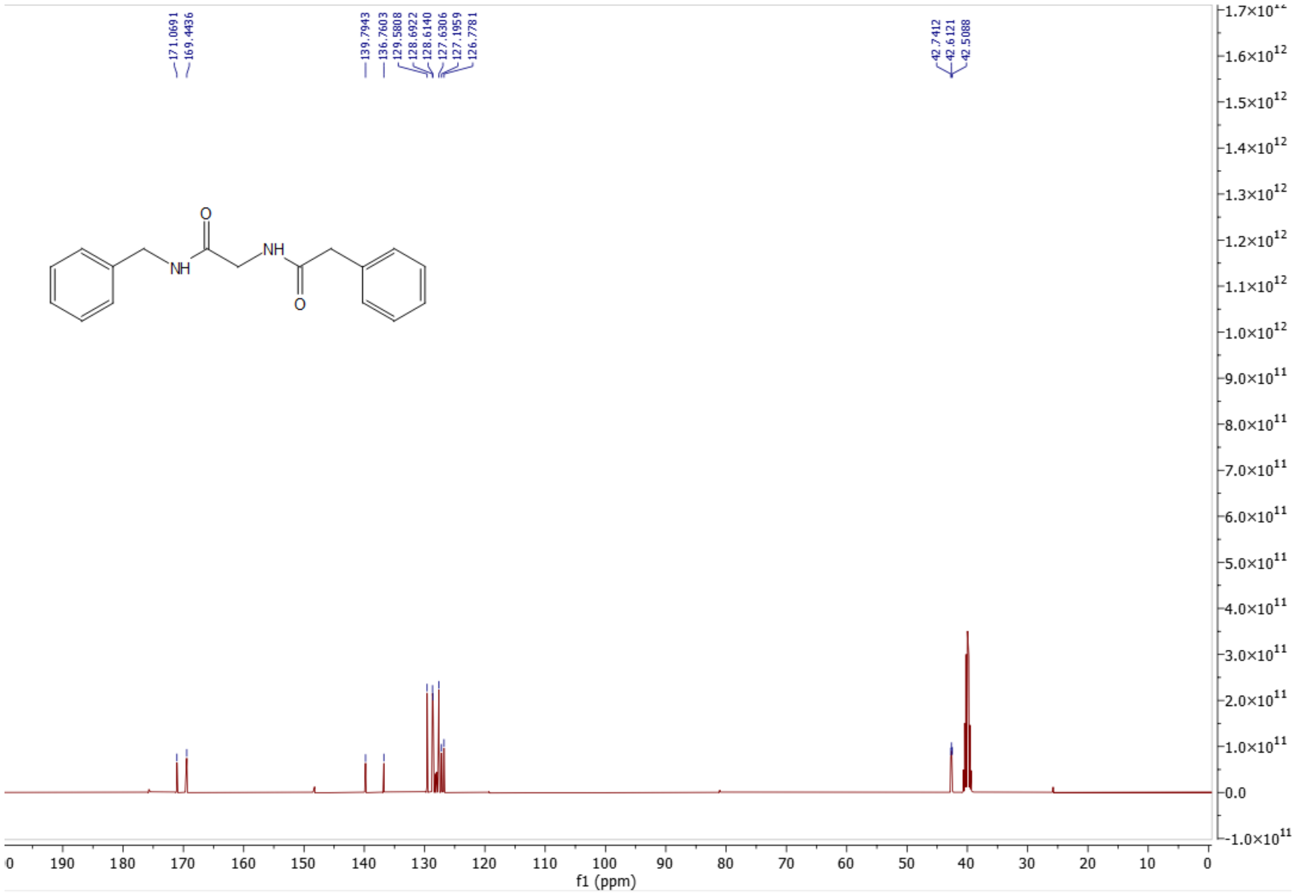

1l: ^{1}H (400 MHz, $CDCl_3$), ^{13}C (101 MHz, $CDCl_3$)

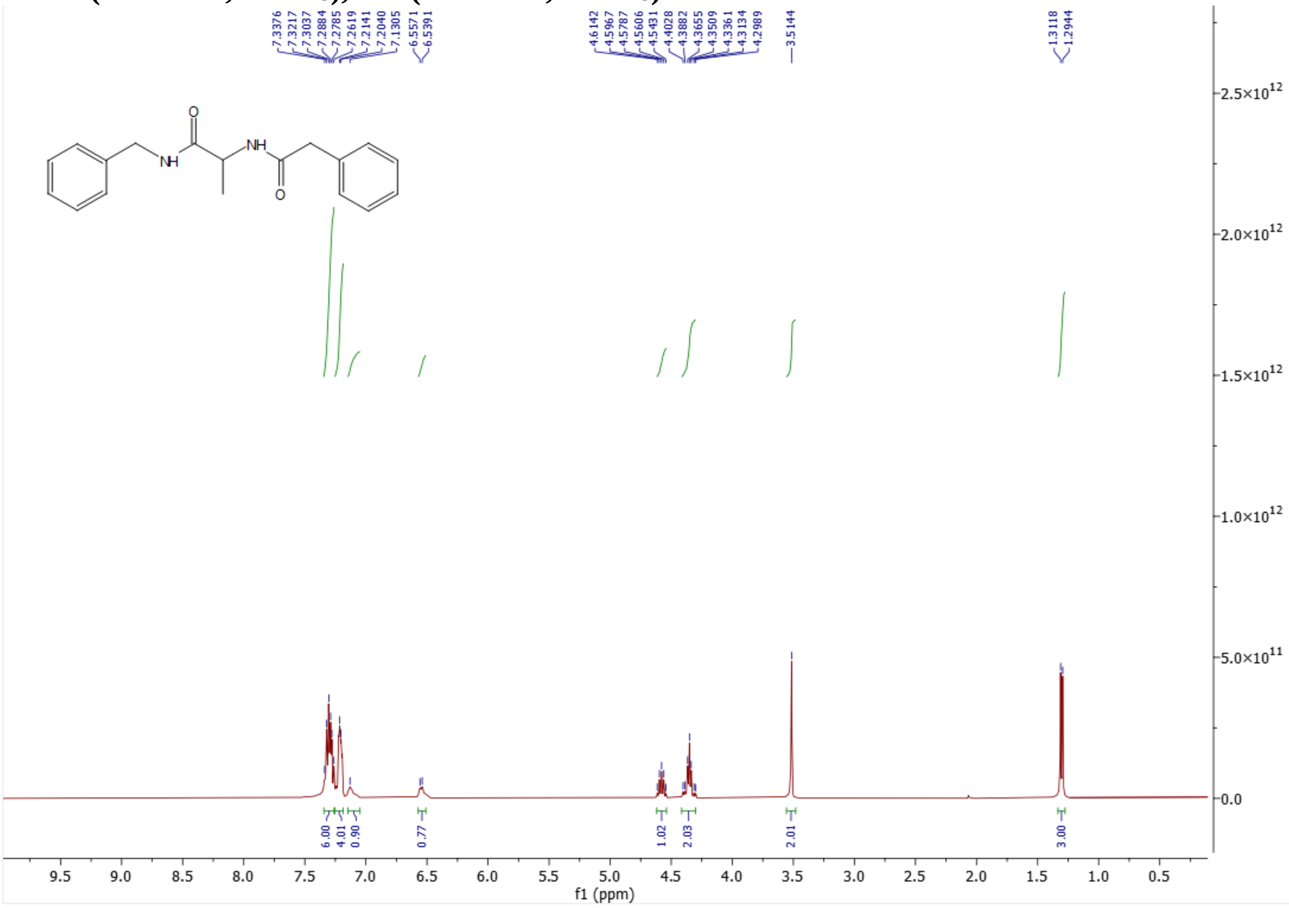

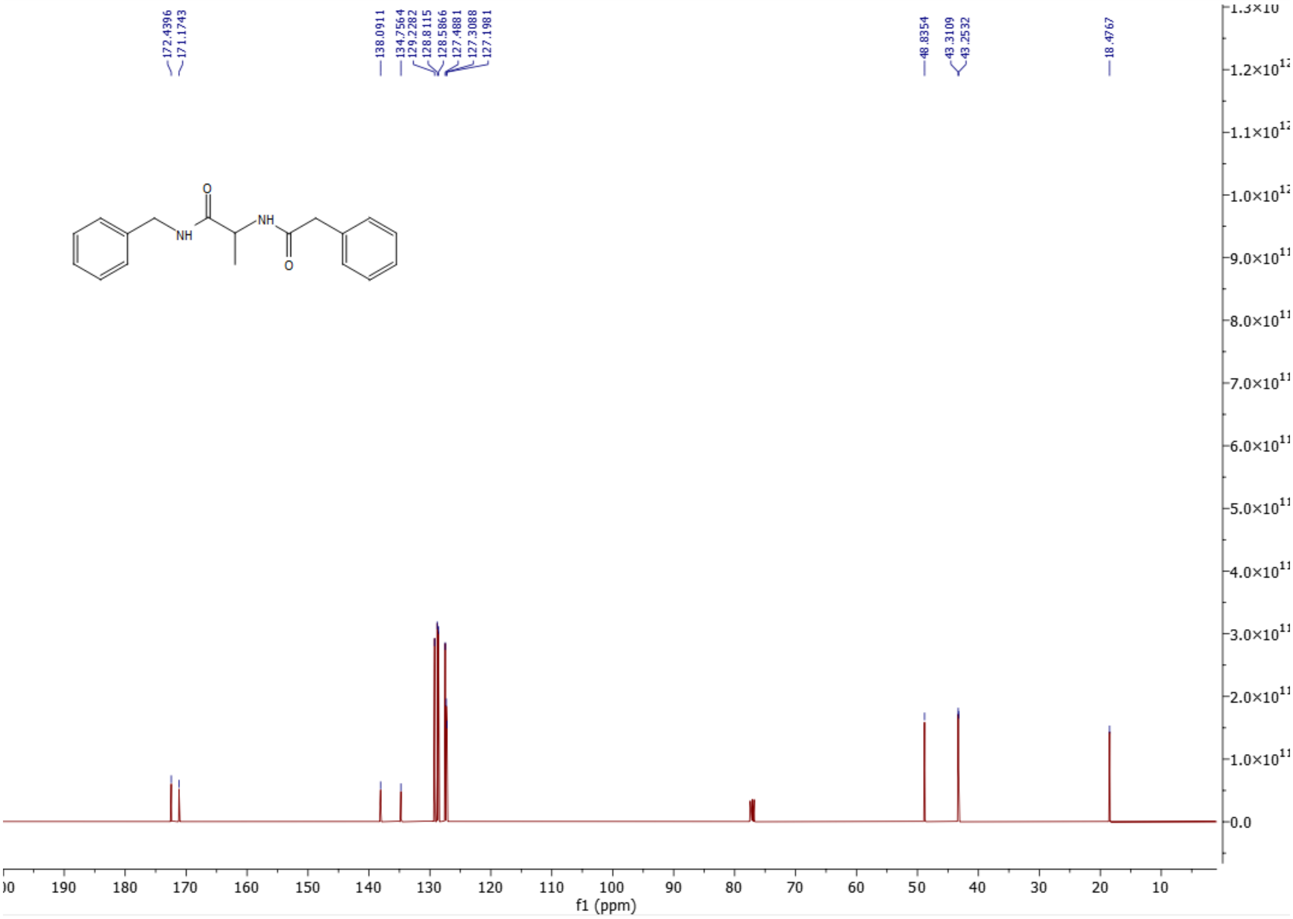

1m: ^{1}H (400 MHz, CDCl$_3$), ^{13}C (101 MHz, CDCl$_3$)

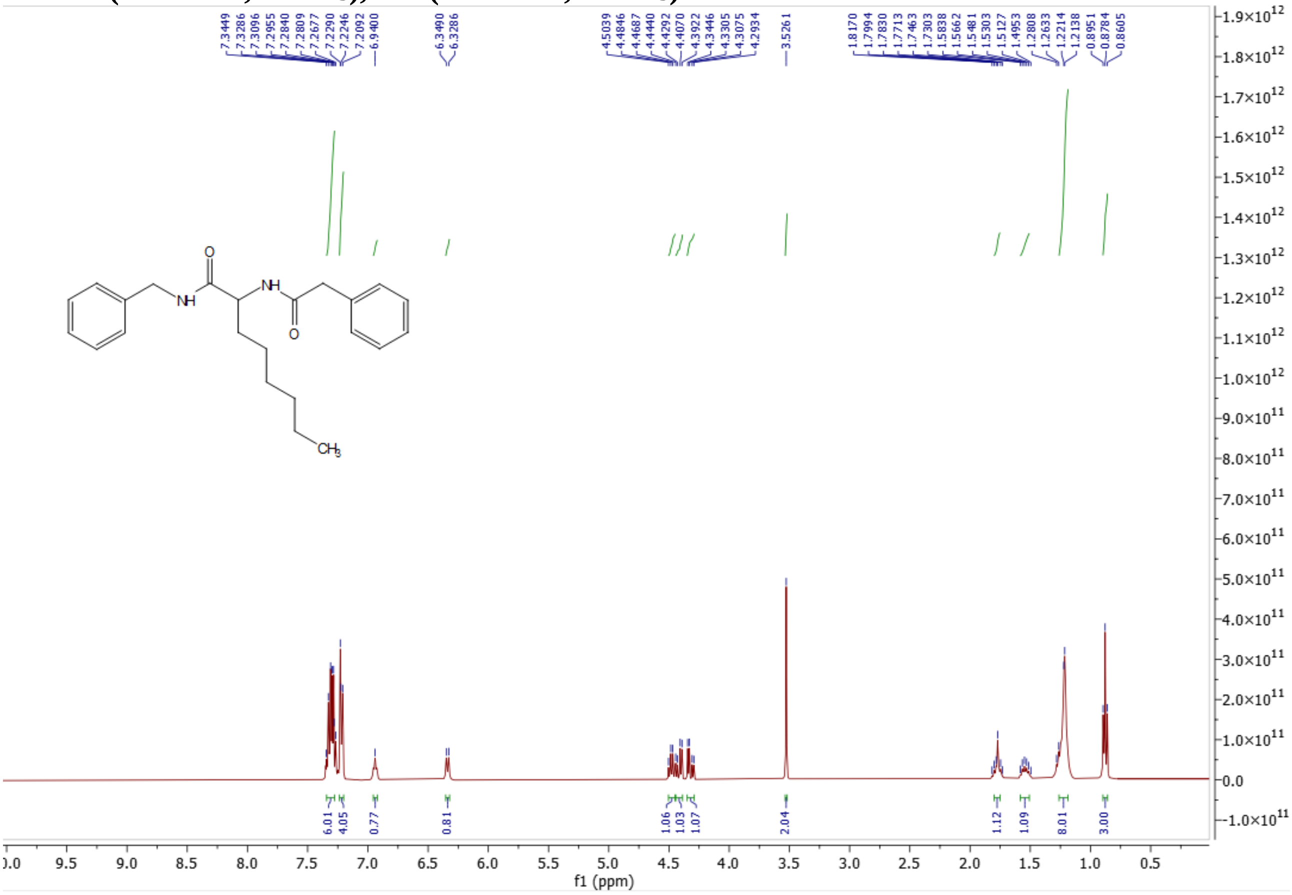

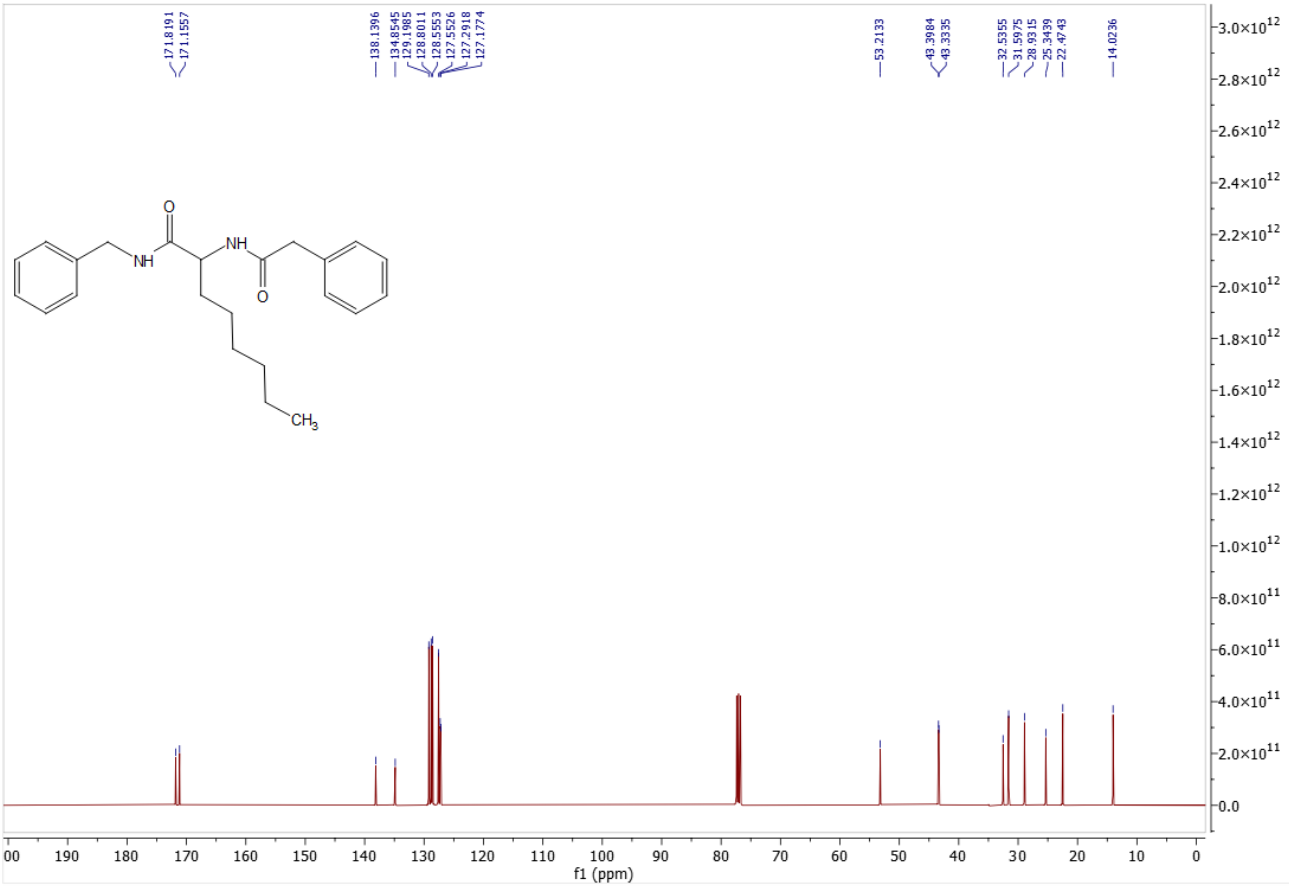

1n: ^{1}H (400 MHz, CD_3OD), ^{13}C (101 MHz, CD_3OD)

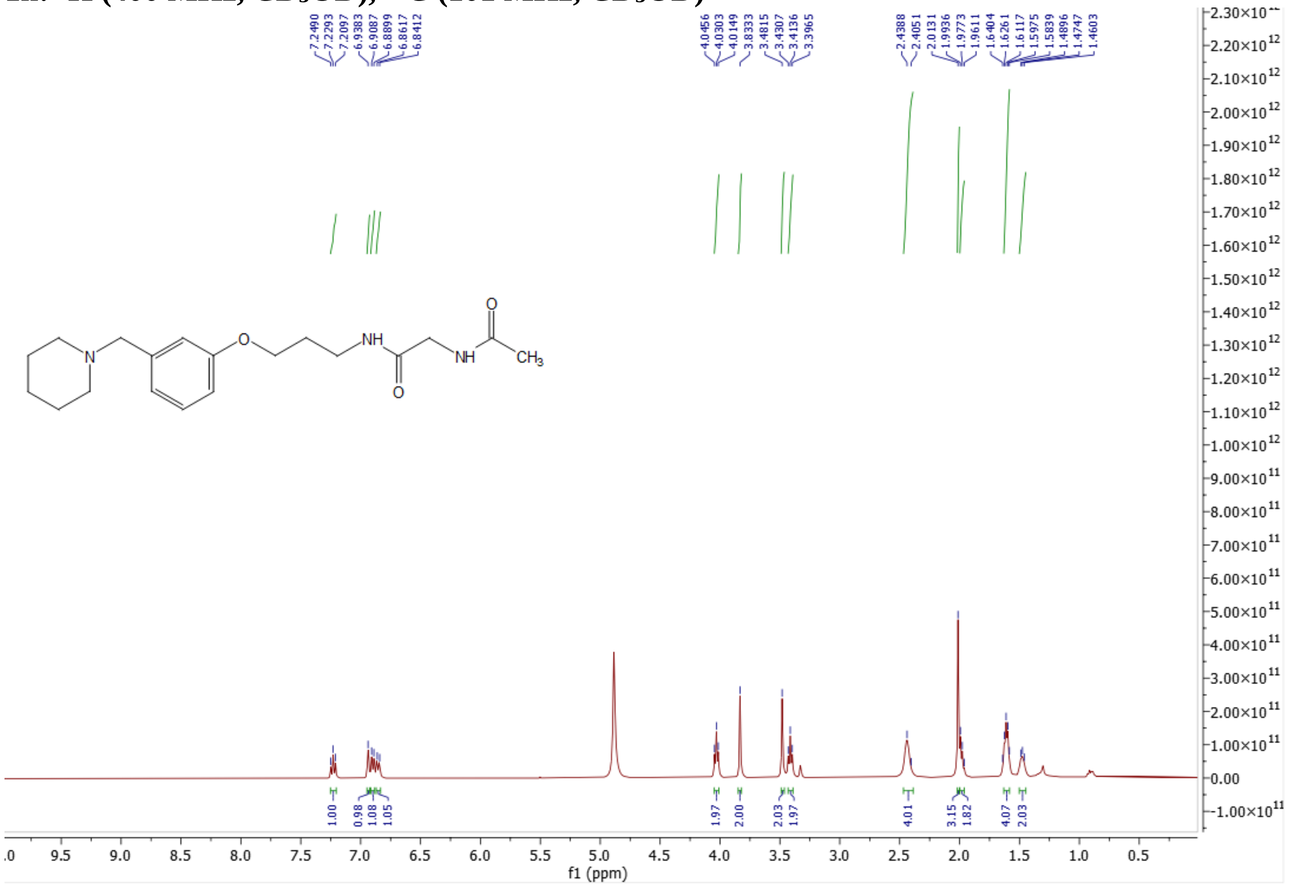

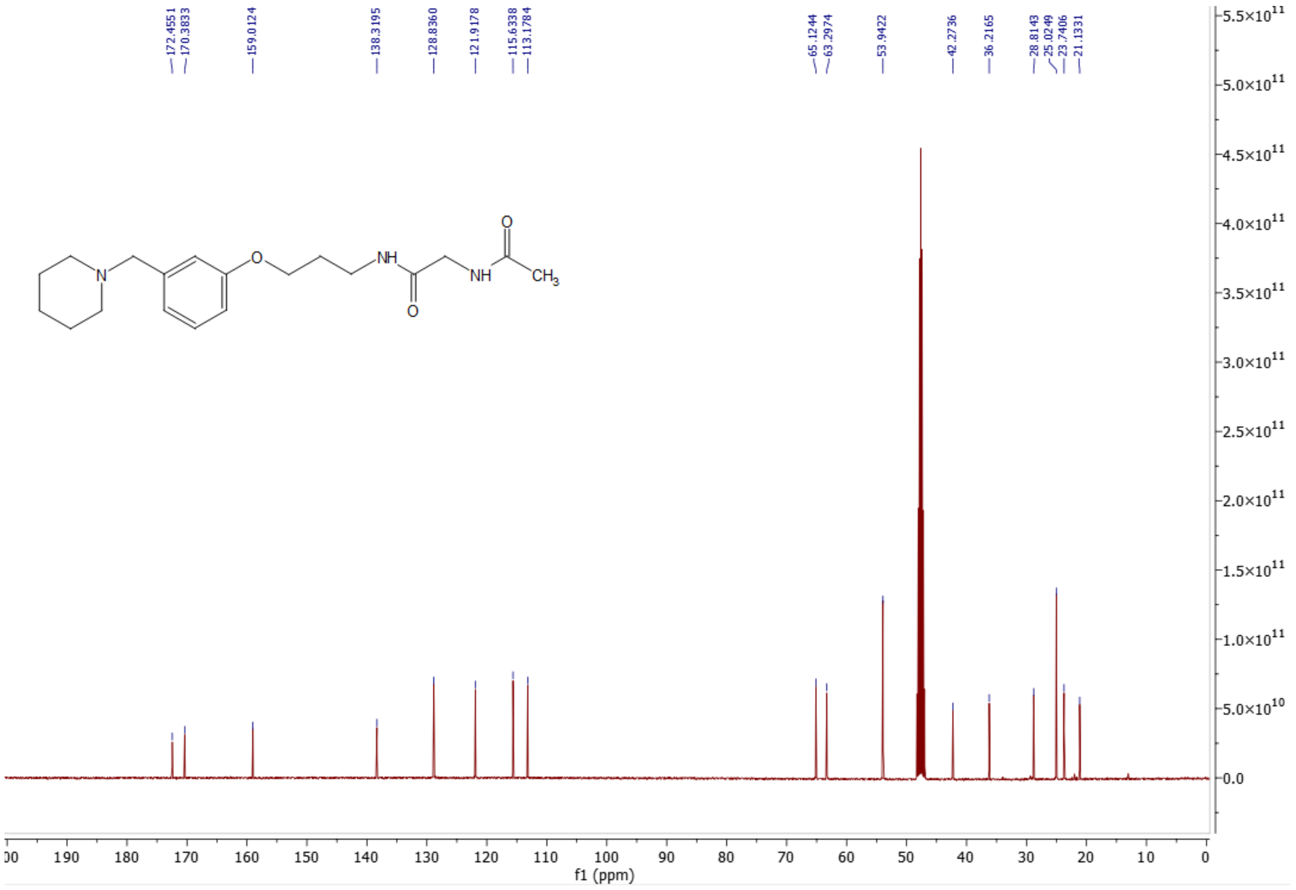

1o: ^{1}H (400 MHz, CD_3OD), ^{13}C (101 MHz, $(CD_3)_2CO$)

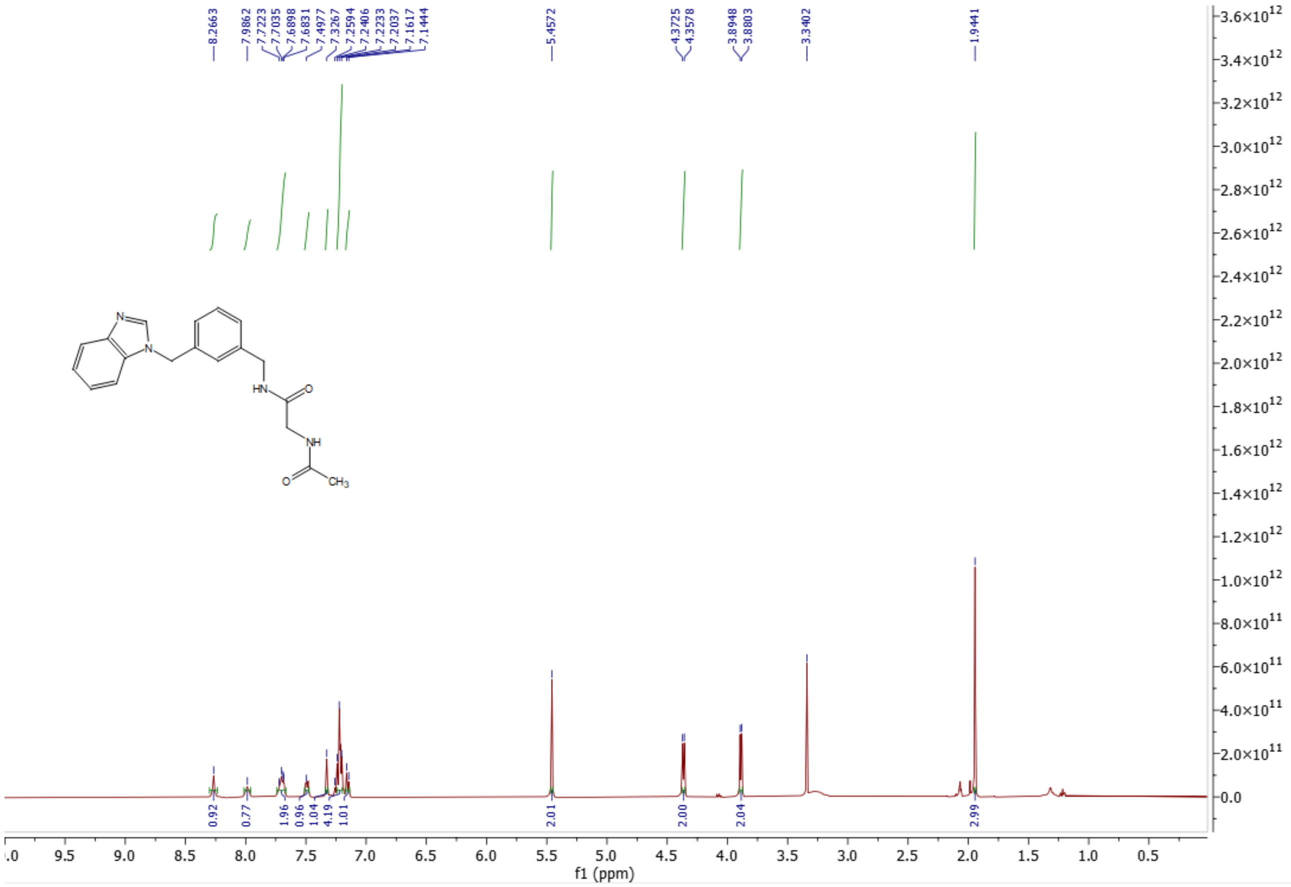

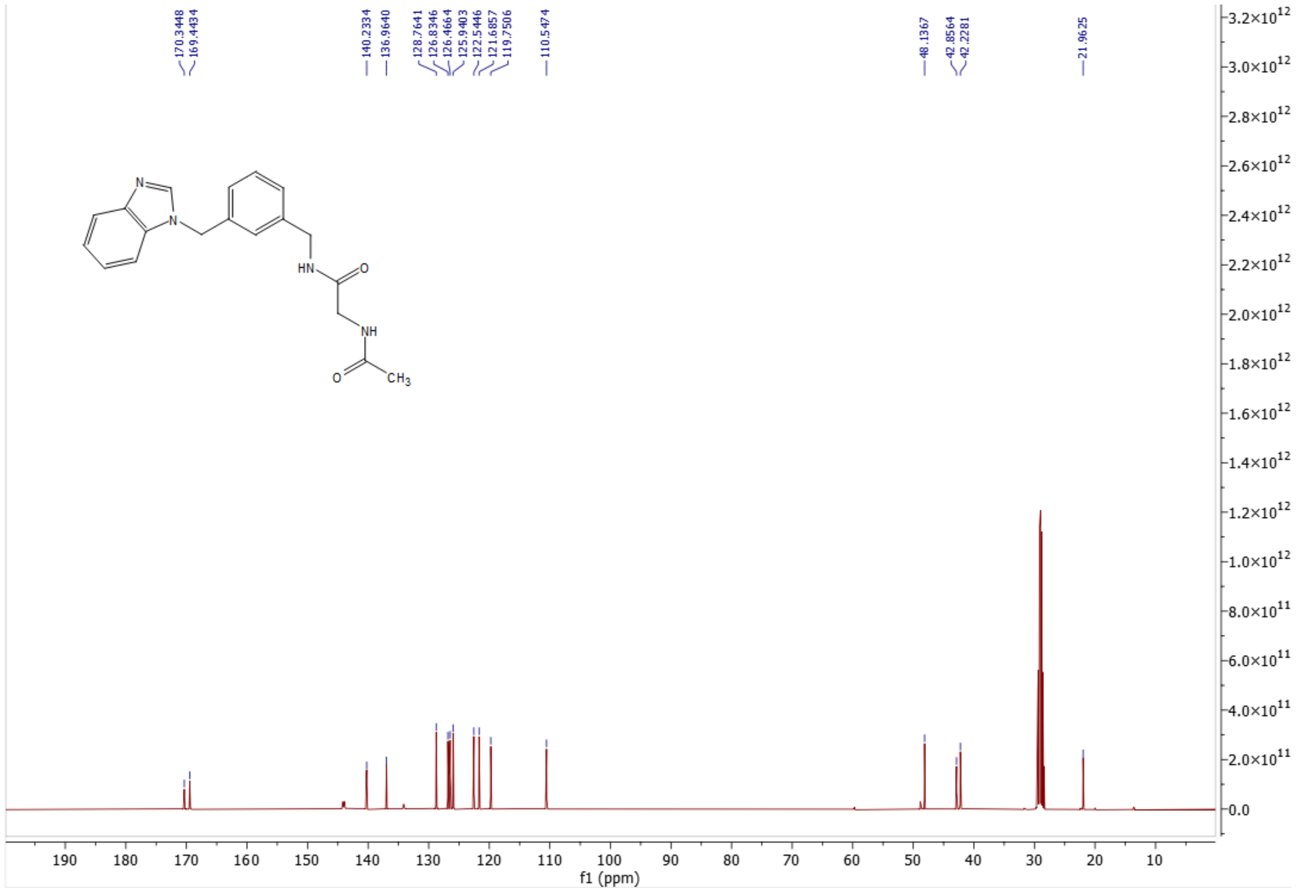

1p: ^{1}H (400 MHz, CD_3OD), ^{13}C (101 MHz, CD_3OD)

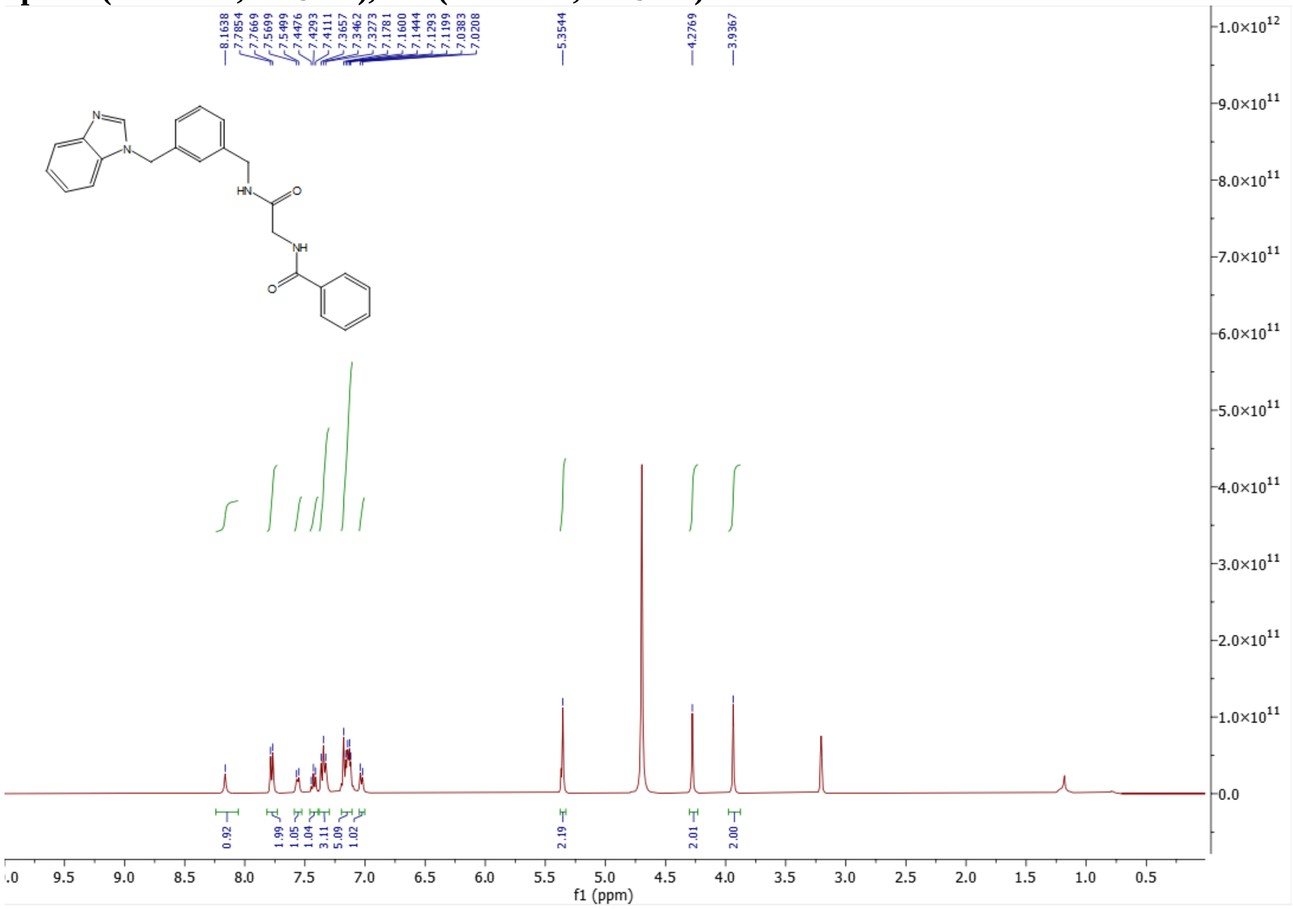

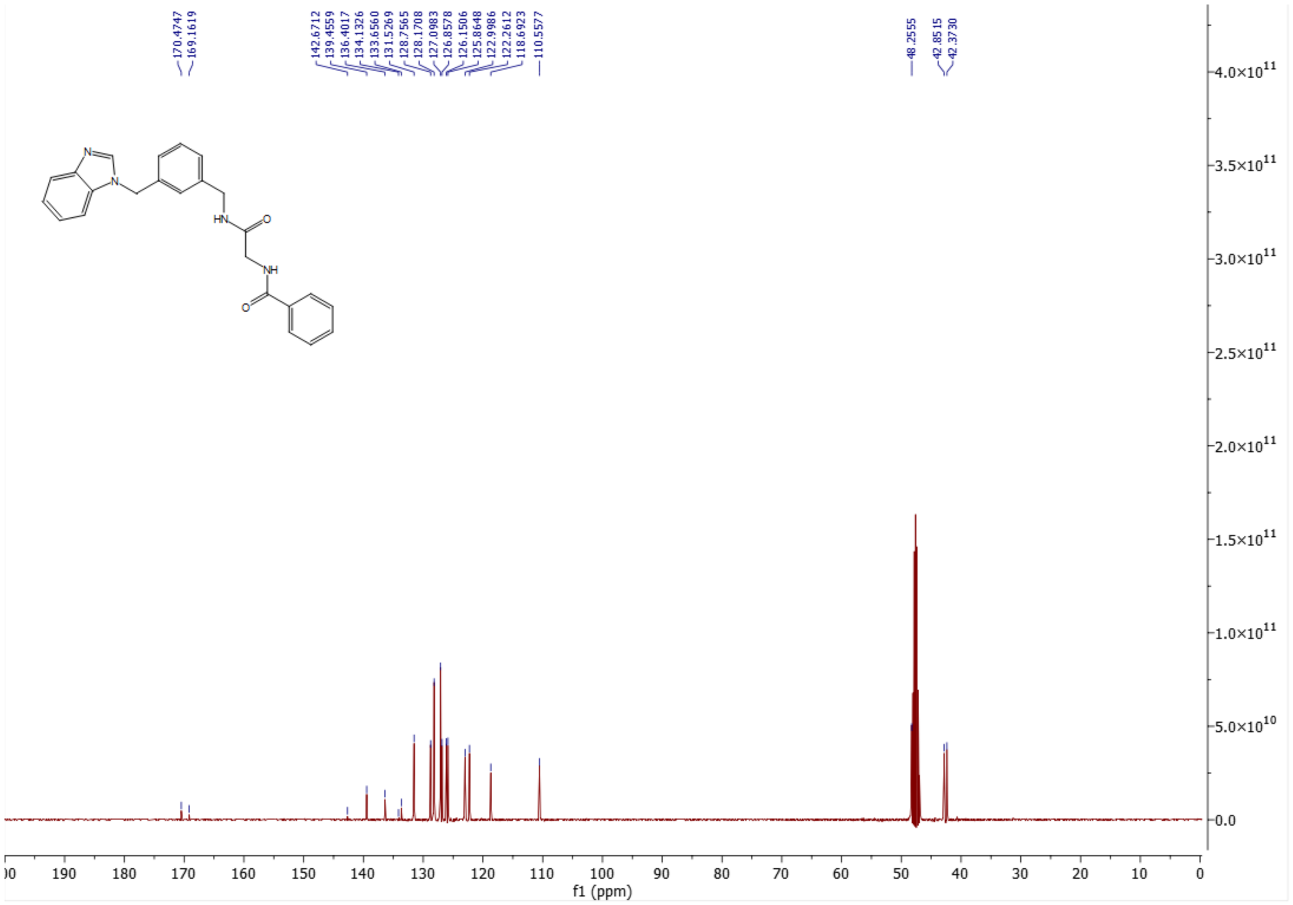

2a: ^{1}H (400 MHz, $CDCl_3$), ^{13}C (101 MHz, $CDCl_3$)

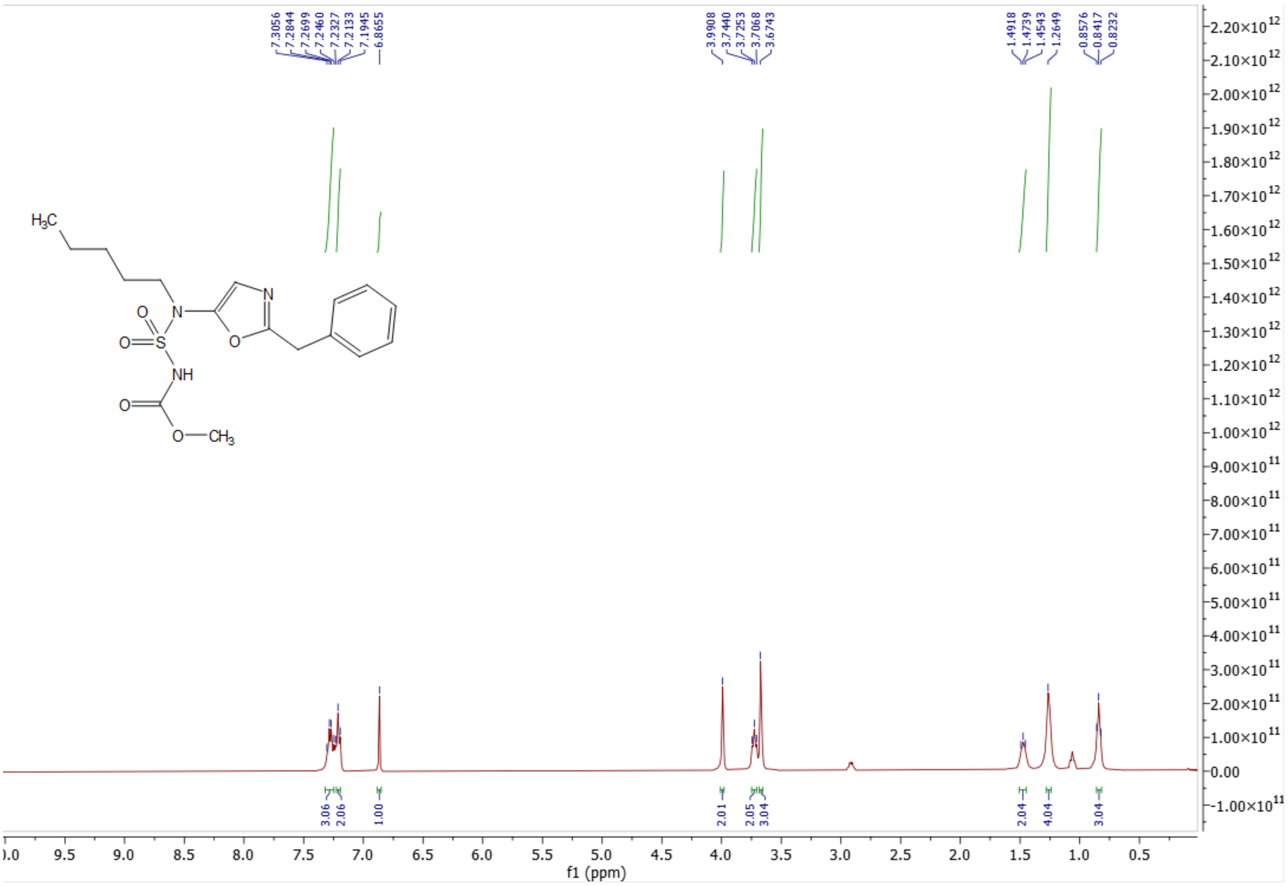

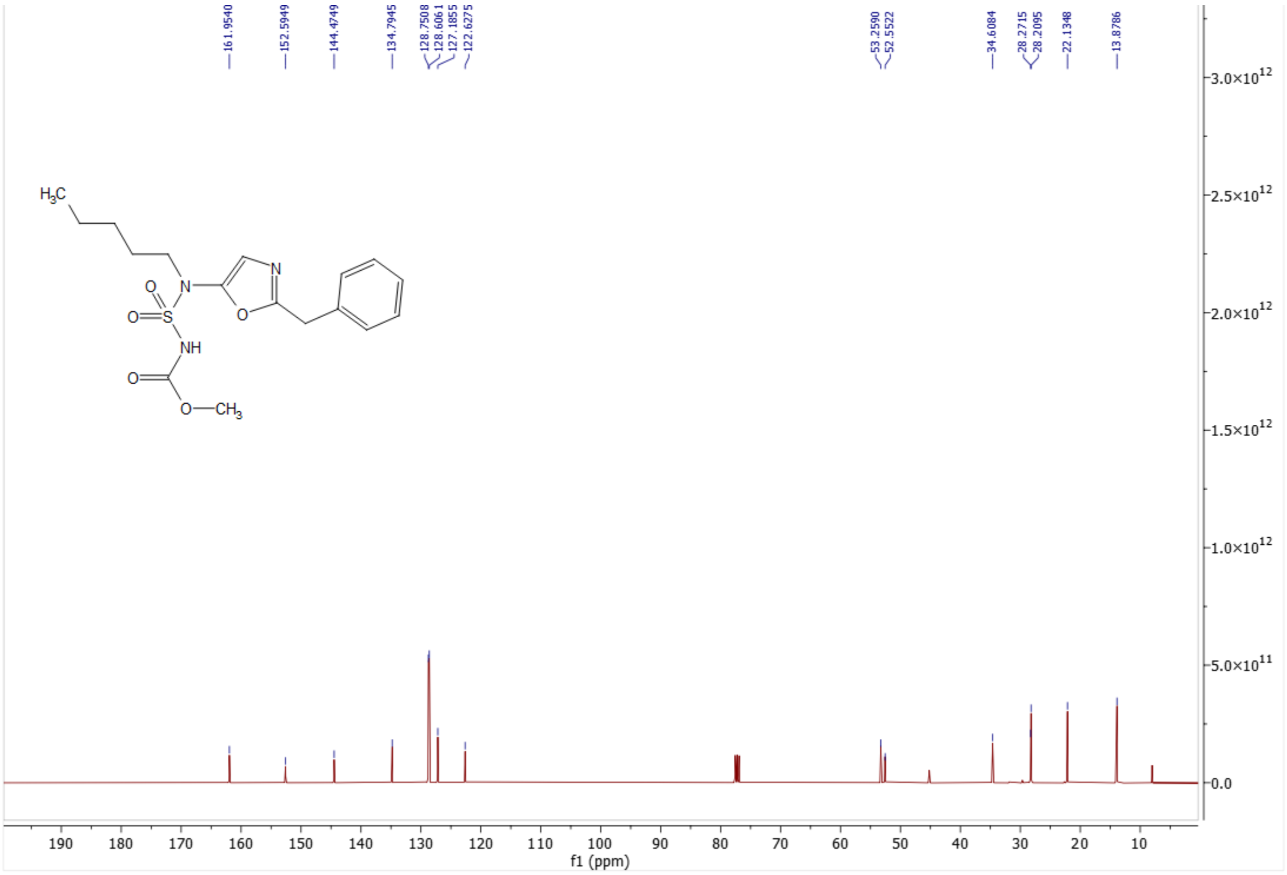

2b: ^{1}H (400 MHz, $CDCl_3$), ^{13}C (101 MHz, $CDCl_3$)

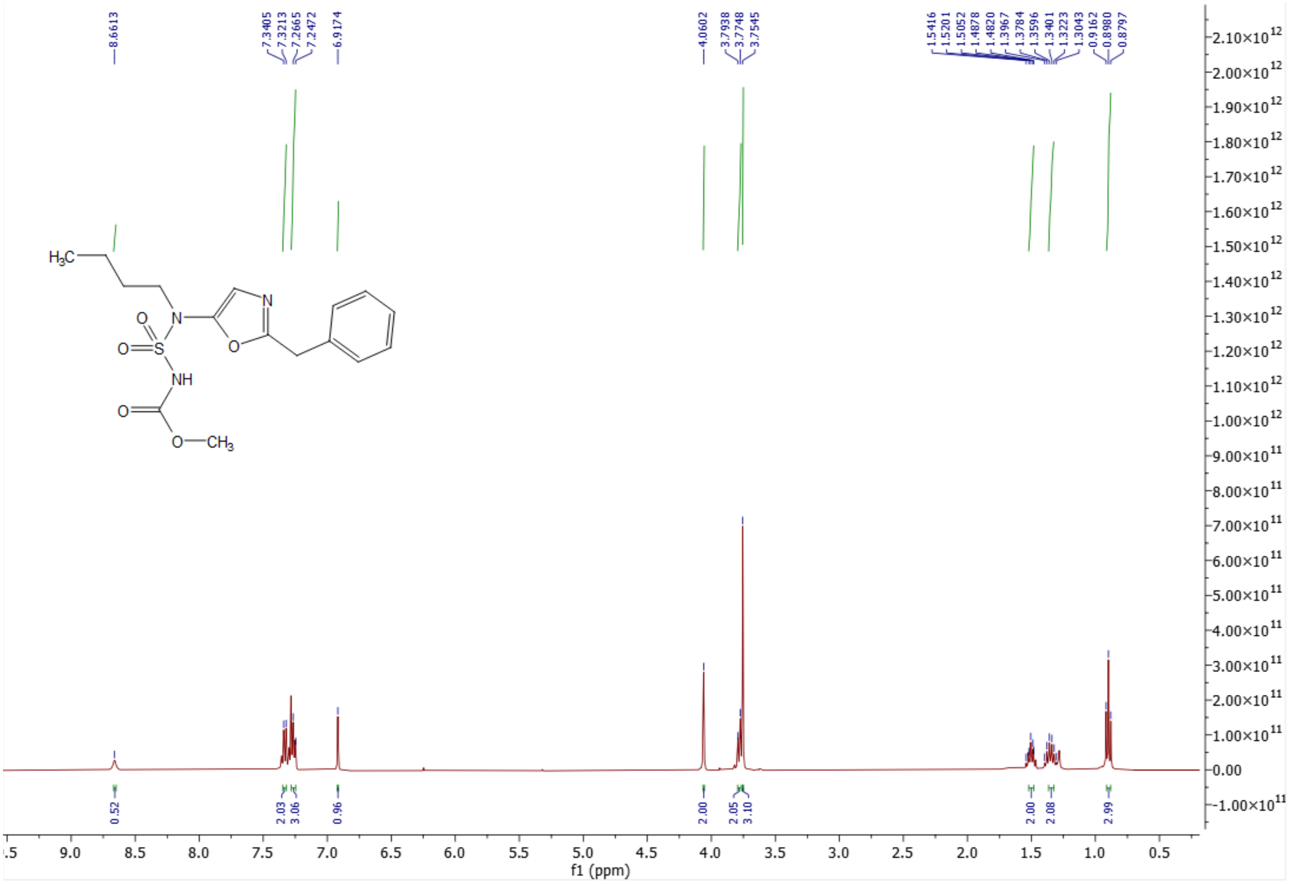

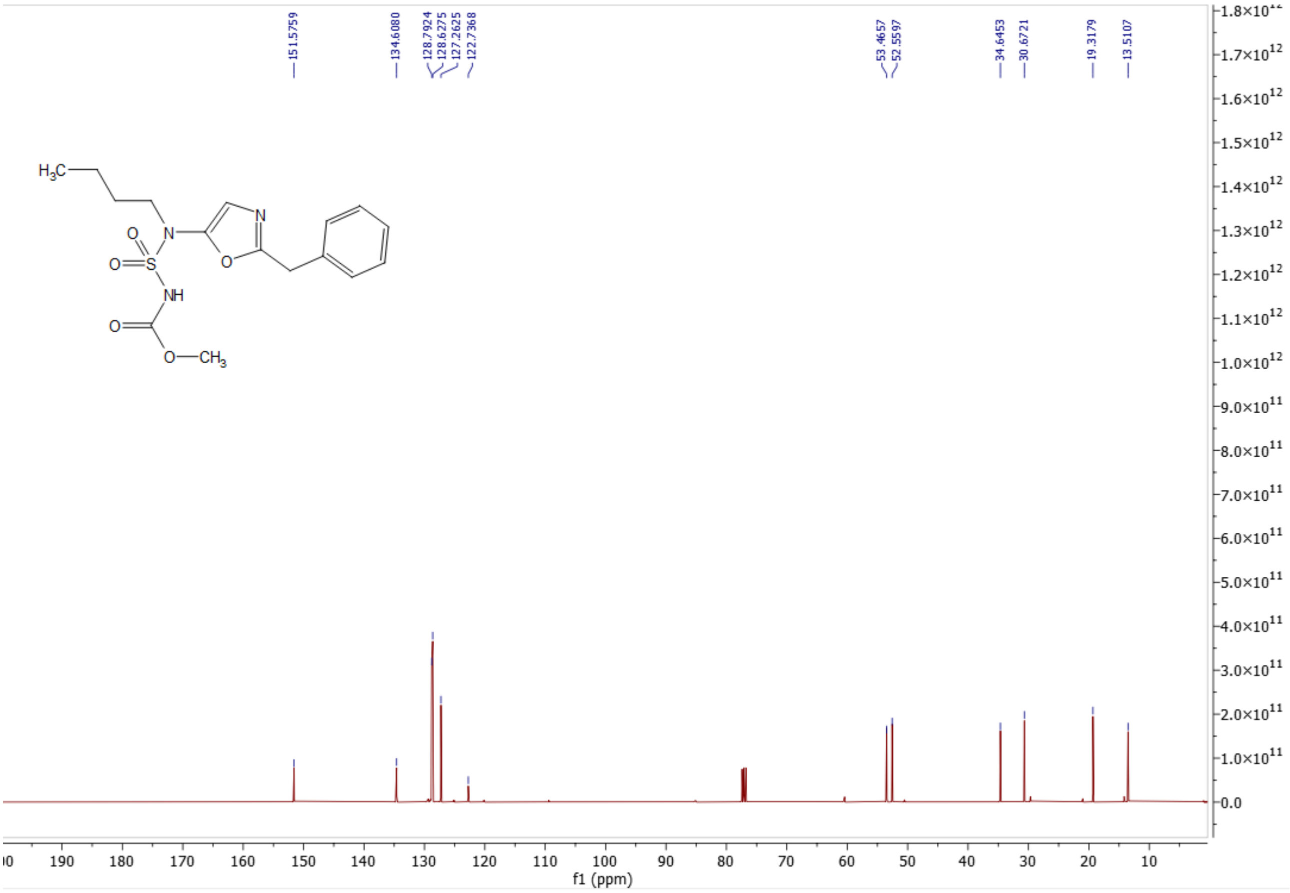

2c: ¹H (400 MHz, $CDCl_3$), ¹³C (101 MHz, $CDCl_3$)

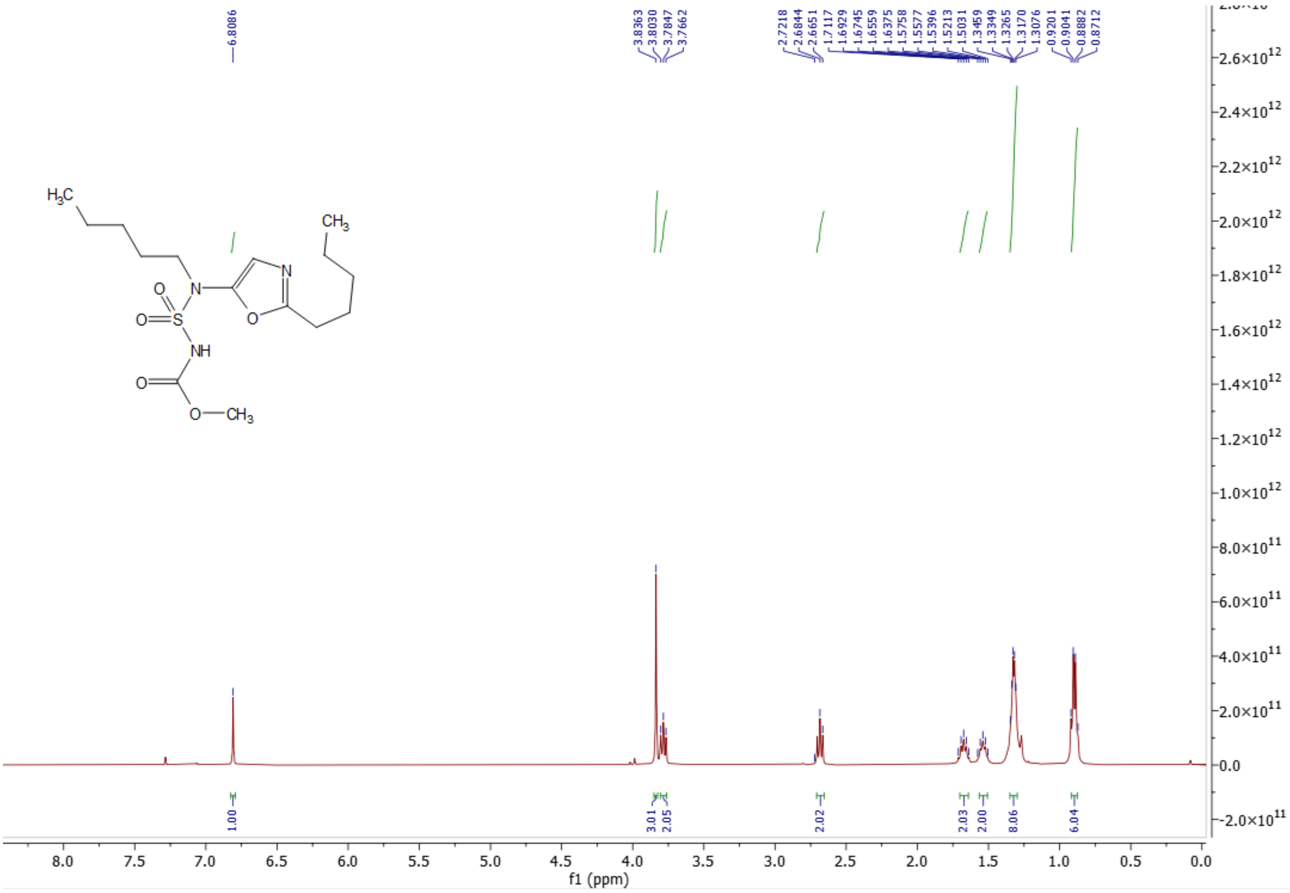

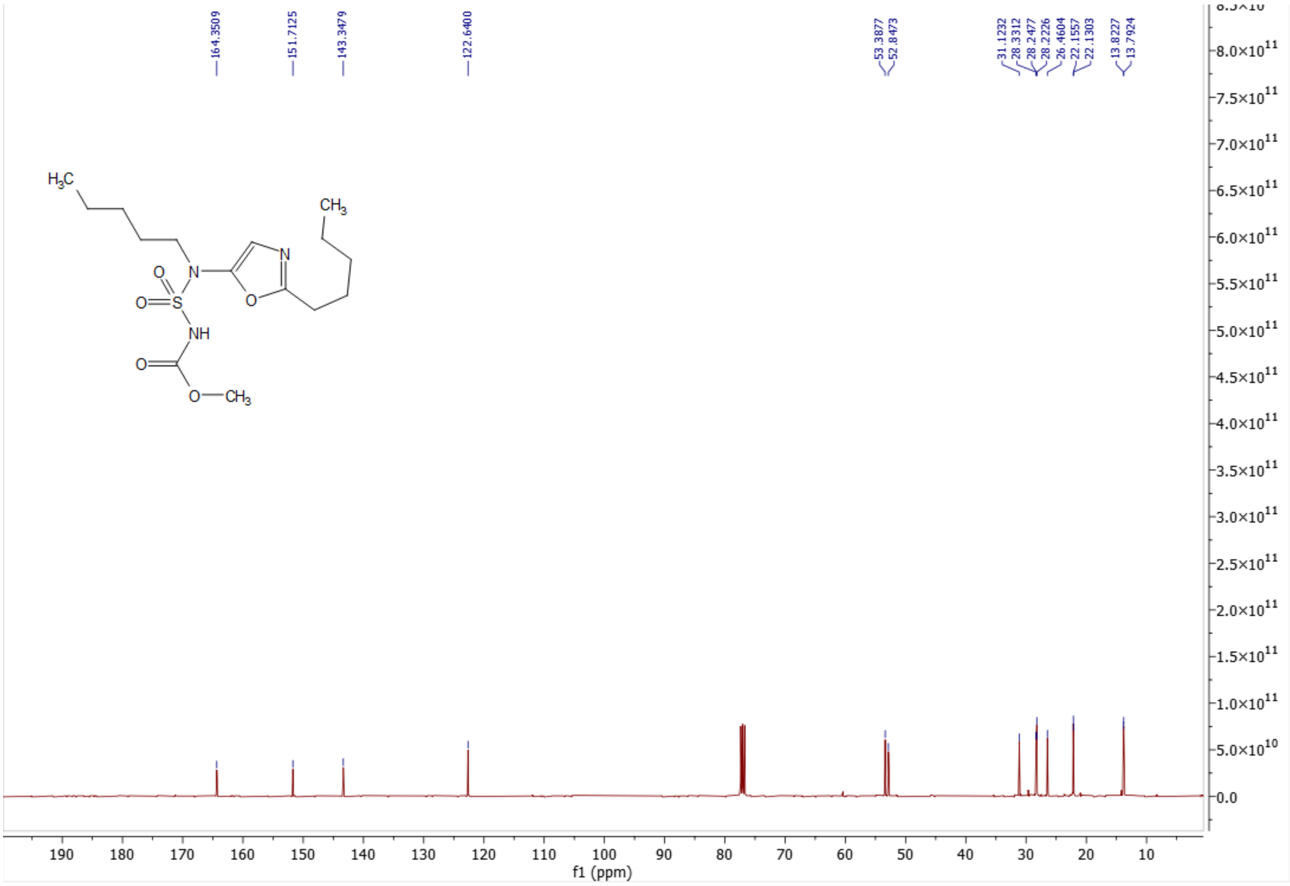

2d: ^{1}H (400 MHz, CD_3OD), ^{13}C (101 MHz, CD_3OD)

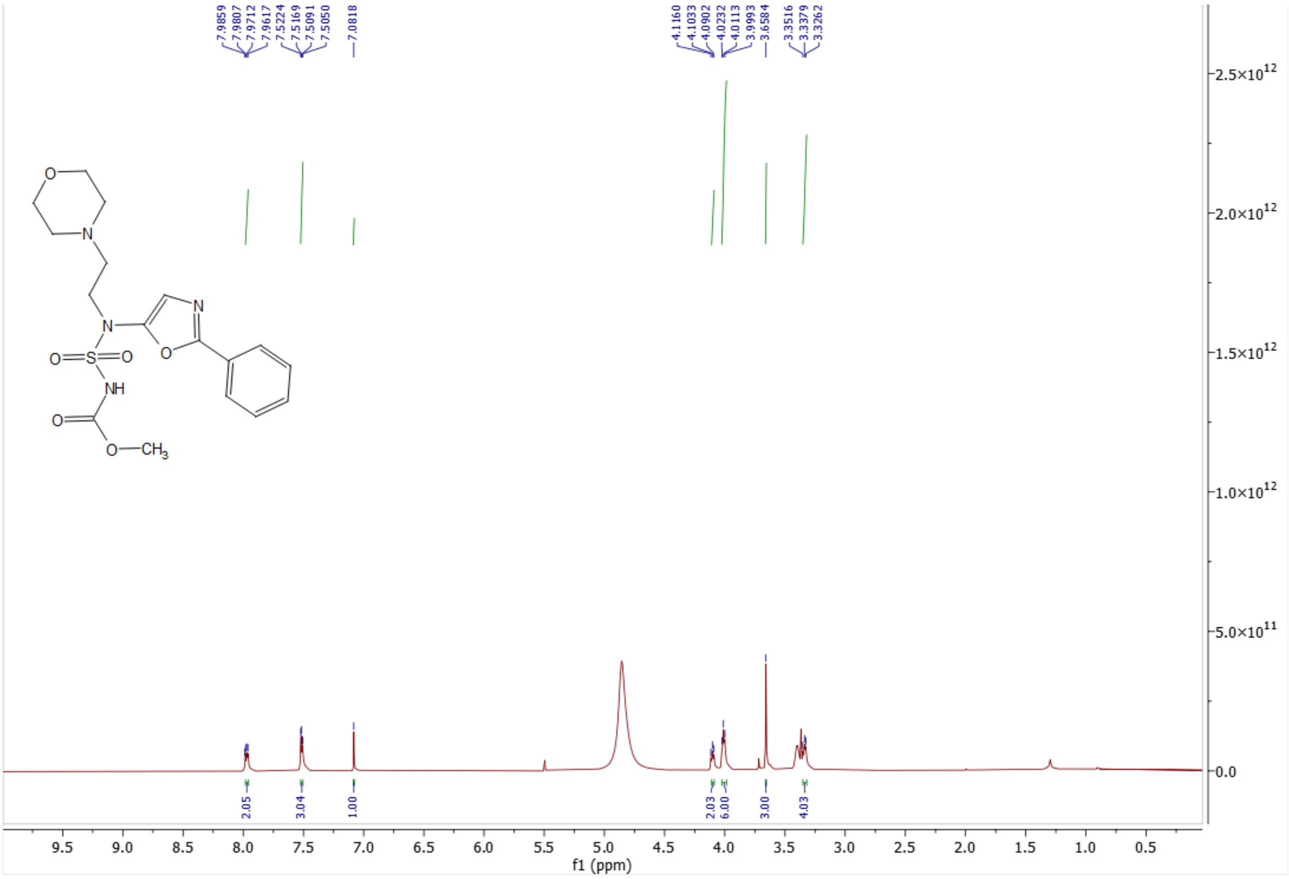

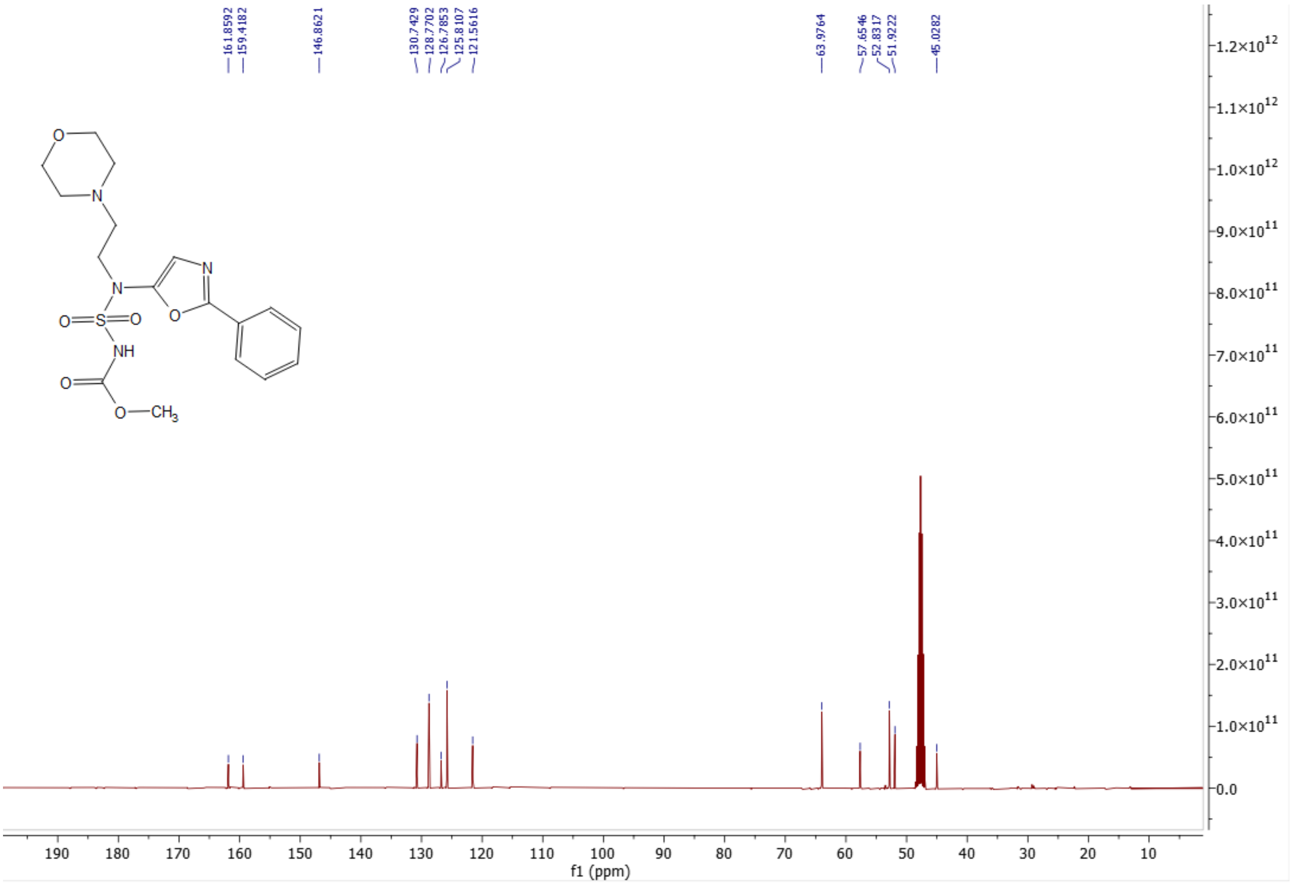

2e: ^{1}H (400 MHz, $CDCl_3$), ^{13}C (101 MHz, $CDCl_3$)

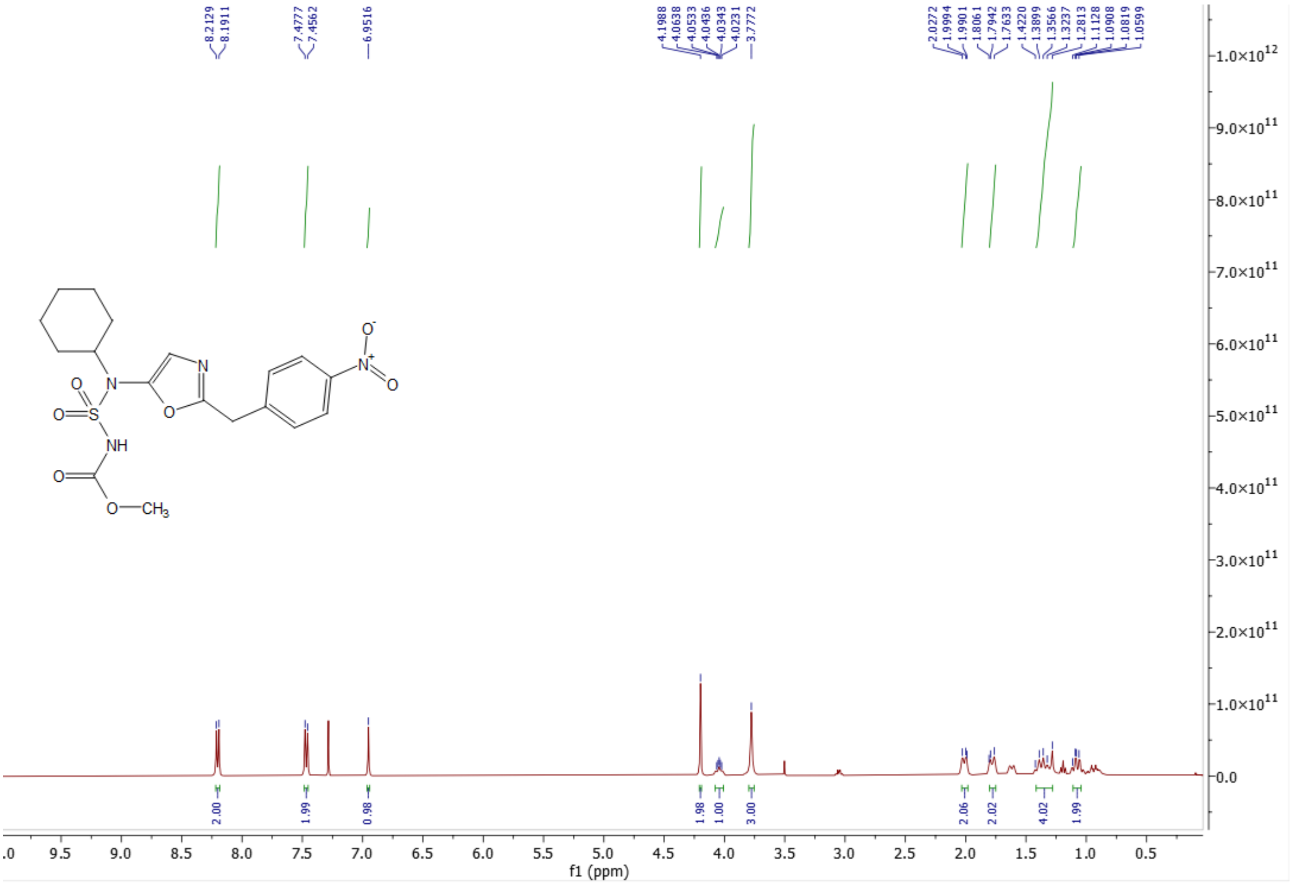

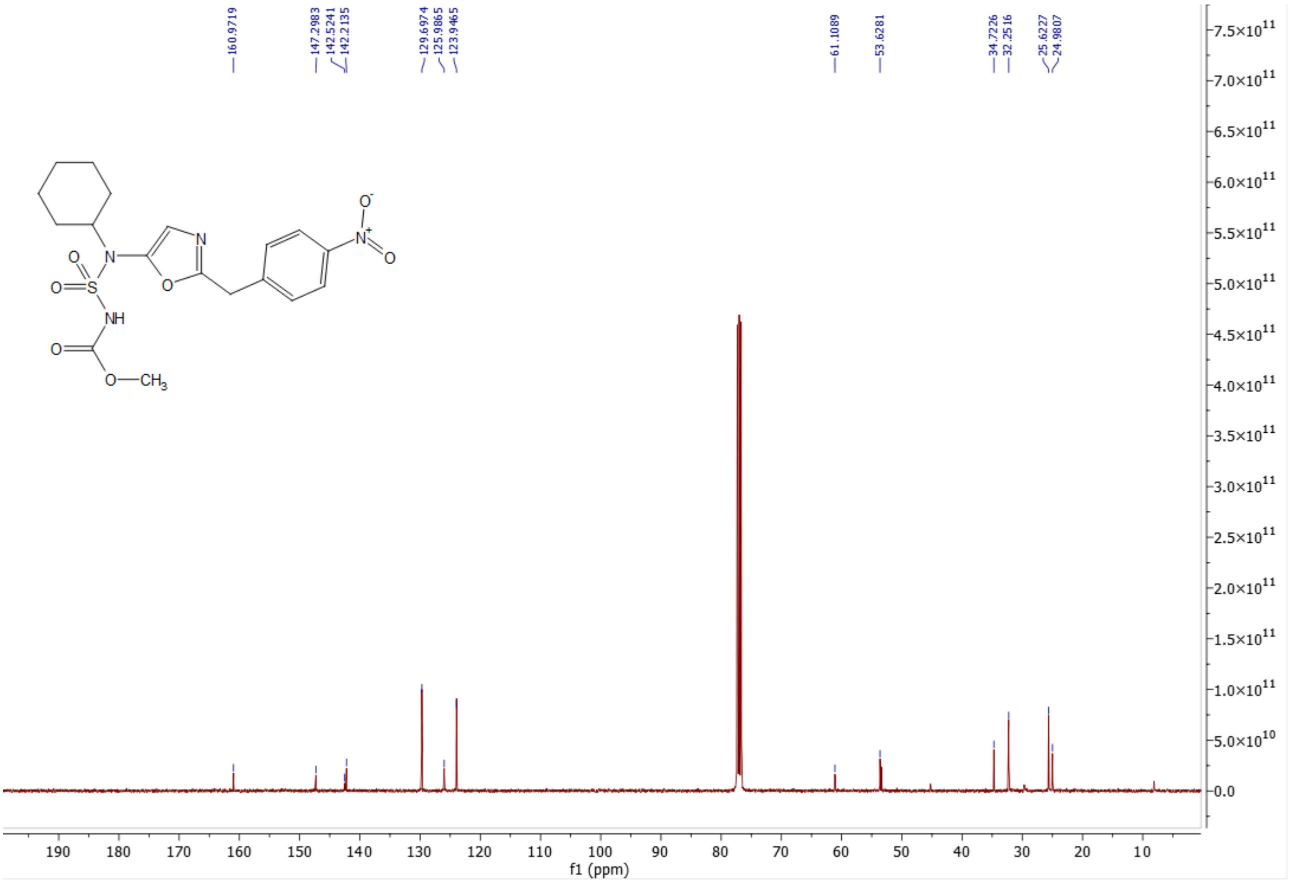

2f: ^{1}H (400 MHz, $CDCl_3$), ^{13}C (101 MHz, $CDCl_3$)

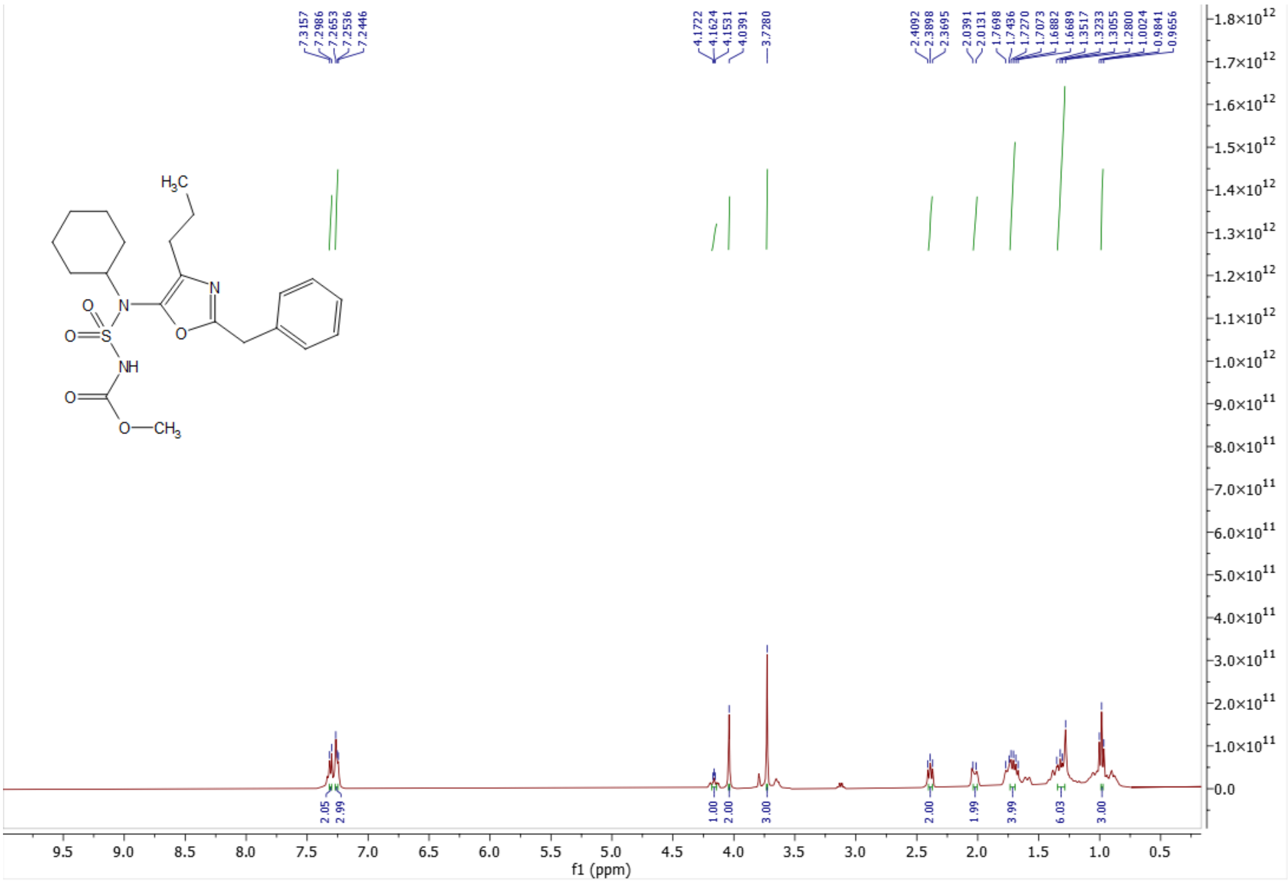

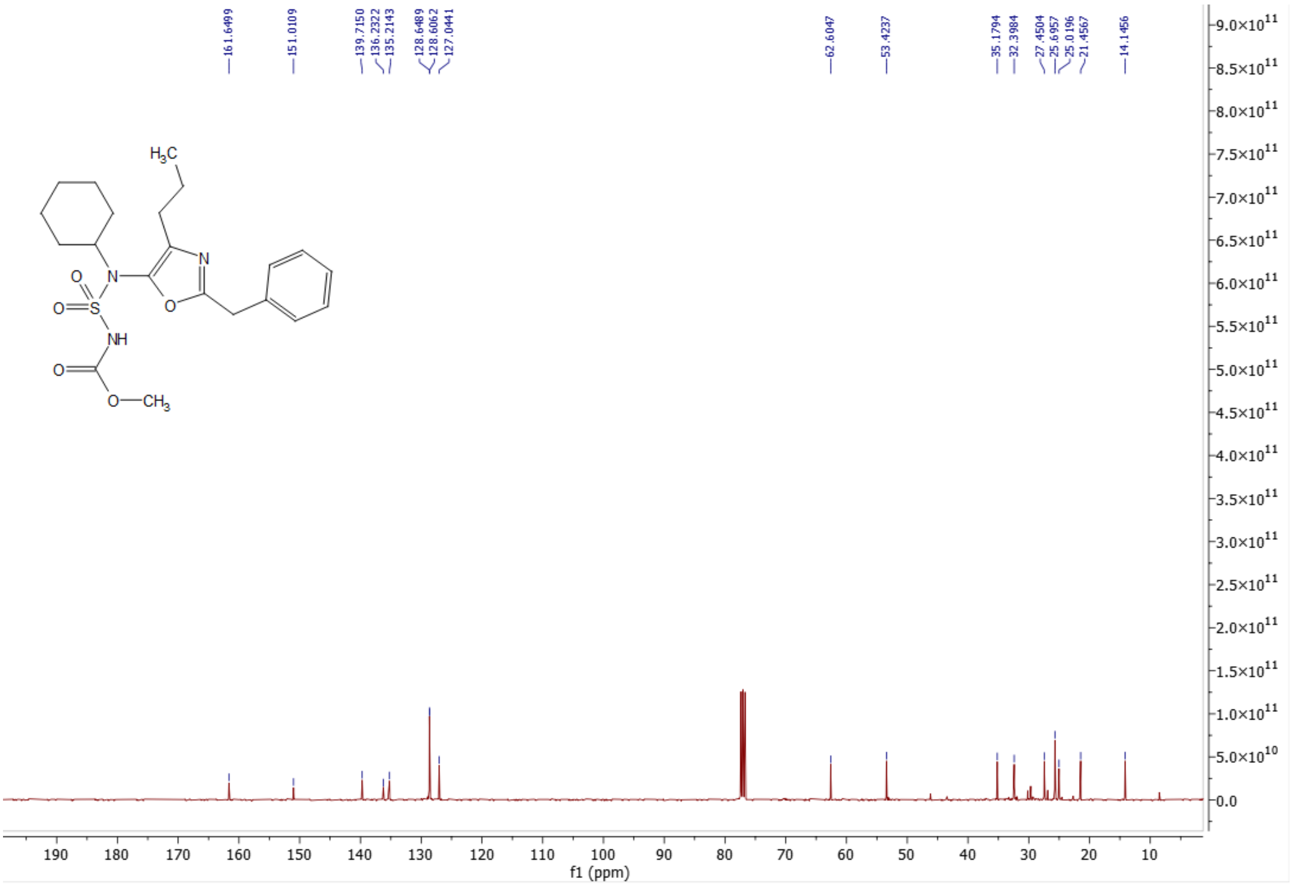

2g: ^{1}H (400 MHz, $CDCl_3$), ^{13}C (101 MHz, $CDCl_3$)

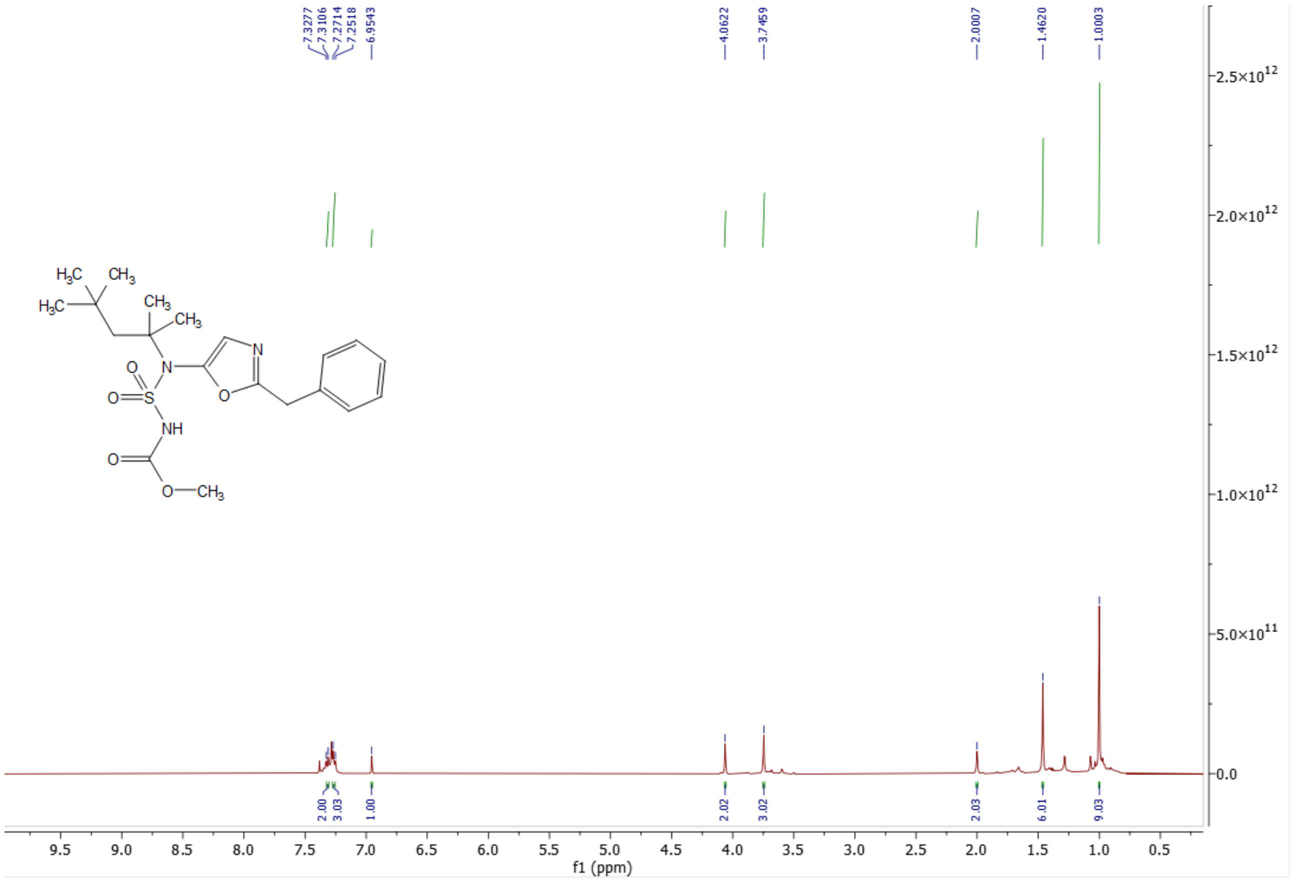

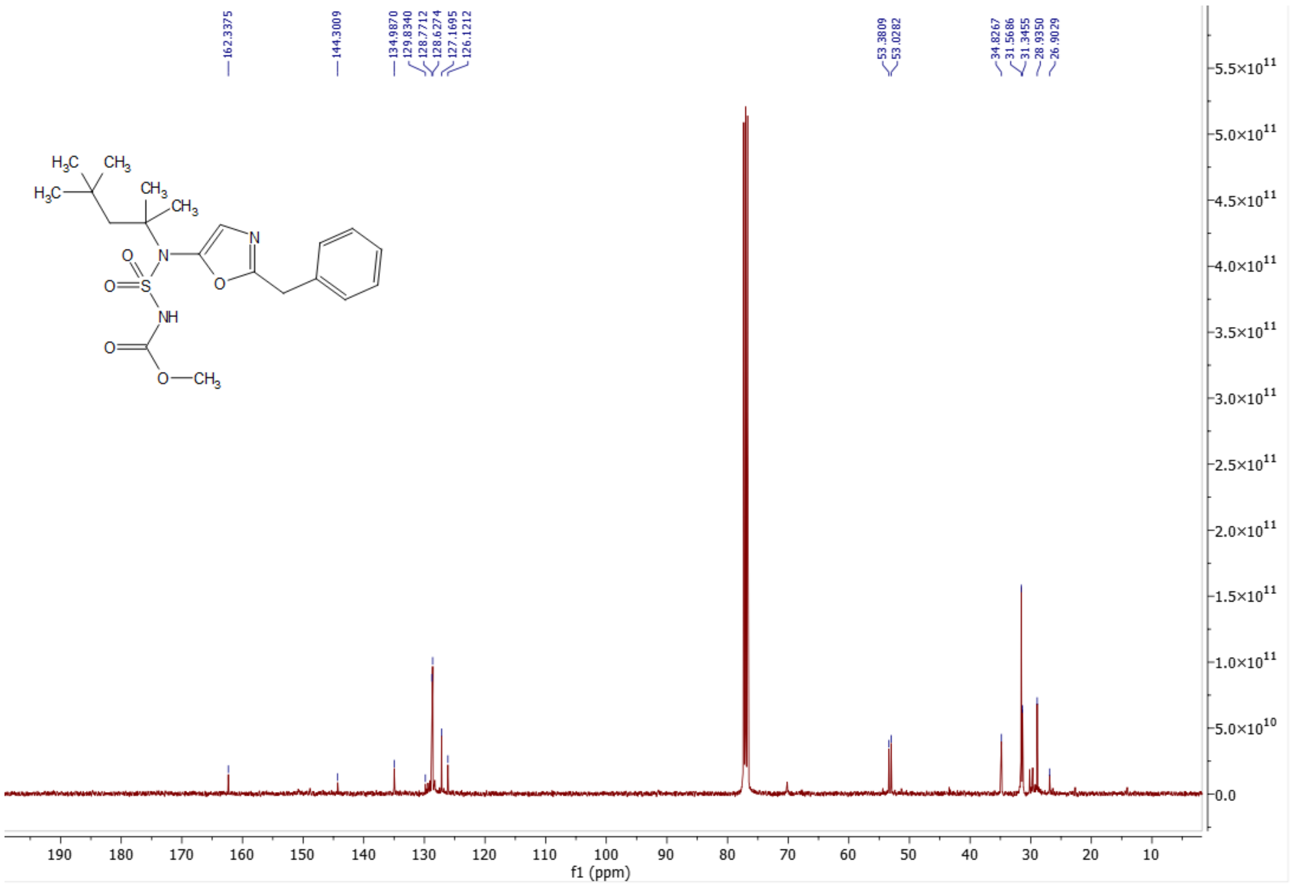

2h: ^{1}H (400 MHz, $CDCl_3$), ^{13}C (101 MHz, $CDCl_3$)

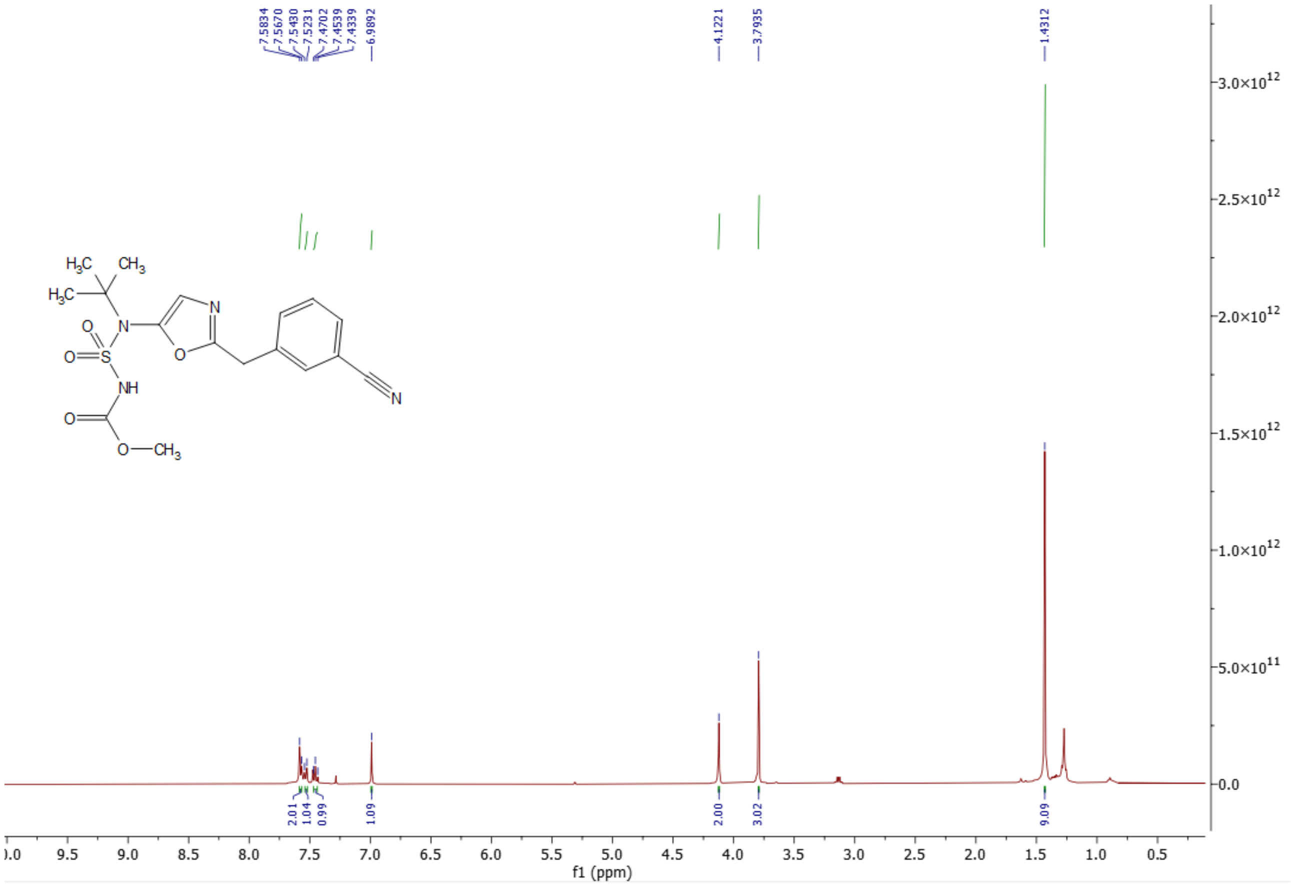

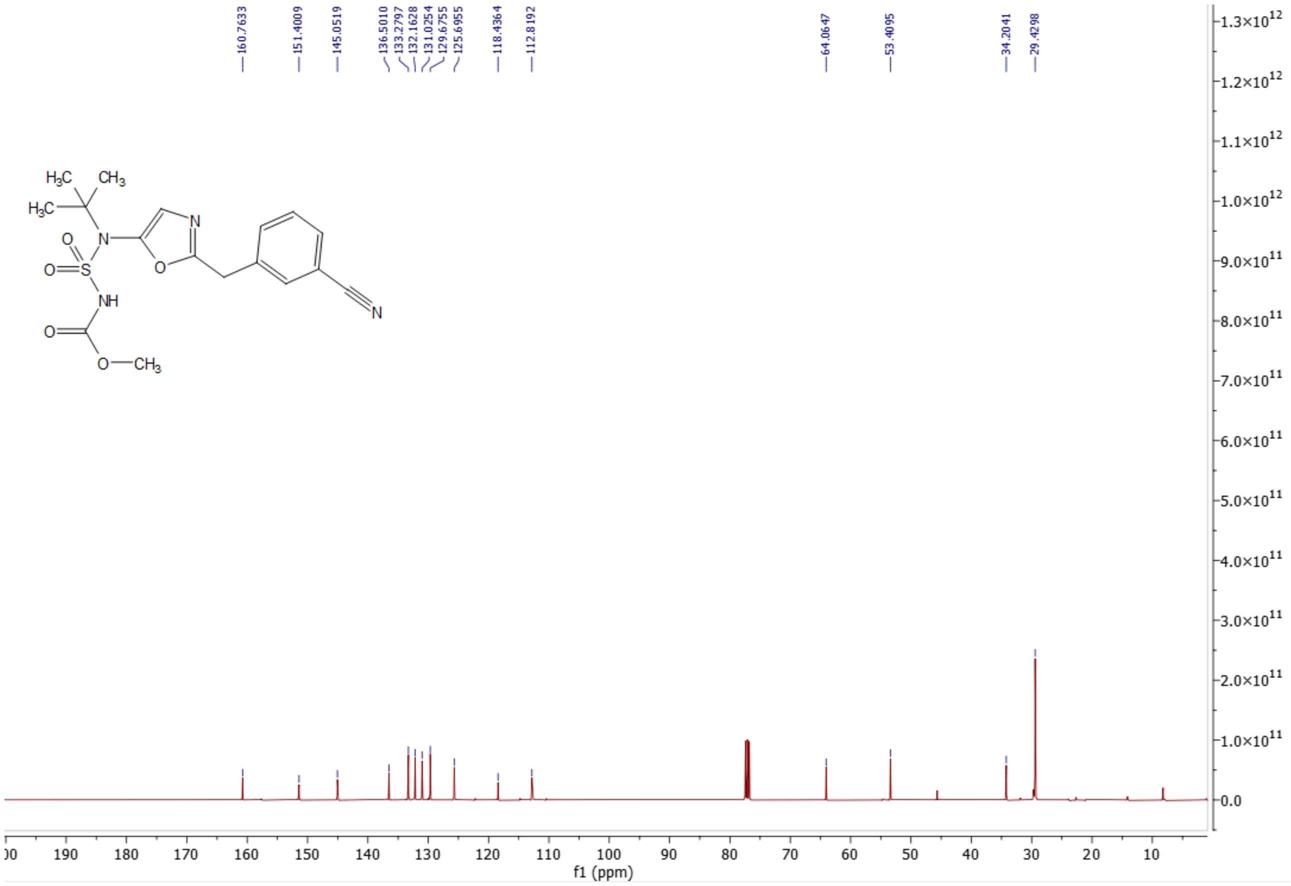

2i: ^{1}H (400 MHz, $CDCl_3$), ^{13}C (101 MHz, $CDCl_3$)

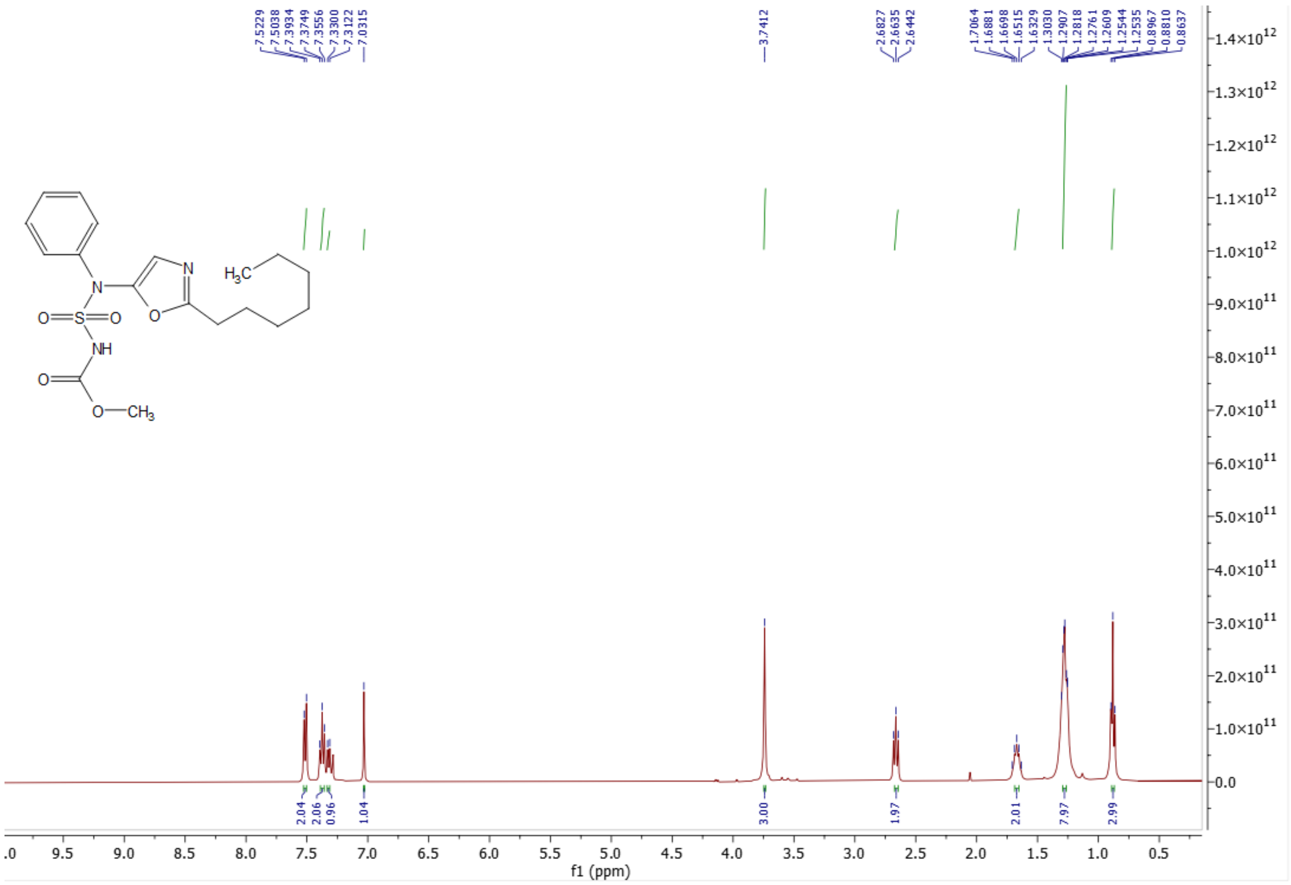

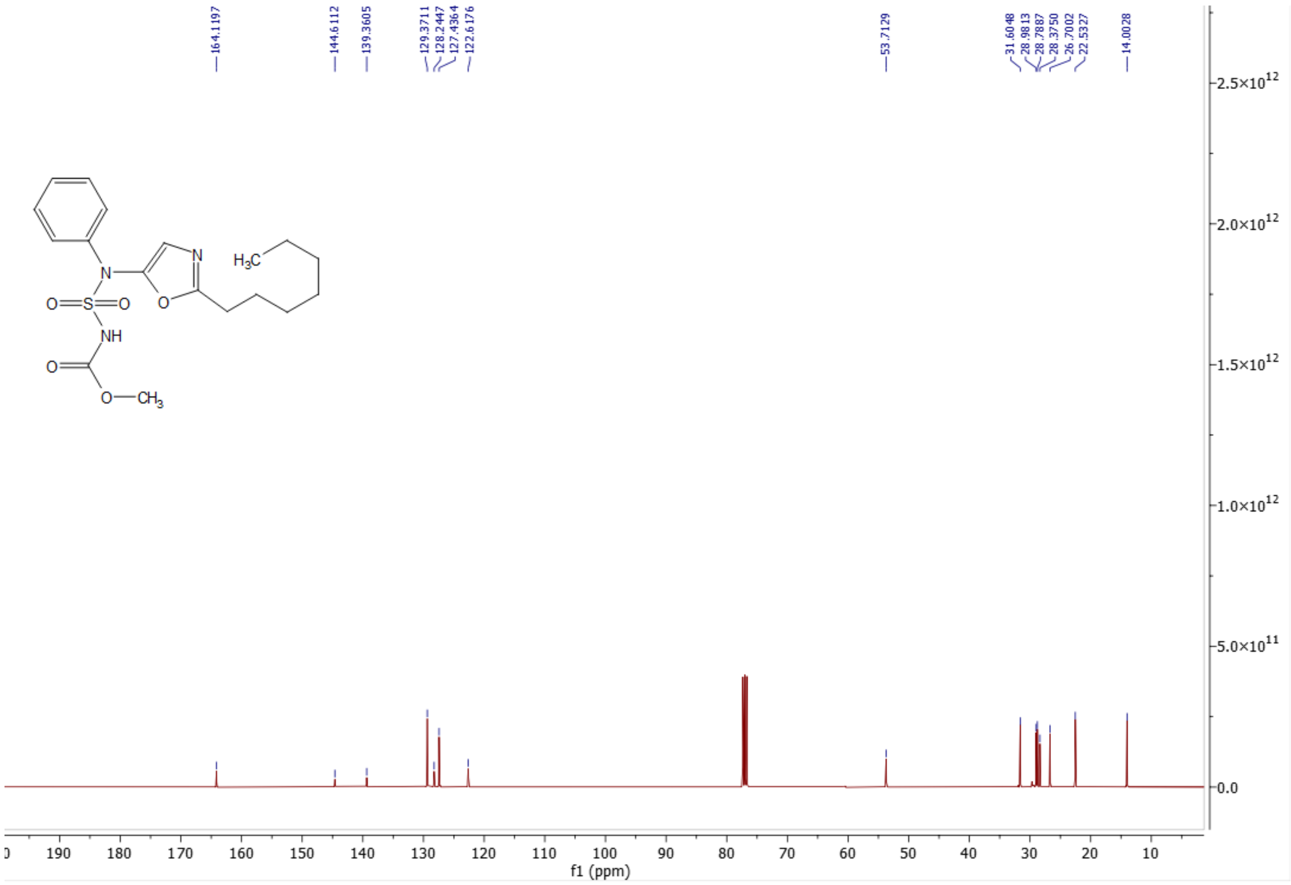

2j: ^{1}H (400 MHz, $CDCl_3$), ^{13}C (101 MHz, $CDCl_3$)

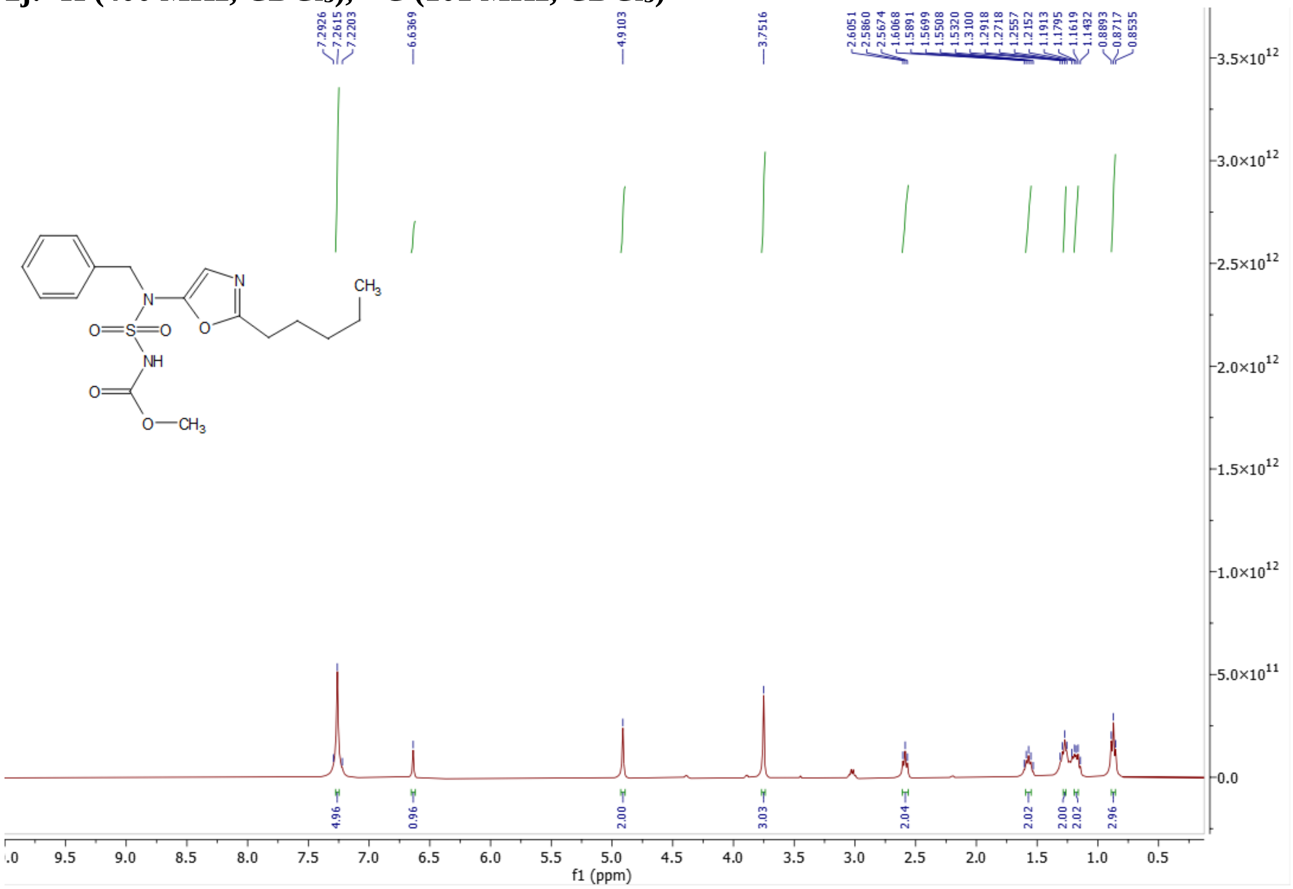

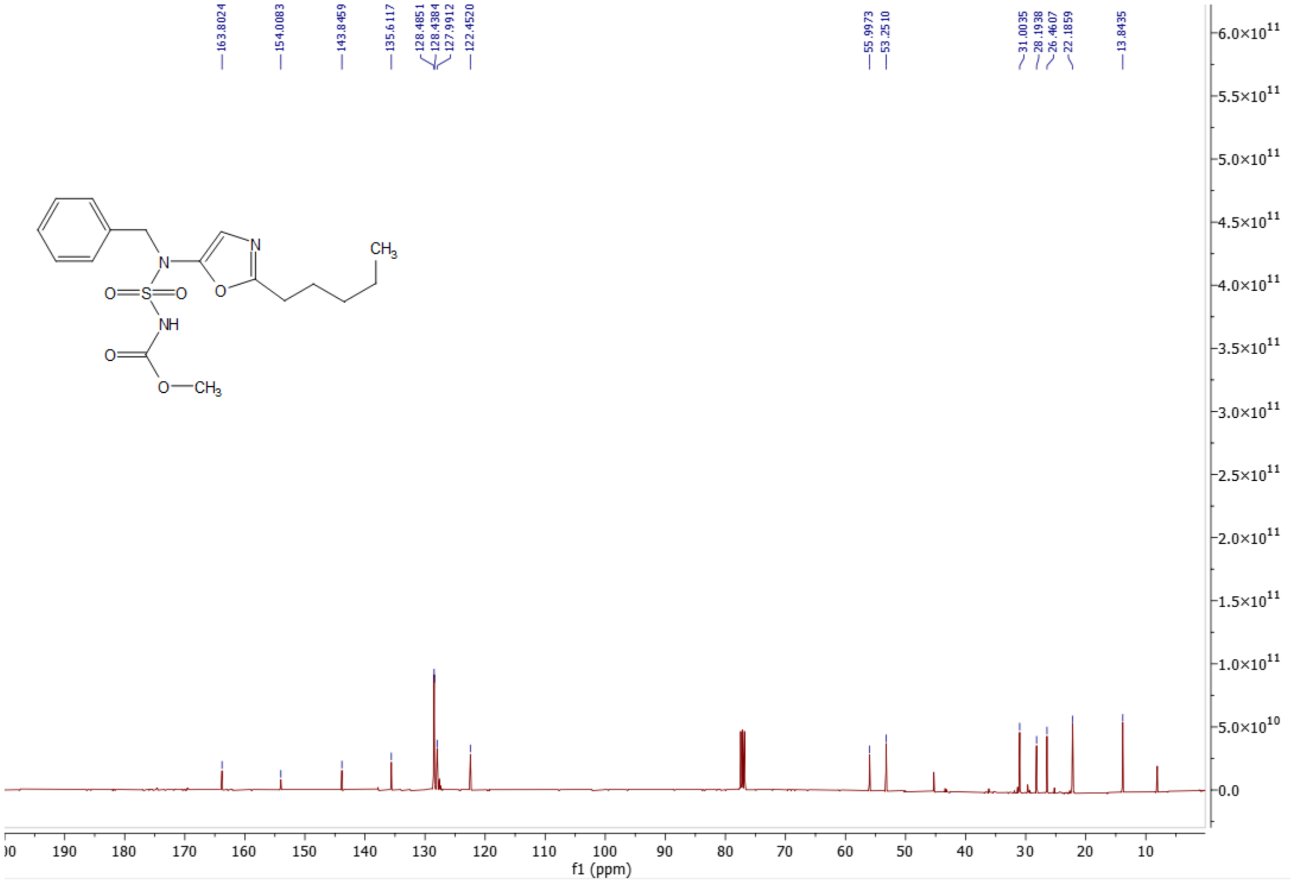

2k: ^{1}H (400 MHz, $CDCl_3$), ^{13}C (101 MHz, $CDCl_3$)

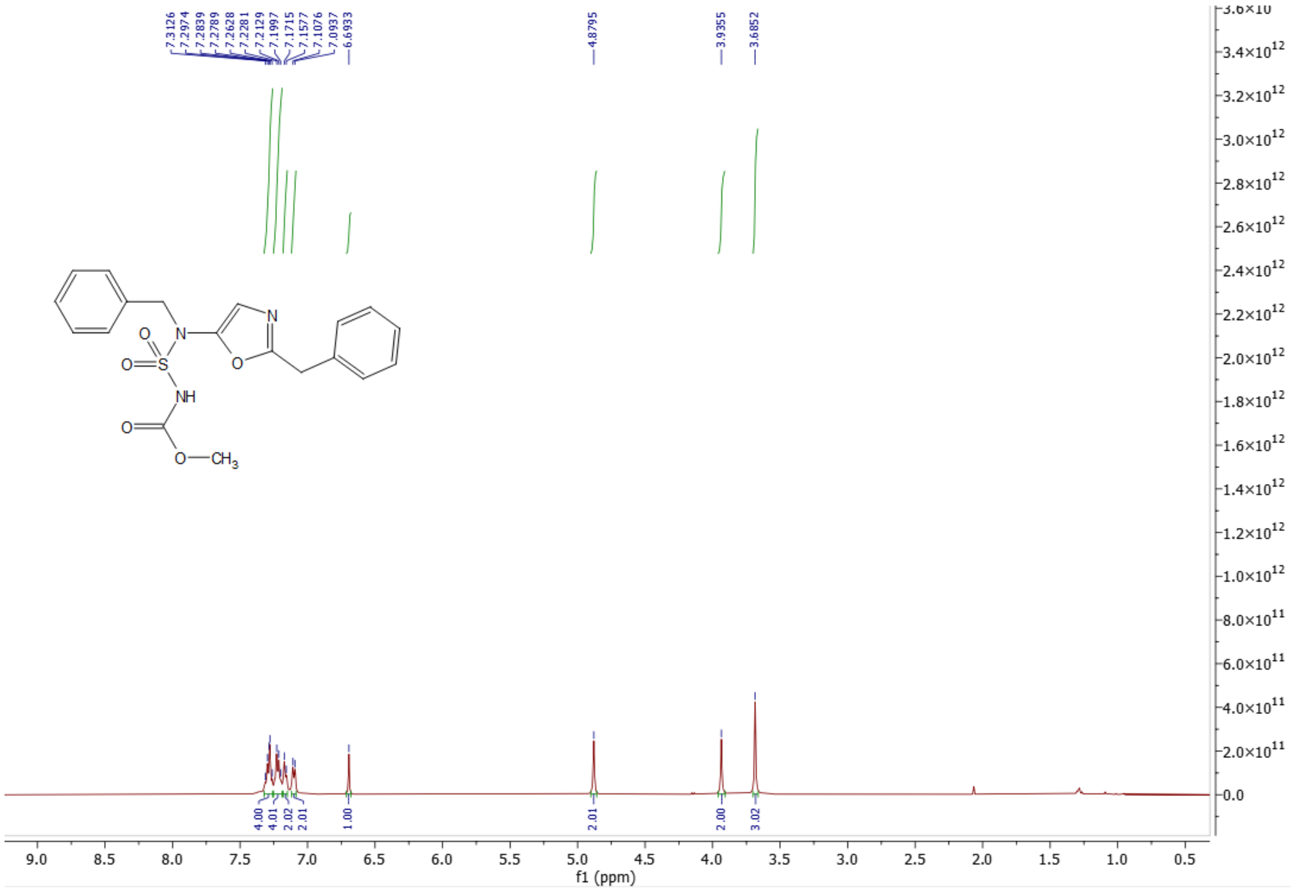

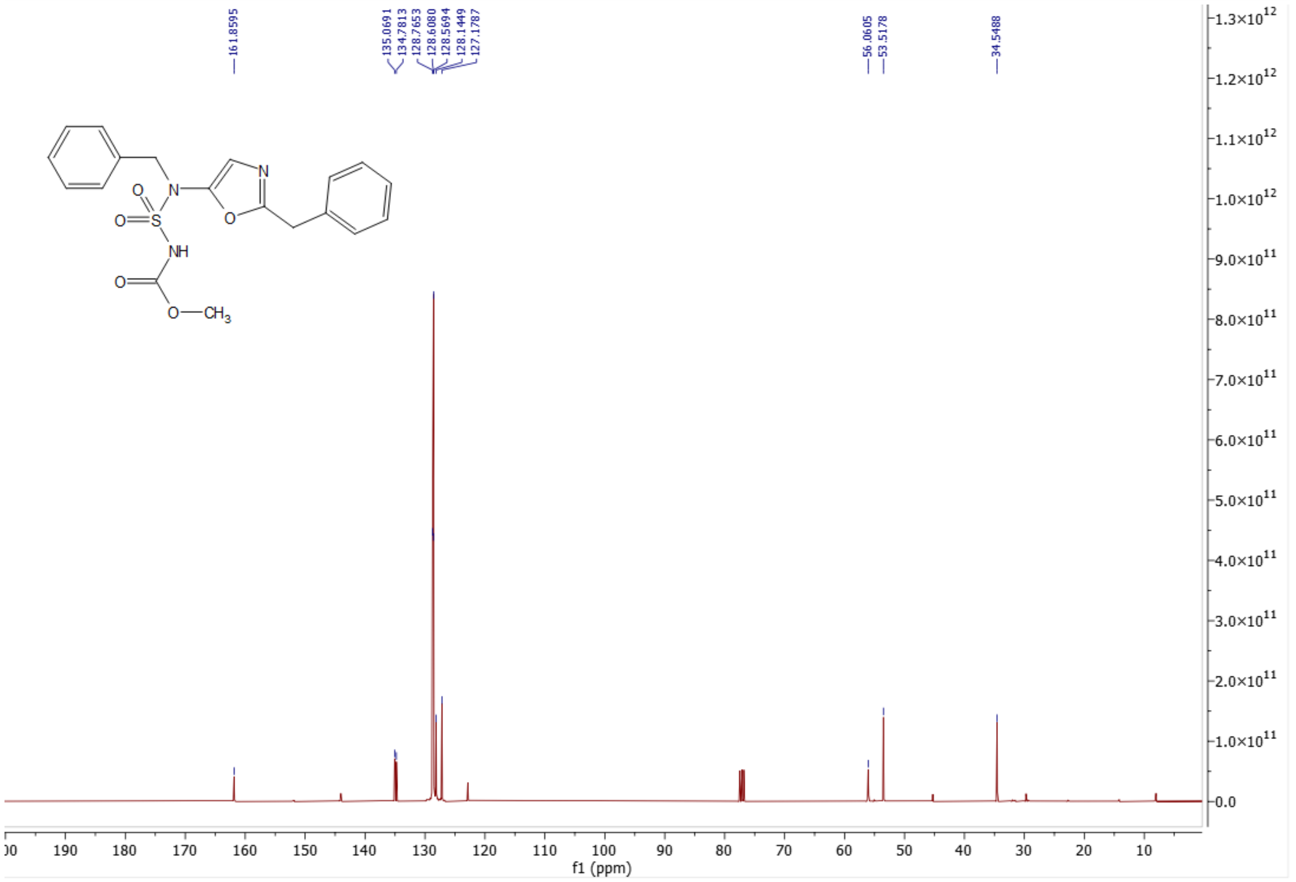

2l: ^{1}H (400 MHz, $CDCl_3$), ^{13}C (101 MHz, $CDCl_3$)

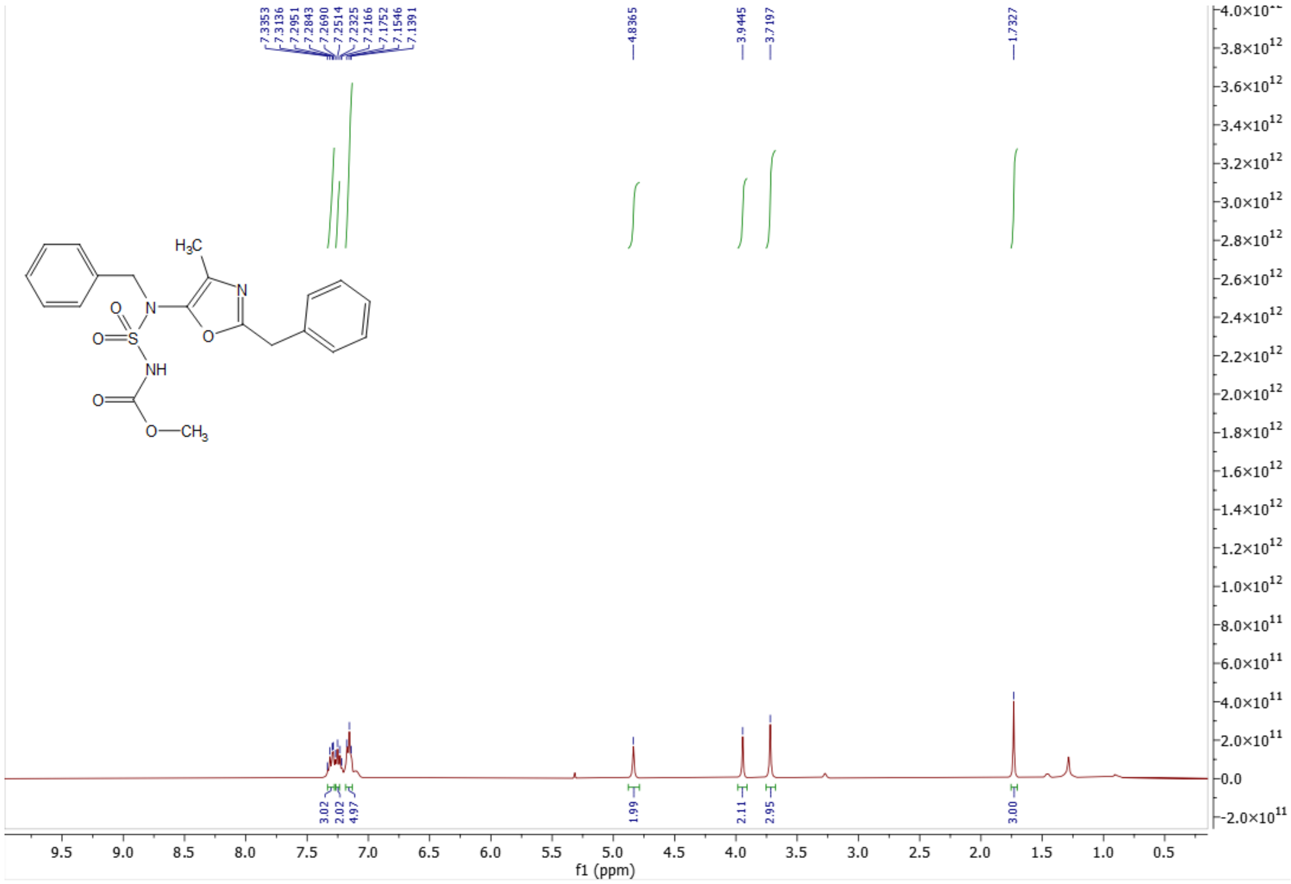

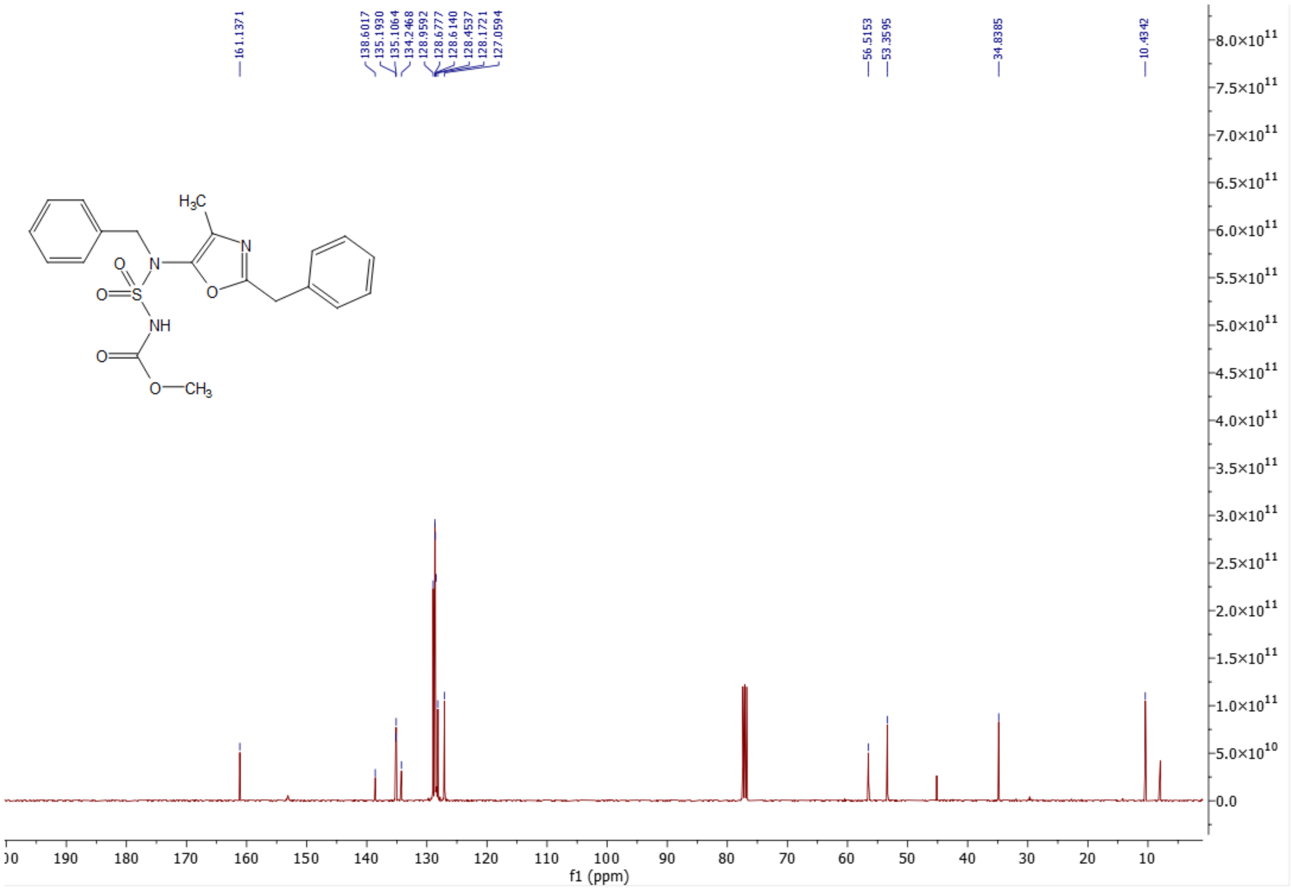

2m: ^{1}H (400 MHz, $CDCl_3$), ^{13}C (101 MHz, $CDCl_3$)

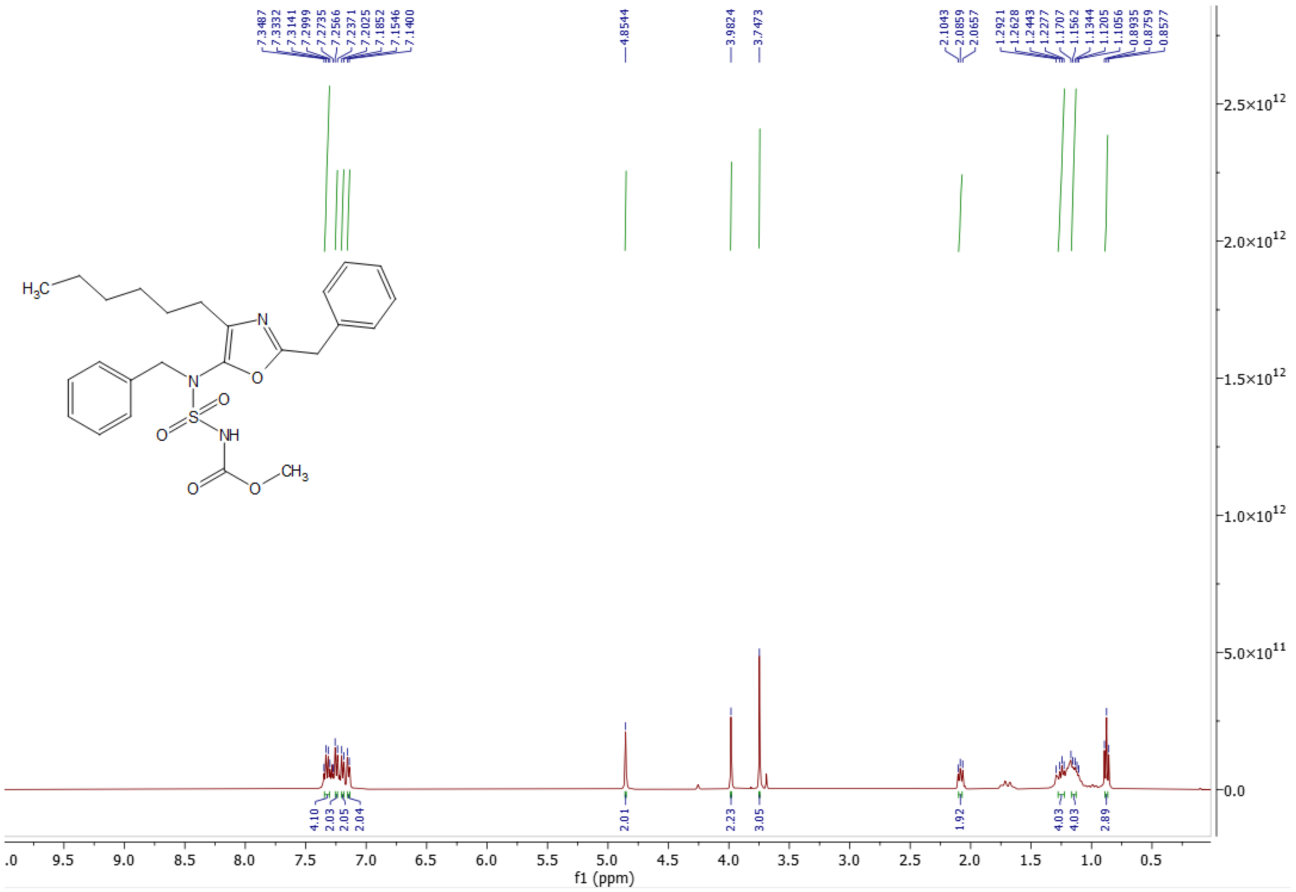

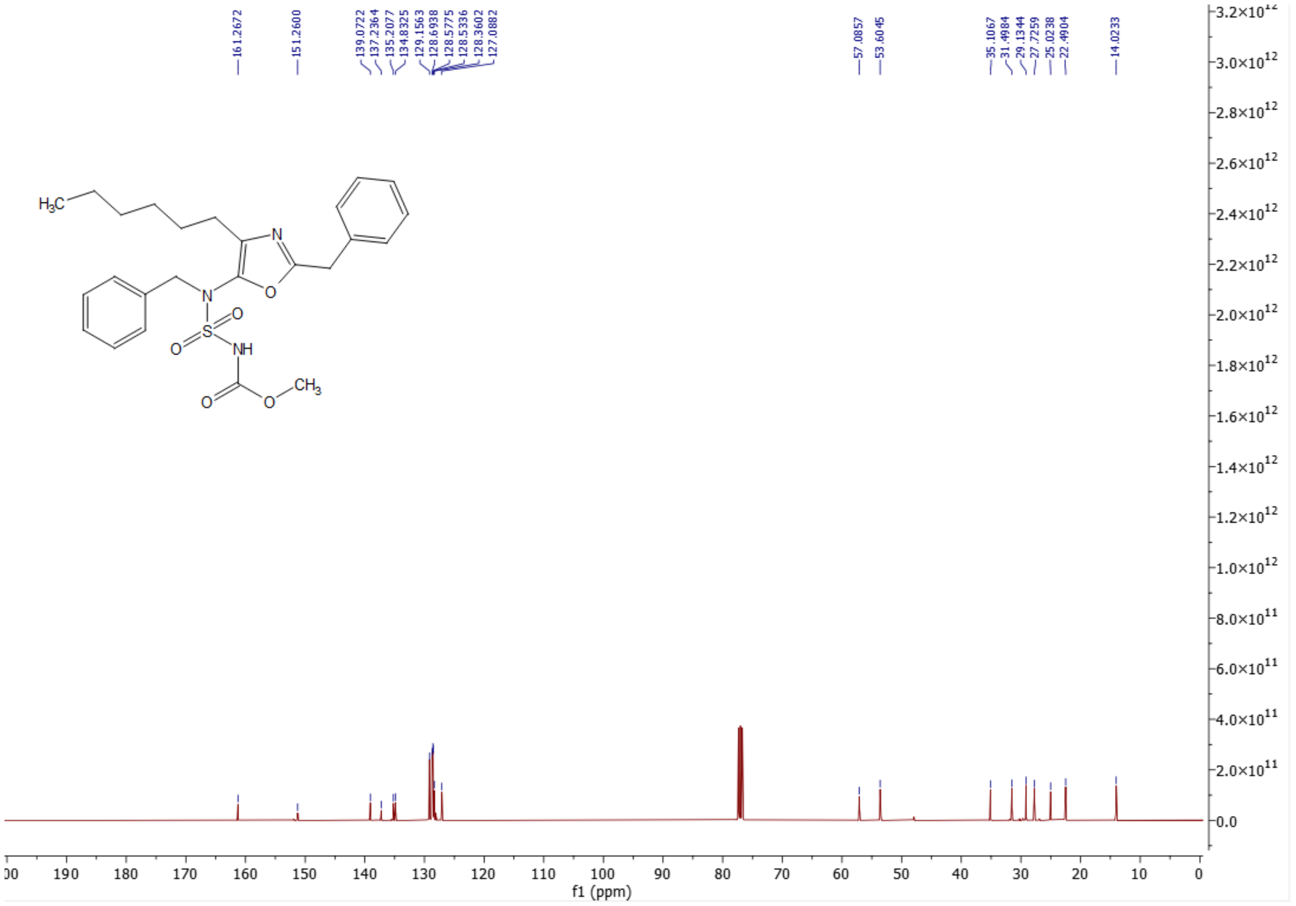

2n: ^{1}H (400 MHz, CD_3OD), ^{13}C (101 MHz, $CDCl_3$)

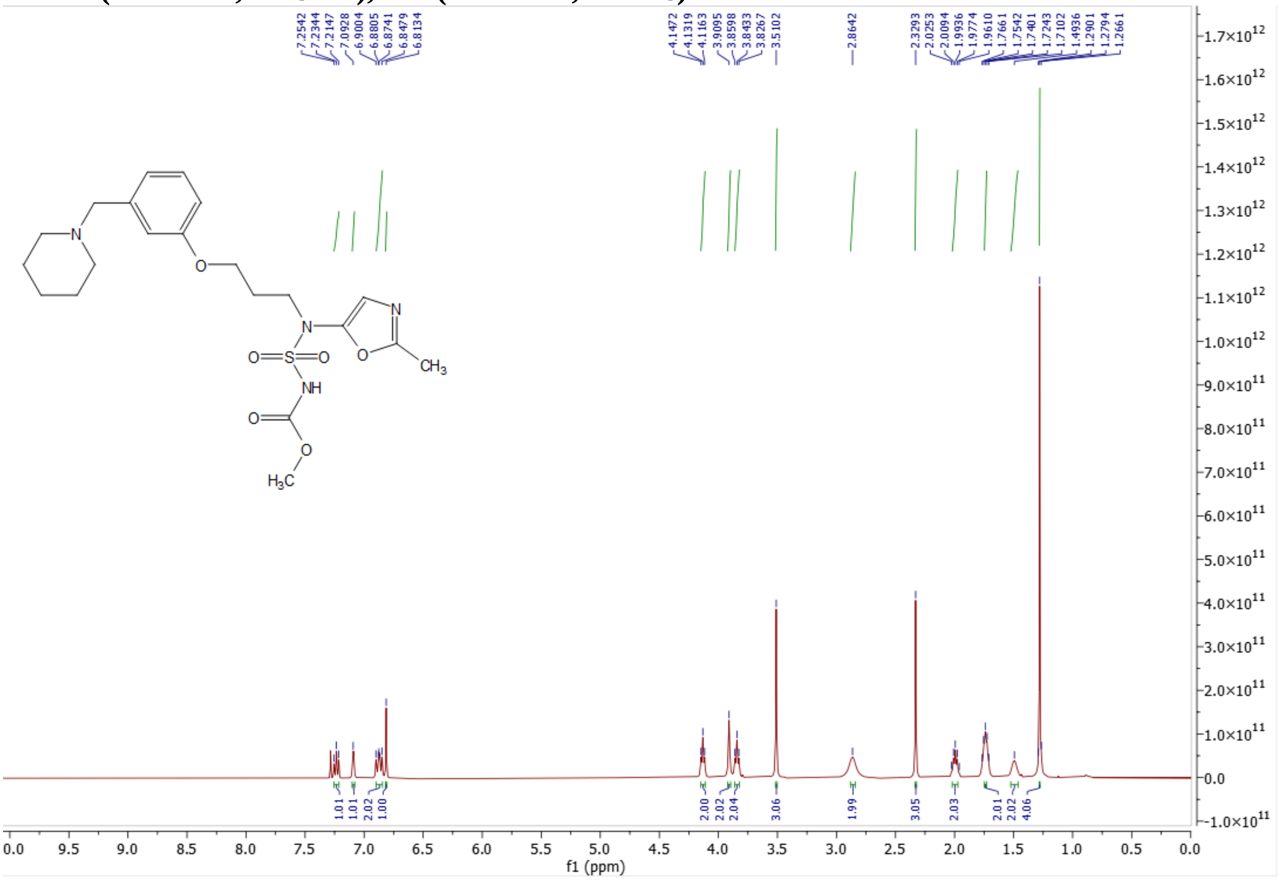

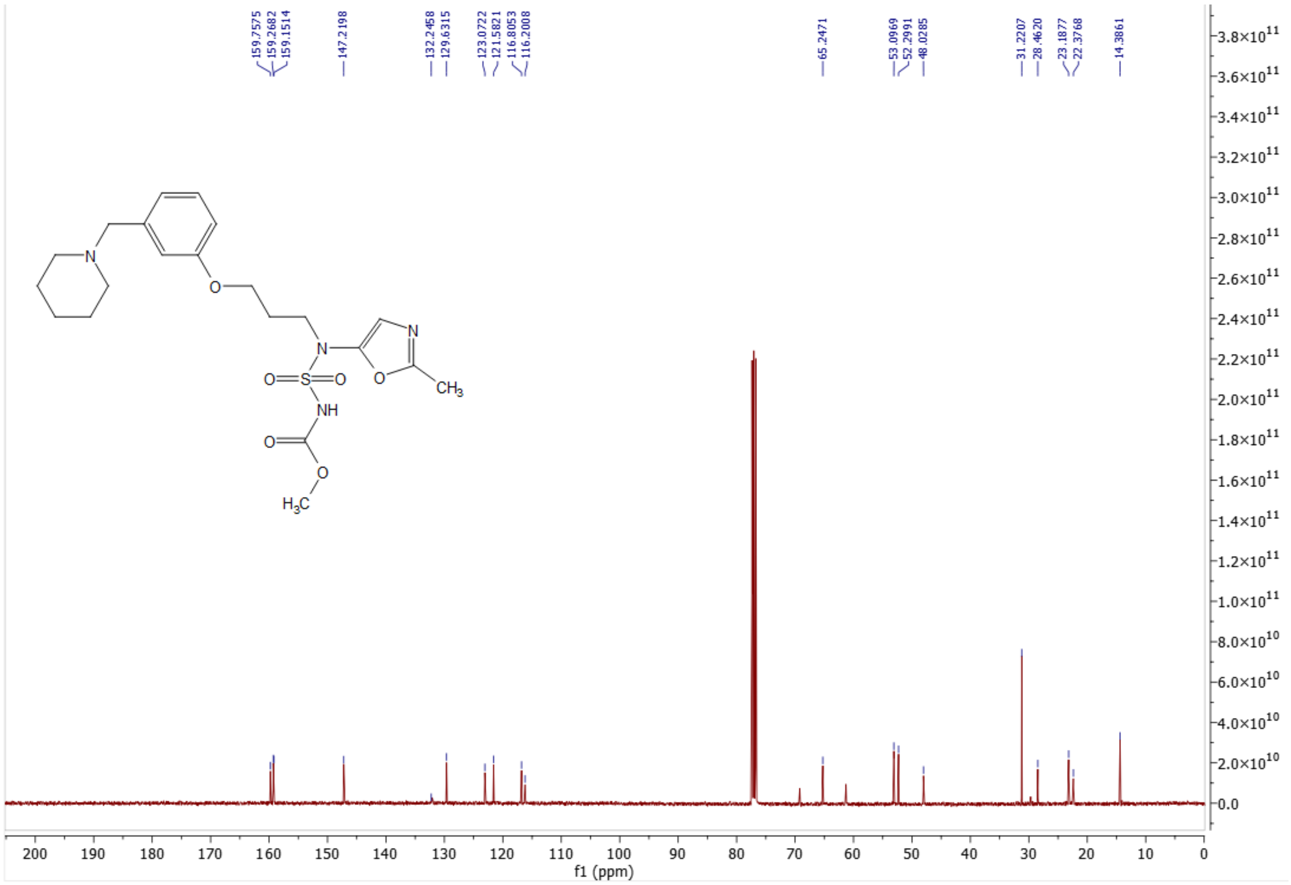

2o: ^{1}H (400 MHz, CD_3OD), ^{13}C (101 MHz, CD_3OD)

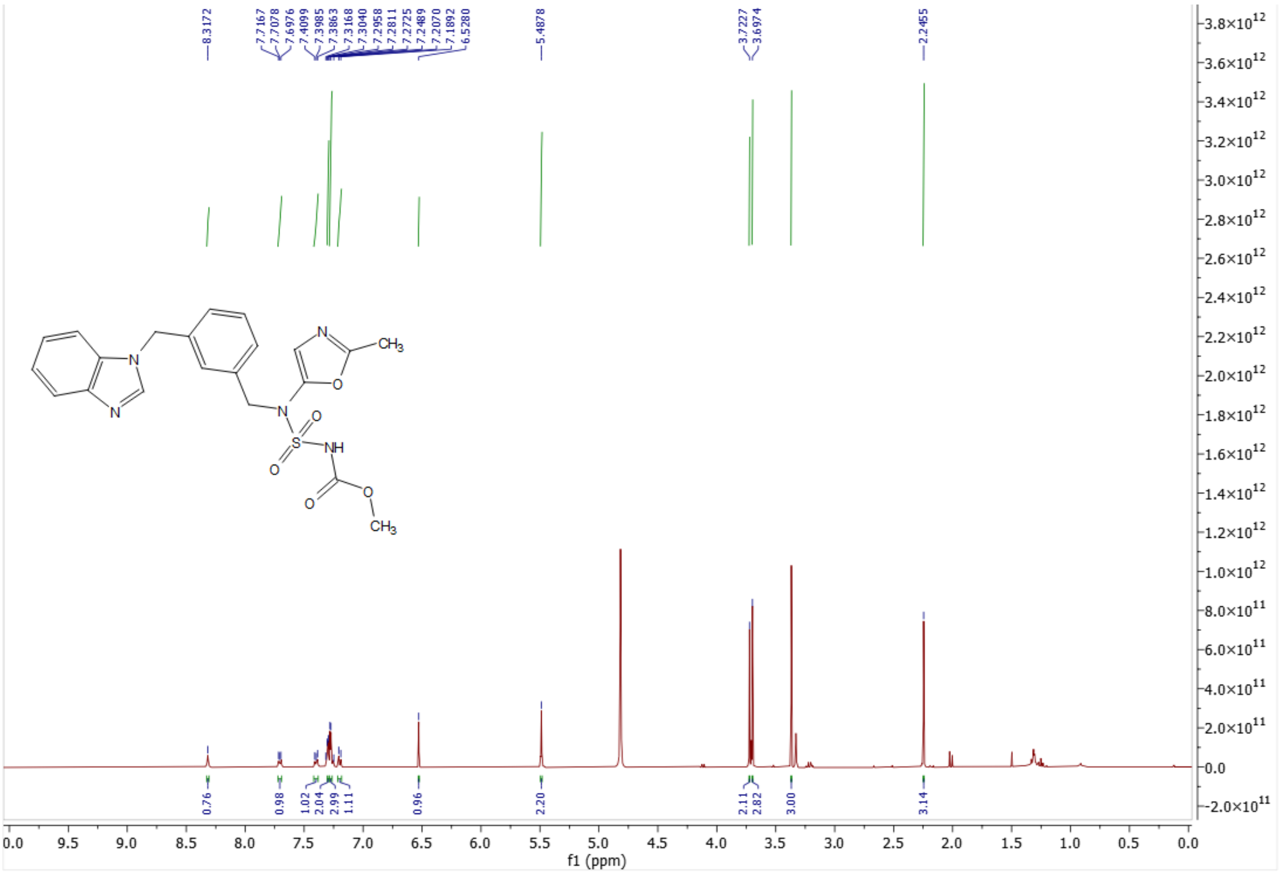

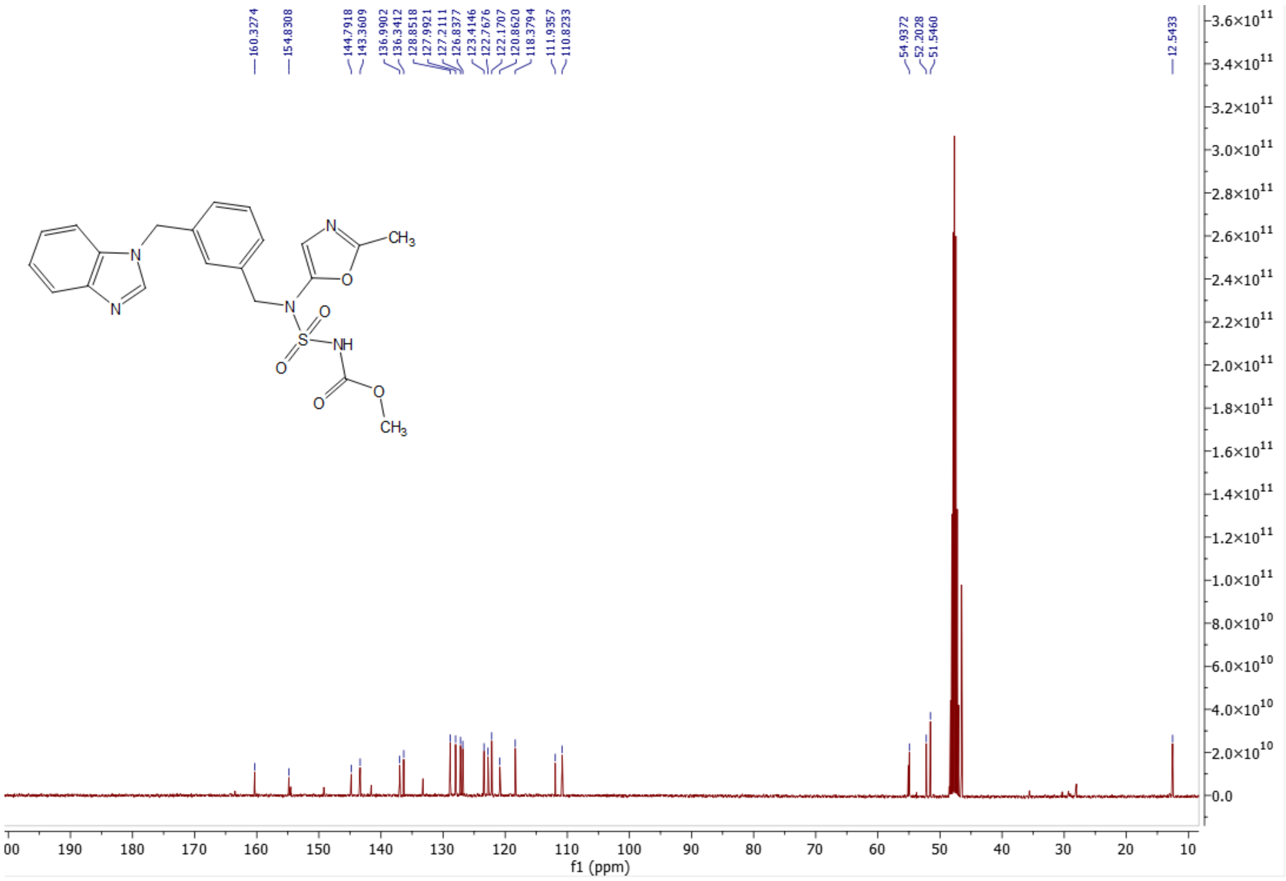

14a: ^{1}H (400 MHz, $CDCl_3$), ^{13}C (101 MHz, $CDCl_3$)

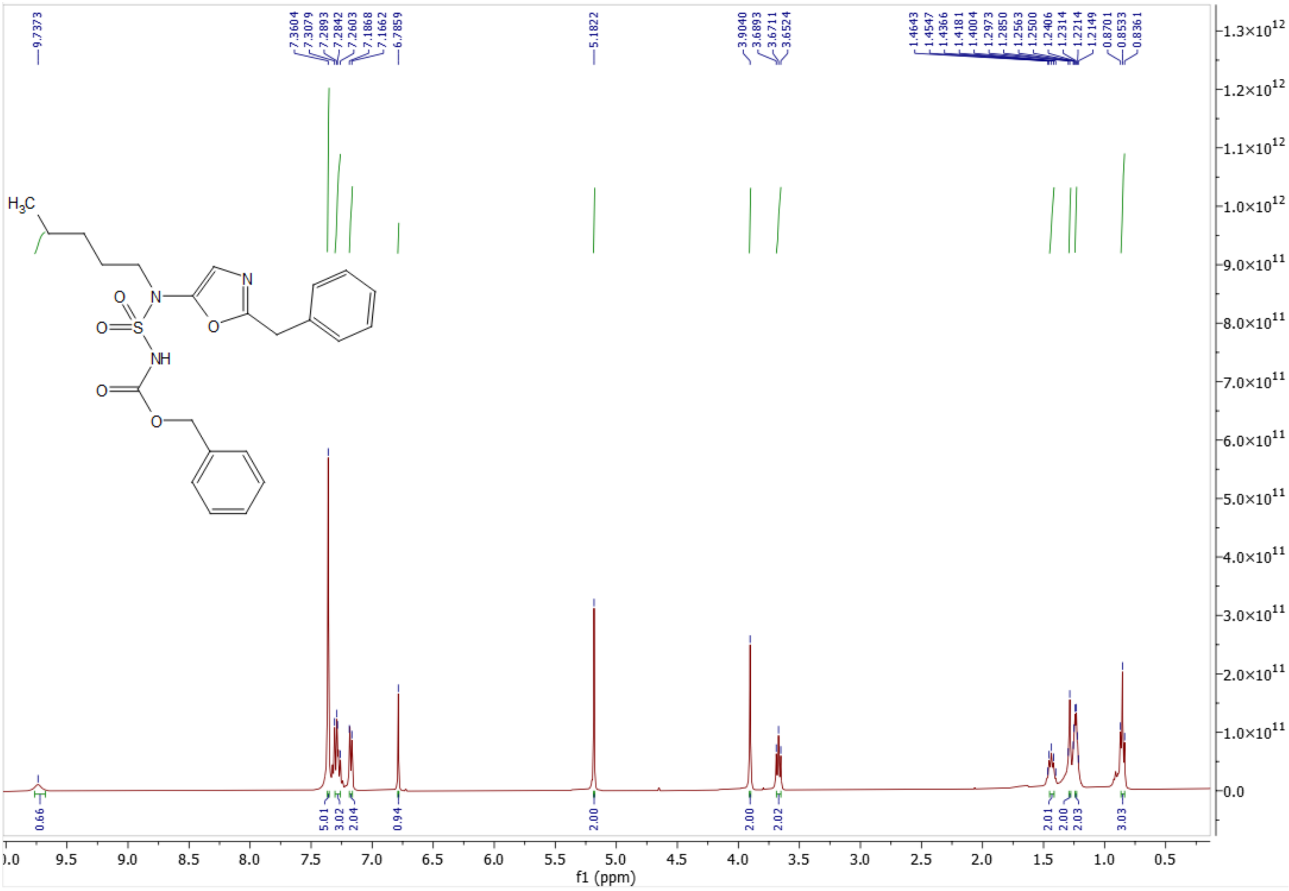

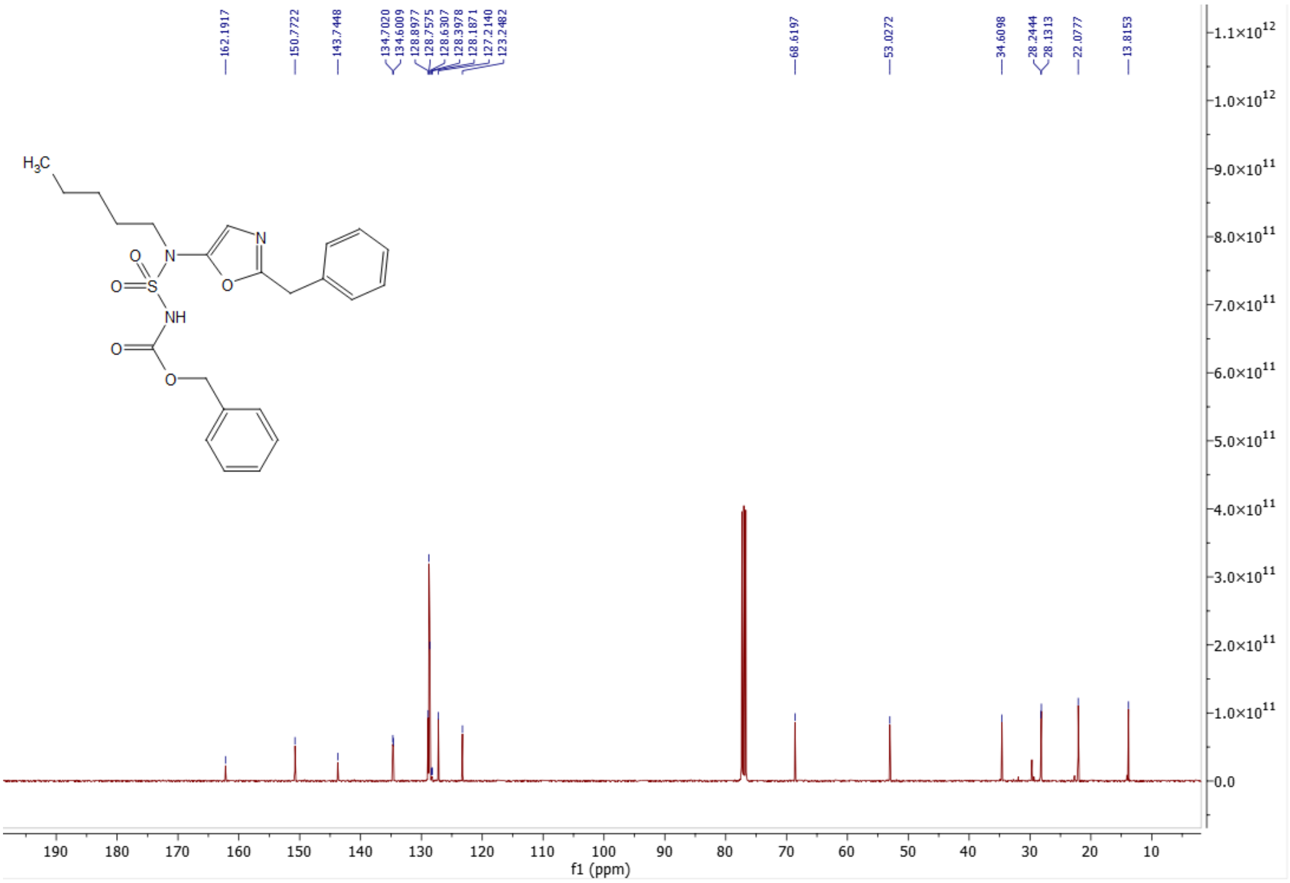

14b: ^{1}H (400 MHz, $CDCl_3$), ^{13}C (101 MHz, $CDCl_3$)

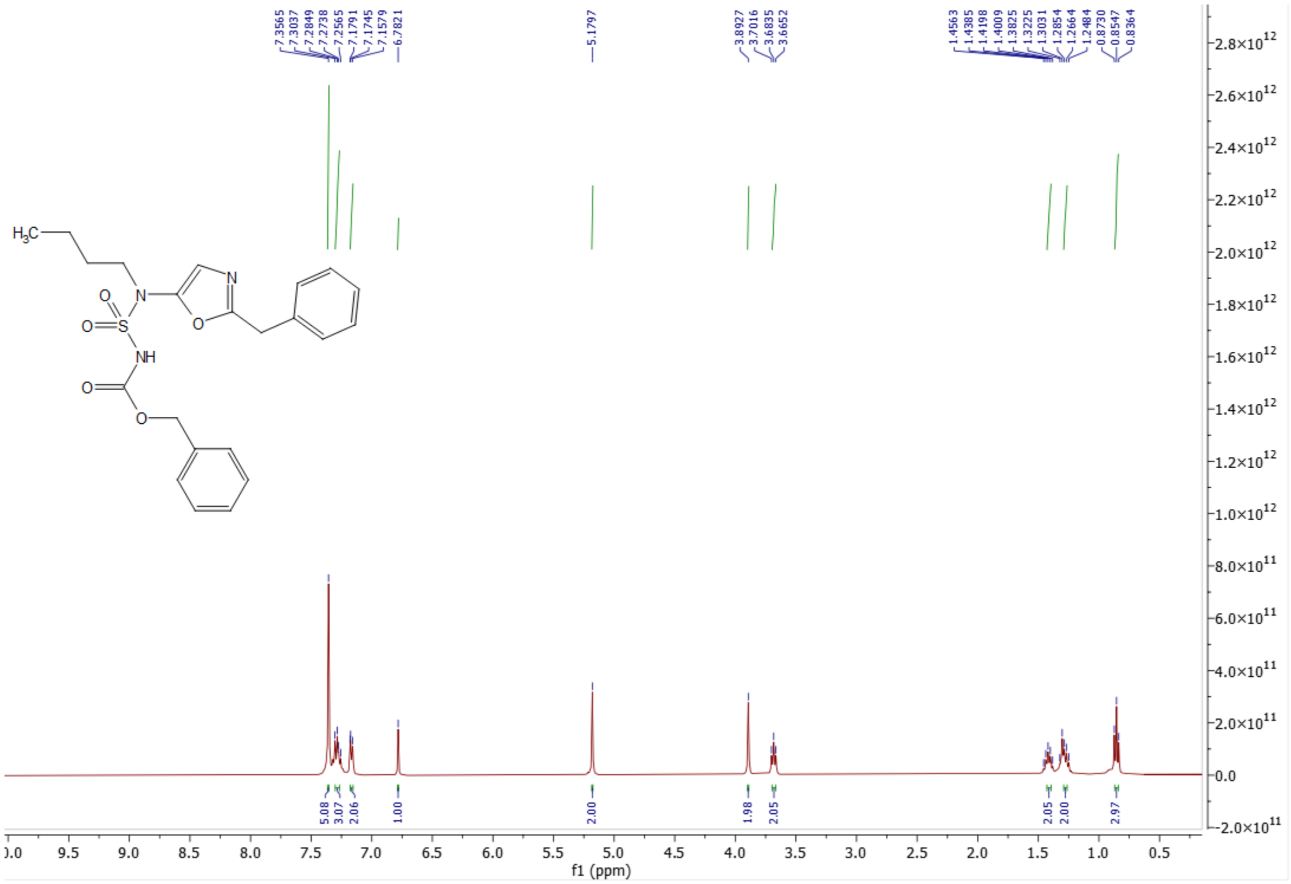

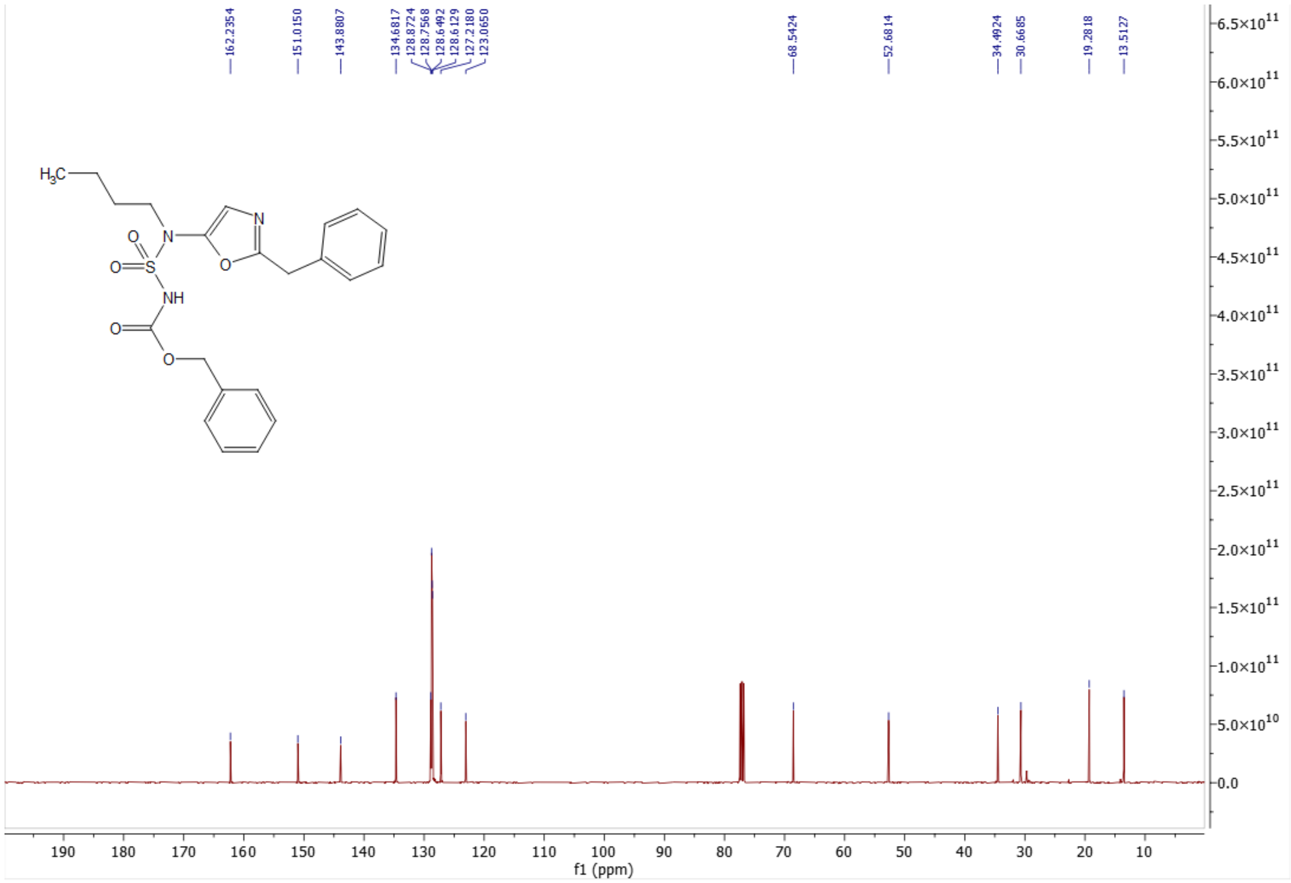

14c: ^{1}H (400 MHz, CDCl$_3$), ^{13}C (101 MHz, CDCl$_3$)

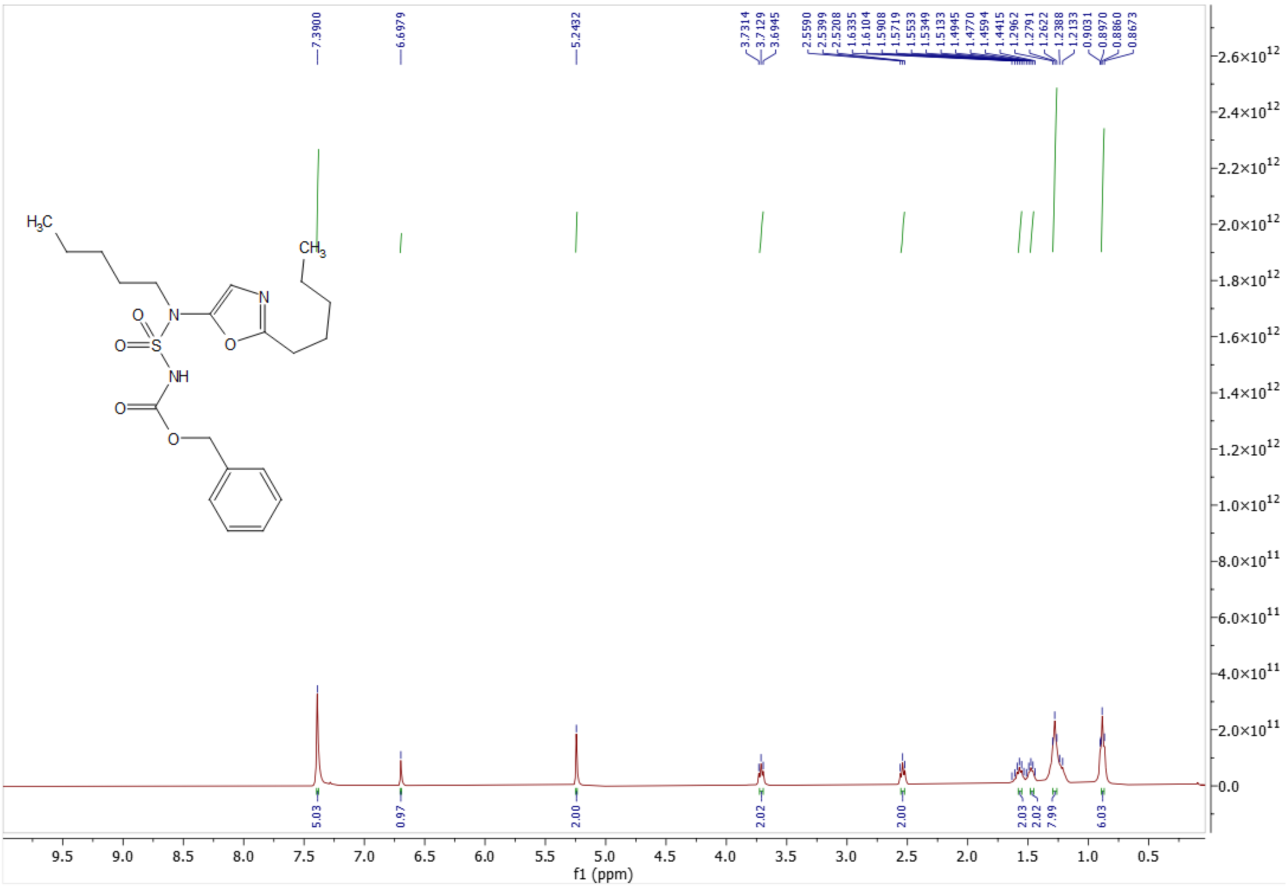

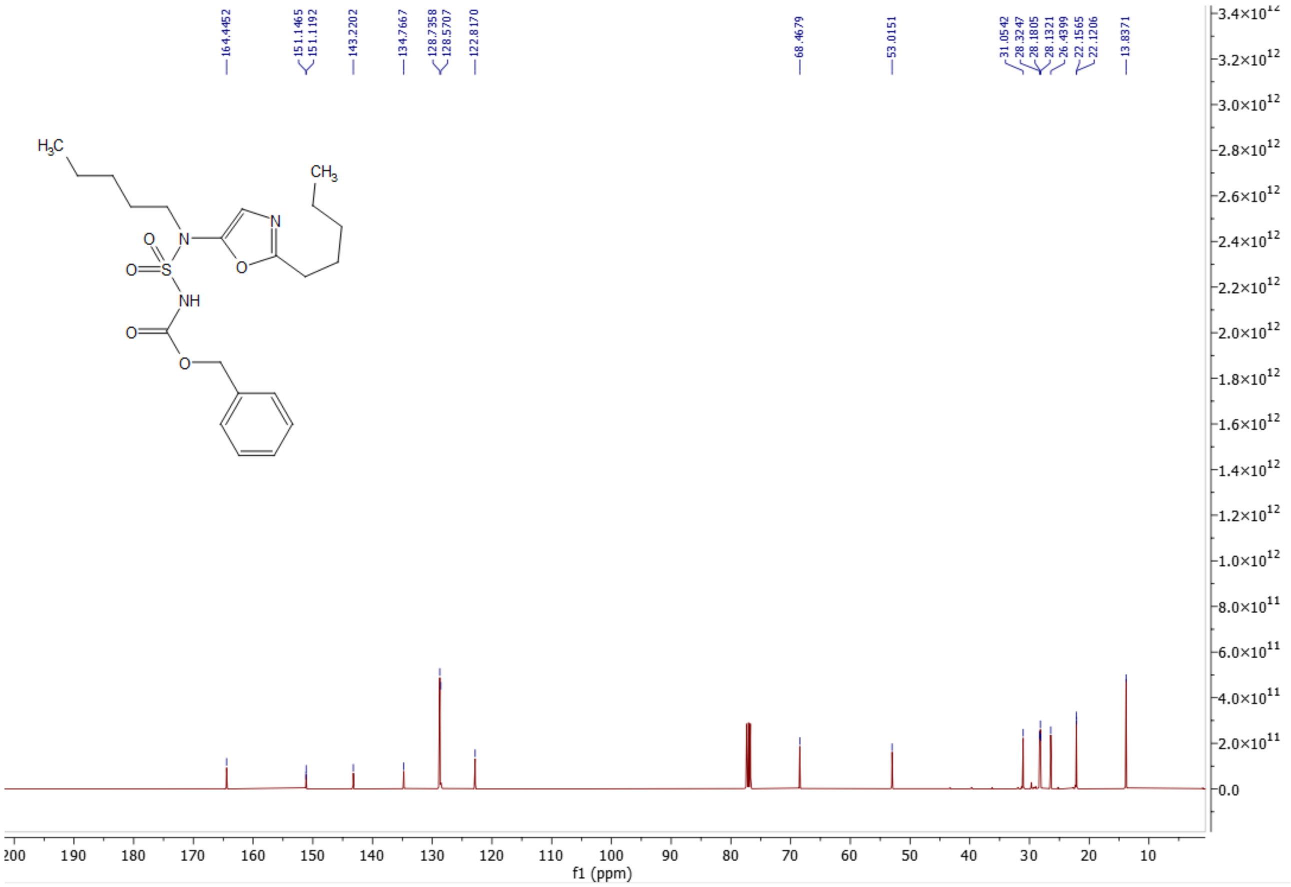

14d: ^{1}H (400 MHz, CD_3OD), ^{13}C (101 MHz, CD_3OD)

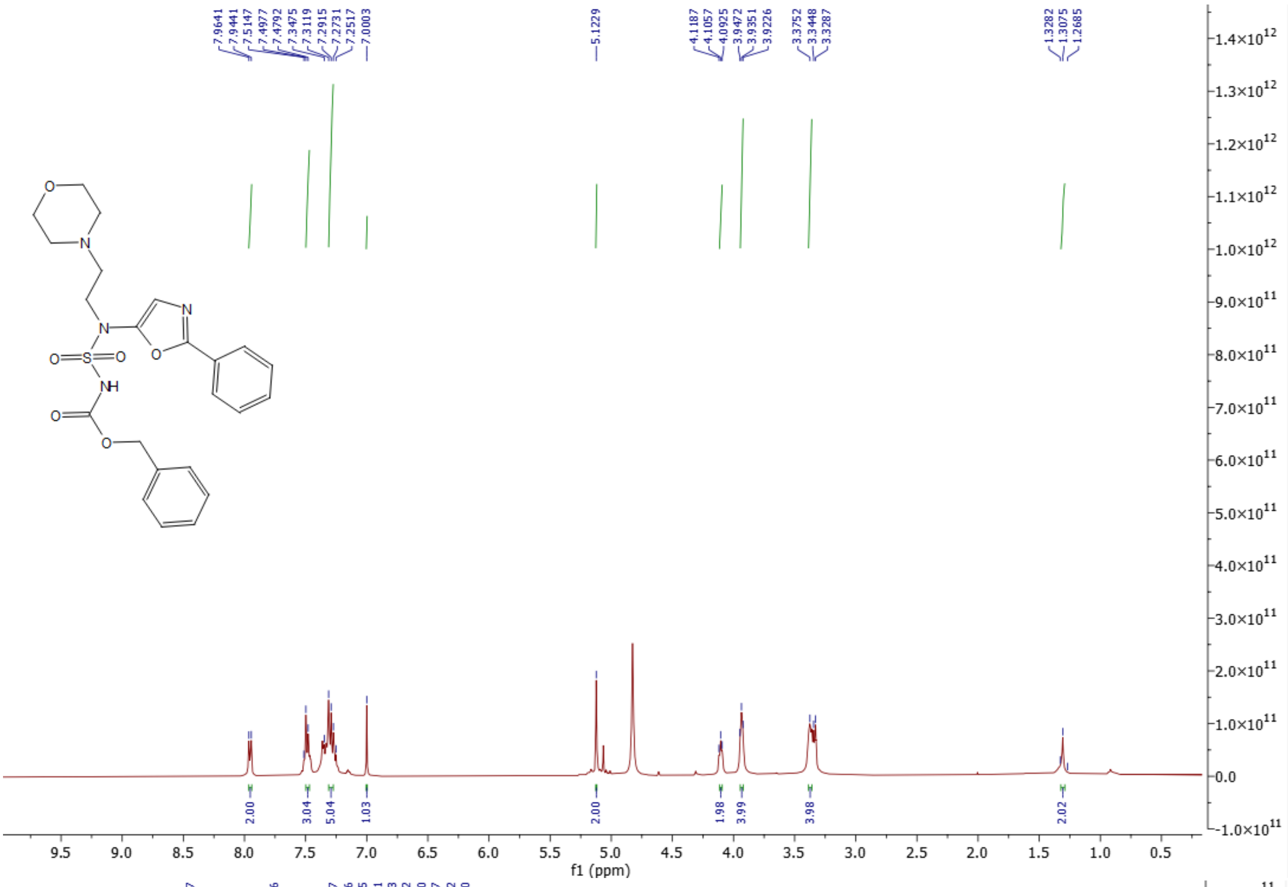

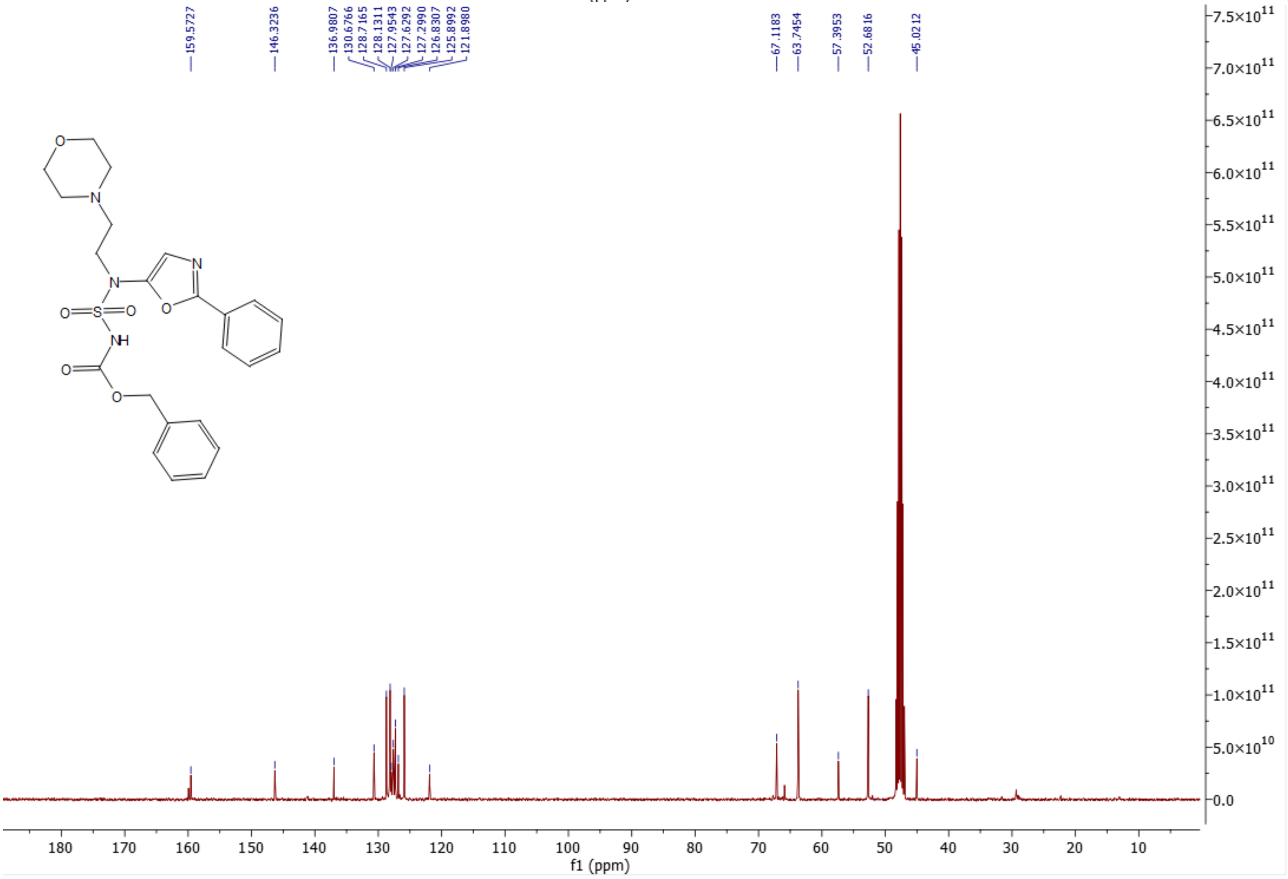

14e: ^{1}H (400 MHz, $CDCl_3$), ^{13}C (101 MHz, $CDCl_3$)

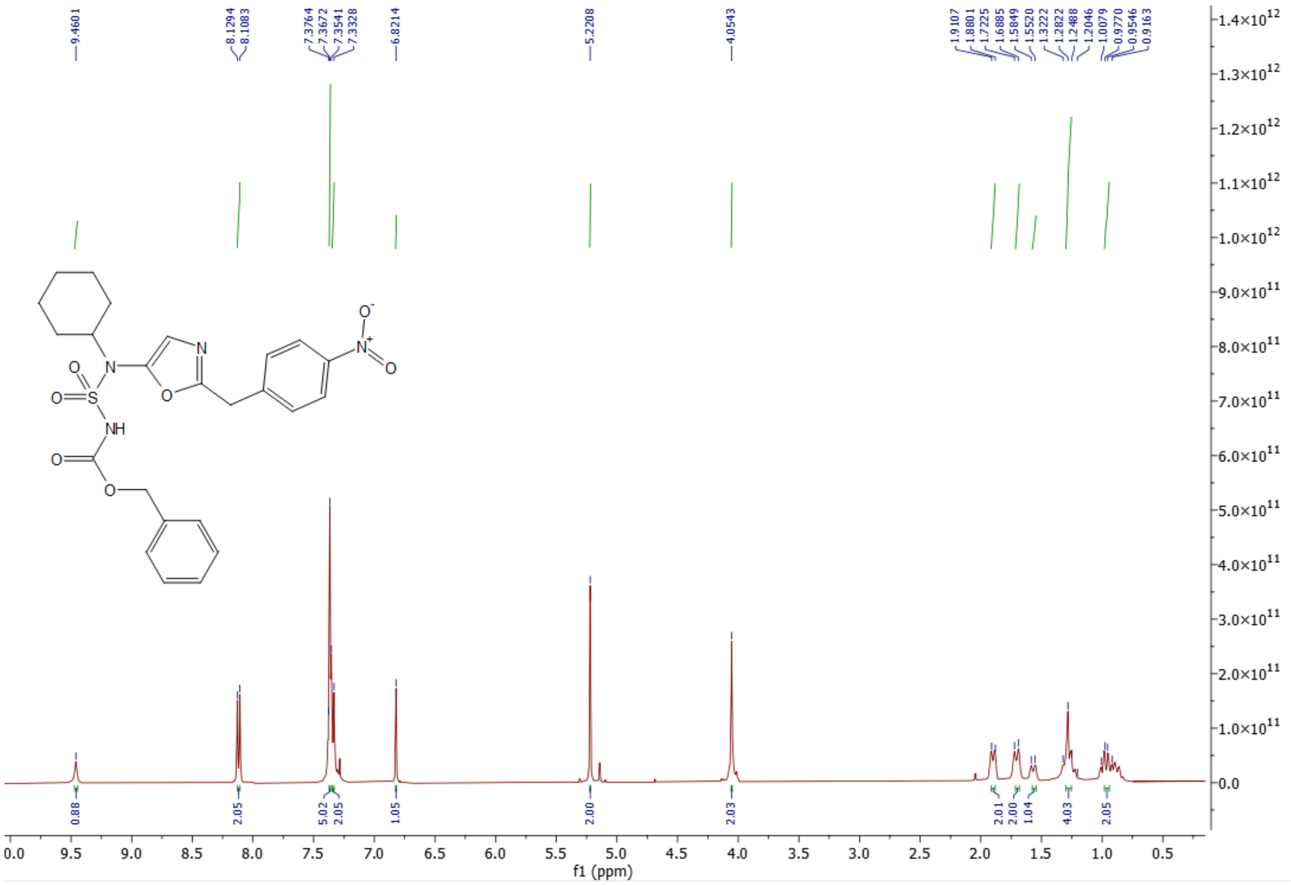

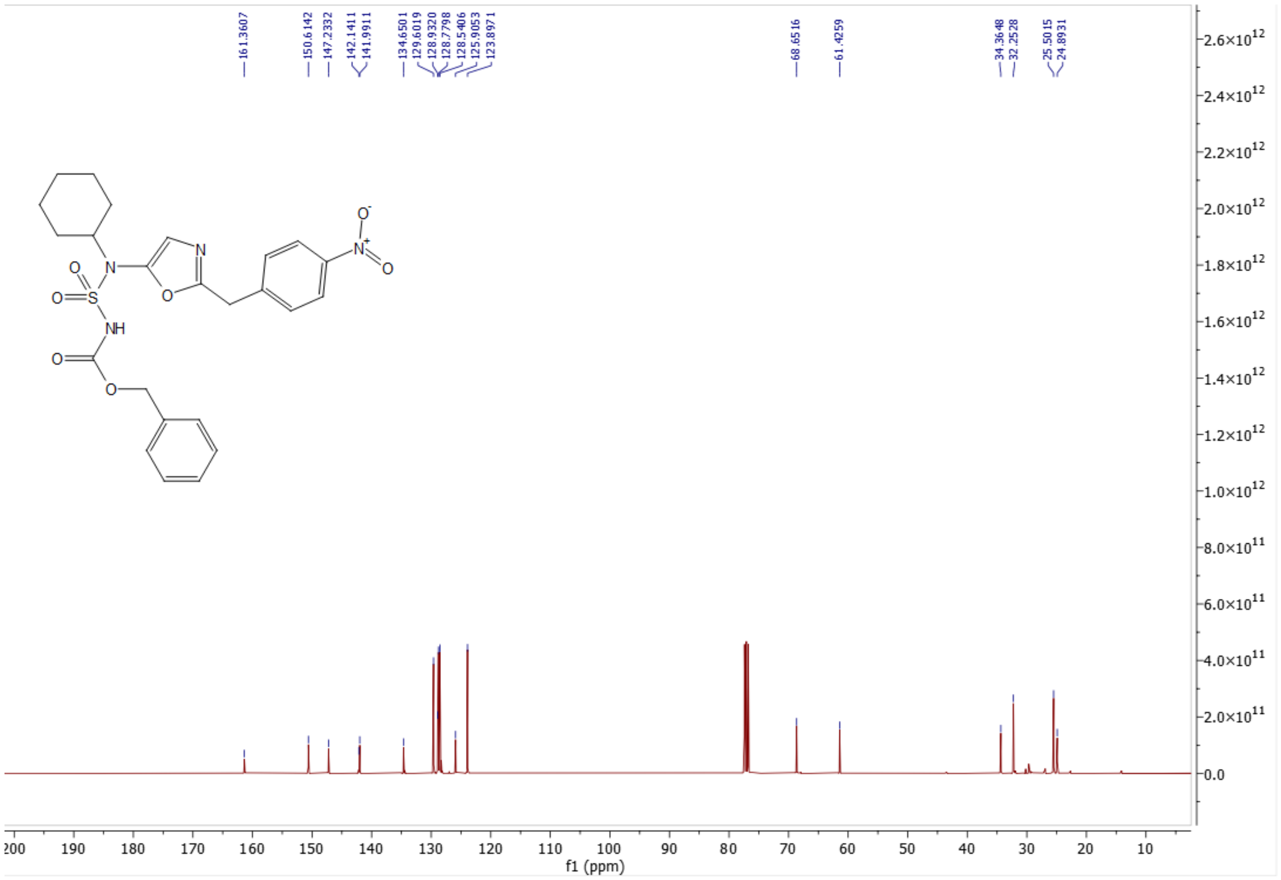

14f: ^{1}H (400 MHz, $CDCl_3$), ^{13}C (101 MHz, $CDCl_3$)

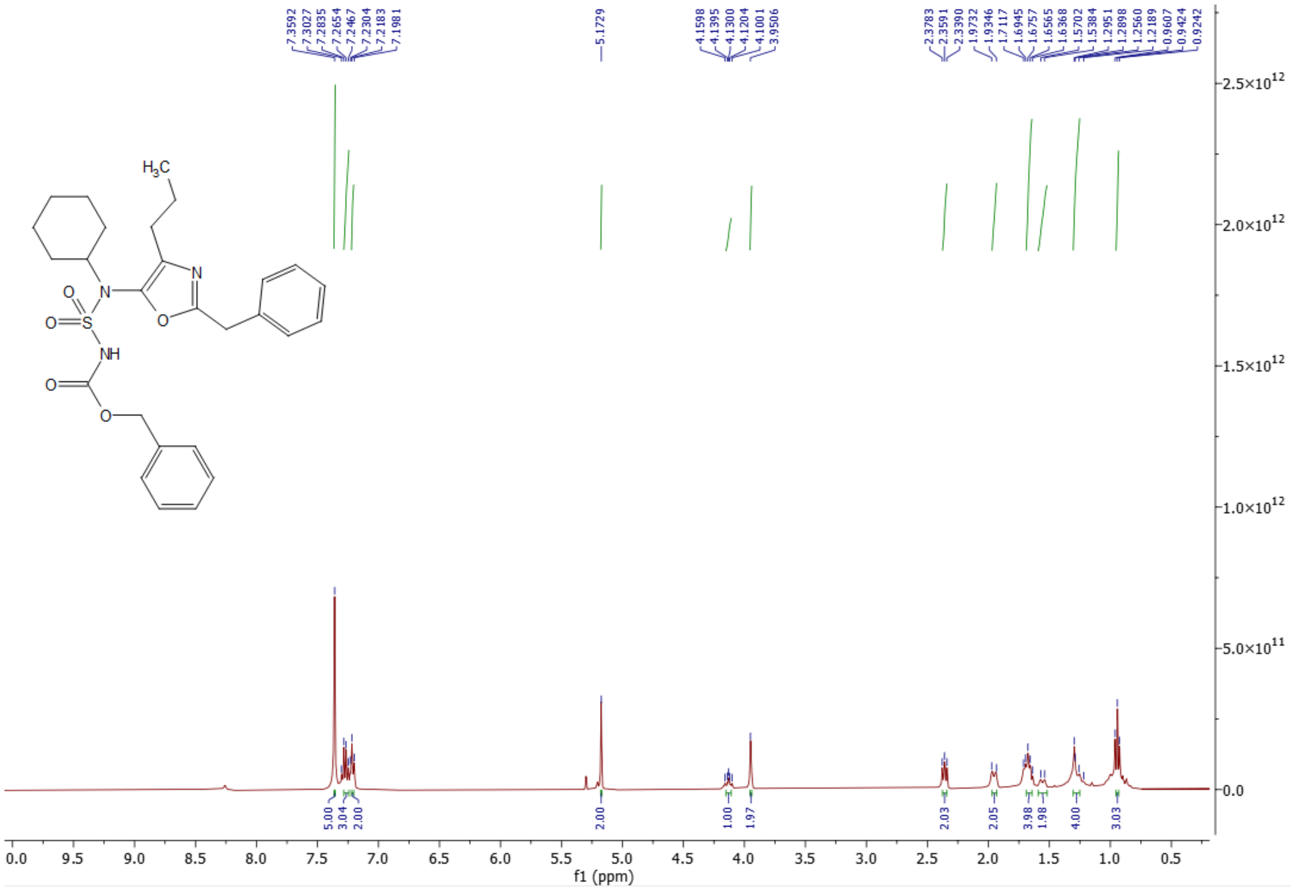

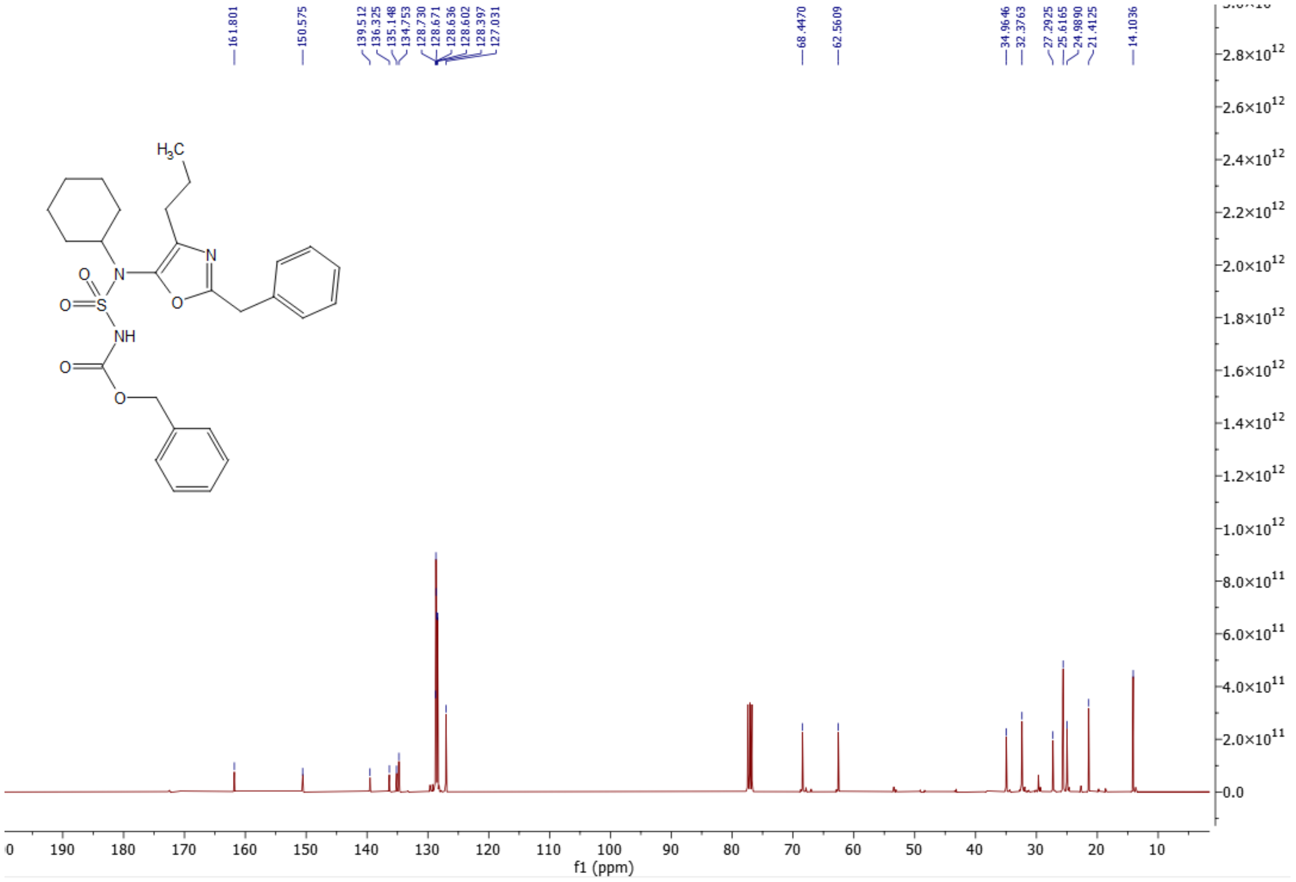

14g: ^{1}H (400 MHz, CDCl$_3$), ^{13}C (101 MHz, CDCl$_3$)

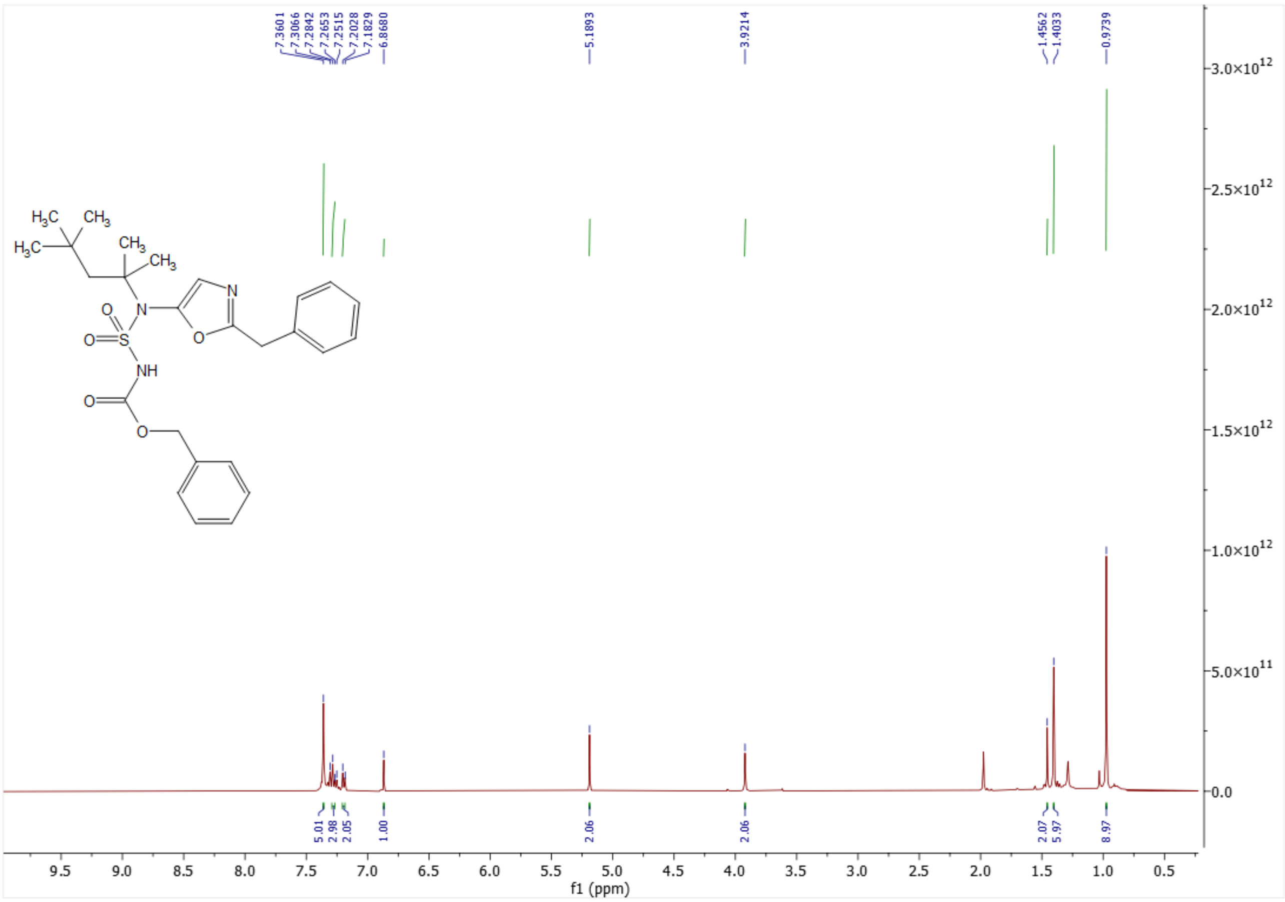

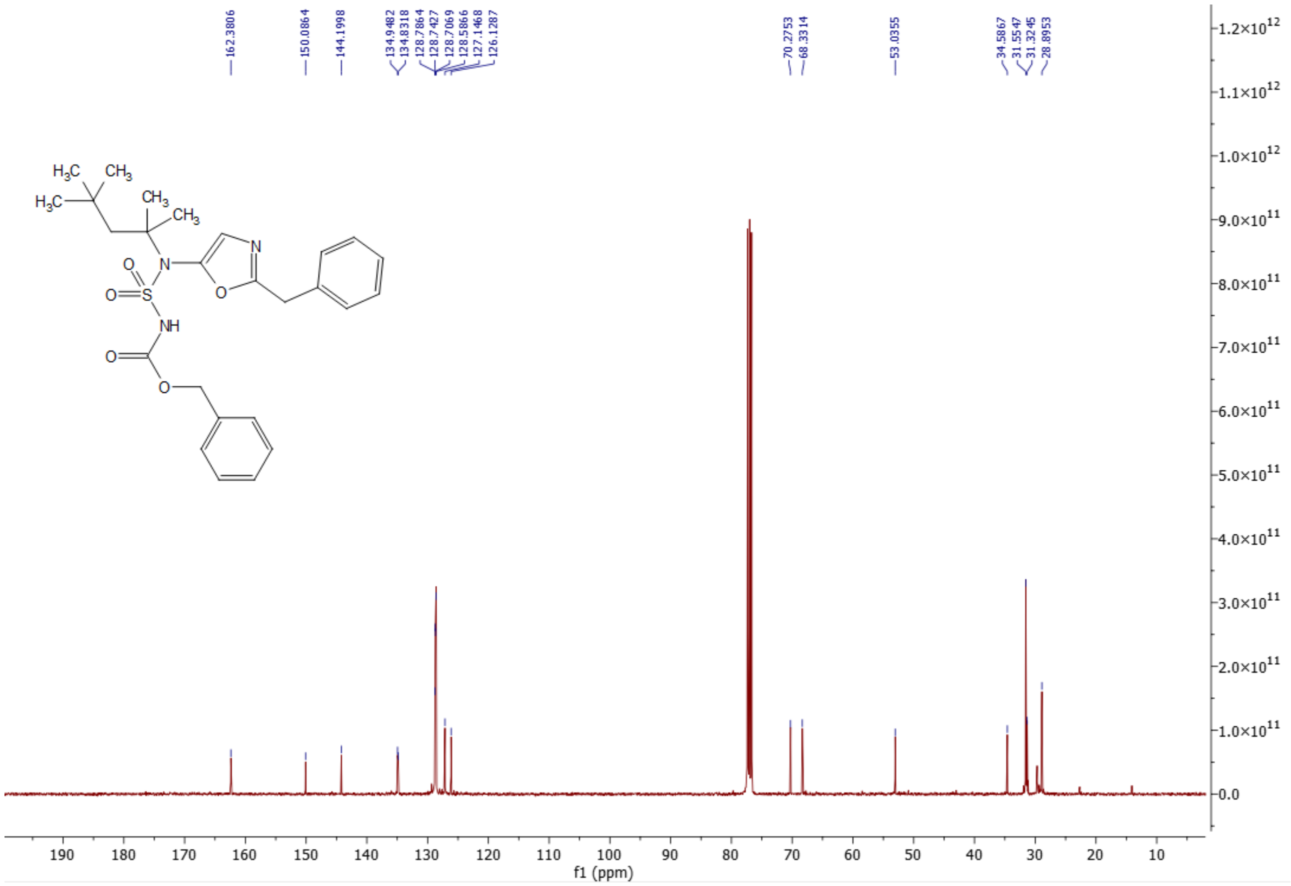

14h: ^{1}H (400 MHz, $CDCl_3$), ^{13}C (101 MHz, $CDCl_3$)

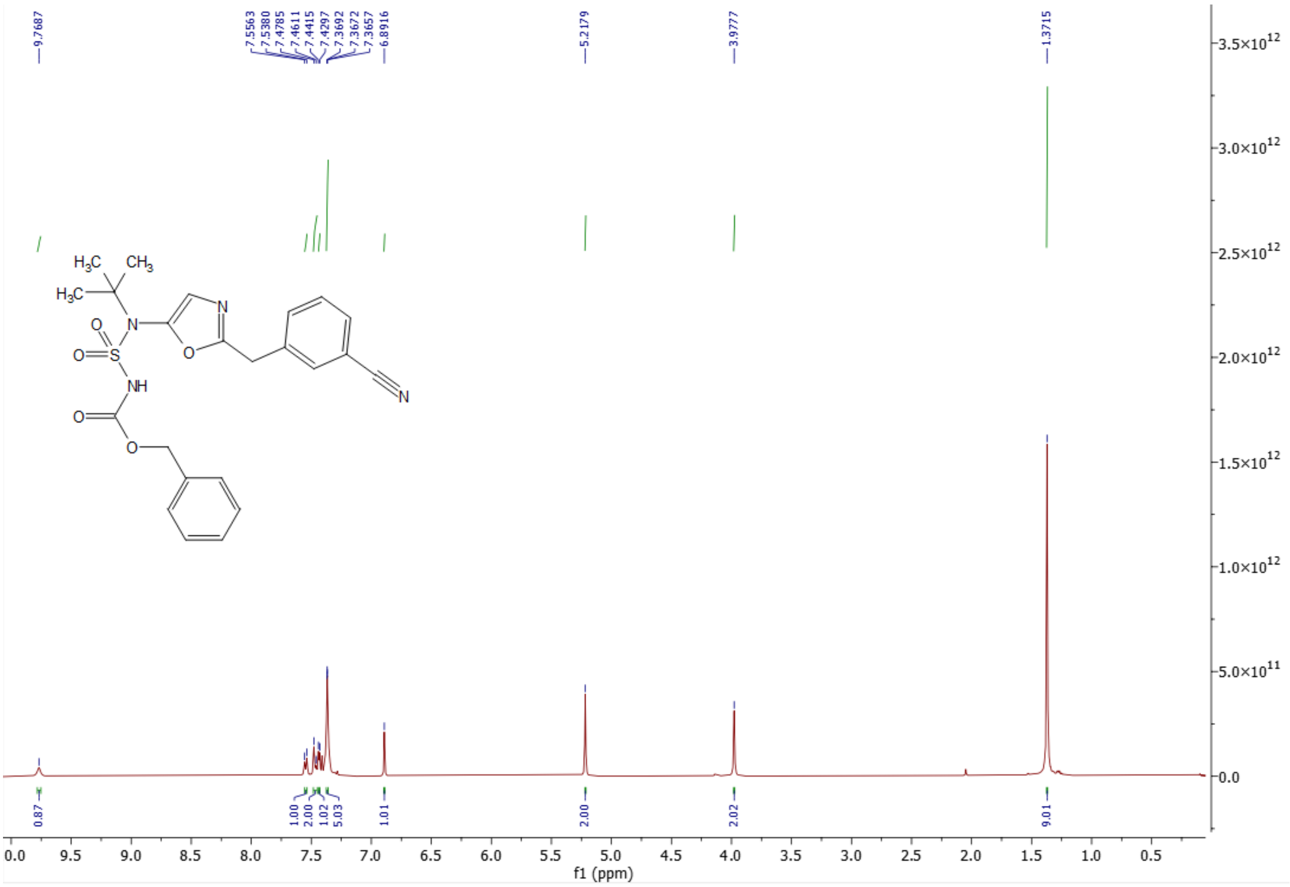

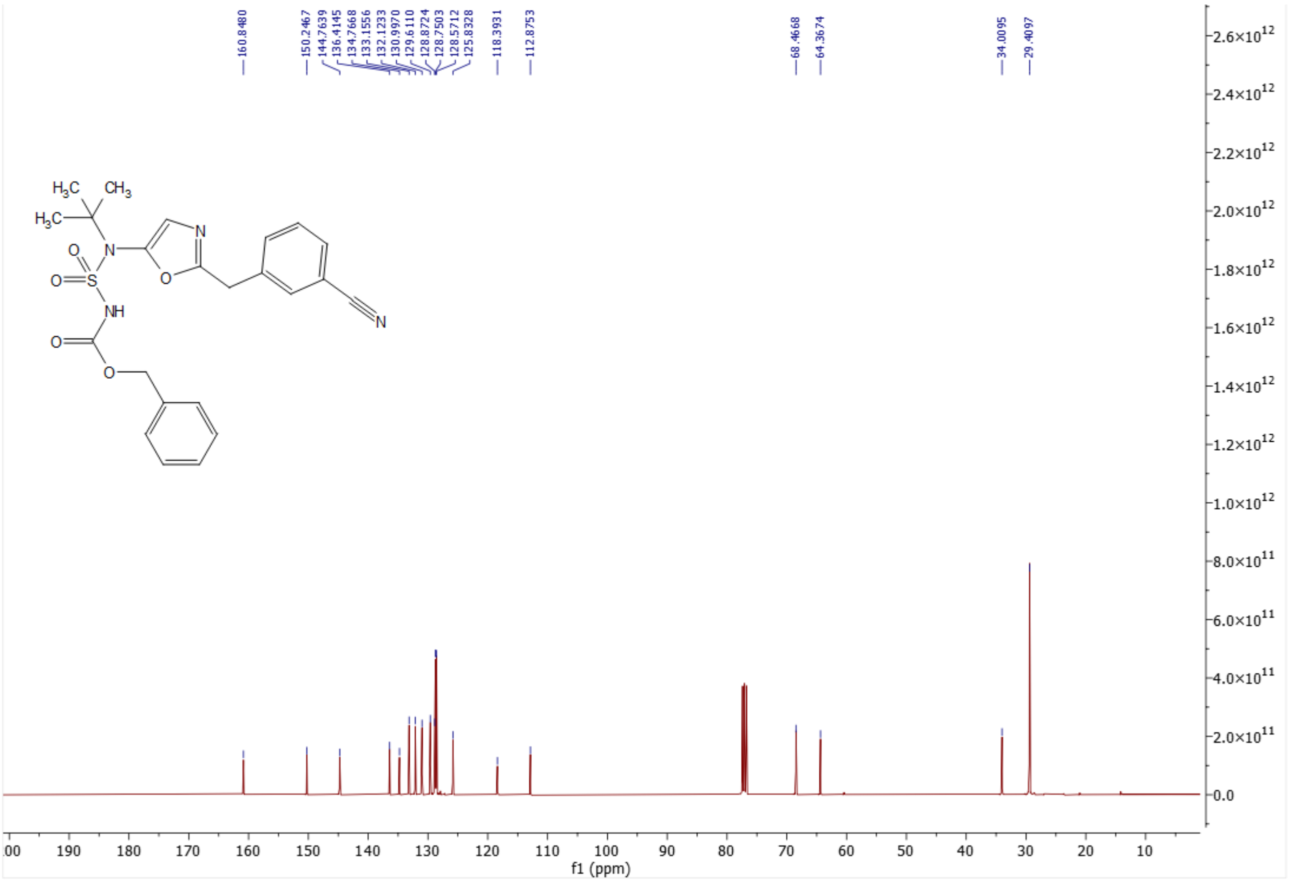

14i: ^{1}H (400 MHz, $CDCl_3$), ^{13}C (101 MHz, $CDCl_3$)

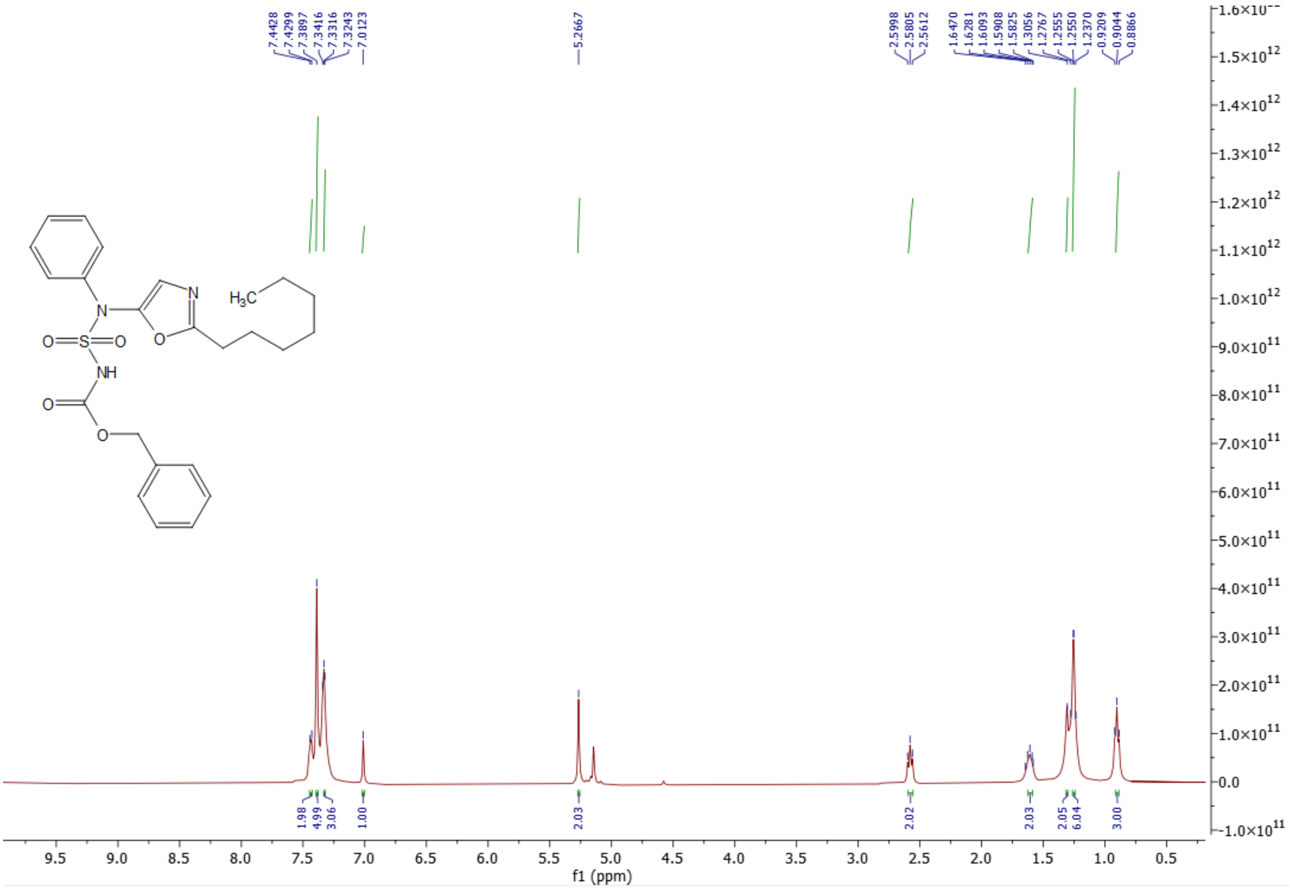

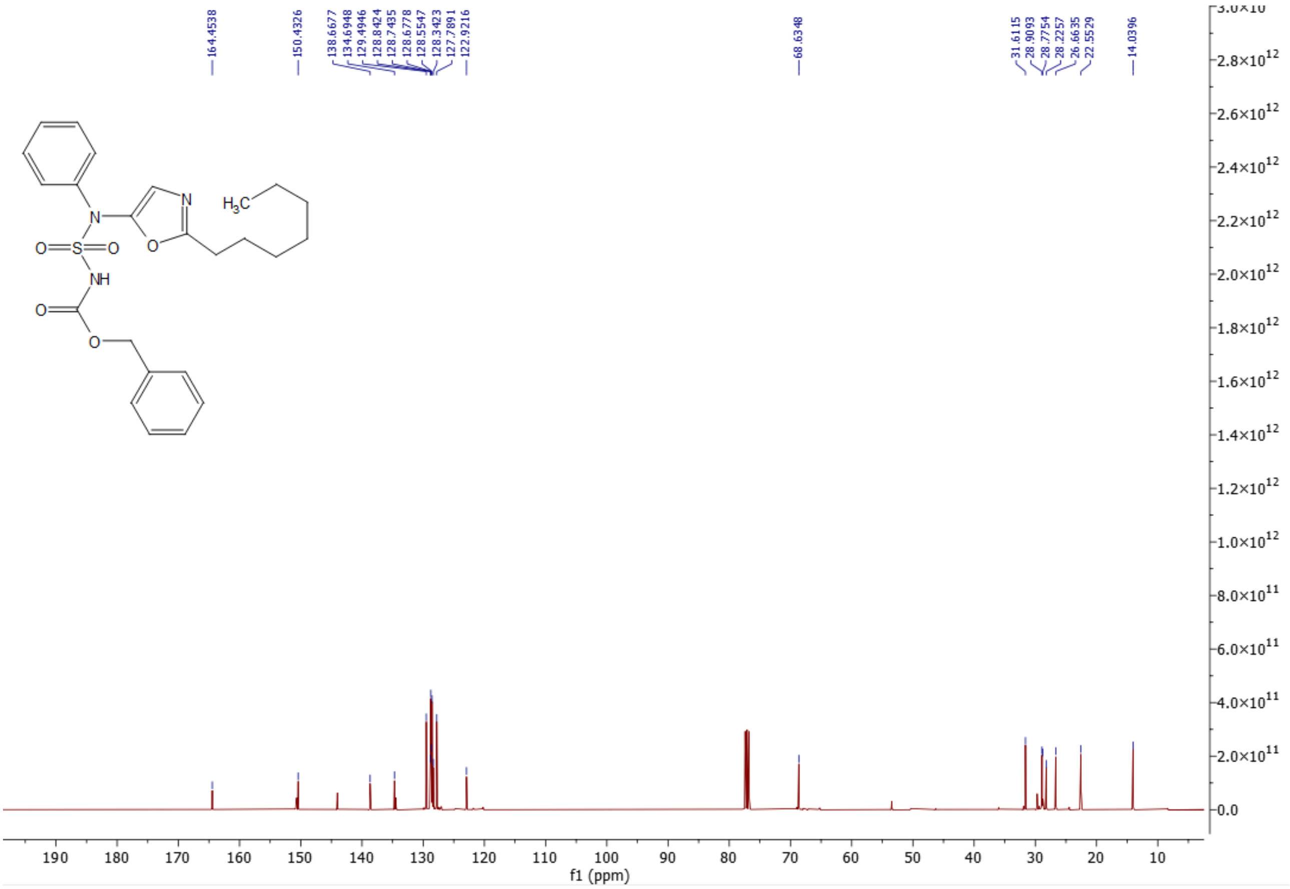

14j: ^{1}H (400 MHz, $CDCl_3$), ^{13}C (101 MHz, $CDCl_3$)

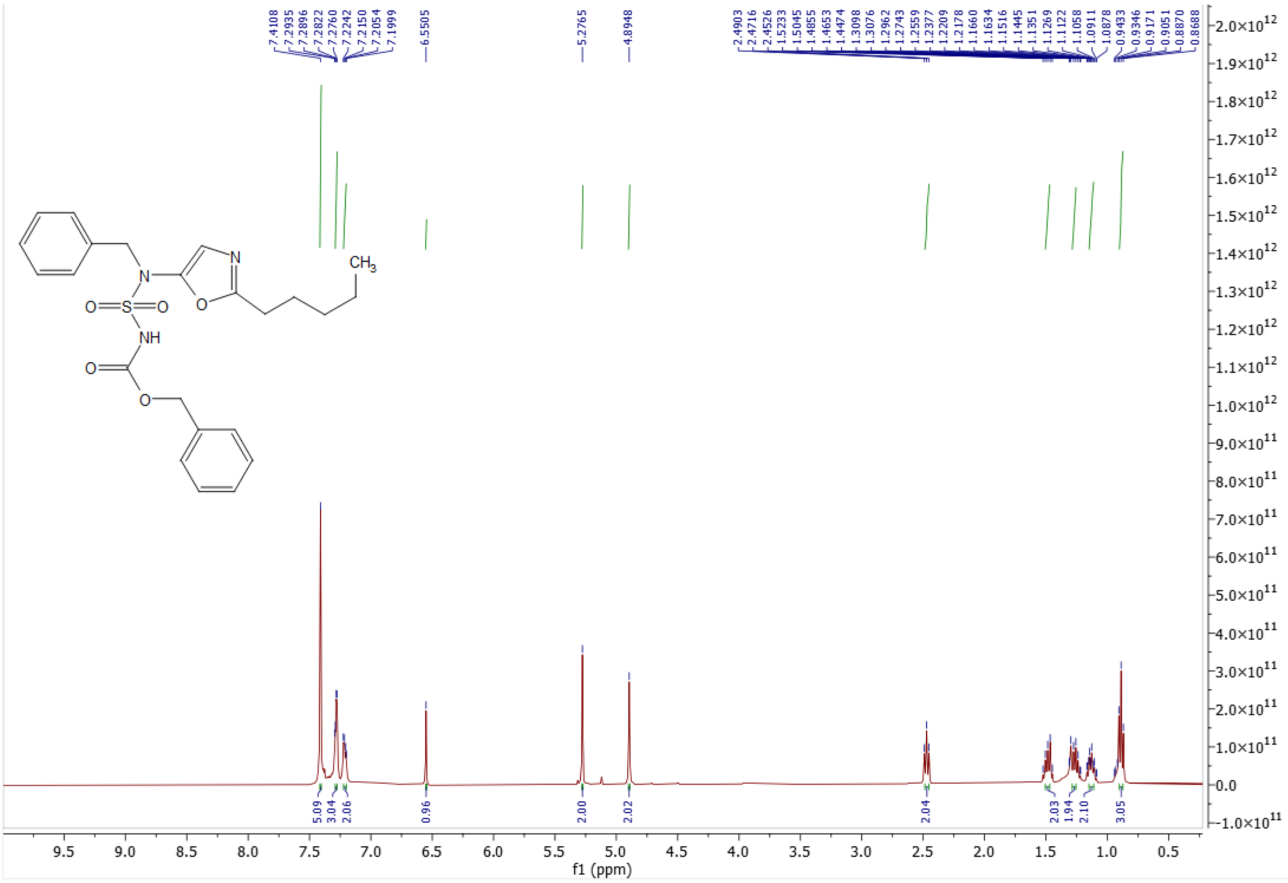

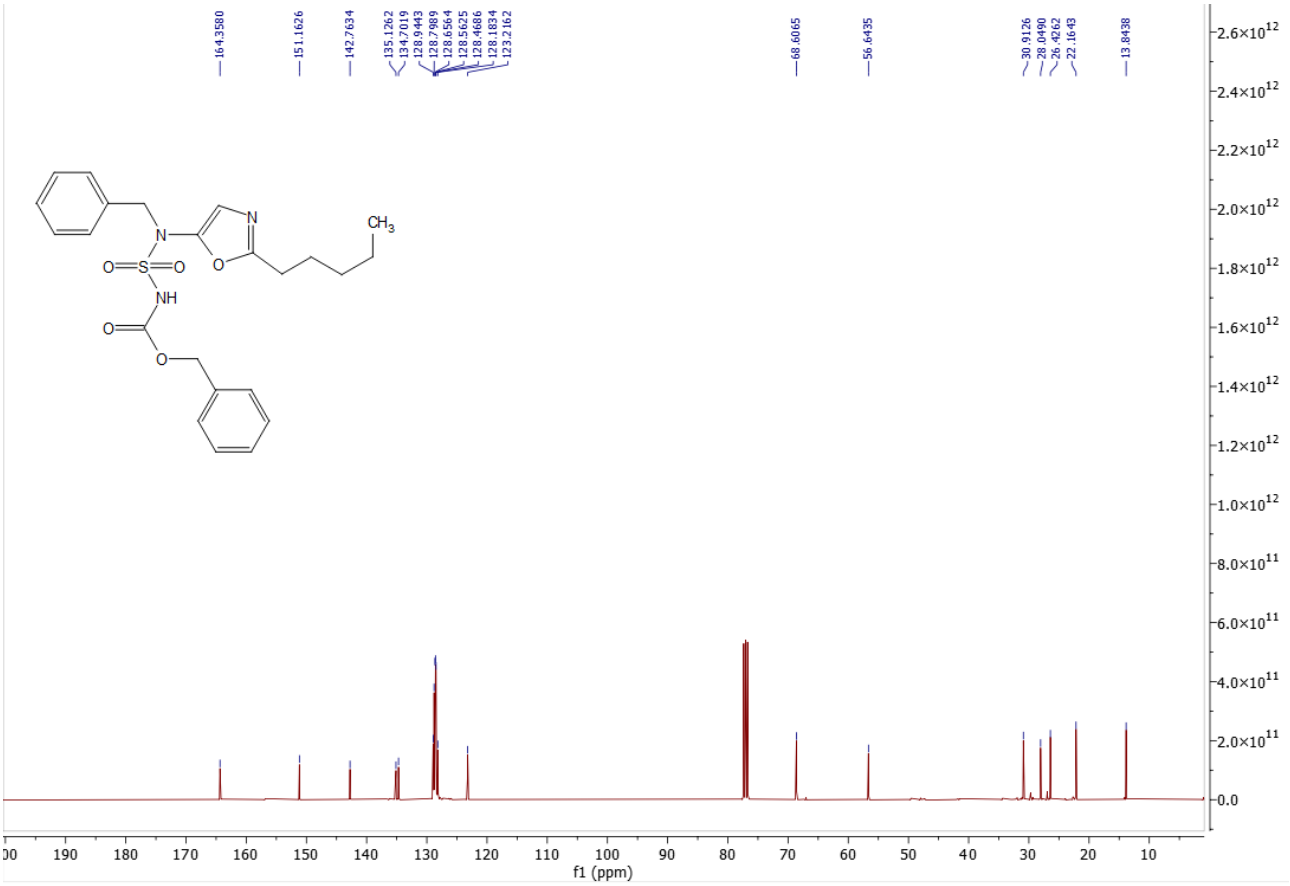

14k: ^{1}H (400 MHz, $CDCl_3$), ^{13}C (101 MHz, $CDCl_3$)

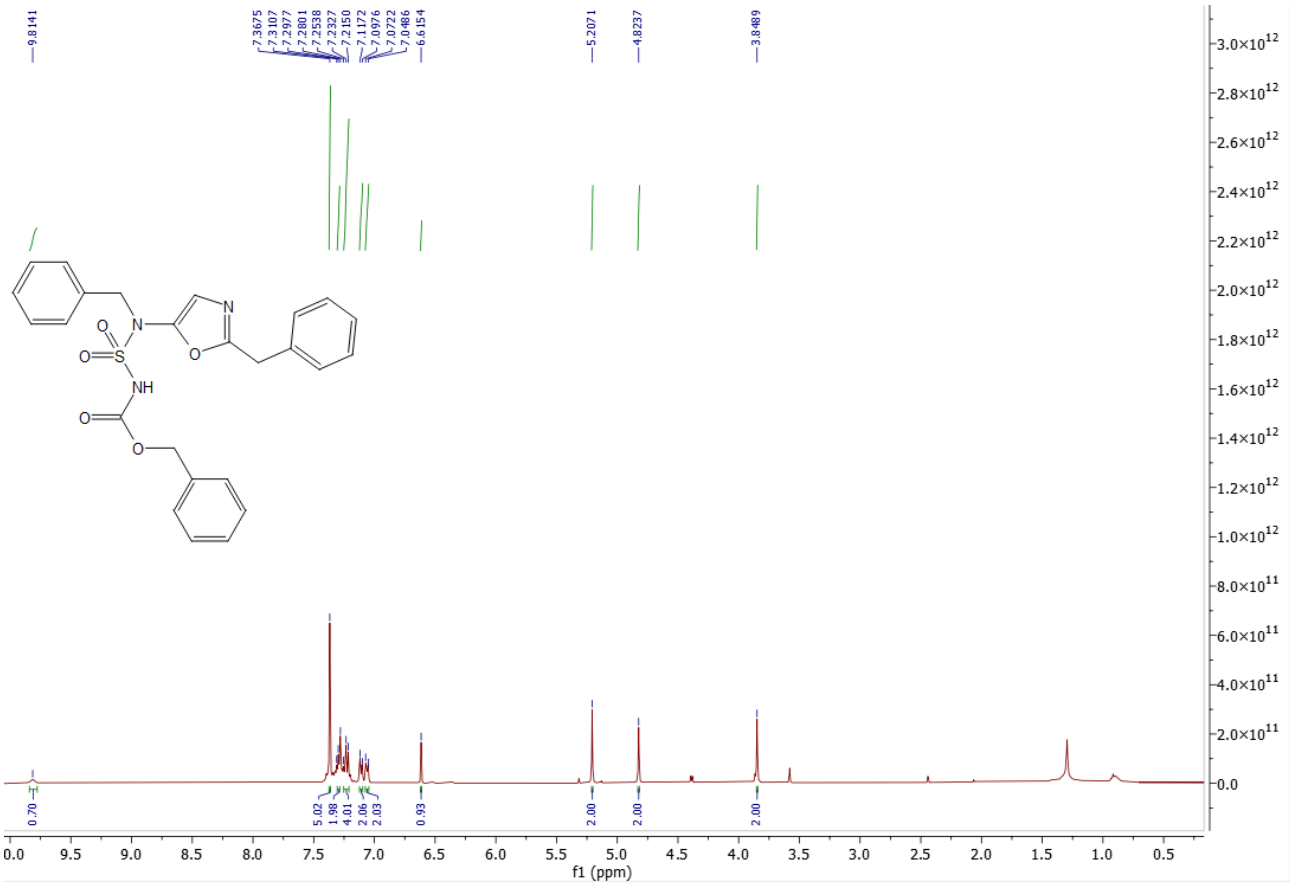

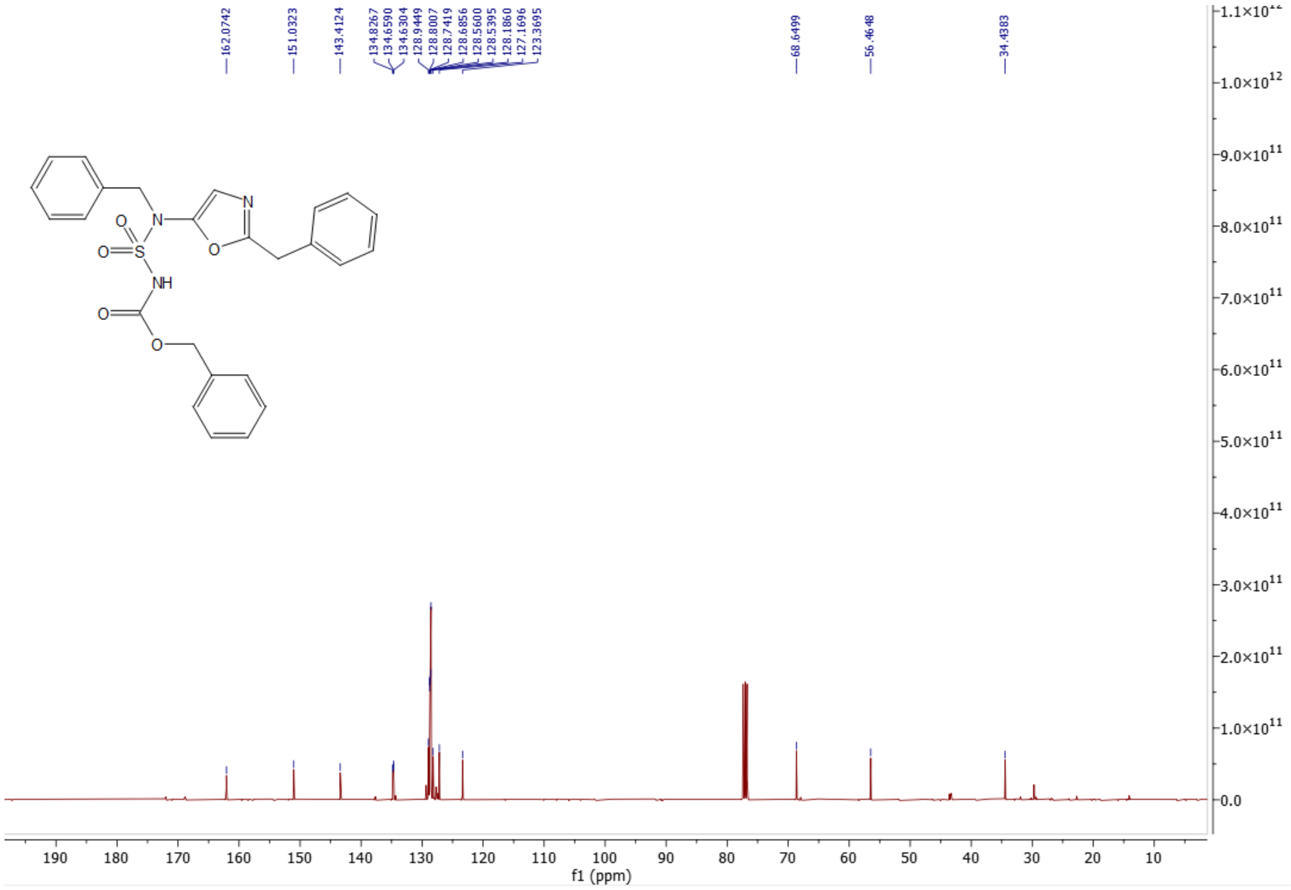

14l: ^{1}H (400 MHz, $CDCl_3$), ^{13}C (101 MHz, $CDCl_3$)

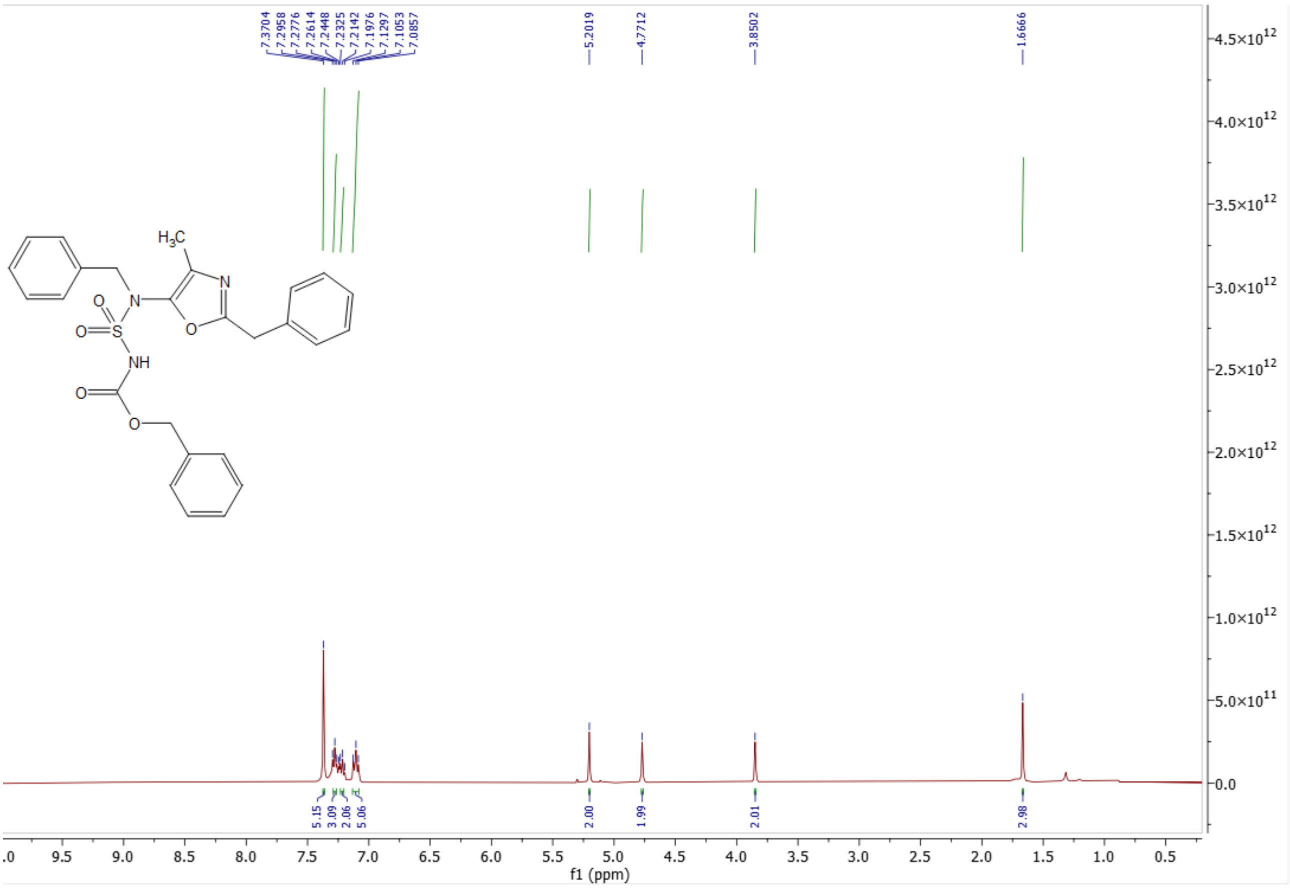

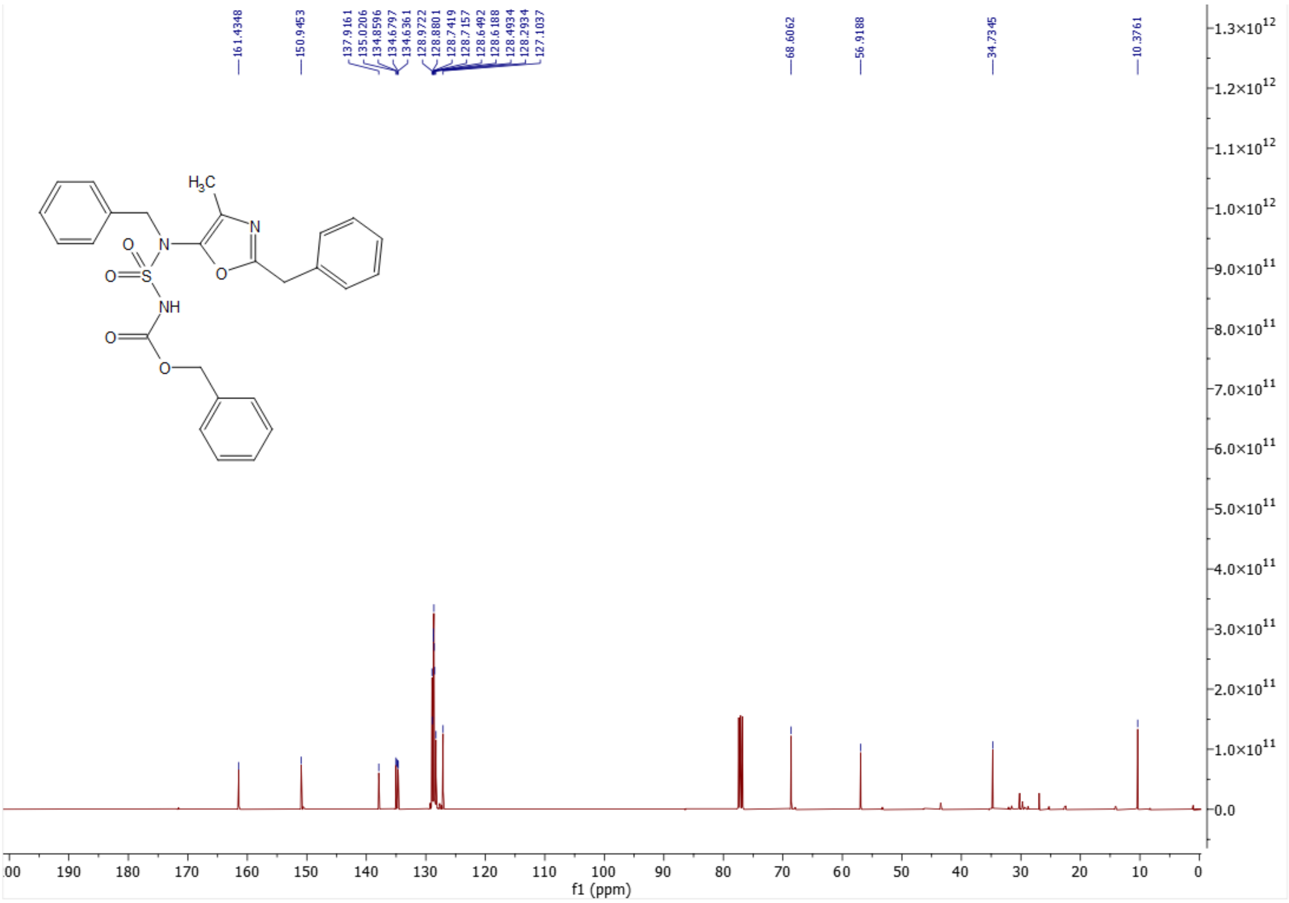

14m: ^{1}H (400 MHz, $CDCl_3$), ^{13}C (101 MHz, $CDCl_3$)

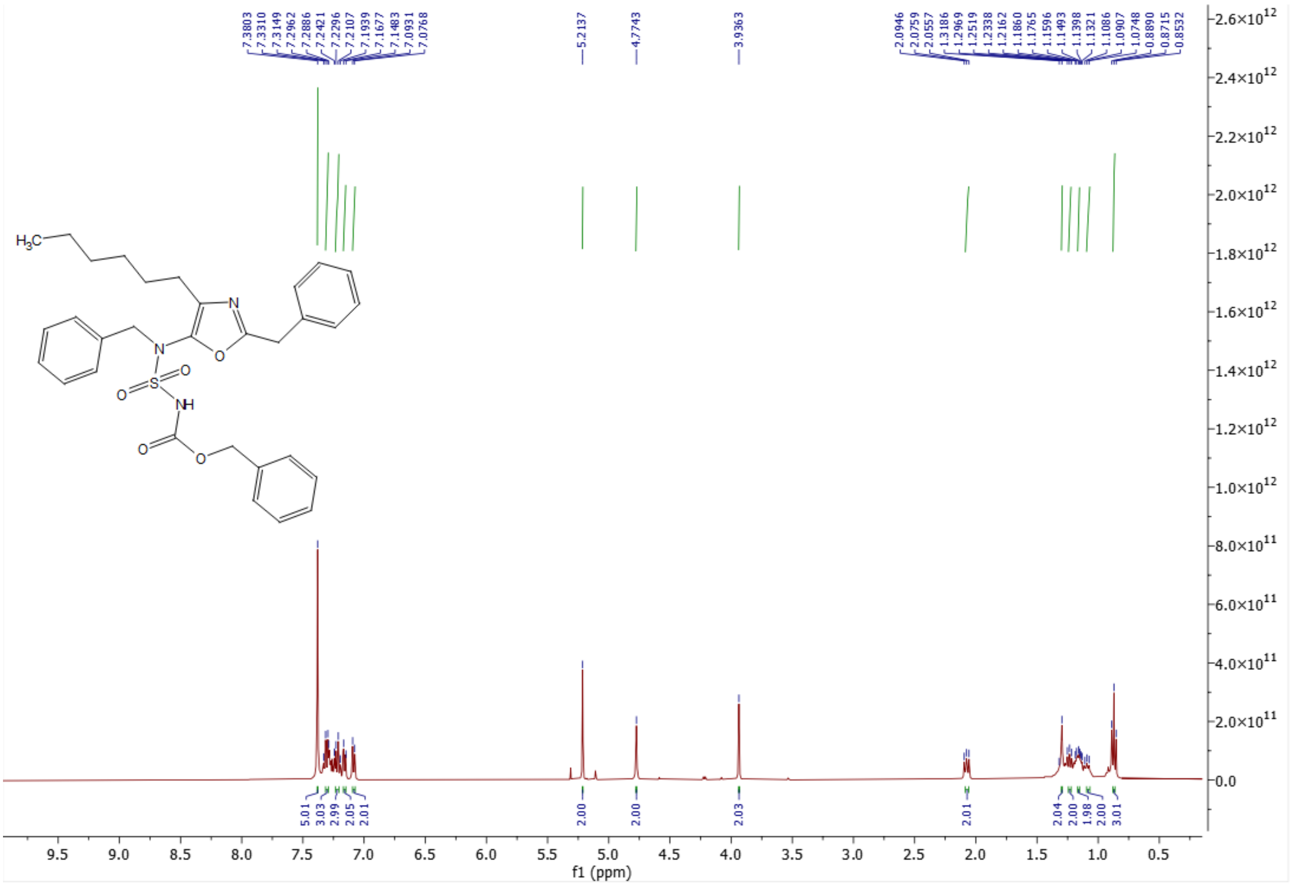

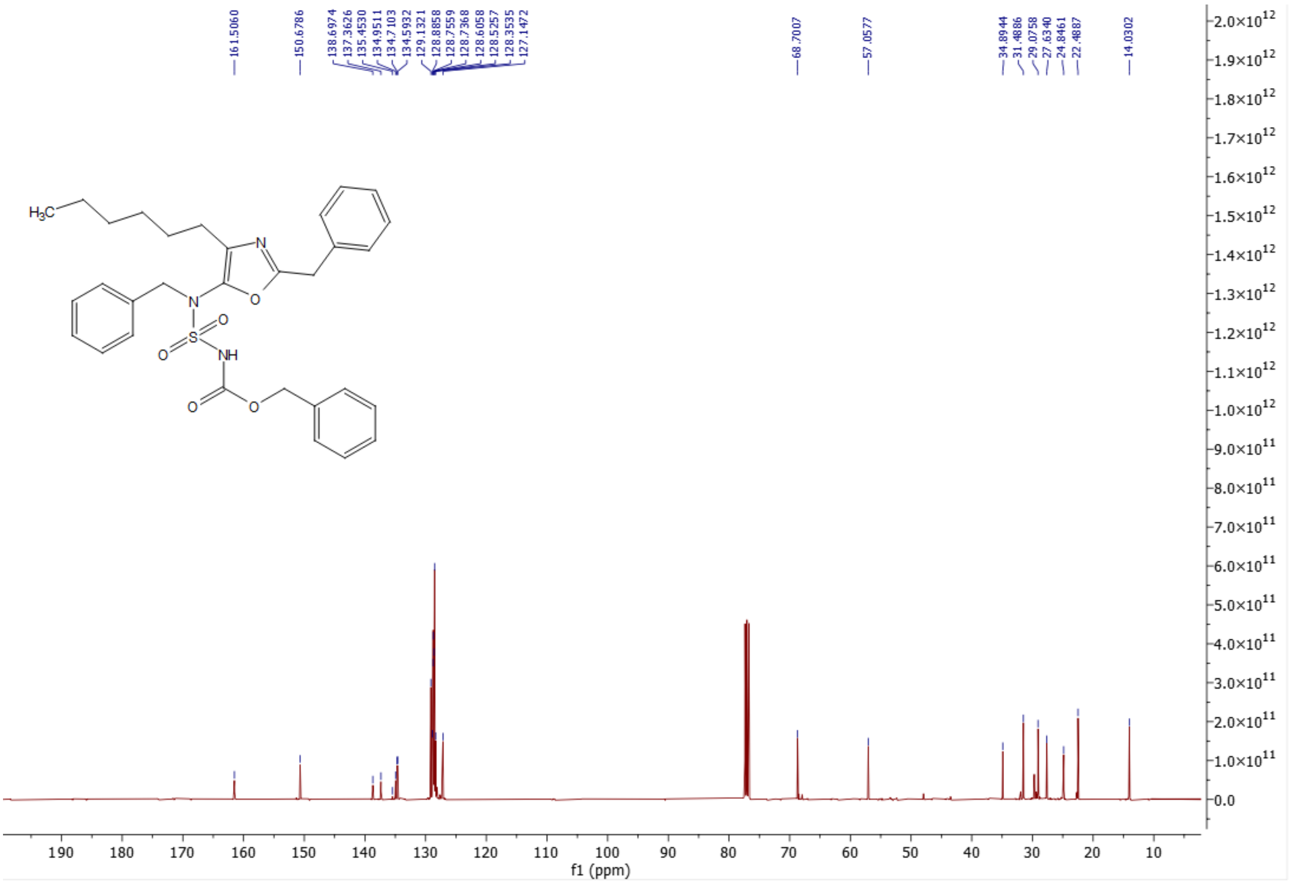

15a: ^{1}H (400 MHz, $CDCl_3$), ^{13}C (101 MHz, $CDCl_3$)

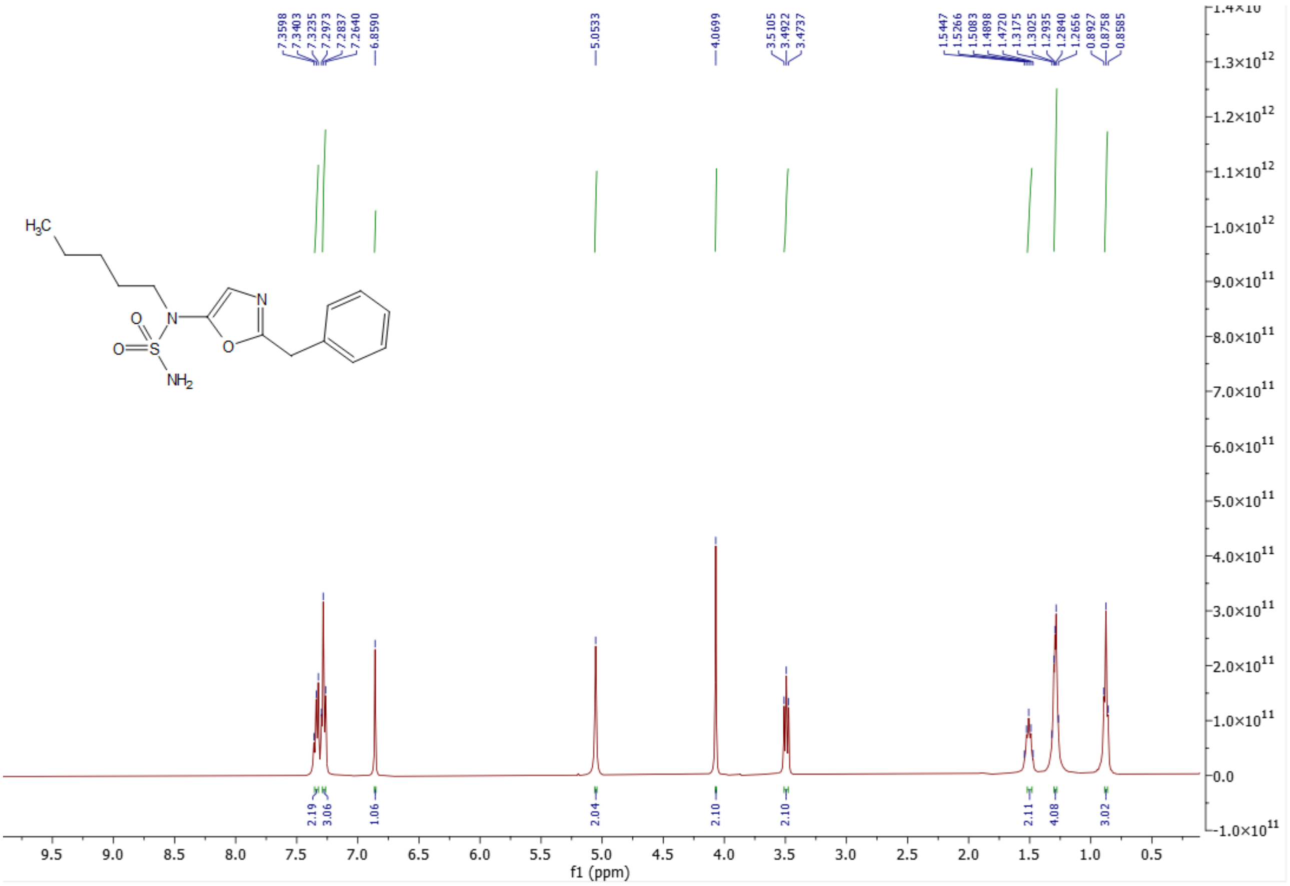

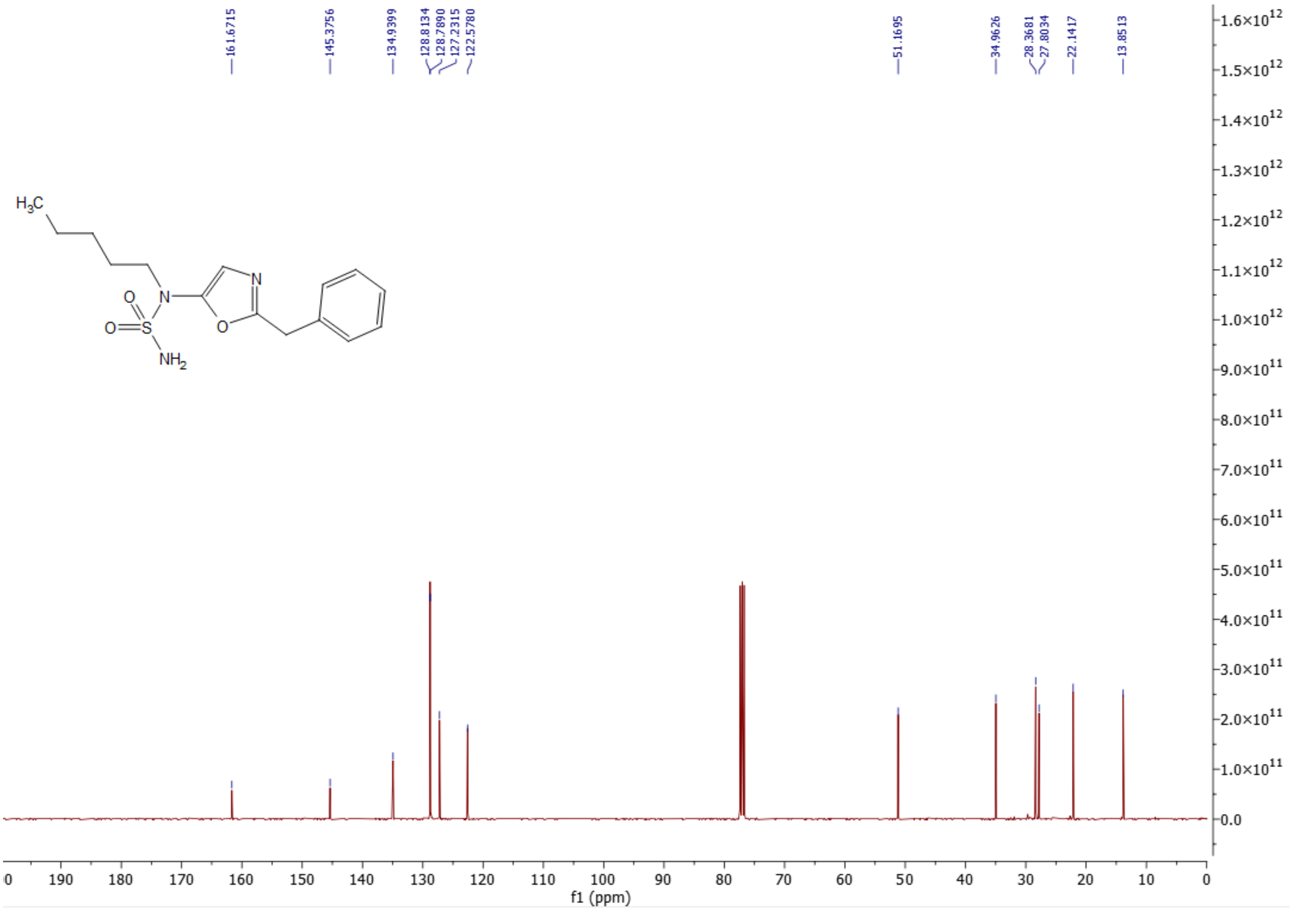

15b: ^{1}H (400 MHz, $CDCl_3$), ^{13}C (101 MHz, $CDCl_3$)

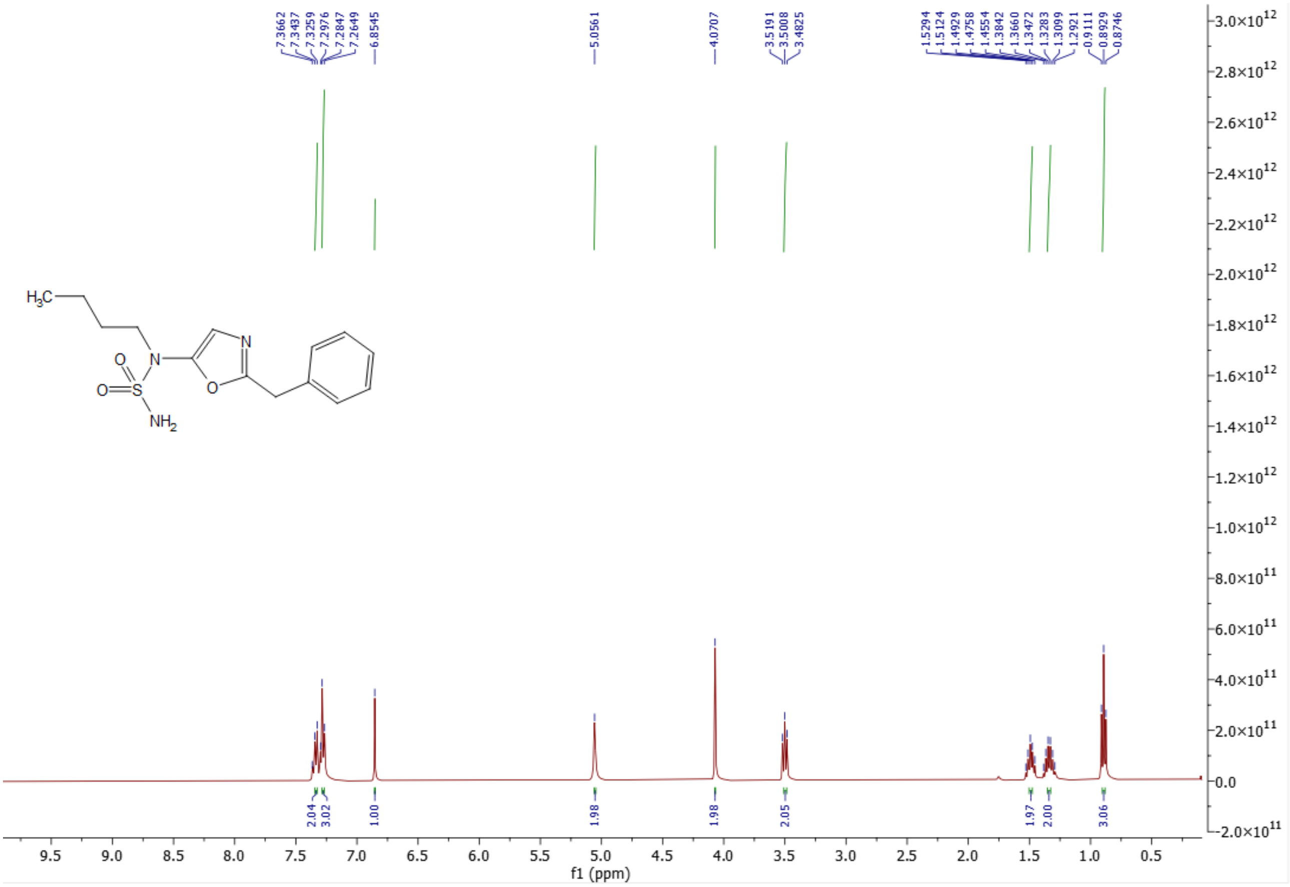

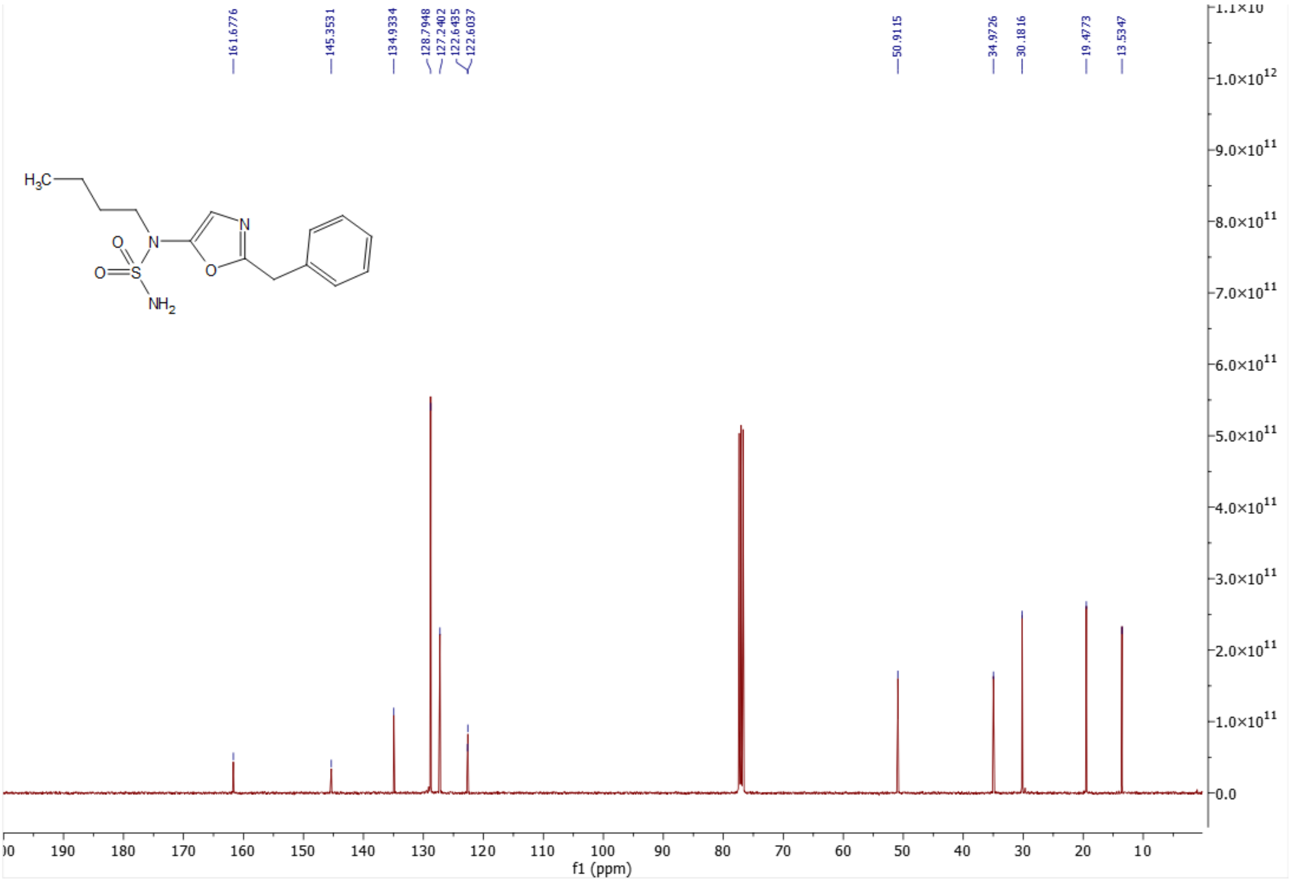

15f: ^{1}H (400 MHz, $(CD_3)_2CO$)), ^{13}C (101 MHz, $(CD_3)_2CO$)

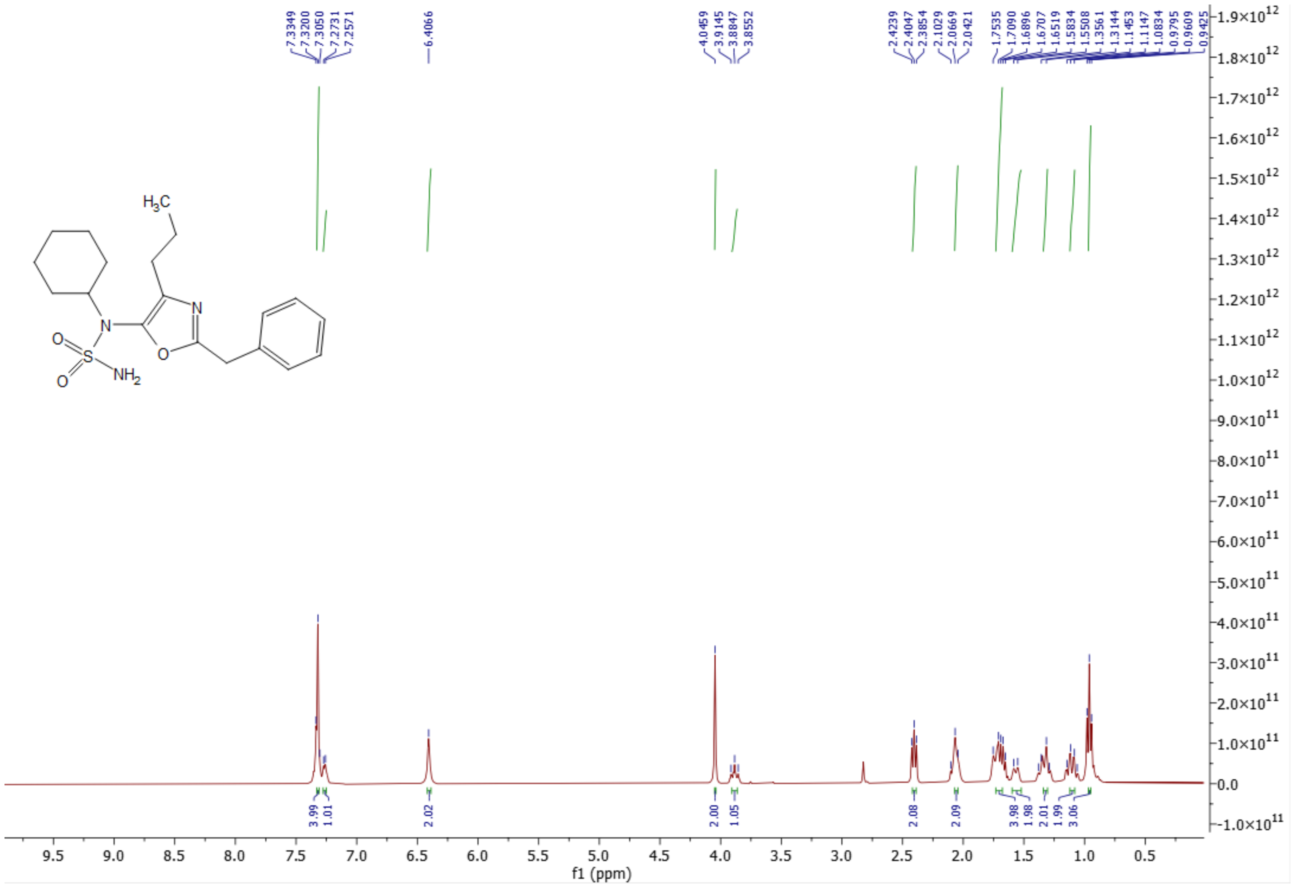

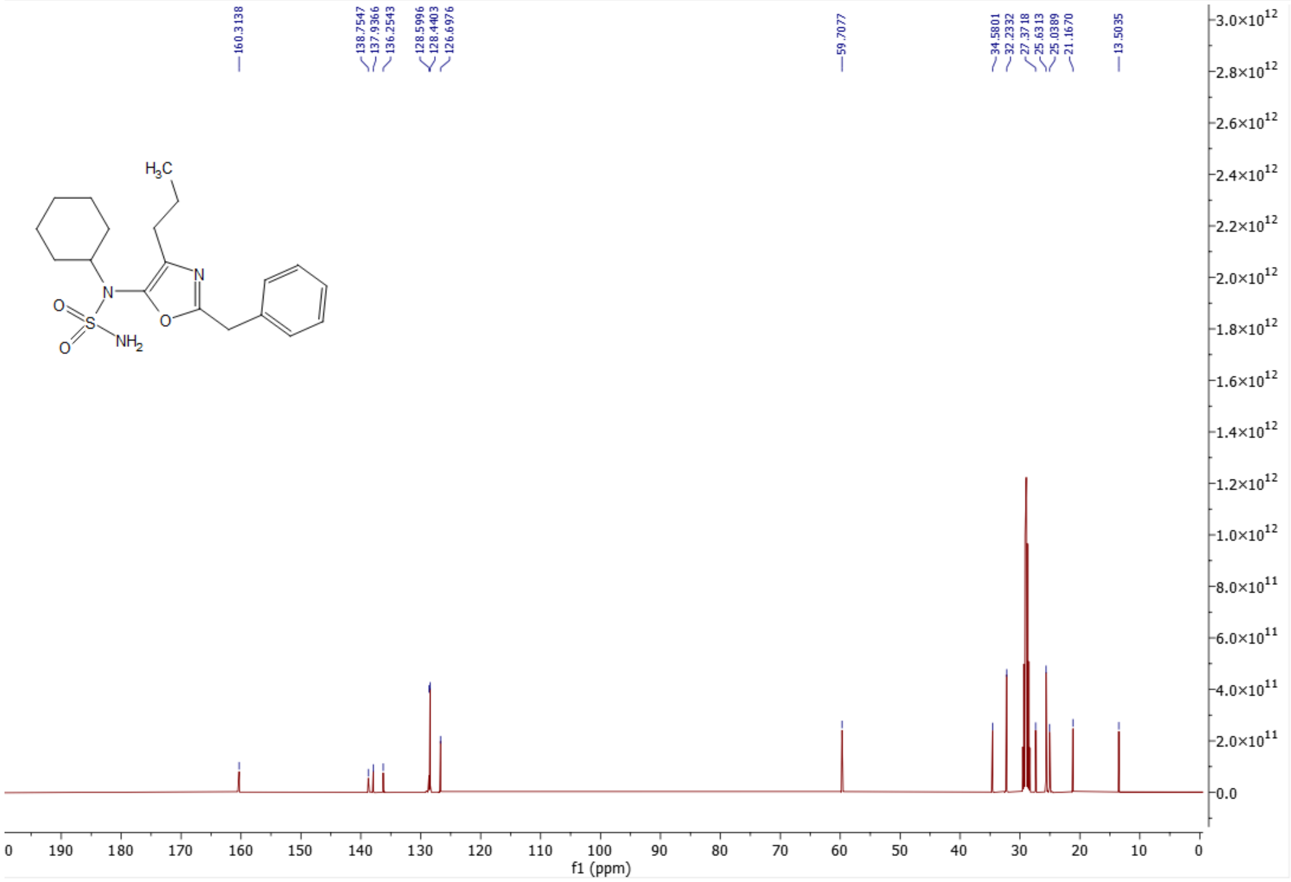

15h: ^{1}H (400 MHz, $(CD_3)_2CO$)), ^{13}C (101 MHz, $(CD_3)_2CO$)

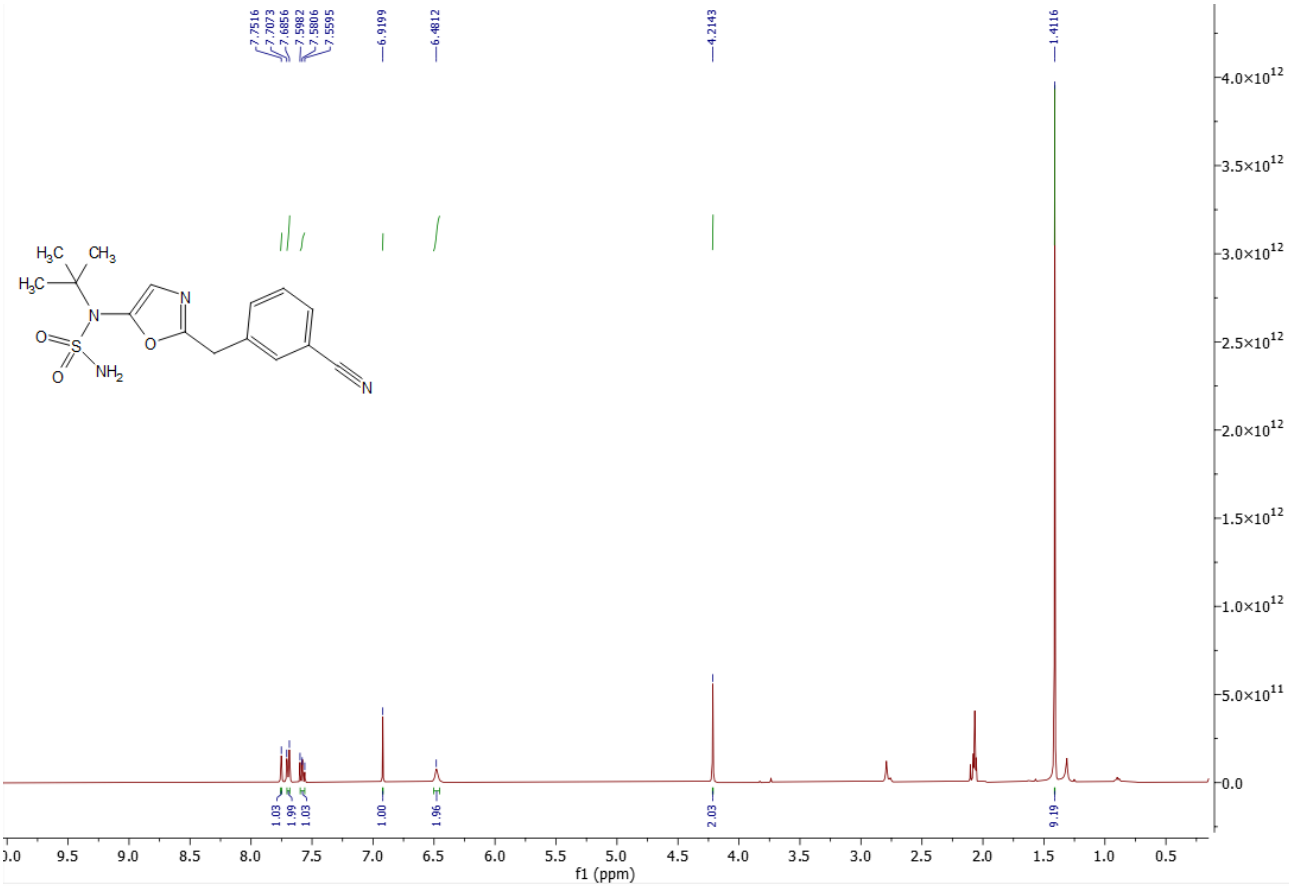

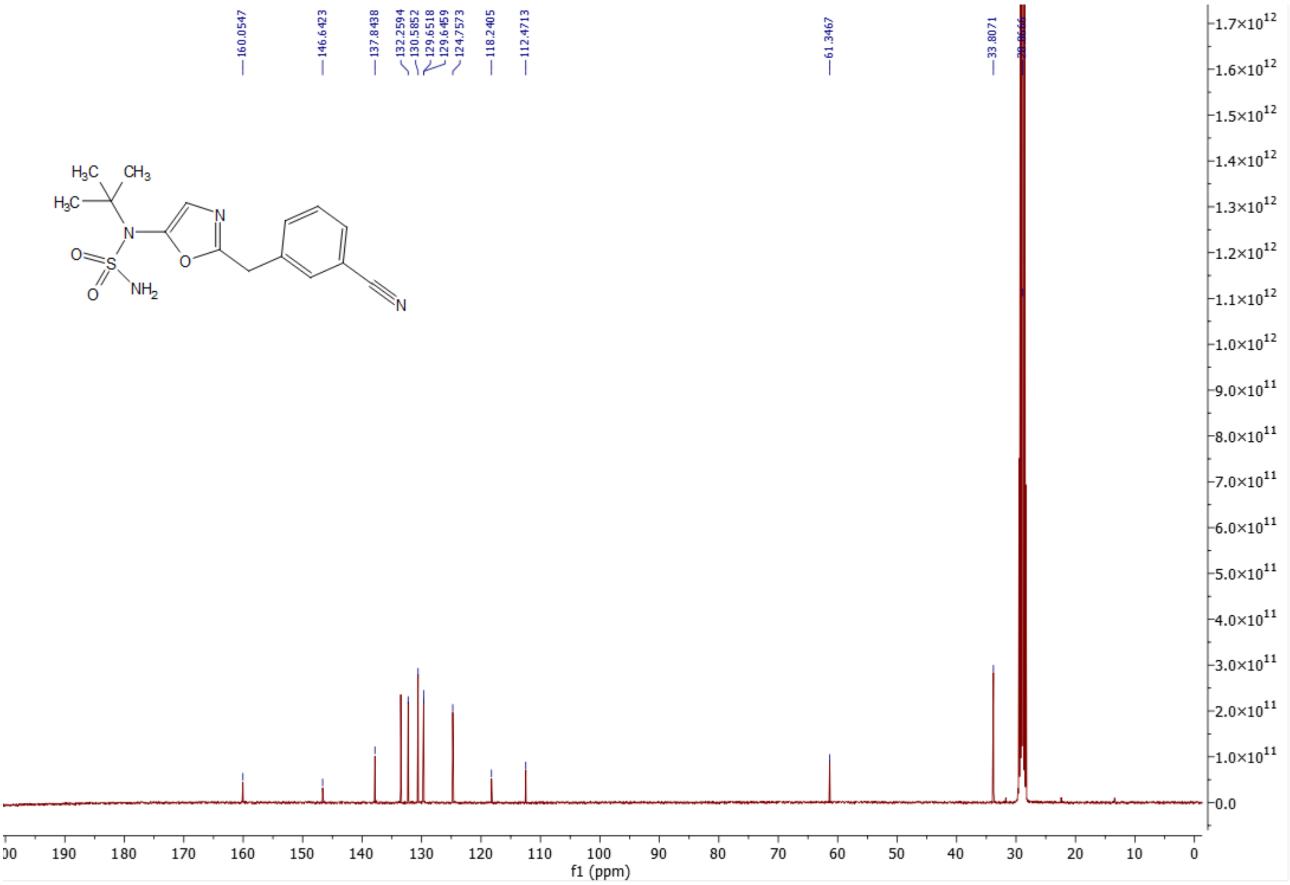

15j: ^{1}H (400 MHz, CD_3OD), ^{13}C (101 MHz, $CDCl_3$)

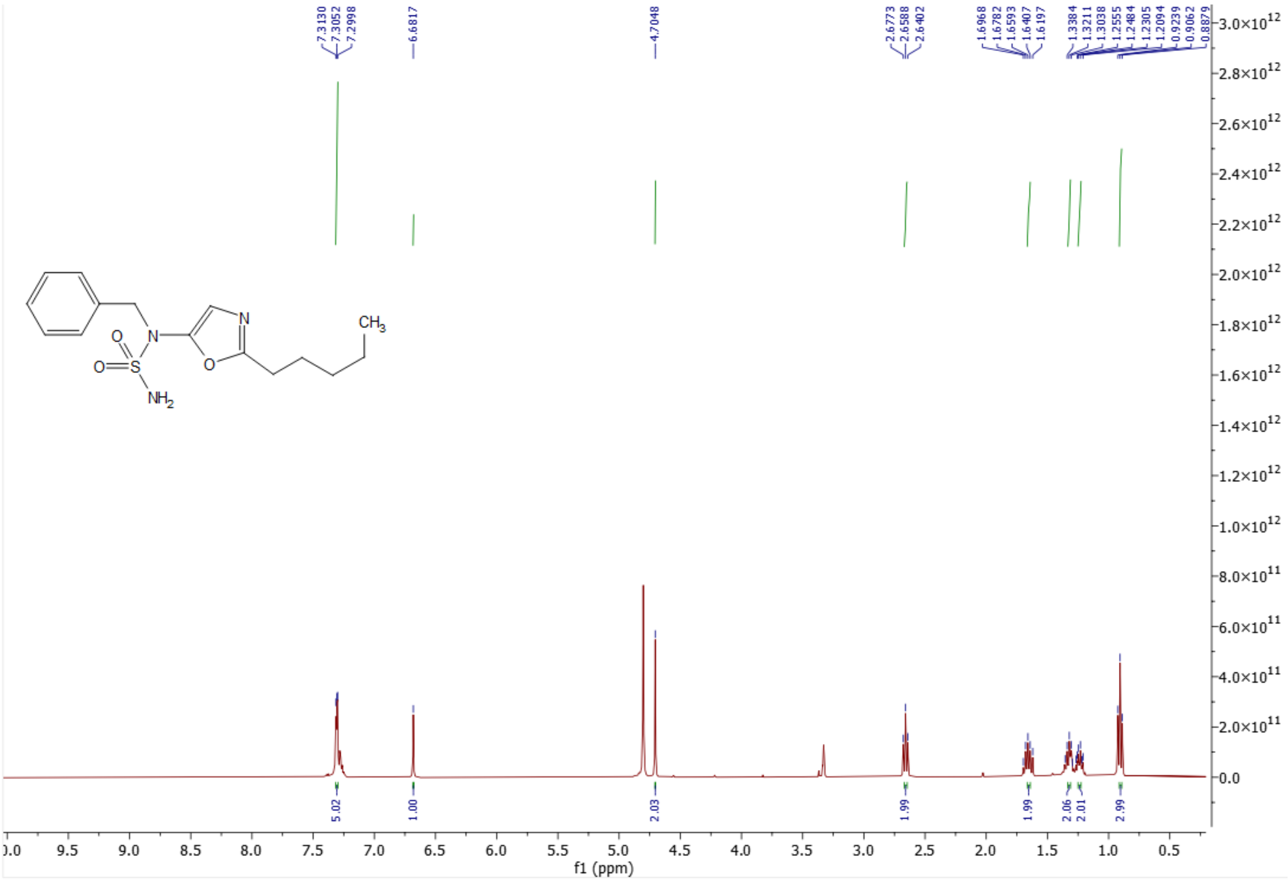

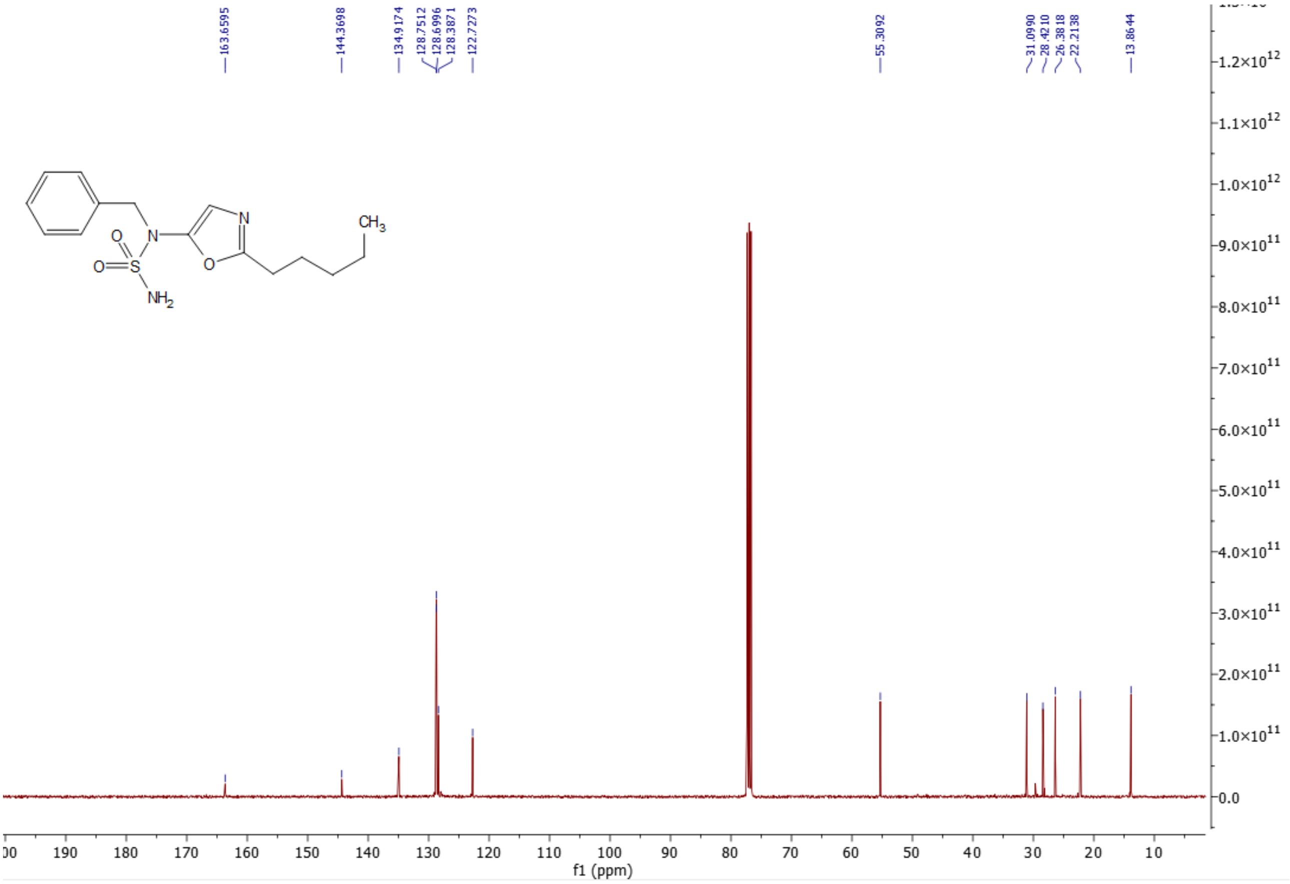

15k: ^{1}H (400 MHz, $(CD_3)_2CO$)), ^{13}C (101 MHz, $(CD_3)_2CO$)

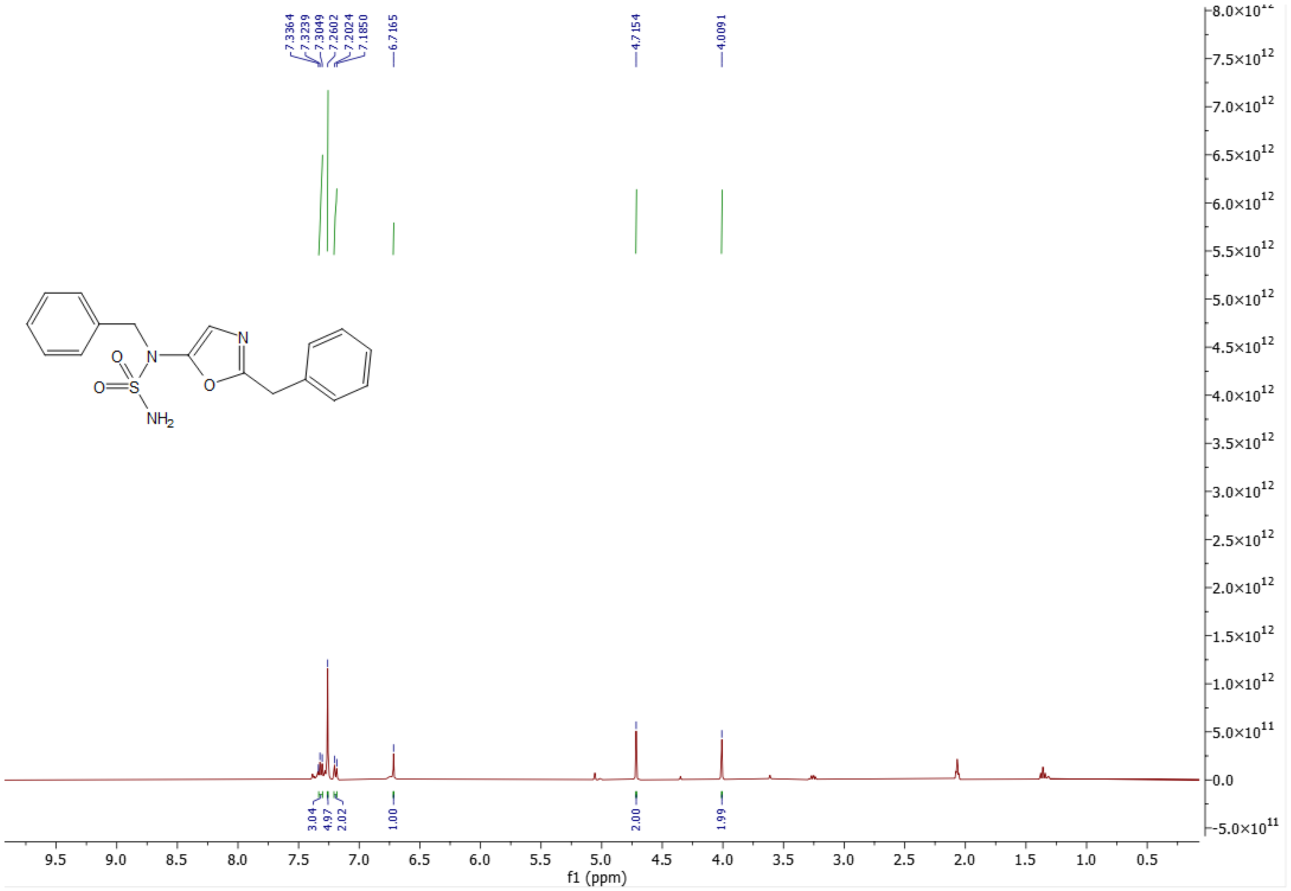

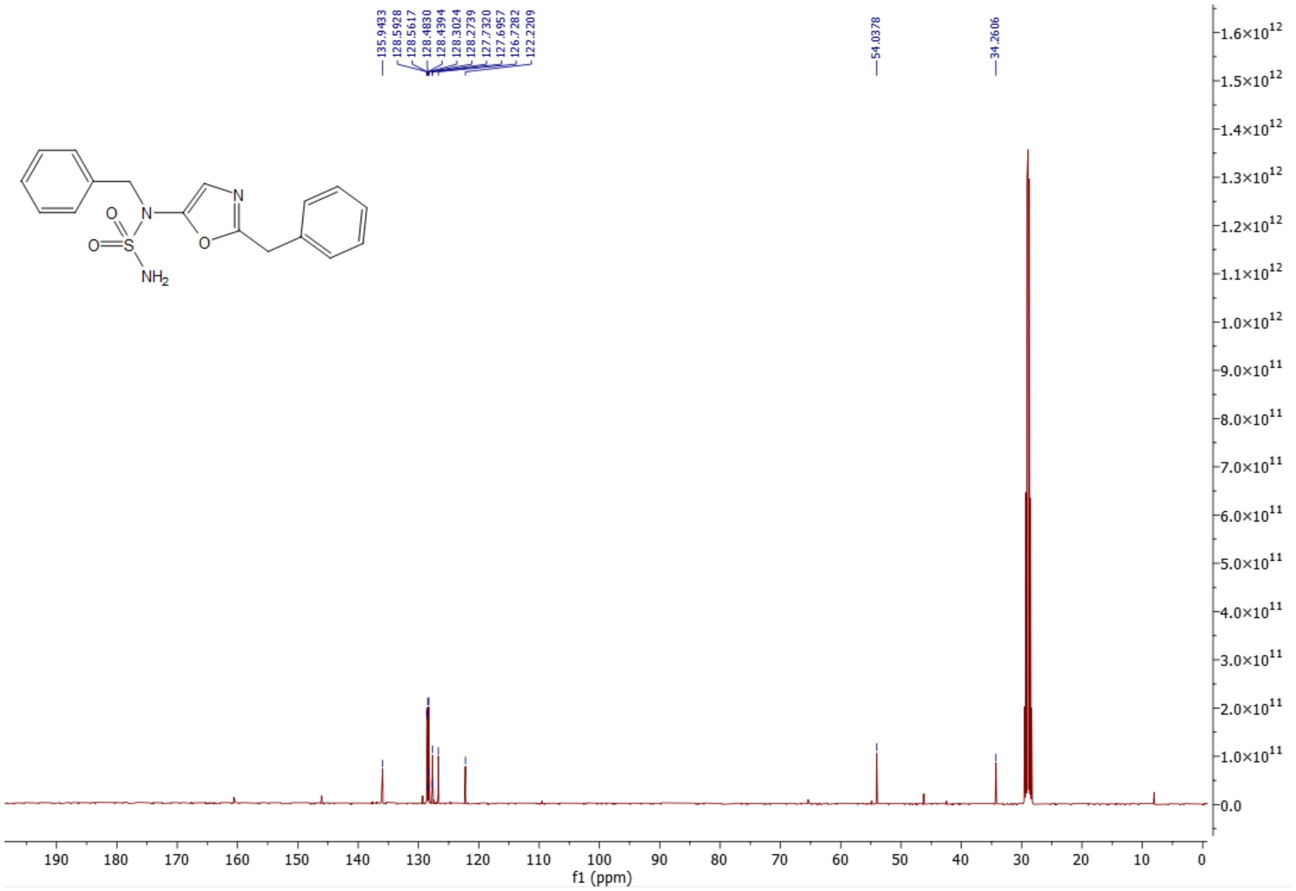

15l: ^{1}H (400 MHz, CDCl$_3$), ^{13}C (101 MHz, CDCl$_3$)

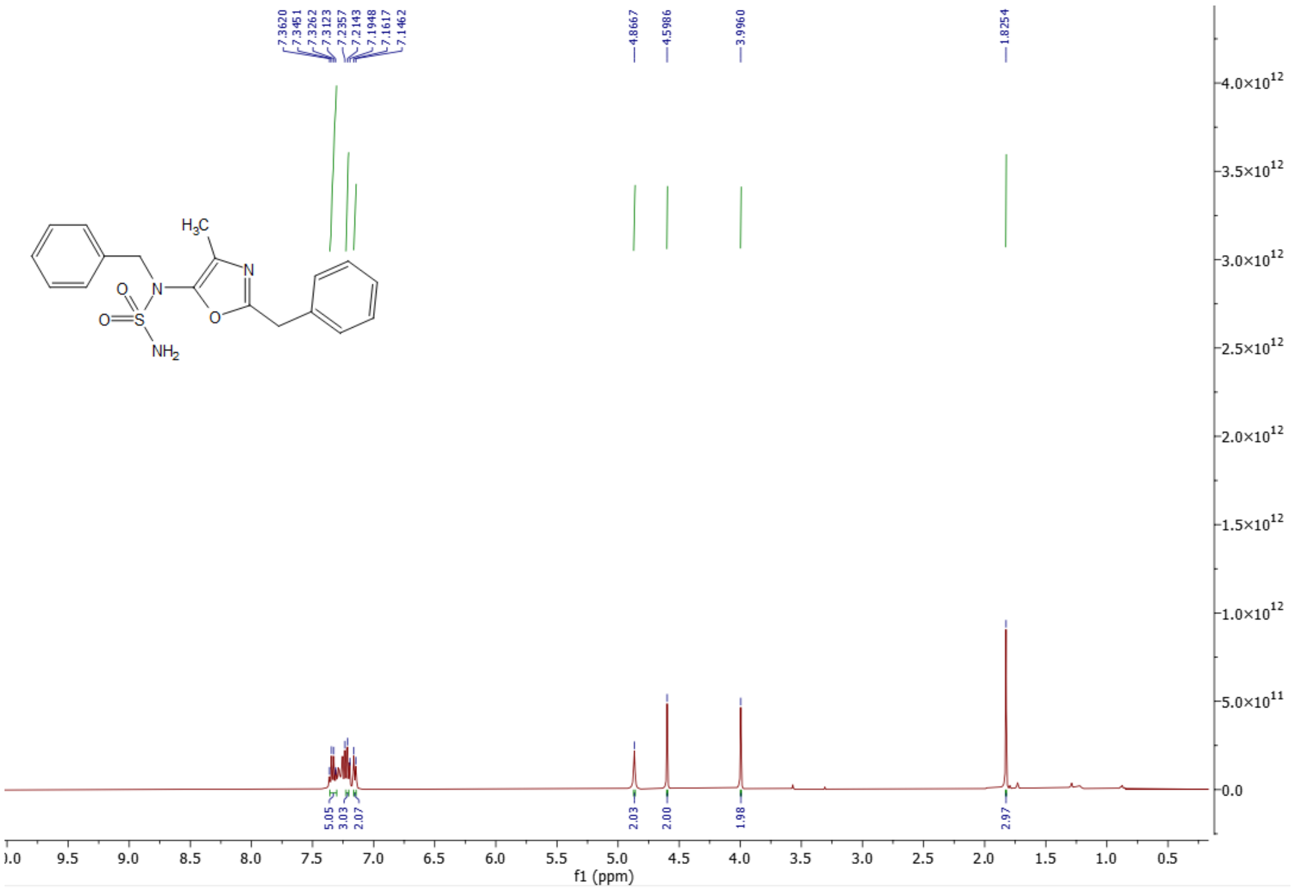

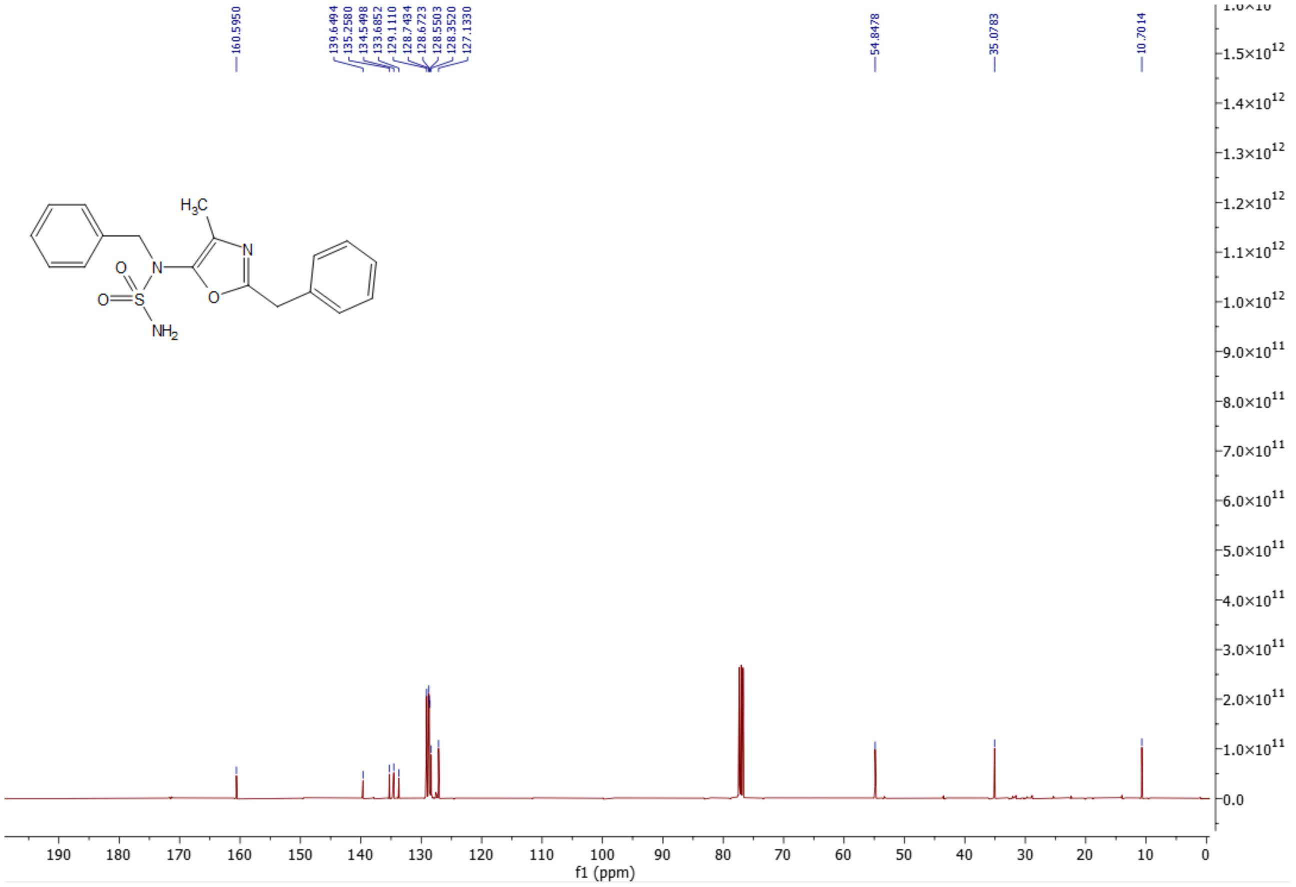

References

1) Yuan, Y.; Liu, H.; Hu, X.; Wei, Y.; Shi, M. Visible-light-induced trifluoromethylation of isonitrile-substituted methylenecyclopropanes: facile access to 6-(Trifluoromethyl)-7,8-dihydrobenzo[k]phenanthridine derivatives. *Chemistry* **2016**, *22*, 13059-13063.

2) Serafini, M.; Torre, E.; Aprile, S.; Del Grosso, E.; Gesù, A.; Griglio, A.; Colombo, G.; Travelli, C.; Paiella, S.; Adamo, A.; Orecchini, E.; Coletti, A.; Pallotta, M. T.; Ugel, S.; Massarotti, A.; Pirali, T.; Fallarini, S. Discovery of highly potent benzimidazole derivatives as indoleamine 2,3-dioxygenase-1 (IDO1) inhibitors: from structure-based virtual screening to *in vivo* pharmacodynamic activity. *J. Med. Chem.* **2020**, *63*, 3047-3065.

3) Serafini, M.; Griglio, A.; Oberto, E.; Pirali, T.; Tron, G. T. The use of 2-Hydroxymethyl benzoic acid as an effective water surrogate in the Passerini reaction: a straightforward access to α-hydroxyamides. *Tetrahedron Lett*. **2017**, *58*, 4786-4789.

4) Nicolaou, K. C.; Snyder, S. A.; Longbottom, D. A.; Nalbandian, A. Z.; Huang, X. New uses for the Burgess reagent in chemical synthesis: methods for the facile and stereoselective formation of sulfamidates, glycosylamines, and sulfamides. *Chemistry* **2004**, *10*, 5581-5606.
